# Supplementary material for: Disubstituted Meldrum’s Acid: Another Scaffold with SuFEx-like Reactivity
Source: Molecules. 2025 Aug 29;30(17):3534. doi: 10.3390/molecules30173534 (PMC12430710; doi:10.3390/molecules30173534)
Supplement: Supplementary file 1 [file molecules-30-03534-s001.zip › molecules-3831127-supplementary.pdf]

Disubstituted Meldrum's Acid: Another Scaffold with SuFEx-like Reactivity

Baoqi Chen, Zhenguo Wang, Xiaole Peng, Jijun Xie, Zhixiu Sun and Le Li*

PCFM Lab and GDHPRC Lab, School of Chemistry, Sun Yat-sen University, Guangzhou 510275, P. R. China; benchan93@163.com (B.C.); wangzhg28@mail2.sysu.edu.cn (Z.W.); pengxle@mail2.sysu.edu.cn (X.P.); xiejj35@mail2.sysu.edu.cn (J. X.); sunzx1996@163.com (Z.S.)

* Correspondence: lile26@mail.sysu.edu.cn

Supporting Information

Table of Content

1. General Information *2*

2. Synthesis of Starting Materials *2*

3. Reaction Optimizations *8*

4. Synthesis and Characterization Data *10*

5. Preparation-scale Derivatization of Acetaminophen *26*

6. Preliminary Mechanistic Experiments *28*

7. References *30*

8. NMR Spectra *31*

1. *General Information*

Materials. All reagents were purchased from commercial sources (Energy Chemical, Shanghai, China) and used without further purification unless otherwise noted. Acetonitrile, *N*-methyl-pyrrolidinone and dimethyl sulfoxide used in the reactions were anhydrous solvents purchased from commercial suppliers (Energy Chemical, Shanghai, China) and used without further drying (≥99.9%, LC-MS, Energy Chemical, China). The petroleum ether (Energy Chemical, Shanghai, China) used was in a boiling range of 60–90 °C. Other solvents were purified according to standard procedures.[1] All reactions were carried out with oven-dried glassware and monitored by thin-layer chromatography (0.20 mm HP-TLC silica gel 60 GF-254 plates, Leyan, Shanghai, China). Visualization was accomplished with UV light, and/or potassium permanganate, or 2% ninhydrin in ethanol stain followed by heating. Flash column chromatography was performed on 200–300 mesh silica gel (Leyan, Shanghai, China). Meldrum's acid (2,2-dimethyl-1,3-dioxane-4,6-dione) and 5-methyl Meldrum's acid (2,2,5-trimethyl-1,3-dioxane-4,6-dione) were purchased from Bide Pharm, Shanghai China, and used without further purification. The deuterated chloroform ($CDCl_3$) (Energy Chemical, Shanghai, China) used contains 0.03% (*v/v*) of tetramethylsilane (TMS).

Instrumentation. ^{1}H, ^{19}F, and ^{13}C NMR spectra were recorded on a Bruker AVANCE III 400 MHz spectrometer (Bruker, Billerica, MA, USA) at 298 K and referenced to residual protium in the NMR solvent ($CDCl_3$, δ 7.26, DMSO-d_6, 2.50 in ^{1}H NMR) and the carbon resonances of the solvent ($CDCl_3$, δ 77.16, DMSO-d_6, 39.52 in ^{13}C NMR). Chemical shifts were reported in parts per million (ppm, δ) downfield from tetramethylsilane. NMR peaks are described as singlet (s), doublet (d), triplet (t), quartet (q), multiplet (m), heptet (hept), complex (comp), and approximate (app). High-resolution mass spectra (HRMS) were recorded on a Thermo Fisher Scientific's Q Exactive UHMR Hybrid Quadrupole-Orbitrap Mass Spectrometer LC/MS (ESI/APCI) (Thermo Fisher, Waltham, MA, USA).

Abbreviations. TEAF = triethylammonium formate, TEA = triethylamine, DIPEA = N,N-diisopropylethylamine, DBU = 1,8-diazabicyclo[5.4.0]undec-7-ene, DMAP = 4-dimethylaminopyridine, BTMG = 2-tert-butyl-1,1,3,3-tetramethylguanidine, NMI = N-methylimidazole, DPPA = diphenyl azidophosphate, HOBt = 1-hydroxybenzotriazole, EDC = 1-ethyl-3-(3-dimethylaminopropyl)carbodiimide, EA = ethyl acetate, DMF = *N,N*-dimethylformamide, DCM = dichloromethane, DCE = dichloroethane, DMSO = dimethyl sulfoxide, THF = tetrahydrofuran, NMP = 1-methyl-2-pyrrolidinone, TLC = thin-layer chromatography, *v/v* = volume per volume, equiv = equivalent, w/o = without, rt = room temperature.

2. *Synthesis of Starting Materials*

2.1. A general procedure A for the 5-substituted-5-methyl-1,3-dioxane-4,6-dione

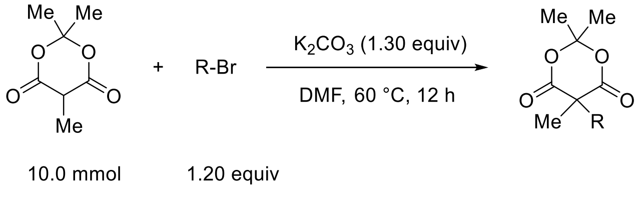

Substituted-Meldrum's acids were prepared according to the literature procedure.[2] 5-methyl Meldrum's acid (1.00 equiv) and K_2CO_3 (1.30 equiv) were dissolved in anhydrous DMF, followed by the addition of alkyl bromide (1.20 equiv). The mixture was then heated to 60 °C for 12 hours. After reaction completion,

the mixture was diluted with 50 mL of EA and 50 mL of water. The organic phase was separated. The aqueous phase was further extracted with 2 × 50 mL of EA. The combined organic layer was separated, washed with saturated aqueous $NaHCO_3$ solution and saturated aqueous NaCl solution, dried over anhydrous sodium sulfate, and concentrated in vacuo to afford the crude product. The crude product was purified by flash column chromatography to afford the purified product.

*2,2,5,5-Tetramethyl-1,3-dioxane-4,6-dione (**1a**)*

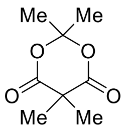

Compound **1a** was prepared according to a modification of the literature.[3] To a solution of Meldrum's acid (1.00 g, 1.00 equiv, 6.94 mmol) in anhydrous DMF (10 mL) in a septum-capped round bottom flask was added DIPEA (2.41 mL, 2.00 equiv, 13.87 mmol) and stirred for 10 minutes. After the addition of methyl iodide (1.30 mL, 3.00 equiv, 20.81 mmol), the resulting reaction mixture was stirred at 60 °C for 2 hours. The reaction solution was concentrated and subjected to silica gel column chromatography (petroleum ether/ethyl acetate = 10/1, *v/v*) to afford 2,2,5,5-tetramethyl-1,3-dioxane-4,6-dione (**1a**) as white solid (1.01 g, 85%).

The NMR data were in agreement with the literature data.[4]

*5-Isopropyl-2,2,5-trimethyl-1,3-dioxane-4,6-dione (**1b**)*

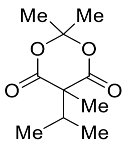

Compound **1b** was prepared according to a modification procedure of the literature procedure.[5] In a round-bottom flask, acetone (0.73 mL, 10.0 mmol, 1.00 equiv) were dissolved in TEAF (TEA/formic acid 5:2 mol/mol) solution (10 mL) prepared by mixing triethylamine and formic acid. After addition of Meldrum's acid (1.44 g, 1.0 mmol, 1.00 equiv), the mixture was stirred at 60 °C (oil bath), and monitored by TLC analysis (around 6 hours). Then, it was cooled to rt, and then poured into ice water (pH = 2–3), leading to the precipitation of 5-isopropyl Meldrum's acid. It can be used directly for next step. Then, the corresponded product and K_2CO_3 (1.80 g, 13.0 mmol, 1.30 equiv) were dissolved in anhydrous DMF (20 mL), followed by the addition of iodomethane (0.75 mL, 12.0 mmol, 1.20 equiv). The mixture was then heated to 60 °C for 12 hours. After reaction completion, the mixture was diluted with 50 mL of EA and 50 mL of water. The organic phase was separated. The aqueous phase was further extracted with 2 × 50 mL of EA. The combined organic layer was separated, washed with saturated aqueous $NaHCO_3$ solution and saturated aqueous NaCl solution, dried over anhydrous sodium sulfate, and concentrated in vacuo to afford the crude product. The crude product was purified by flash column chromatography (petroleum ether/ethyl acetate = 10/1, *v/v*) to afforded 5-isopropyl-2,2,5-trimethyl-1,3-dioxane-4,6-dione (**1b**) as a colorless oil (1.20 g, 60%).

$\mathbf{R}_f$ = 0.30 (petroleum ether/ethyl acetate = 10/1, *v/v*).

^{1}H NMR (400 MHz, CDCl3) δ 2.29 (hept, J = 6.9 Hz, 1H), 1.68 (s, 3H), 1.64 (s, 3H), 1.50 (s, 3H), 0.99 (d, J = 6.9 Hz, 6H).

^{13}C NMR (101 MHz, $CDCl_3$) δ 170.3 (2C), 104.7, 53.5, 39.3, 30.6, 28.0, 20.3, 18.1 (2C).

*5-Isobutyl-2,2,5-trimethyl-1,3-dioxane-4,6-dione (**1c**)*

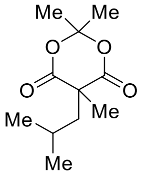

Compound **1c** was prepared according to the general procedure A using 5-methyl Meldrum's acid (1.58 g, 10.00 mmol, 1.00 equiv) and 1-bromo-2-methylpropane (1.30 mL, 12.0 mmol, 1.20 equiv). Purification by flash column chromatography (petroleum ether/ethyl acetate = 10/1, *v/v*) afforded 5-isobutyl-2,2,5-trimethyl-1,3-dioxane-4,6-dione(**1c**) as a white solid (1.60 g, 66%).

R_f= 0.30 (petroleum ether/ethyl acetate = 10/1, *v/v*).

^{1}H NMR (400 MHz, $CDCl_3$) δ 2.00 (d, *J* = 6.7 Hz, 2H), 1.76 (s, 3H), 1.73 (s, 3H), 1.67 – 1.56 (comp, 4H), 0.88 (d, *J* = 6.7 Hz, 6H).

^{13}C NMR (101 MHz, $CDCl_3$) δ 170.5 (2C), 104.9, 49.9, 48.2, 30.0, 28.3, 25.8, 25.6, 22.9 (2C).

*2,2,5-Trimethyl-5-(prop-2-yn-1-yl)-1,3-dioxane-4,6-dione (**1d**)*

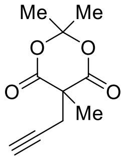

Compound **1d** was prepared according to the general procedure A using 5-methyl Meldrum's acid (1.58 g, 10.00 mmol, 1.00 equiv) and 3-bromoprop-1-yne (1.03 mL, 12.0 mmol, 1.20 equiv). Purification by flash column chromatography (petroleum ether/ethyl acetate = 10/1, *v/v*) afforded 2,2,5-trimethyl-5-(prop-2-yn-1-yl)-1,3-dioxane-4,6-dione (**1d**) as a white solid (1.28 g, 65%).

R_f= 0.30 (petroleum ether/ethyl acetate = 10/1, *v/v*).

^{1}H NMR (400 MHz, $CDCl_3$) δ 2.92 – 2.83 (m, 2H), 2.14 – 2.07 (m, 1H), 1.82 (s, 3H), 1.77 (s, 3H), 1.64 (s, 3H).

^{13}C NMR (101 MHz, $CDCl_3$) δ 169.1 (2C), 105.7, 78.4, 72.4, 49.7, 29.4, 29.2, 28.3, 24.9.

*5-(Cyclohexylmethyl)-2,2,5-trimethyl-1,3-dioxane-4,6-dione (**1e**)*

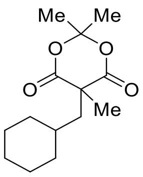

Compound **1e** was prepared according to the general procedure A using 5-methyl Meldrum's acid (1.58 g, 10.00 mmol, 1.00 equiv) and (bromomethyl)cyclohexane (1.67 mL, 12.0 mmol, 1.20 equiv). Purification by flash column chromatography (petroleum ether/ethyl acetate = 10/1, *v/v*) afforded 5-(cyclohexylmethyl)-2,2,5-trimethyl-1,3-dioxane-4,6-dione(**1e**) as a white solid (1.10 g, 43%).

R_f= 0.30 (petroleum ether/ethyl acetate = 10/1, *v/v*).

^{1}H NMR (400 MHz, $CDCl_3$) δ 2.02 (d, J = 6.5 Hz, 2H), 1.78 (s, 3H), 1.76 (s, 3H), 1.72 – 1.57 (comp, 8H), 1.42 – 1.24 (m, 2H), 1.24 – 1.03 (comp, 3H), 1.03 – 0.86 (m, 2H).

^{13}C NMR (101 MHz, $CDCl_3$) δ 170.5 (2C), 104.9, 48.6, 47.9, 35.1, 33.4 (2C), 29.9, 28.4, 25.9 (2C), 25.9, 25.5.

*5-Benzyl-2,2,5-trimethyl-1,3-dioxane-4,6-dione (**1f**)*

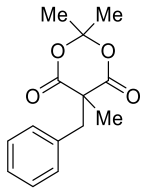

Compound **1f** was prepared according to the general procedure A using 5-methyl Meldrum's acid (1.58 g, 10.00 mmol, 1.00 equiv) and benzyl bromide (1.42 mL, 12.0 mmol, 1.20 equiv). Purification by flash column chromatography (petroleum ether/ethyl acetate = 10/1, *v/v*) afforded 5-benzyl-2,2,5-trimethyl-1,3-dioxane-4,6-dione (**1f**) as a white solid (2.00 g, 78%).

R_f = 0.30 (petroleum ether/ethyl acetate = 10/1, *v/v*).

^{1}H NMR (400 MHz, CDCl3) δ 7.43 – 7.05 (comp, 5H), 3.33 (s, 2H), 1.75 (s, 3H), 1.59 (s, 3H), 0.90 (s, 3H).

^{13}C NMR (101 MHz, $CDCl_3$) δ 169.9 (2C), 135.5, 130.2 (2C), 128.9 (2C), 127.9, 105.4, 52.4, 45.0, 29.5, 28.4, 26.0.

*5-(4-Fluorobenzyl)-2,2,5-trimethyl-1,3-dioxane-4,6-dione (**1g**)*

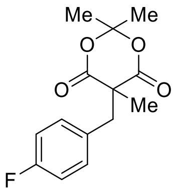

Compound **1g** was prepared according to the general procedure A using 5-methyl Meldrum's acid (1.58 g, 10.00 mmol, 1.00 equiv) and 1-(bromomethyl)-2-fluorobenzene (1.44 mL, 12.0 mmol, 1.20 equiv). Purification by flash column chromatography (petroleum ether/ethyl acetate = 10/1, *v/v*) afforded 5-(4-fluorobenzyl)-2,2,5-trimethyl-1,3-dioxane-4,6-dione (**1g**) as a white solid (1.36 g, 51%).

R_f = 0.30 (petroleum ether/ethyl acetate = 10/1, *v/v*).

^{1}H NMR (400 MHz, $CDCl_3$) δ 7.18 – 7.13 (m, 2H), 7.01 – 6.91 (m, 2H), 3.30 (s, 2H), 1.75 (s, 3H), 1.62 (s, 3H), 1.02 (s, 3H).

^{19}F NMR (377 MHz, $CDCl_3$) δ –114.3.

^{13}C NMR (101 MHz, $CDCl_3$) δ 169.8 (2C), 162.4 (d, J_{C-F} = 246.8 Hz), 131.8 (d, J_{C-F} = 8.1 Hz, 2C), 131.1 (d, J_{C-F} = 3.3 Hz), 115.6 (d, J_{C-F} = 21.2 Hz, 2C), 105.3, 52.2, 44.0, 29.4, 28.4, 25.7.

*5-(3-Fluorobenzyl)-2,2,5-trimethyl-1,3-dioxane-4,6-dione (**1h**)*

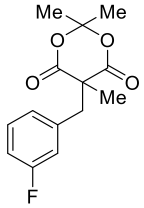

Compound **1h** was prepared according to the general procedure A using 5-methyl Meldrum's acid (1.58 g, 10.00 mmol, 1.00 equiv) and 1-(bromomethyl)-3-fluorobenzene (1.47 mL, 12.0 mmol, 1.20 equiv). Purification by flash column chromatography (petroleum ether/ethyl acetate = 10/1, *v/v*) afforded 5-(3-fluorobenzyl)-2,2,5-trimethyl-1,3-dioxane-4,6-dione (**1h**) as a white solid (1.75 g, 66%).

$\mathbf{R}_{\boldsymbol{f}}$= 0.30 (petroleum ether/ethyl acetate = 10/1, *v/v*).

^{1}H NMR (400 MHz, $CDCl_3$) δ 7.28 – 7.21 (m, 1H), 7.02 – 6.88 (comp, 3H), 3.34 (s, 2H), 1.77 (s, 3H), 1.64 (s, 3H), 1.04 (s, 3H).

^{19}F NMR (377 MHz, $CDCl_3$) δ –112.3.

^{13}C NMR (101 MHz, $CDCl_3$) δ 169.6 (2C), 162.8 (d, J = 247.0 Hz), 137.7 (d, $J_{C\text{–}F}$ = 7.2 Hz), 130.3 (d, $J_{C\text{–}F}$ = 8.3 Hz), 125.8 (d, $J_{C\text{–}F}$ = 3.0 Hz), 117.1 (d, $J_{C\text{–}F}$ = 21.5 Hz), 114.8 (d, $J_{C\text{–}F}$ = 21.0 Hz), 105.3, 52.0, 44.3 (d, $J_{C\text{–}F}$ = 1.9 Hz), 29.3, 28.5, 26.0.

*5-(2-Fluorobenzyl)-2,2,5-trimethyl-1,3-dioxane-4,6-dione (**1i**)*

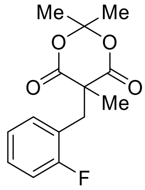

Compound **1i** was prepared according to the general procedure A using 5-methyl Meldrum's acid (1.58 g, 10.00 mmol, 1.00 equiv) and 1-(bromomethyl)-2-fluorobenzene (1.44 mL, 12.0 mmol, 1.20 equiv). Purification by flash column chromatography (petroleum ether/ethyl acetate = 10/1, *v/v*) afforded 5-(2-fluorobenzyl)-2,2,5-trimethyl-1,3-dioxane-4,6-dione (**1i**) as a white solid (1.75 g, 62%).

$\mathbf{R}_{\boldsymbol{f}}$= 0.30 (petroleum ether/ethyl acetate = 10/1, *v/v*).

^{1}H NMR (400 MHz, $CDCl_3$) δ 7.29 – 7.16 (comp, 2H), 7.12 – 6.99 (comp, 2H), 3.39 (s, 2H), 1.74 (s, 3H), 1.64 (s, 3H), 1.22 (s, 3H).

^{19}F NMR (377 MHz, $CDCl_3$) δ –114.9.

^{13}C NMR (101 MHz, $CDCl_3$) δ 169.5 (2C), 161.1 (d, $J_{C\text{–}F}$ = 248.3 Hz), 132.1 (d, $J_{C\text{–}F}$ = 3.9 Hz), 129.8 (d, $J_{C\text{–}F}$ = 8.1 Hz), 124.3 (d, $J_{C\text{–}F}$ = 3.7 Hz), 122.0 (d, $J_{C\text{–}F}$ = 15.5 Hz), 115.7 (d, $J_{C\text{–}F}$ = 22.4 Hz), 105.2, 50.6, 38.7 (d, $J_{C\text{–}F}$ = 1.9 Hz), 29.8, 28.1, 24.4.

*2,2,5-Trimethyl-5-(4-(trifluoromethyl)benzyl)-1,3-dioxane-4,6-dione (**1j**)*

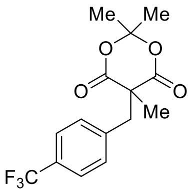

Compound **1j** was prepared according to the general procedure A using 5-methyl Meldrum's acid (1.58 g, 10.00 mmol, 1.00 equiv) and 1-(chloromethyl)-4-(trifluoromethyl)benzene (1.77 mL, 12.0 mmol, 1.20 equiv). Purification by flash column chromatography (petroleum ether/ethyl acetate = 10/1, *v/v*) afforded 2,2,5-trimethyl-5-(4-(trifluoromethyl)benzyl)-1,3-dioxane-4,6-dione (**1j**) as a white solid (1.75 g, 62%).

R_f= 0.25 (petroleum ether/ethyl acetate = 10/1, *v/v*).

^{1}H NMR (400 MHz, $CDCl_3$) δ 7.54 (d, J = 8.0 Hz, 2H), 7.31 (d, J = 8.0 Hz, 2H), 3.38 (s, 2H), 1.77 (s, 3H), 1.55 (s, 6H), 1.02 (s, 3H).

^{19}F NMR (377 MHz, $CDCl_3$) δ –62.8.

^{13}C NMR (101 MHz, $CDCl_3$) δ 169.6 (2C), 139.4, 130.6 (2C),130.2 (q, J_{C-F} = 32.9 Hz), 125.7 (q, J_{C-F} = 3.8 Hz, 2C), 124.1 (q, J_{C-F} = 272.7 Hz), 105.4, 52.1, 44.6, 29.5, 28.4, 25.8.

3. *Reaction Optimizations*

Table S1. Evaluation of bases.[a]

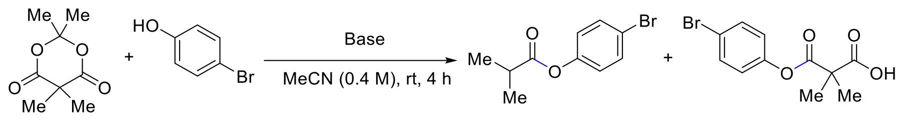

1a **2a** **3a** **3a'**

Entry	Base	Yield [%][b]
1	None	0
2	Na_2CO_3	0
3	K_2CO_3	0
4	Cs_2CO_3	4
5	K_3PO_4	0
6	TEA	34
7	DIPEA	36
8	DBU	69
9	BTMG	84
10	DMAP	23
11	NMI	0

[a]Reactions were run with **1a** (0.10 mmol), 4-bromphenol (1.10 equiv), base (1.10 equiv) in anhydrous acetonitrile 250.0 μL) at rt for 4 h. [b]Yields were determined by ^{1}H NMR with 1,3,5-trimethoxybenzene as an internal standard.

Table S2. Evaluation of solvents.[a]

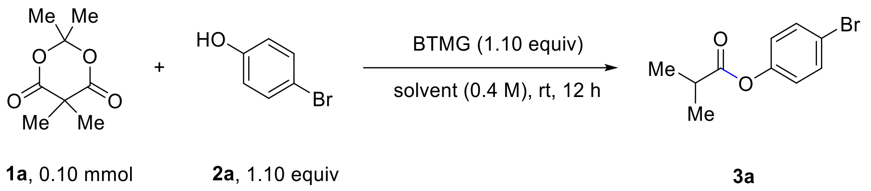

Entry	Solvent	Yield [%][b]
1	MeCN	100
2	DCM	87
3	Toluene	100
4	THF	100
5	NMP	100
6	DMSO	99
7[c]	DMSO	87
8[d]	DMSO	80

[a]Reactions were run with **1a** (0.10 mmol), 4-bromphenol (1.10 equiv), BTMG (1.10 equiv) in anhydrous solvents (250.0 μL) at rt for 12 h. [b]Yields were determined by ^{1}H NMR with 1,3,5-trimethoxybenzene as an internal standard. [c]Reactions were run in 5% H_2O–DMSO at 60 °C for 1 h. [d]Reactions were run in 20% H_2O–DMSO at 60 °C for 1 h.

4. *Synthesis and Characterization Data*

4.1 A general procedure for the preparation of the esters **3**

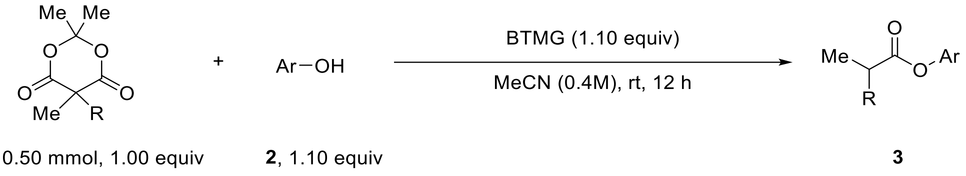

In a 4.0-mL vial, 5,5-substituted-Meldrum's acid **1** (0.50 mmol, 1.00 equiv), BTMG (94.2 mg, 0.55 mmol, 1.10 equiv) were dissolved in anhydrous MeCN (1.25 mL). The phenol (0.55 mmol, 1.10 equiv) was added and stirred at rt for 12 hours. After the reaction was completed, the mixture was diluted with EA (20 mL), washed with 2 M aqueous HCl solution (15 mL). The organic layer was separated, washed with saturated brine (20 mL), dried over anhydrous sodium sulfate, filtered, and concentrated in vacuo. The crude product was then purified by silica gel column chromatography.

*4-Bromophenyl isobutyrate (**3a**)*

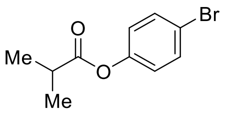

Compound **3a** was prepared according to the general procedure using **1a** (86.0 mg, 0.50 mmol, 1.00 equiv) and *p*-bromophenol (95.2 mg, 0.55 mmol, 1.10 equiv) at rt for 12 hours. Purification by flash column chromatography (petroleum ether/ethyl acetate = 20/1, *v/v*) afforded 4-bromophenyl isobutyrate (**3a**) as a colorless oil (111.8 mg, 92%).

$\mathbf{R_f}$ = 0.40 (petroleum ether/ethyl acetate = 40/1, *v/v*).

^{1}**H NMR** (400 MHz, $CDCl_3$) δ 7.48 (d, *J* = 7.5 Hz, 2H), 6.97 (d, *J* = 7.5 Hz, 2H), 2.79 (hept, *J* = 6.9 Hz, 1H), 1.31 (d, *J* = 6.9 Hz, 6H).

^{13}**C NMR** (101 MHz, $CDCl_3$) δ 175.3, 149.9, 132.4 (2C), 123.4 (2C), 118.7, 34.2, 18.9 (2C).

HRMS−ESI (m/z) for $C_{10}H_{11}BrO_2$ $[M + Na]^+$: calcd 264.9834 (^{79}Br), 266.9814 (^{81}Br), found 264.9835 (^{79}Br), 266.9814 (^{81}Br).

*4-Bromophenyl 2,3-dimethylbutanoate (**3b**)*

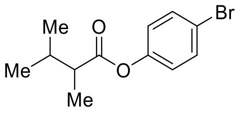

Compound **3b** was prepared according to the general procedure using **1b** (100.0 mg, 0.50 mmol, 1.00 equiv) and *p*-bromophenol (95.2 mg, 0.55 mmol, 1.10 equiv) at rt for 12 hours. Purification by flash column chromatography (petroleum ether/ethyl acetate = 20/1, *v/v*) afforded 4-bromophenyl 2,3-dimethylbutanoate (**3b**) as a colorless oil (115.2 mg, 85%).

$\mathbf{R_f}$ = 0.40 (petroleum ether/ethyl acetate = 40/1, *v/v*).

^{1}H NMR (400 MHz, $CDCl_3$) δ 7.5 (d, *J* = 8.8 Hz, 2H), 7.0 (d, *J* = 8.9 Hz, 2H), 2.5 (m, 1H), 2.1 (m, 1H), 1.3 (d, *J* = 7.1 Hz, 3H), 1.04 (d, *J* = 6.8 Hz, 1H), 1.00 (d, *J* = 6.7 Hz, 1H).

^{13}C NMR (101 MHz, $CDCl_3$) δ 174.5, 149.9, 132.4 (2C), 123.4 (2C), 118.7, 46.2, 31.2, 20.7, 19.2, 13.7.

HRMS−ESI (m/z) for $C_{12}H_{15}BrO_2$ $[M + Na]^+$: calcd 293.0147 (^{79}Br), 295.0127 (^{81}Br), found 293.0149 (^{79}Br), 295.0127 (^{81}Br).

*4-Bromophenyl 2,4-dimethylpentanoate (**3c**)*

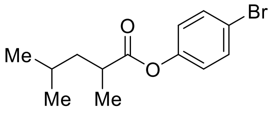

Compound **3c** was prepared according to the general procedure using **1c** (107.0 mg, 0.50 mmol, 1.00 equiv) and *p*-bromophenol (95.2 mg, 0.55 mmol, 1.10 equiv) at rt for 12 hours. Purification by flash column chromatography (petroleum ether/ethyl acetate = 20/1, *v/v*) afforded 4-bromophenyl 2,4-dimethylpentanoate (**3c**) as a colorless oil (126.4 mg, 90%).

R_f = 0.40 (petroleum ether/ethyl acetate = 40/1, *v/v*).

^{1}H NMR (400 MHz, $CDCl_3$) δ 7.48 (d, *J* = 8.8 Hz, 2H), 6.96 (d, *J* = 8.7 Hz, 2H), 2.84 – 2.64 (m, 1H), 1.83 – 1.64 (comp, 2H), 1.42 – 1.31 (m, 1H), 1.28 (d, *J* = 6.9 Hz, 3H), 0.97 (d, *J* = 6.2 Hz, 1H), 0.94 (d, *J* = 6.1 Hz, 1H).

^{13}C NMR (101 MHz, $CDCl_3$) δ 175.4, 150.0, 132.5 (2C), 123.5 (2C), 118.9, 43.0, 37.9, 26.2, 22.7, 22.6, 17.5.

HRMS−ESI (m/z) for $C_{13}H_{17}BrO_2$ $[M + Na]^+$: calcd 307.0304 (^{79}Br), 309.0283 (^{81}Br), found 307.0304 (^{79}Br), 309.0288 (^{81}Br).

*4-Bromophenyl 2-methylpent-4-ynoate (**3d**)*

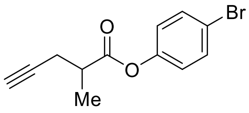

Compound **3d** was prepared according to the general procedure using **1d** (98.0 mg, 0.50 mmol, 1.00 equiv) and *p*-bromophenol (95.2 mg, 0.55 mmol, 1.10 equiv) at rt for 12 hours. Purification by flash column chromatography (petroleum ether/ethyl acetate = 20/1, *v/v*) afforded 4-bromophenyl 2-methylpent-4-ynoate (**3d**) as a colorless oil (121.9 mg, 90%).

R_f = 0.40 (petroleum ether/ethyl acetate = 40/1, *v/v*).

^{1}H NMR (400 MHz, $CDCl_3$) δ 7.49 (d, *J* = 8.9 Hz, 2H), 6.99 (d, *J* = 8.8 Hz, 2H), 2.91 (m, 1H), 2.59 (comp, 2H), 2.06 (s, 1H), 1.41 (d, *J* = 7.1 Hz, 3H).

^{13}C NMR (101 MHz, $CDCl_3$) δ 173.1, 149.9, 132.6 (2C), 123.5 (2C), 119.1, 81.0, 70.5, 39.0, 22.9, 16.4.

HRMS−ESI (m/z) for $C_{12}H_{11}BrO_2$ $[M + Na]^+$: calcd 288.9835 (^{79}Br), 290.9815 (^{81}Br), found 288.9831 (^{79}Br), 290.9815 (^{81}Br).

*4-Bromophenyl 3-cyclohexyl-2-methylpropanoate (**3e**)*

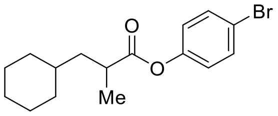

Compound **3e** was prepared according to the general procedure using **1e** (127.0 mg, 0.50 mmol, 1.00 equiv) and *p*-bromophenol (95.2 mg, 0.55 mmol, 1.10 equiv) at rt for 12 hours. Purification by flash column chromatography (petroleum ether/ethyl acetate = 20/1, *v/v*) afforded 4-bromophenyl 3-cyclohexyl-2-methylpropanoate (**3e**) as a colorless oil (137.7 mg, 85%).

$\mathbf{R}_f$ = 0.40 (petroleum ether/ethyl acetate = 40/1, *v/v*).

^{1}H NMR (400 MHz, $CDCl_3$) δ 7.48 (d, *J* = 8.8 Hz, 2H), 6.96 (d, *J* = 8.8 Hz, 2H), 2.84 – 2.71 (m, 1H), 1.84 – 1.62 (comp, 6H), 1.37 (comp, 2H), 1.27 (d, *J* = 6.9 Hz, 3H), 1.23 – 1.10 (comp, 3H), 0.93 (comp, 2H).

^{13}C NMR (101 MHz, $CDCl_3$) δ 175.5, 150.1, 132.6 (2C), 123.5 (2C), 118.8, 41.6, 37.2, 35.7, 33.5, 33.3, 26.7, 26.4, 26.4, 17.6.

HRMS−ESI (m/z) for $C_{16}H_{21}BrO_2$ $[M + Na]^+$: calcd 347.0617 (^{79}Br), 349.0596 (^{81}Br), found 347.0617 (^{79}Br), 349.0598 (^{81}Br).

*4-Bromophenyl 2-methyl-3-phenylpropanoate (**3f**)*

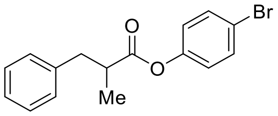

Compound **3f** was prepared according to the general procedure using **1f** (124.0 mg, 0.50 mmol, 1.00 equiv) and *p*-bromophenol (95.2 mg, 0.55 mmol, 1.10 equiv) at rt for 12 hours. Purification by flash column chromatography (petroleum ether/ethyl acetate = 20/1, *v/v*) afforded 4-bromophenyl 2-methyl-3-phenylpropanoate (**3f**) as a colorless oil (145.2 mg, 91%).

$\mathbf{R}_f$ = 0.40 (petroleum ether/ethyl acetate = 40/1, *v/v*).

^{1}H NMR (400 MHz, $CDCl_3$) δ 7.44 (d, *J* = 8.4 Hz, 2H), 7.31 (m, 2H), 7.23 (comp, 3H), 6.79 (d, *J* = 8.3 Hz, 2H), 3.09 (dd, *J* = 13.3, 7.7 Hz, 1H), 2.99 (m, 1H), 2.83 (dd, *J* = 13.3, 6.6 Hz, 1H), 1.32 (d, *J* = 6.6 Hz, 3H).

^{13}C NMR (101 MHz, $CDCl_3$) δ 174.4, 149.8, 139.0, 132.5 (2C), 129.2 (2C), 128.6 (2C), 126.7, 123.4 (2C), 118.9, 41.8, 40.0, 17.1.

HRMS−ESI (m/z) for $C_{16}H_{15}BrO_2$ $[M + Na]^+$: calcd 341.0148 (^{79}Br), 343.0128 (^{81}Br), found 341.0149 (^{79}Br), 343.0132 (^{81}Br).

*4-Bromophenyl 3-(4-fluorophenyl)-2-methylpropanoate (**3g**)*

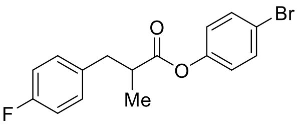

Compound **3g** was prepared according to the general procedure using **1g** (133.0 mg, 0.50 mmol, 1.00 equiv) and *p*-bromophenol (95.2 mg, 0.55 mmol, 1.10 equiv) at rt for 12 hours. Purification by flash column chromatography (petroleum ether/ethyl acetate = 20/1, *v/v*) afforded 4-bromophenyl 3-(4-fluorophenyl)-2-methylpropanoate (**3g**) as a colorless oil (151.7 mg, 95%).

$\mathbf{R}_f$ = 0.40 (petroleum ether/ethyl acetate = 40/1, *v/v*).

^{1}H NMR (400 MHz, $CDCl_3$) δ 7.46 (d, *J* = 8.8 Hz, 2H), 7.19 (dd, *J* = 8.3, 5.4 Hz, 2H), 7.00 (t, *J* = 8.5 Hz, 2H), 6.81 (d, *J* = 8.8 Hz, 2H), 3.07 (dd, *J* = 13.4, 7.7 Hz, 1H), 2.94 (m, 1H), 2.81 (dd, *J* = 13.4, 6.8 Hz, 1H), 1.31 (d, *J* = 6.9 Hz, 3H).

^{19}F NMR (377 MHz, $CDCl_3$) δ –116.4.

^{13}C NMR (101 MHz, $CDCl_3$) δ 174.2, 161.9 (d, J_{C-F} = 244.7 Hz), 149.8, 134.7 (d, J_{C-F} = 3.2 Hz), 132.6 (2C), 130.6 (d, J_{C-F} = 7.8 Hz, 2C), 123.4 (2C), 119.0, 115.5 (d, J_{C-F} = 21.2 Hz, 2C), 41.9, 39.1, 17.0.

HRMS−ESI (m/z) for $C_{16}H_{14}BrFO_2$ [M + Na]$^+$: calcd 359.0053 (^{79}Br), 361.0033 (^{81}Br), found 359.0052 (^{79}Br), 361.0035 (^{81}Br).

*4-Bromophenyl 3-(3-fluorophenyl)-2-methylpropanoate (**3h**)*

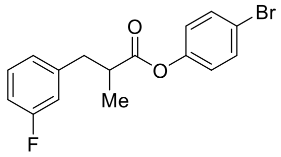

Compound **3h** was prepared according to the general procedure using **1h** (133.0 mg, 0.50 mmol, 1.00 equiv) and *p*-bromophenol (95.2 mg, 0.55 mmol, 1.10 equiv) at rt for 12 hours. Purification by flash column chromatography (petroleum ether/ethyl acetate = 20/1, *v/v*) afforded 4-bromophenyl 3-(3-fluorophenyl)-2-methylpropanoate (**3h**) as a colorless oil (146.7 mg, 87%).

$\mathbf{R}_f$ = 0.40 (petroleum ether/ethyl acetate = 40/1, *v/v*).

^{1}H NMR (400 MHz, $CDCl_3$) δ 7.46 (d, *J* = 8.8 Hz, 2H), 7.26 (m, 1H), 7.00 (m, 1H), 6.95 (comp, 2H), 6.83 (d, *J* = 8.4 Hz, 2H), 3.10 (dd, *J* = 13.4, 7.7 Hz, 1H), 2.98 (m, 1H), 2.82 (dd, *J* = 13.4, 6.9 Hz, 1H), 1.32 (d, *J* = 6.9 Hz, 3H).

^{19}F NMR (377 MHz, $CDCl_3$) δ –113.3.

^{13}C NMR (101 MHz, $CDCl_3$) δ 174.0, 162.9 (d, J_{C-F} = 246.0 Hz), 149.6, 141.4 (d, J_{C-F} = 7.2 Hz), 132.4 (2C), 130.0 (d, J_{C-F} = 8.3 Hz), 124.7 (d, J_{C-F} = 2.9 Hz), 123.2 (2C), 118.9, 115.9 (d, J_{C-F} = 21.1 Hz), 113.6 (d, J_{C-F} = 21.1 Hz), 41.4, 39.4, 17.0.

HRMS−ESI (m/z) for $C_{16}H_{14}BrFO_2$ [M + Na]$^+$: calcd 359.0053 (^{79}Br), 361.0033 (^{81}Br), found 359.0052 (^{79}Br), 361.0032 (^{81}Br).

*4-Bromophenyl 3-(2fluorophenyl)-2-methylpropanoate (**3i**)*

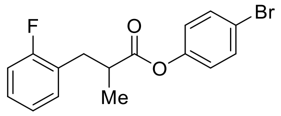

Compound **3i** was prepared according to the general procedure using **1i** (133.0 mg, 0.50 mmol, 1.00 equiv) and *p*-bromophenol (95.2 mg, 0.55 mmol, 1.10 equiv) at rt for 12 hours. Purification by flash column chromatography (petroleum ether/ethyl acetate = 20/1, *v/v*) afforded 4-bromophenyl 3-(2-fluorophenyl)-2-methylpropanoate (**3i**) as a colorless oil (143.3 mg, 93%).

$\mathbf{R}_f$ = 0.40 (petroleum ether/ethyl acetate = 40/1, *v/v*).

^{1}H NMR (400 MHz, $CDCl_3$) δ 7.45 (d, *J* = 8.8 Hz, 2H), 7.25 – 7.17 (comp, 2H), 7.11 – 7.00 (comp, 2H), 6.82 (d, *J* = 8.8 Hz, 2H), 3.14 – 2.99 (comp, 2H), 2.97 – 2.85 (m, 1H), 1.33 (d, *J* = 6.6 Hz, 3H)

^{19}F NMR (377 MHz, $CDCl_3$) δ –117.6.

^{13}C NMR (101 MHz, $CDCl_3$) δ 174.1, 161.4 (d, J_{C-F} = 245.3 Hz), 149.7, 132.4 (2C), 131.5 (d, J_{C-F} = 4.7 Hz), 128.5 (d, J_{C-F} = 8.2 Hz), 125.8 (d, J_{C-F} = 15.7 Hz), 124.1 (d, J_{C-F} = 3.6 Hz), 123.3 (2C), 118.8, 115.4 (d, J_{C-F} = 22.1 Hz), 40.2, 33.2 (d, J_{C-F} = 2.1 Hz), 17.0.

HRMS−ESI (m/z) for $C_{16}H_{14}BrFO_2$ $[M + Na]^+$: 359.0053 (^{79}Br), 361.0033 (^{81}Br), found 359.0054 (^{79}Br), 361.0033 (^{81}Br).

*4-Bromophenyl 3-cyclohexyl-2-methylpropanoate (**3j**)*

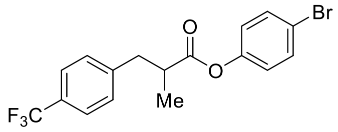

Compound **3j** was prepared according to the general procedure using **1j** (158.0 mg, 0.50 mmol, 1.00 equiv) and *p*-bromophenol (95.2 mg, 0.55 mmol, 1.10 equiv) at rt for 12 hours. Purification by flash column chromatography (petroleum ether/ethyl acetate = 20/1, *v/v*) afforded 4-bromophenyl 3-cyclohexyl-2-methylpropanoate (**3j**) as a colorless oil (168.0 mg, 93%).

$\mathbf{R}_f$ = 0.40 (petroleum ether/ethyl acetate = 40/1, *v/v*).

^{1}H NMR (400 MHz, $CDCl_3$) δ 7.57 (d, *J* = 7.9 Hz, 2H), 7.46 (d, *J* = 8.8 Hz, 2H), 7.35 (d, *J* = 7.9 Hz, 2H), 6.80 (d, *J* = 8.9 Hz, 2H), 3.16 (dd, *J* = 13.4, 7.7 Hz, 1H), 3.01 (m, 1H), 2.88 (dd, *J* = 13.4, 7.0 Hz, 1H), 1.34 (d, *J* = 6.8 Hz, 3H).

^{19}F NMR (377 MHz, $CDCl_3$) δ –62.8.

^{13}C NMR (101 MHz, $CDCl_3$) δ 173.9, 149.7, 143.1, 132.6 (2C), 129.5 (2C), 129.1 (q, J_{C-F} = 32.1 Hz), 125.6 (q, J_{C-F} = 3.8 Hz, 2C), 124.3 (q, J_{C-F} = 272.0 Hz), 123.3 (2C), 119.1, 41.5, 39.5, 17.1.

HRMS−ESI (m/z) for $C_{17}H_{14}BrF_3O_2$ $[M + Na]^+$: calcd 409.0021 (^{79}Br), 411.0001 (^{81}Br), found 409.0025 (^{79}Br), 411.0014 (^{81}Br).

*4-Nitrophenyl isobutyrate (**3k**)*

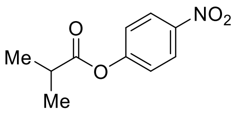

Compound **3k** was prepared according to the general procedure using **1a** (86.0 mg, 0.50 mmol, 1.00 equiv) and *p*-nitrophenol (76.5 mg, 0.55 mmol, 1.10 equiv) at rt for 12 hours. Purification by flash column chromatography (petroleum ether/ethyl acetate = 10/1, *v/v*) afforded 4-nitrophenyl isobutyrate (**3k**) as a white solid (88.8 mg, 85%).

The NMR data of **3k** were in agreement with the literature data.[6]

$\mathbf{R_f}$ = 0.50 (petroleum ether/ethyl acetate = 10/1, *v/v*).

HRMS−ESI (m/z) for $C_{10}H_{11}NO_4$ $[M + Na]^+$: calcd 232.0581, found 232.0580.

*4-(Trifluoromethyl)phenyl isobutyrate (**3l**)*

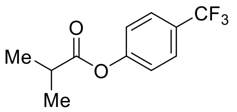

Compound **3l** was prepared according to the general procedure using **1a** (86.0 mg, 0.50 mmol, 1.00 equiv) and 4-(trifluoromethyl)phenol (89.2 mg, 0.55 mmol, 1.10 equiv) at rt for 12 hours. Purification by flash column chromatography (petroleum ether/ethyl acetate = 40/1, *v/v*) afforded 4-(trifluoromethyl)phenyl isobutyrate (**3l**) as a colorless oil (104.4 mg, 90%).

$\mathbf{R_f}$ = 0.40 (petroleum ether/ethyl acetate = 40/1, *v/v*).

^{1}H NMR (400 MHz, $CDCl_3$) δ 7.64 (d, J = 8.6 Hz, 2H), 7.20 (d, J = 8.6 Hz, 2H), 2.82 (hept, J = 7.0 Hz, 1H), 1.33 (d, J = 7.0 Hz, 6H).

^{19}F NMR (377 MHz, $CDCl_3$) δ –62.2.

^{13}C NMR (101 MHz, $CDCl_3$) δ175.1, 153.4, 128.1 (q, J_{C-F} = 31.2 Hz), 126.7 (q, J_{C-F} = 3.8 Hz, 2C), 126.2 (q, J_{C-F} = 271.3 Hz), 122.0 (2C), 34.2, 18.8 (2C).

HRMS−ESI (m/z) for $C_{11}H_{11}F_3O_2$ $[M + Na]^+$: calcd 255.0603, found 255.0607.

*4-(1H-Tetrazol-1-yl)phenyl isobutyrate (**3m**)*

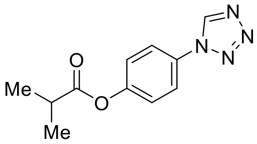

Compound **3m** was prepared according to the general procedure using **1a** (86.0 mg, 0.50 mmol, 1.00 equiv) and 4-(1H-tetrazol-1-yl)phenol (89.2 mg, 0.55 mmol, 1.10 equiv) at rt for 12 hours. Purification by flash column chromatography (petroleum ether/ethyl acetate = 10/1, *v/v*) afforded 4-(1H-tetrazol-1-yl)phenyl isobutyrate (**3m**) as a colorless oil (98.7 mg, 85%).

$\mathbf{R_f}$ = 0.35 (petroleum ether/ethyl acetate = 10/1, *v/v*).

^{1}H NMR (400 MHz, $CDCl_3$) δ 8.97 (s, 1H), 7.72 (d, J = 8.6 Hz, 2H), 7.33 (d, J = 8.6 Hz, 2H), 2.85 (hept, J = 7.0 Hz, 1H), 1.35 (d, J = 7.0 Hz, 6H).

^{13}C NMR (101 MHz, $CDCl_3$) δ 175.1, 151.9, 140.6, 131.1, 123.5 (2C), 122.5 (2C), 34.2, 18.8 (2C).

HRMS−ESI (m/z) for $C_{11}H_{12}N_4O_2$ $[M + Na]^+$: calcd 255.0852, found 255.0854.

*4-(Dimethylamino)phenyl isobutyrate (**3n**)*

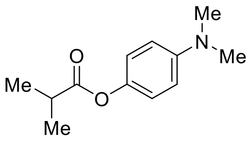

Compound **3n** was prepared according to the general procedure using **1a** (86.0 mg, 0.50 mmol, 1.00 equiv) and 4-(dimethylamino)phenol (75.4 mg, 0.55 mmol, 1.10 equiv) at rt for 12 hours. Purification by flash column chromatography (petroleum ether/ethyl acetate = 20/1, *v/v*) afforded 4-(dimethylamino)phenyl isobutyrate (**3n**) as a white solid (88.0 mg, 85%).

$\mathbf{R_f}$ = 0.40 (petroleum ether/ethyl acetate = 40/1, *v/v*). **mp** 59−60 ºC (petroleum ether).

^{1}H NMR (400 MHz, $CDCl_3$) δ 6.93 (d, J = 9.1 Hz, 2H), 6.70 (d, J = 9.0 Hz, 2H), 2.92 (s, 6H), 2.76 (hept, J = 7.0 Hz, 1H), 1.30 (d, J = 7.0 Hz, 6H).

^{13}C NMR (101 MHz, $CDCl_3$) δ 176.2, 148.7, 141.8, 121.7 (2C), 113.2 (2C), 41.0 (2C), 34.1, 19.0 (2C).

HRMS−ESI (m/z) for $C_{12}H_{17}NO_2$ $[M + H]^+$: calcd 208.1332, found 208.1334.

*4-Methoxyphenyl isobutyrate (**3o**)*

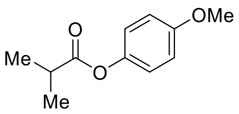

Compound **3o** was prepared according to the general procedure using **1a** (86.0 mg, 0.50 mmol, 1.00 equiv) and 4-methoxyphenol (68.3 mg, 0.55 mmol, 1.10 equiv) at rt for 12 hours. Purification by flash column chromatography (petroleum ether/ethyl acetate = 20/1, *v/v*) afforded 4-methoxyphenyl isobutyrate (**3o**) as a colorless oil (91.2 mg, 94%).

The NMR data of **3o** were in agreement with the literature data.[7]

$\mathbf{R_f}$ = 0.40 (petroleum ether/ethyl acetate = 20/1, *v/v*).

HRMS−ESI (m/z) for $C_{11}H_{14}O_3$ $[M + Na]^+$: calcd 217.0835, found 217.0837.

*Methyl 4-(isobutyryloxy)benzoate (**3p**)*

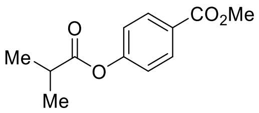

Compound **3p** was prepared according to the general procedure using **1a** (86.0 mg, 0.50 mmol, 1.00 equiv) and methyl 4-hydroxybenzoate (83.7 mg, 0.55 mmol, 1.10 equiv) at rt for 4 hours. Purification by flash column chromatography (petroleum ether/ethyl acetate = 20/1, *v/v*) afforded methyl 4-(isobutyryloxy)benzoate (**3p**) as a colorless oil (100.0 mg, 90%).

$\mathbf{R}_f$ = 0.40 (petroleum ether/ethyl acetate = 40/1, *v/v*).

^{1}H NMR (400 MHz, $CDCl_3$) δ 8.07 (d, *J* = 9.1 Hz, 2H), 7.15 (d, *J* = 9.1 Hz, 2H), 3.91 (s, 3H), 2.81 (hept, *J* = 7.0 Hz, 1H), 1.32 (d, *J* = 6.9 Hz, 6H).

^{13}C NMR (101 MHz, $CDCl_3$) δ 175.1, 166.4, 154.6, 131.1 (2C), 127.6, 121.6 (2C), 52.2, 34.2, 18.9 (2C).

HRMS−ESI (m/z) for $C_{12}H_{14}O_4$ $[M + Na]^+$: calcd 245.0785, found 245.0787.

*4-Formylphenyl isobutyrate (**3q**)*

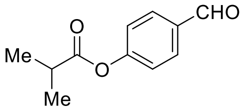

Compound **3q** was prepared according to the general procedure using **1a** (86.0 mg, 0.50 mmol, 1.00 equiv) and 4-hydroxybenzaldehyde (67.2 mg, 0.55 mmol, 1.10 equiv) at rt for 12 hours. Purification by flash column chromatography (petroleum ether/ethyl acetate = 20/1, *v/v*) afforded 4-formylphenyl isobutyrate (**3q**) as a colorless oil (82.6 mg, 86%).

$\mathbf{R}_f$ = 0.40 (petroleum ether/ethyl acetate = 40/1, *v/v*).

^{1}H NMR (400 MHz, $CDCl_3$) δ 9.99 (s, 1H), 7.92 (d, *J* = 8.5 Hz, 2H), 7.27 (d, *J* = 8.4 Hz, 2H), 2.83 (hept, *J* = 6.9 Hz, 1H), 1.35 (d, *J* = 6.9 Hz, 6H).

^{13}C NMR (101 MHz, $CDCl_3$) δ 190.9, 174.9, 155.7, 133.9, 131.2 (2C), 122.3 (2C), 34.3, 18.8 (2C).

HRMS−ESI (m/z) for $C_{11}H_{12}O_3$ $[M + Na]^+$: calcd 215.0679, found 215.0681.

*Naphthalen-1-yl isobutyrate (**3r**)*

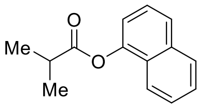

Compound **3r** was prepared according to the general procedure using **1a** (86.0 mg, 0.50 mmol, 1.00 equiv) and naphthalen-1-ol (79.3 mg, 0.55 mmol, 1.10 equiv) at rt for 12 hours. Purification by flash column chromatography (petroleum ether/ethyl acetate = 20/1, *v/v*) afforded naphthalen-1-yl isobutyrate (**3r**) as a colorless oil (98.6mg, 92%).

R_f = 0.40 (petroleum ether/ethyl acetate = 40/1, *v/v*).

^{1}H NMR (400 MHz, $CDCl_3$) δ 8.00 – 7.86 (comp, 2H), 7.77 (d, *J* = 8.3 Hz, 1H), 7.60 – 7.46 (comp, 3H), 7.28 (d, *J* = 7.4 Hz, 1H), 3.04 (hept, *J* = 7.0 Hz, 1H), 1.49 (d, *J* = 7.0 Hz, 6H).

^{13}C NMR (101 MHz, $CDCl_3$) δ 175.6, 146.7, 134.7, 128.1, 127.0, 126.4, 126.4, 125.9, 125.4, 121.1, 118.0, 34.5, 19.2 (2C).

HRMS−ESI (m/z) for $C_{14}H_{14}O_2$ $[M + Na]^+$: calcd 237.0886, found 237.0887.

*[1,1'-Biphenyl]-2-yl isobutyrate (**3s**)*

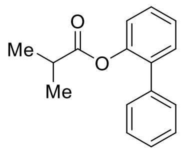

Compound **3s** was prepared according to the general procedure using **1a** (86.0 mg, 0.50 mmol, 1.00 equiv) and [1,1'-biphenyl]-2-ol (93.6 mg, 0.55 mmol, 1.10 equiv) at rt for 12 hours Purification by flash column chromatography (petroleum ether/ethyl acetate = 20/1, *v/v*) afforded [1,1'-biphenyl]-2-yl isobutyrate (**3s**) as a colorless oil (111.7 mg, 93%).

R_f = 0.40 (petroleum ether/ethyl acetate = 40/1, *v/v*).

^{1}H NMR (400 MHz, $CDCl_3$) δ 7.45 – 7.29 (comp, 8H), 7.13 (d, *J* = 7.9 Hz, 1H), 2.62 (hept, *J* = 6.9 Hz, 1H), 1.10 (d, *J* = 6.8 Hz, 6H).

^{13}C NMR (101 MHz, $CDCl_3$) δ 175.4, 147.9, 137.6, 135.2, 130.9, 129.1 (2C), 128.5, 128.1 (2C), 127.4, 126.1, 122.8, 34.1, 18.7 (2C).

HRMS−ESI (m/z) for $C_{16}H_{16}O_2$ $[M + Na]^+$: calcd 263.1042, found 263.1044.

*2,4-Dibromophenyl isobutyrate (**3t**)*

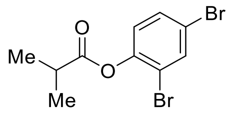

Compound **3t** was prepared according to the general procedure using **1a** (86.0 mg, 0.50 mmol, 1.00 equiv) and 2,4-dibromophenol (138.5 mg, 0.55 mmol, 1.10 equiv) at rt for 12 hours. Purification by flash column chromatography (petroleum ether/ethyl acetate = 20/1, *v/v*) afforded methyl 2,4-dibromophenyl isobutyrate (**3t**) as a colorless oil (137.2 mg, 86%).

R_f = 0.40 (petroleum ether/ethyl acetate = 40/1, *v/v*).

^{1}H NMR (400 MHz, $CDCl_3$) δ 7.46 (d, *J* = 8.7 Hz, 1H), 7.33 – 7.22 (comp, 2H), 2.86 (hept, *J* = 7.0 Hz, 1H), 1.36 (d, *J* = 7.0 Hz, 6H)..

^{13}C NMR (101 MHz, $CDCl_3$) δ 174.2, 148.9, 134.2, 130.3, 127.1, 121.1, 115.3, 34.2, 18.9 (2C).

HRMS−ESI (m/z) for $C_{10}H_{10}{}^{79}Br_2O_2$ $[M + Na]^+$: calcd 342.8940, found 342.8944; $C_{10}H_{10}{}^{79}Br^{81}Br\ O_2$ $[M + Na]^+$: calcd 344.8920, found 344.8919.

*Methyl 3-(isobutyryloxy)-2-naphthoate (**3u**)*

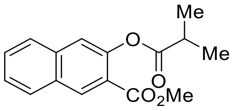

Compound **3u** was prepared according to the general procedure using **1a** (86.0 mg, 0.50 mmol, 1.00 equiv) and methyl 3-hydroxy-2-naphthoate (111.2 mg, 0.55 mmol, 1.10 equiv) at rt for 12 hours. Purification by flash column chromatography (petroleum ether/ethyl acetate = 20/1, *v/v*) afforded methyl 3-(isobutyryloxy)-2-naphthoate (**3u**) as a white solid (114.7 mg, 84%).

$\mathbf{R_f}$ = 0.40 (petroleum ether/ethyl acetate = 20/1, *v/v*). **mp** 58−59 °C (petroleum ether).

^{1}H NMR (400 MHz, $CDCl_3$) δ 8.58 (s, 1H), 7.93 (d, *J* = 8.2 Hz, 1H), 7.80 (d, *J* = 8.2 Hz, 1H), 7.63 – 7.48 (comp, 3H), 3.92 (s, 3H), 2.93 (hept, *J* = 7.0 Hz, 1H), 1.39 (d, *J* = 7.1 Hz, 6H).

^{13}C NMR (101 MHz, $CDCl_3$) δ176.1, 165.3, 147.0, 135.7, 133.7, 130.7, 129.1, 128.9, 127.3, 126.6, 122.5, 121.1, 52.3, 34.3, 19.0 (2C).

HRMS−ESI (m/z) for $C_{16}H_{16}O_4$ $[M + Na]^+$: calcd 295.0941, found 295.0943.

*2,6-Dimethylphenyl isobutyrate (**3v**)*

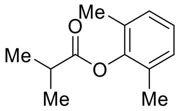

Compound **3v** was prepared according to the general procedure using **1a** (86.0 mg, 0.50 mmol, 1.00 equiv) and 2,6-dimethylphenol (68.2 mg, 0.55 mmol, 1.10 equiv) at 60 °C for 12 hours. Purification by flash column chromatography (petroleum ether/ethyl acetate = 20/1, *v/v*) afforded 2,6-dimethylphenyl isobutyrate (**3v**) as a colorless oil (89.4 mg, 93%).

$\mathbf{R_f}$ = 0.40 (petroleum ether/ethyl acetate = 40/1, *v/v*).

^{1}H NMR (400 MHz, $CDCl_3$) δ 7.15 – 6.93 (comp, 3H), 2.87 (hept, *J* = 7.0 Hz, 1H), 2.13 (s, 6H), 1.36 (d, *J* = 7.0 Hz, 6H).

^{13}C NMR (101 MHz, $CDCl_3$) δ 174.7, 148.1, 130.1, 128.6 (2C), 125.7 (2C), 34.3, 19.2 (2C), 16.3 (2C).

HRMS−ESI (m/z) for $C_{12}H_{16}O_2$ $[M + Na]^+$: calcd 215.1042, found 215.1041.

*2-Oxo-2H-chromen-7-yl isobutyrate (**3w**)*

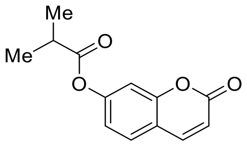

Compound **3w** was prepared according to the general procedure using **1a** (86.0 mg, 0.50 mmol, 1.00 equiv) and 7-hydroxy-2H-chromen-2-one (89.2 mg, 0.55 mmol, 1.10 equiv) at rt for 12 hours. Purification by flash column chromatography (petroleum ether/ethyl acetate = 20/1, *v/v*) afforded 2-oxo-2H-chromen-7-yl isobutyrate (**3w**) as a white solid (98.7 mg, 85%).

R*f* = 0.45 (petroleum ether/ethyl acetate = 20/1, *v/v*). **mp** 104−105 °C (petroleum ether).

^{1}H NMR (400 MHz, $CDCl_3$) δ 7.69 (d, *J* = 9.5 Hz, 1H), 7.49 (d, *J* = 8.4 Hz, 1H), 7.10 (s, 1H), 7.04 (d, *J* = 8.4 Hz, 1H), 6.39 (d, *J* = 9.5 Hz, 1H), 2.84 (hept, *J* = 7.0 Hz, 1H), 1.34 (d, *J* = 6.9 Hz, 6H).

^{13}C NMR (101 MHz, $CDCl_3$) δ 174.9, 160.4, 154.7, 153.5, 142.9, 128.5, 118.4, 116.6, 116.0, 110.4, 34.2, 18.8 (2C).

HRMS−ESI (m/z) for $C_{13}H_{12}O_4$ $[M + Na]^+$: calcd 255.0628, found 255.0628.

*5-Bromopyridin-3-yl isobutyrate (**3x**)*

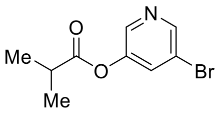

Compound **3x** was prepared according to the general procedure using **1a** (86.0 mg, 0.50 mmol, 1.00 equiv) and 5-bromopyridin-3-ol (95.7 mg, 0.55 mmol, 1.10 equiv) at rt for 12 hours. Purification by flash column chromatography (petroleum ether/ethyl acetate = 20/1, *v/v*) afforded 5-bromopyridin-3-yl isobutyrate (**3x**) as a colorless oil (100.1 mg, 82%).

R*f* = 0.45 (petroleum ether/ethyl acetate = 20/1, *v/v*).

^{1}H NMR (400 MHz, $CDCl_3$) δ 8.57 (s, 1H), 8.38 (s, 1H), 7.71 (s, 1H), 2.86 (hept, *J* = 7.0 Hz, 1H), 1.35 (d, *J* = 7.0 Hz, 6H).

^{13}C NMR (101 MHz, $CDCl_3$) δ 174.6, 147.9, 147.5, 141.6, 132.1, 119.9, 34.1, 18.8 (2C).

HRMS−ESI (m/z) for $C_9H_{10}BrNO_2$ $[M + H]^+$: calcd 243.9968 (^{79}Br), 245.9947 (^{81}Br), found 243.9970 (^{79}Br), 245.9948 (^{81}Br).

*2-Chloropyrimidin-5-yl isobutyrate (**3y**)*

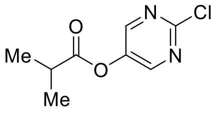

Compound **3y** was prepared according to the general procedure using **1a** (86.0 mg, 0.50 mmol, 1.00 equiv) and 2-chloropyrimidin-5-ol (71.8 mg, 0.55 mmol, 1.10 equiv) at rt for 12 hours. Purification by flash

column chromatography (petroleum ether/ethyl acetate = 20/1, *v/v*) afforded 2-chloropyrimidin-5-yl isobutyrate (**3y**) as a colorless oil (85.3 mg, 85%).

R*f* = 0.45 (petroleum ether/ethyl acetate = 20/1, *v/v*).

^{1}H NMR (400 MHz, $CDCl_3$) δ 8.50 (s, 2H), 2.87 (hept, *J* = 7.0 Hz, 1H), 1.34 (d, *J* = 7.0 Hz, 6H).

^{13}C NMR (101 MHz, $CDCl_3$) δ 174.1, 157.1, 152.6 (2C), 145.1, 34.1, 18.7 (2C).

HRMS−ESI (m/z) for $C_8H_9ClN_2O_2$ $[M + H]^+$: calcd 201.0426, found 201.0426.

4.2 A general procedure for alcohols, thiol, thiophenol, and amines.

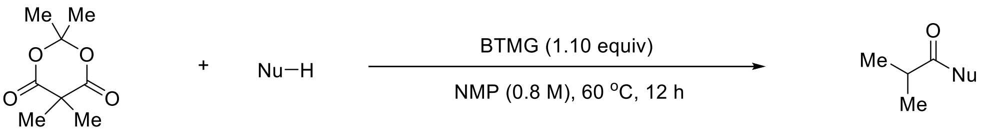

In a 4.0-mL vial, 2,2,5,5-tetramethyl-1,3-dioxane-4,6-dione (**1a**) (86.0 mg, 0.50 mmol, 1.00 equiv), BTMG (94.2 mg, 0.55 mmol, 1.10 equiv) were dissolved in anhydrous NMP (625.0 μL). Then, the nucleophile (0.55 mmol, 1.10 equiv) was added, and the mixture was stirred at 60 °C for 12 hours. After the reaction was completed, the mixture was diluted with EA (20 mL), washed with 2 M aqueous HCl solution (15 mL). The organic layer was separated, and washed with saturated brine (20 mL), dried over anhydrous sodium sulfate, filtered, and concentrated in vacuo. The crude product was then purified by silica gel column chromatography.

*4-Bromobenzyl isobutyrate (**4a**)*

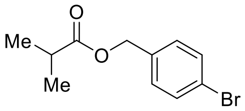

Compound **4a** was prepared according to the general procedure using **1a** (86.0 mg, 0.50 mmol, 1.00 equiv) and (4-bromophenyl)methanol (102.9 mg, 0.55 mmol, 1.10 equiv) at 60 °C for 12 hours. Purification by flash column chromatography (petroleum ether/ethyl acetate = 20/1, *v/v*) afforded 4-bromobenzyl isobutyrate (**4a**) as a colorless oil (117.0 mg, 91%).

R*f* = 0.55 (petroleum ether/ethyl acetate = 20/1, *v/v*).

^{1}H NMR (400 MHz, $CDCl_3$) δ 7.49 (d, *J* = 8.3 Hz, 2H), 7.22 (d, *J* = 8.3 Hz, 2H), 5.06 (s, 2H), 2.59 (hept, *J* = 7.0 Hz, 1H), 1.18 (d, *J* = 7.1 Hz, 6H).

^{13}C NMR (101 MHz, $CDCl_3$) δ 176.8, 135.3, 131.7 (2C), 129.7 (2C), 122.1, 65.2, 34.0, 19.0 (2C).

HRMS−ESI (m/z) for $C_{11}H_{13}BrO_2$ $[M + Na]^+$: calcd 278.9992 (^{79}Br), 280.9971 (^{81}Br), found 278.9991 (^{79}Br), 280.9977 (^{81}Br).

*3-Phenylpropyl isobutyrate (**4b**)*

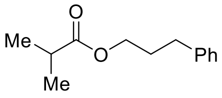

Compound **4b** was prepared according to the general procedure using **1a** (86.0 mg, 0.50 mmol, 1.00 equiv) and 3-phenylpropanol (74.9 mg, 0.55 mmol, 1.10 equiv) at 60 °C for 12 hours. Purification by flash column chromatography (petroleum ether/ethyl acetate = 20/1, *v/v*) afforded 3-phenylpropyl isobutyrate (**4b**) as a colorless oil (96.0 mg, 93%).

$\mathbf{R}_f$ = 0.55 (petroleum ether/ethyl acetate = 20/1, *v/v*).

^{1}**H NMR** (400 MHz, $CDCl_3$) δ 7.33 – 7.24 (m, 2H), 7.23 – 7.15 (comp, 3H), 4.09 (t, *J* = 6.5 Hz, 2H), 2.69 (t, *J* = 7.7 Hz, 2H), 2.55 (hept, *J* = 7.0 Hz, 1H), 1.96 (tt, *J* = 14.0, 6.5 Hz, 2H), 1.18 (d, *J* = 7.0 Hz, 6H).

^{13}**C NMR** (101 MHz, $CDCl_3$) δ 177.2, 141.3, 128.45 (2C), 128.41 (2C), 126.0, 63.5, 34.1, 32.2, 30.3, 19.0 (2C).

HRMS−ESI (m/z) for $C_{13}H_{18}O_2$ $[M + Na]^+$: calcd 229.1199, found 229.1201.

*Benzhydryl isobutyrate (**4c**)*

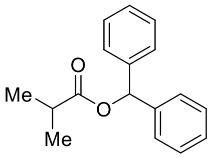

Compound **4c** was prepared according to the general procedure using **1a** (86.0 mg, 0.50 mmol, 1.00 equiv) and diphenylmethanol (101.3 mg, 0.55 mmol, 1.10 equiv) at 60 °C for 12 hours. Purification by flash column chromatography (petroleum ether/ethyl acetate = 20/1, *v/v*) afforded benzhydryl isobutyrate (**4c**) as a colorless oil (114.4 mg, 90%).

$\mathbf{R}_f$ = 0.55 (petroleum ether/ethyl acetate = 20/1, *v/v*).

^{1}**H NMR** (400 MHz, $CDCl_3$) δ 7.39 – 7.27 (comp, 10H), 6.86 (s, 1H), 2.67 (hept, *J* = 7.0 Hz, 1H), 1.21 (d, *J* = 6.9 Hz, 6H).

^{13}**C NMR** (101 MHz, $CDCl_3$) δ 176.0, 140.5 (2C), 128.5 (4C), 127.8 (2C), 127.0 (4C), 76.6, 34.2, 18.9 (2C).

HRMS−ESI (m/z) for $C_{17}H_{18}O_2$ $[M + Na]^+$: calcd 277.1199, found 277.1198.

*Cyclohexyl isobutyrate (**4d**)*

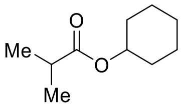

Compound **4d** was prepared according to the general procedure using **1a** (86.0 mg, 0.50 mmol, 1.00 equiv) and cyclohexanol (55.1 mg, 0.55 mmol, 1.10 equiv) at 80 °C for 12 hours. Purification by flash column

chromatography (petroleum ether/ethyl acetate = 20/1, *v/v*) afforded cyclohexyl isobutyrate (**4d**) as a colorless oil (69.8 mg, 82%).

R*f* = 0.55 (petroleum ether/ethyl acetate = 20/1, *v/v*).

The NMR data of **4d** were in agreement with the literature data.[8]

HRMS−ESI (m/z) for $C_{10}H_{18}O_2$ $[M + Na]^+$: calcd 193.1199, found 193.1205.

*S-(4-Fluorophenyl) 2-methylpropanethioate (**4e**)*

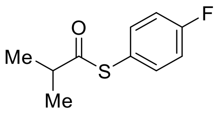

Compound **4e** was prepared according to the general procedure using **1a** (86.0 mg, 0.50 mmol, 1.00 equiv) and 4-fluorothiophenol (74.5 mg, 0.55 mmol, 1.10 equiv) at rt for 12 hours. Purification by flash column chromatography (petroleum ether/ethyl acetate = 10/1, *v/v*) afforded S-(4-fluorophenyl) 2-methylpropanethioate (**4e***)* as a colorless oil (81.2 mg, 82%).

R*f* = 0.65 (petroleum ether/ethyl acetate = 10/1, *v/v*).

^{1}H NMR (400 MHz, $CDCl_3$) δ 7.41 – 7.33 (m, 2H), 7.14 – 7.05 (m, 2H), 2.86 (hept, *J* = 6.9 Hz, 1H), 1.26 (d, *J* = 6.8 Hz, 6H).

^{19}F NMR (377 MHz, $CDCl_3$) δ –111.6.

^{13}C NMR (101 MHz, $CDCl_3$) δ 202.0, 163.5 (d, *J* = 249.6 Hz), 136.8 (d, *J* = 8.6 Hz, 2C), 123.3, 116.5 (d, *J* = 22.2 Hz, 2C), 43.1, 19.5 (2C).

HRMS−ESI (m/z) for $C_{10}H_{11}FOS$ $[M + H]^+$: calcd 199.0587, found 199.0584.

*S-Dodecyl 2-methylpropanethioate (**4f**)*

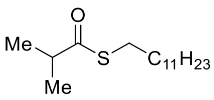

Compound **4f** was prepared according to the general procedure using **1a** (86.0 mg, 0.50 mmol, 1.00 equiv) and dodecane-1-thiol (111.3 mg, 0.55 mmol, 1.10 equiv) at rt for 12 hours. Purification by flash column chromatography (petroleum ether/ethyl acetate = 3/1, *v/v*) afforded S-dodecyl 2-methylpropanethioate (**4f**) as a colorless oil (51.1 mg, 90%).

R*f* = 0.60 (petroleum ether/ethyl acetate = 10/1, *v/v*).

^{1}H NMR (400 MHz, $CDCl_3$) δ 2.84 (t, *J* = 7.4 Hz, 2H), 2.72 (hept, *J* = 6.9 Hz, 1H), 1.65 – 1.49 (m, 2H), 1.42 – 1.21 (comp, 18H), 1.18 (d, *J* = 7.0 Hz, 6H), 0.88 (t, *J* = 6.7 Hz, 3H).

^{13}C NMR (101 MHz, $CDCl_3$) δ 204.5, 43.3, 32.1, 29.8 (3C), 29.7, 29.6, 29.5, 29.3, 29.0, 28.7, 22.8, 19.6 (2C), 14.2.

HRMS−ESI (m/z) for $C_{16}H_{32}OS$ $[M + H]^+$: calcd 273.2247, found 273.2247.

*N-(4-Bromobenzyl)isobutyramide (**4g**)*

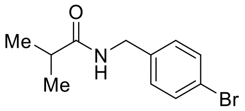

Compound **4g** was prepared according to the general procedure using **1a** (86.0 mg, 0.50 mmol, 1.00 equiv) and (4-bromophenyl)methanamine (102.3 mg, 0.55 mmol, 1.10 equiv) at 100 °C for 12 hours. Purification by flash column chromatography (petroleum ether/ethyl acetate = 10/1, *v/v*) afforded *N*-(4-bromobenzyl)isobutyramide (**4g**) as a yellow solid (117.4 mg, 92%).

$\mathbf{R_f}$ = 0.30 (petroleum ether/ethyl acetate = 10/1, *v/v*). **mp** 114−115 °C (petroleum ether).

^{1}H NMR (400 MHz, $CDCl_3$) δ 7.44 (d, *J* = 8.4 Hz, 2H), 7.13 (d, *J* = 8.4 Hz, 2H), 5.83 (s, 1H), 4.37 (d, *J* = 5.9 Hz, 2H), 2.38 (hept, *J* = 6.9 Hz, 1H), 1.17 (d, *J* = 6.9 Hz, 6H).

^{13}C NMR (101 MHz, $CDCl_3$) δ 177.0, 137.8, 131.9 (2C), 129.5 (2C), 121.4, 42.9, 35.8, 19.7 (2C).

HRMS−ESI (m/z) for $C_{11}H_{14}BrNO$ $[M + H]^+$: calcd 256.0332 (^{79}Br), 258.0312 (^{81}Br), found 256.0032 (^{79}Br), 258.0312 (^{81}Br).

*N-(3-Phenylpropyl)isobutyramide (**4h**)*

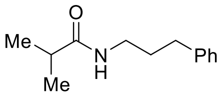

Compound **4h** was prepared according to the general procedure using **1a** (86.0 mg, 0.50 mmol, 1.00 equiv) and 3-phenylpropan-1-amine (74.4 mg, 0.55 mmol, 1.10 equiv) at 100 °C for 12 hours. Purification by flash column chromatography (petroleum ether/ethyl acetate = 20/1, *v/v*) afforded *N*-(3-phenylpropyl)isobutyramide (**4h**) as a yellow oil (93.4 mg, 91%).

$\mathbf{R_f}$ = 0.20 (petroleum ether/ethyl acetate = 20/1, *v/v*).

^{1}H NMR (400 MHz, $CDCl_3$) δ 7.39 – 7.24 (m, 2H), 7.23 – 7.12 (comp, 3H), 5.43 (br, 1H), 3.29 (q, *J* = 6.5 Hz, 2H), 2.65 (t, *J* = 7.6 Hz, 2H), 2.28 (hept, *J* = 6.9 Hz, 1H), 1.91 – 1.77 (m, 2H), 1.12 (d, *J* = 6.9 Hz, 6H).

^{13}C NMR (101 MHz, $CDCl_3$) δ 177.0, 141.7, 128.6 (2C), 128.5 (2C), 126.2, 39.2, 35.8, 33.5, 31.4, 19.7 (2C).

HRMS−ESI (m/z) for $C_{13}H_{19}NO$ $[M + H]^+$: calcd 206.1540, found 206.1540.

*N-(Heptadecan-9-yl)isobutyramide (**4i**)*

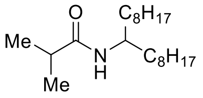

Compound **4i** was prepared according to the general procedure using **1a** (86.0 mg, 0.50 mmol, 1.00 equiv) and heptadecan-9-amine (140.5 mg, 0.55 mmol, 1.10 equiv) at 100 °C for 12 hours. Purification by flash

column chromatography (petroleum ether/ethyl acetate = 20/1, *v/v*) afforded *N-(heptadecan-9-yl)isobutyramide* (**4i**) as a white solid (146.5 mg, 90%).

R*f* = 0.50 (petroleum ether/ethyl acetate = 10/1, *v/v*). **mp** 85−86 °C (petroleum ether).

^{1}H NMR (400 MHz, $CDCl_3$) δ5.05 (d, *J* = 9.2 Hz, 1H), 3.88 (m, 1H), 2.31 (hept, *J* = 6.9 Hz, 1H), 1.47 (m, 2H), 1.27 (comp, 26H), 1.15 (d, *J* = 6.9 Hz, 6H), 0.87 (t, *J* = 6.7 Hz, 6H).

^{13}C NMR (101 MHz, $CDCl_3$) δ 176.5, 49.0, 36.1, 35.5 (2C), 32.0 (2C), 29.75 (2C), 29.67 (2C), 29.4 (2C), 26.0 (2C), 22.8 (2C), 19.9 (2C), 14.2 (2C).

HRMS−ESI (m/z) for $C_{21}H_{43}NO$ $[M + H]^+$: calcd 326.3418, found 326.3418.

*N-Cyclohexylisobutyramide (**4j**)*

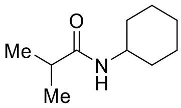

Compound **4j** was prepared according to the general procedure using **1a** (86.0 mg, 0.50 mmol, 1.00 equiv) and cyclohexanamine (54.5 mg, 0.55 mmol, 1.10 equiv) at 100 °C for 12 hours. Purification by flash column chromatography (petroleum ether/ethyl acetate = 10/1, *v/v*) afforded N-cyclohexylisobutyramide (**4j**) as a white solid (71.9 mg, 85%).

R*f* = 0.50 (petroleum ether/ethyl acetate = 10/1, *v/v*). **mp** 116−117 °C (petroleum ether).

^{1}H NMR (400 MHz, $CDCl_3$) δ 5.36 (s, 1H), 3.79 – 3.64 (m, 1H), 2.28 (hept, *J* = 6.8 Hz, 1H), 1.88 (m, 2H), 1.75 – 1.64 (m, 2H), 1.64 – 1.50 (m, 1H), 1.36 (m, 2H), 1.12 (comp, 9H).

^{13}C NMR (101 MHz, $CDCl_3$) δ 176.1, 47.9, 35.9, 33.3 (2C), 25.7, 25.0 (2C), 19.8 (2C).

HRMS−ESI (m/z) for $C_{10}H_{19}NO$ $[M + H]^+$: calcd 170.1540, found 170.1540.

*2-Methyl-1-morpholinopropan-1-one (**4k**)*

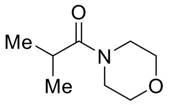

Compound **4k** was prepared according to the general procedure using **1a** (86.0 mg, 0.50 mmol, 1.00 equiv) and morpholine (47.9 mg, 0.55 mmol, 1.10 equiv) at 100 °C for 12 hours. Purification by flash column chromatography (petroleum ether/ethyl acetate = 3/1, *v/v*) afforded 2-methyl-1-morpholinopropan-1-one (**4k**) as a colorless oil (51.1 mg, 62%).

R*f* = 0.45 (petroleum ether/ethyl acetate = 3/1, *v/v*).

^{1}H NMR (400 MHz, $CDCl_3$) δ 3.74 – 3.63 (comp, 4H), 3.63 – 3.56 (m, 2H), 3.50 (m, 2H), 2.74 (hept, *J* = 6.8 Hz, 1H), 1.11 (d, *J* = 6.8 Hz, 6H).

^{13}C NMR (101 MHz, $CDCl_3$) δ 175.7, 67.1, 66.9, 46.1, 42.2, 30.0, 19.4 (2C).

HRMS−ESI (m/z) for $C_8H_{15}NO_2$ $[M + H]^+$: calcd 158.1176, found 158.1176.

5. *Preparation-scale Derivatization of Acetaminophen*

5.1 Gram-scale Preparation of *4-Acetamidophenyl isobutyrate* (**3z**)

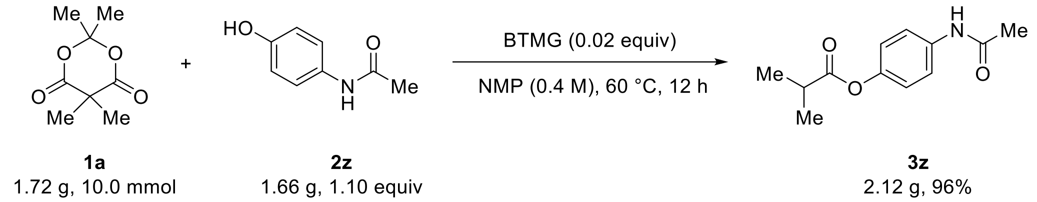

To a 50-mL round-bottom flask, 5,5-dimethyl-Meldrum's acid **1a** (1.72 g, 10.0 mmol, 1.00 equiv), BTMG (34.2 mg, 0.20 mmol, 0.02 equiv) were dissolved in anhydrous NMP (25.0 mL). Then, acetaminophen (1.66 g, 11.0 mmol, 1.10 equiv) was added, and stirred at 60 °C for 12 hours. After the reaction was completed, the mixture was diluted with EA (100 mL), and washed with 2 × 2 M aqueous HCl solution (50 mL). The organic layer was separated, washed with saturated brine (100 mL), dried over anhydrous sodium sulfate, filtered, and concentrated in vacuo. The crude product was then purified by silica gel column chromatography (petroleum ether/ethyl acetate = 3/1, *v/v*), which afforded 4-acetamidophenyl isobutyrate (**3z**) as a white solid (2.12 g, 96%).

*4-Acetamidophenyl isobutyrate (**3z**)*

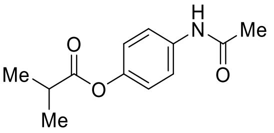

R*f* = 0.35 (petroleum ether/ethyl acetate = 3/1, *v/v*). **mp** 111−112 °C (petroleum ether).

^{1}H NMR (400 MHz, $CDCl_3$) δ 7.51 (d, *J* = 8.4 Hz, 2H), 7.23 (s, 1H), 7.04 (d, *J* = 8.4 Hz, 2H), 2.81 (hept, *J* = 7.0 Hz, 1H), 2.19 (s, 3H), 1.33 (d, *J* = 7.0 Hz, 6H).

^{13}C NMR (101 MHz, $CDCl_3$) δ 176.1, 168.5, 147.0, 135.6, 121.8 (2C), 120.9 (2C), 34.1, 24.4, 18.9 (2C).

HRMS−ESI (m/z) for $C_{12}H_{15}NO_3$ $[M + H]^+$: calcd 222.1125, found 222.1126.

5.2 Synthesis of *4-Acetamidophenyl 2-(((benzyloxy)carbonyl)amino)-2-methylpropanoate* (**6**)

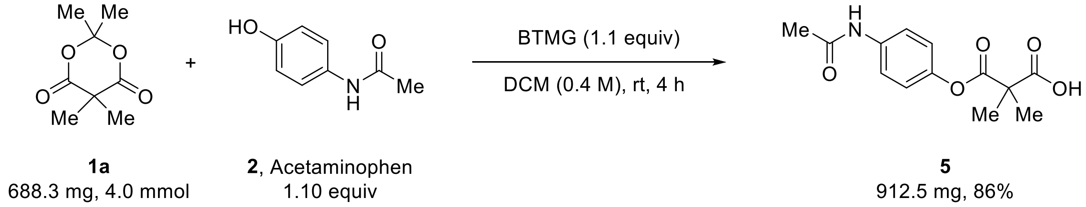

Step 1: To a 25-mL round-bottom flask, 2,2,5,5-tetramethyl-1,3-dioxane-4,6-dione (**1a**) (688.3 mg, 4.0 mmol, 1.00 equiv) and BTMG (753.6 mg, 0.55 mmol, 1.10 equiv) were dissolved in anhydrous DCM (10.0 mL). Then, 4-acetamidophenol (665.1 mg, 4.4 mmol, 1.10 equiv) was added, and the mixture was stirred at rt for 4 hours. After the reaction was completed, the mixture was diluted with DCM (20 mL), washed with 2 M aqueous HCl solution (15 mL). The organic layer was separated, washed with saturated

brine (20 mL), dried over anhydrous sodium sulfate, filtered, and concentrated in vacuo. The crude product was then purified by silica gel column chromatography (ethyl acetate), which afforded 3-(4-acetamidophenoxy)-2,2-dimethyl-3-oxopropanoic acid (**5**) as a white solid (912.5 mg, 86%).

R*f* = 0.20 (dichloromethane /methanol = 40/1, *v/v*). **mp** 166−168 °C (petroleum ether).

^{1}H NMR (400 MHz, DMSO-*d*$_6$) δ 13.04 (br, s, 1H), 9.98 (s, 1H), 7.61 (d, *J* = 9.0 Hz, 2H), 7.00 (d, *J* = 8.9 Hz, 2H), 2.04 (s, 3H), 1.46 (s, 6H).

^{13}C NMR (101 MHz, DMSO-*d*$_6$) δ 173.4, 171.6, 168.2, 145.5, 137.1, 121.4 (2C), 119.9 (2C), 49.4, 23.9, 22.4 (2C).

HRMS−ESI (m/z) for $C_{13}H_{16}NO_5$ $[M + H]^+$: calcd 266.1023, found 266.1030.

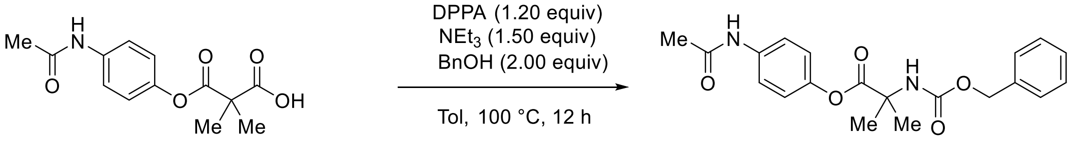

5, 132.5 mg, 0.50 mmol **6**, 140.6 mg, 76%

Step 2: To a 4.0-mL vial, the malonate half-ester **5** (132.5 mg, 0.50 mmol, 1.00 equiv), DPPA (165.1 mg, 0.60 mmol, 1.20 equiv), and TEA (75.9 mg, 0.75 mmol, 1.50 equiv) were dissolved in anhydrous toluene (2.5 mL). The mixture was stirred at 100 °C for 12 hours. After it was cooled to rt, benzyl alcohol (108.1 mg, 1.00 mmol, 2.00 equiv) was added and stirred at 100 °C for 12 hours. After the reaction was completed, the mixture was diluted with EA (20 mL), and washed with 2 M aqueous HCl solution (15 mL). The organic layer was separated, washed with saturated brine (20 mL), dried over anhydrous sodium sulfate, filtered, and concentrated in vacuo. The crude product was then purified by silica gel column chromatography (petroleum ether/ethyl acetate = 1/1, *v/v*), which afforded 4-acetamidophenyl 2-(((benzyloxy)carbonyl)amino)-2-methylpropanoate (**6**) as a white solid (140.6 mg, 76%).

R*f* = 0.35 (petroleum ether/ethyl acetate = 1/1, *v/v*). **mp** 189−190 °C (petroleum ether).

^{1}H NMR (400 MHz, DMSO-*d*$_6$) δ 9.97 (s, 1H), 7.98 (s, 1H), 7.57 (d, *J* = 8.4 Hz, 2H), 7.41 – 7.26 (comp, 5H), 6.90 (d, *J* = 8.5 Hz, 2H), 5.08 (s, 2H), 2.05 (s, 3H), 1.49 (s, 6H).

^{13}C NMR (101 MHz, DMSO-*d*$_6$) δ 173.3, 168.2, 155.3, 145.9, 136.9, 136.8, 128.3 (2C), 127.8 (3C), 121.6 (2C), 119.8 (2C), 65.4, 55.6, 24.9 (2C), 23.9.

HRMS−ESI (m/z) for $C_{20}H_{22}N_2O_5$ $[M + H]^+$: calcd 371.1602, found 371.1610.

5.3 One-pot Preparation of *4-Acetamidophenyl 3-((4-bromobenzyl)amino)-2,2-dimethyl-3-oxopropanoate* (**7**)

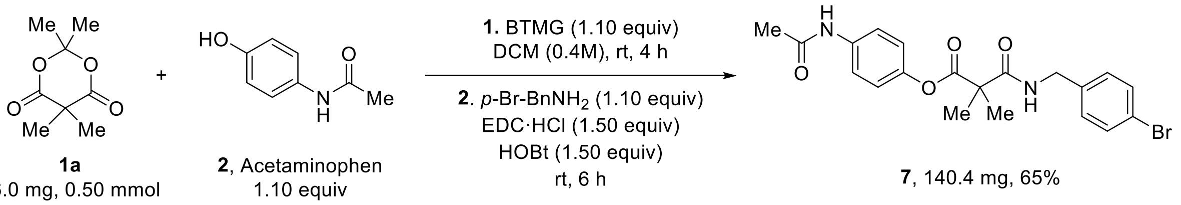

1a 86.0 mg, 0.50 mmol

2, Acetaminophen 1.10 equiv

7, 140.4 mg, 65%

In a 4.0-mL vial, 2,2,5,5-tetramethyl-1,3-dioxane-4,6-dione (**1a**) (86.0 mg, 0.50 mmol, 1.00 equiv) and BTMG (94.2 mg, 0.55 mmol, 1.10 equiv) were dissolved in anhydrous DCM (1.25 mL). Then, 4-acetamidophenol (83.1 mg, 0.55 mmol, 1.10 equiv) was added, and the mixture was stirred at rt for 4 hours. Then, *p*-BrBnNH$_2$ (102.3 mg, 0.55 mmol, 1.10 equiv), EDC·HCl (143.8 mg, 0.75 mmol, 1.50

equiv), and HOBt (101.3 mg, 0.75 mmol, 1.50 equiv) were added, and the mixture was stirred at rt for 6 h. After the reaction was completed, the mixture was diluted with EA (20 mL), and washed with 2 M aqueous HCl solution (15 mL). The organic layer was separated, washed with saturated brine (20 mL), dried over anhydrous sodium sulfate, filtered, and concentrated in vacuo. The crude product was then purified by silica gel column chromatography (petroleum ether/ethyl acetate = 3/1, *v/v*), which afforded 4-acetamidophenyl 3-((4-bromobenzyl)amino)-2,2-dimethyl-3-oxopropanoate (**7**) as a white solid (140.4 mg, 65%).

$\mathbf{R_f}$ = 0.35 (petroleum ether/ethyl acetate = 3/1, *v/v*). **mp** 179−180 °C (petroleum ether).

^{1}H NMR (400 MHz, DMSO-d_6) δ 9.97 (s, 1H), 8.50 (t, J = 6.0 Hz, 1H), 7.59 (d, J = 8.9 Hz, 2H), 7.48 (d, J = 8.3 Hz, 2H), 7.23 (d, J = 8.4 Hz, 2H), 6.95 (d, J = 8.9 Hz, 2H), 4.29 (d, J = 5.9 Hz, 1H), 2.04 (s, 3H), 1.49 (s, 6H).

^{13}C NMR (101 MHz, DMSO-d_6) δ 172.2, 171.5, 168.2, 145.6, 139.0, 137.0, 131.0 (2C), 129.2 (2C), 121.5 (2C), 119.8 (2C), 119.7, 50.0, 41.9, 23.9, 22.7 (2C).

HRMS−ESI (m/z) for $C_{20}H_{21}BrN_2O_4$ $[M + H]^+$: calcd 433.0758 (^{79}Br), 435.0737 (^{81}Br), found 433.0766 (^{79}Br), 435.0745 (^{81}Br).

6. *Preliminary Mechanistic Experiments*

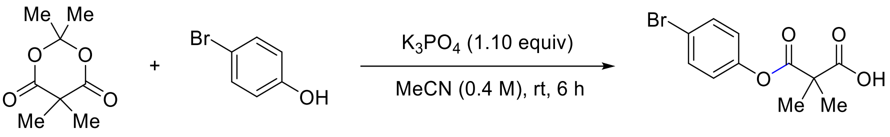

1a, 86.0 mg, 0.50 mmol **2a**, 1.10 equiv **3a'**, 119.3 mg, 90%

In a 4.0-mL vial, 5,5-dimethyl-Meldrum's acid **1a** (86.0 mg, 0.50 mmol, 1.00 equiv), anhydrous K_3PO_4 (116.7 mg, 0.55 mmol, 1.10 equiv) were dissolved in anhydros MeCN (1.25 mL). The 4-bromophenol **2a** (95.2 mg, 0.55 mmol, 1.10 equiv) was added and stirred at rt for 6 hours. After the reaction was completed, the mixture was diluted with DCM (20 mL), washed with 2 M aqueous HCl solution (20 mL). The organic layer was separated, washed with saturated brine (20 mL), dried over anhydrous sodium sulfate, filtered, and concentrated in vacuo. The crude product was then purified by silica gel column chromatography.

*3-(4-Bromophenoxy)-2,2-dimethyl-3-oxopropanoic acid (**3a'**)*

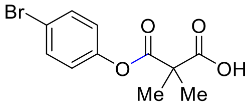

Compound **3a′** was purified by flash column chromatography (dichloromethane/methanol = 40/1, *v/v*) afforded 3-(4-bromophenoxy)-2,2-dimethyl-3-oxopropanoic acid (**3a′**) as a white solid (119.3 mg, 90%).

$\mathbf{R_f}$ = 0.20 (dichloromethane /methanol = 40/1, *v/v*). mp 103−104 °C (petroleum ether).

^{1}H NMR (400 MHz, $CDCl_3$) δ 7.49 (d, J = 8.8 Hz, 2H), 6.99 (d, J = 8.8 Hz, 2H), 1.62 (s, 6H).

^{13}C NMR (101 MHz, $CDCl_3$) δ 178.7, 170.9, 149.9, 132.8 (2C), 123.3 (2C), 119.5, 50.4, 22.9 (2C).

HRMS−ESI (m/z) for $C_{11}H_{11}BrO_4$ $[M - H]^-$: calcd 284.9767 (^{79}Br), 286.9747 (^{81}Br), found 284.9769 (^{79}Br), 286.9749 (^{81}Br).

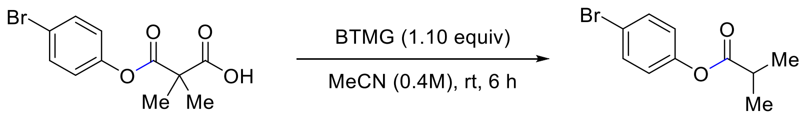

3a'
28.6 mg, 0.10 mmol

3a

In a 4.0-mL vial, 3-(4-bromophenoxy)-2,2-dimethyl-3-oxopropanoic acid **3a′** (28.6 mg, 0.10 mmol, 1.00 equiv), BTMG (18.8 mg, 0.11 mmol, 1.10 equiv) were dissolved in anhydrous MeCN (250.0 μL). The mixture was stirred at rt for 6 hours. The presence of **3a** was then confirmed by TLC and ^{1}H NMR analysis.

7. *References*

1. Armarego, W. L. F.; Perrin, D. D. Purification of Laboratory Chemicals, 4th ed.; Butterworth-Heinemann: Oxford, **1997**.
2. Dumas, A. M.; Fillion, E. Meldrum's Acids and 5-Alkylidene Meldrum's Acids in Catalytic Carbon−Carbon Bond-Forming Processes. *Acc. Chem. Res.* **2010**, *43*, 440–454. DOI: 10.1021/ar900229z
3. More, S.-V.; Chang, T.-T.; Chiao, Y.-P.; Jao, S-C.; Lu, C-K.; Li. W.-S. Glycosylation enhances the anti-migratory activities of isomalyngamide A analogs. *Eur. J. Med. Chem.* **2013**, *64*, 169–178. DOI: 10.1016/j.ejmech.2013.03.044
4. Kawamoto, K.; Zhong, M.; Wang, R.; Olsen, B. D.; Johnson, J. A. Loops versus Branch Functionality in Model Click Hydrogels. *Macromolecules* **2015**, *48*, 8980–8988. DOI: 10.1021/acs.macromol.5b02243
5. Li, J.-S.; Da, Y.-D.; Chen, G.-Q.; Yang, Q.; Li, Z.-W.; Yang, F.; Huang, P.-M. Solvent-, and Catalyst-Free Acylation of Anilines with Meldrum's Acids: A Neat Access to Anilides. *Chemistry Select*. **2017**, *2*, 1770–1773. DOI: 10.1002/slct.201601965
6. Sakakura, A.; Kawajiri, K.; Ohkubo, T.; Kosugi, Y.; Ishihara, K. Widely Useful DMAP-Catalyzed Esterification under Auxiliary Base- and Solvent-Free Conditions. *J. Am. Chem. Soc.* **2007**, *129*, 14775–14779. DOI: 10.1021/ja075824w
7. Mensah, E.; Earl, L. Mild and Highly Efficient Copper(I) Inspired Acylation of Alcohols and Polyols. *Catalysts* **2017**, *7*, 33. DOI: 10.3390/catal7010033
8. Yang, C.-H.; Fan, W.-W.; Liu, G.-Q.; Duan, L.; Li, L.; Li, Y.-M. On the Understanding of $BF_3 \cdot Et_2O$-Promoted Intra- and Intermolecular Amination and Oxygenation of Unfunctionalized Olefins. *RSC Adv*. **2015**, *5*, 61081–61093. DOI: 10.1039/C5RA10513G

8. *NMR Spectra*

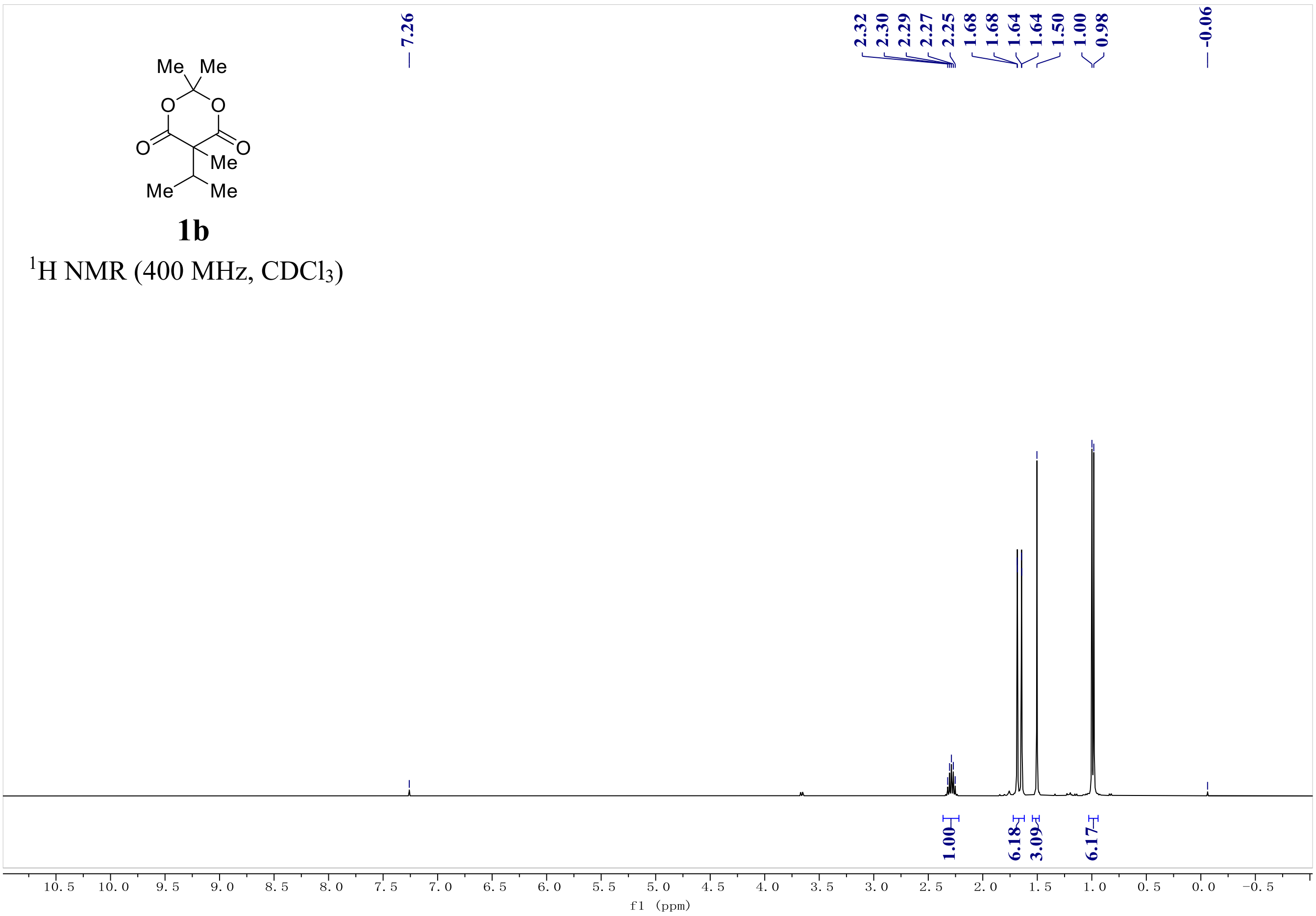

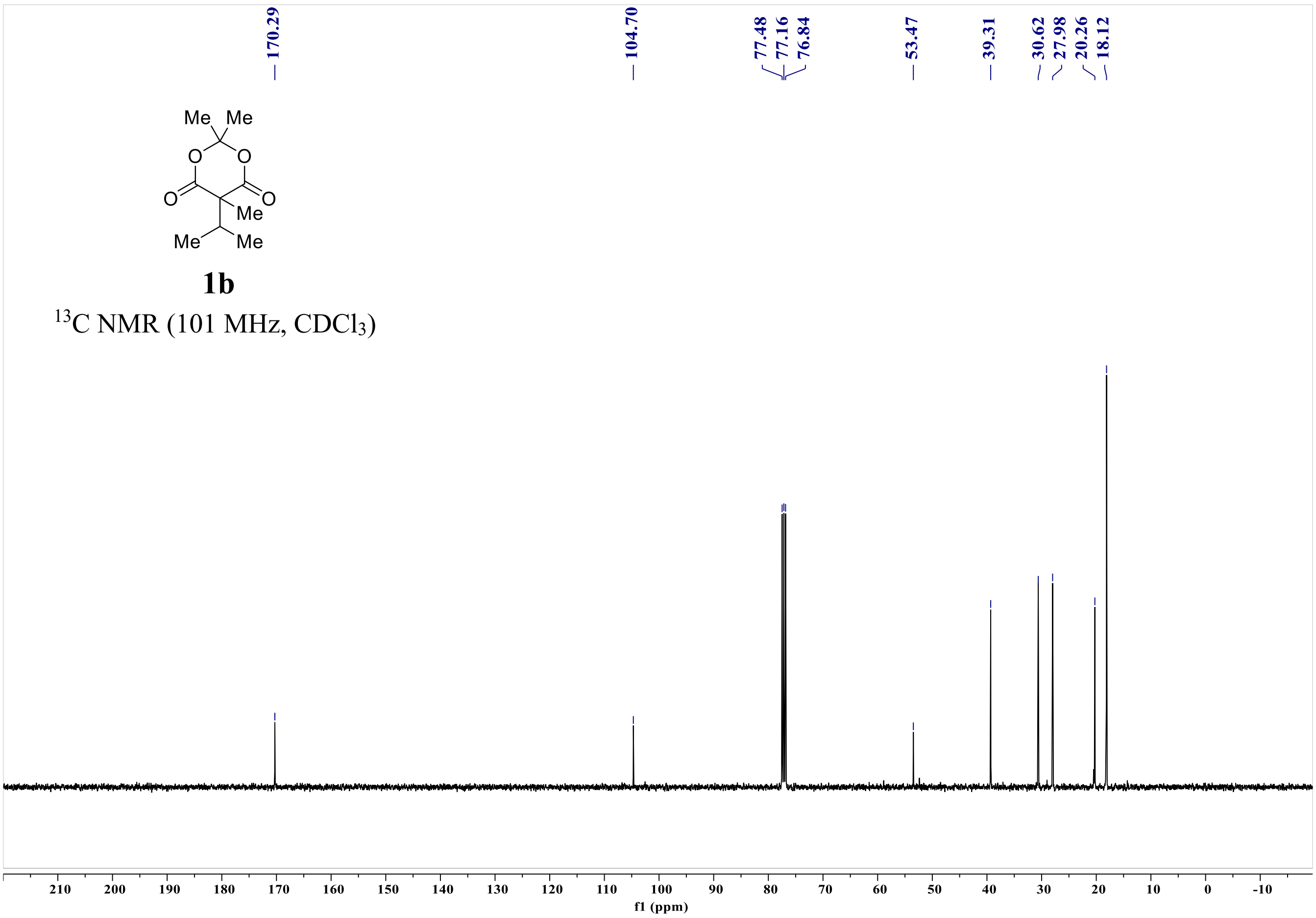

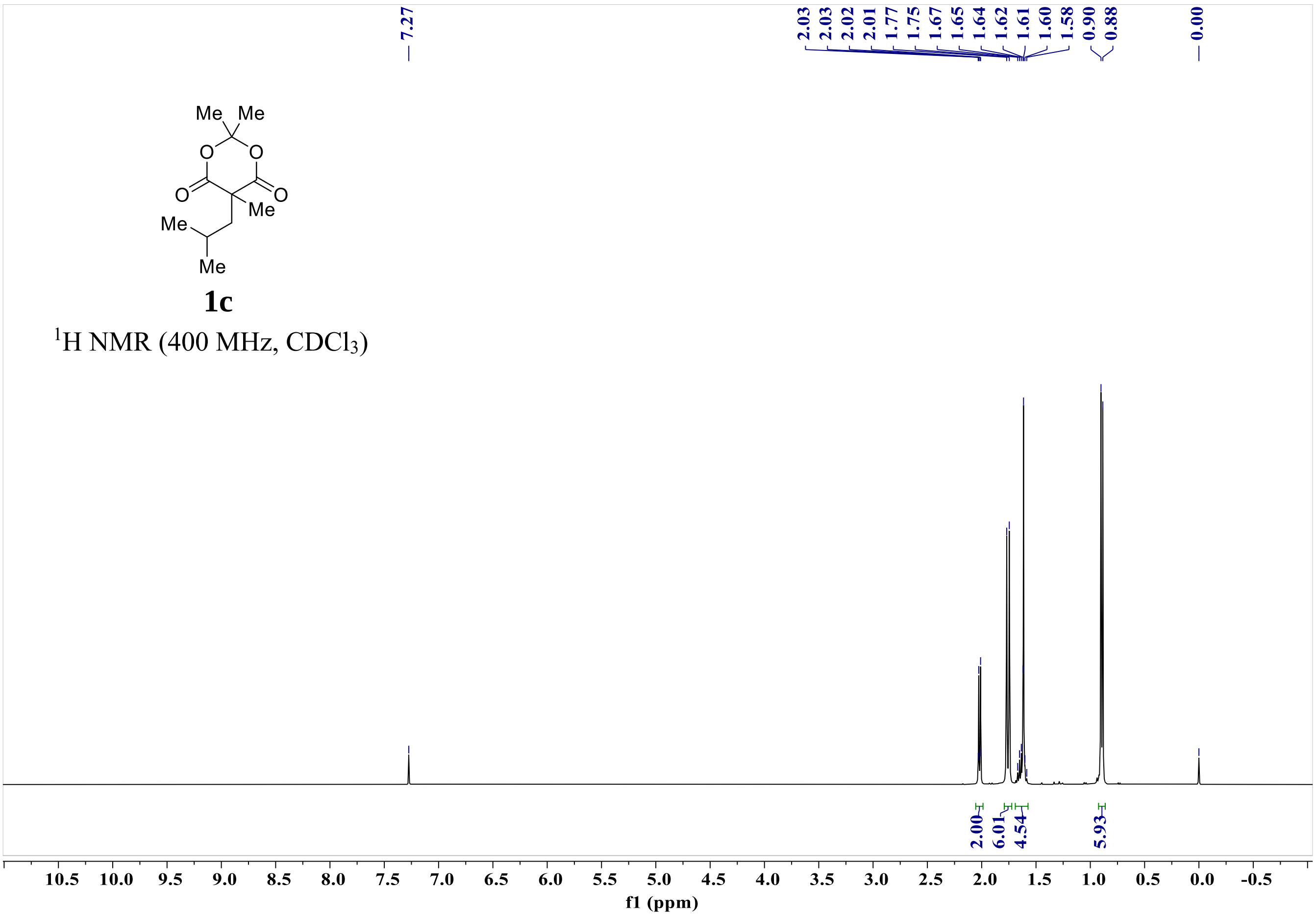

1c

^{1}H NMR (400 MHz, $CDCl_3$)

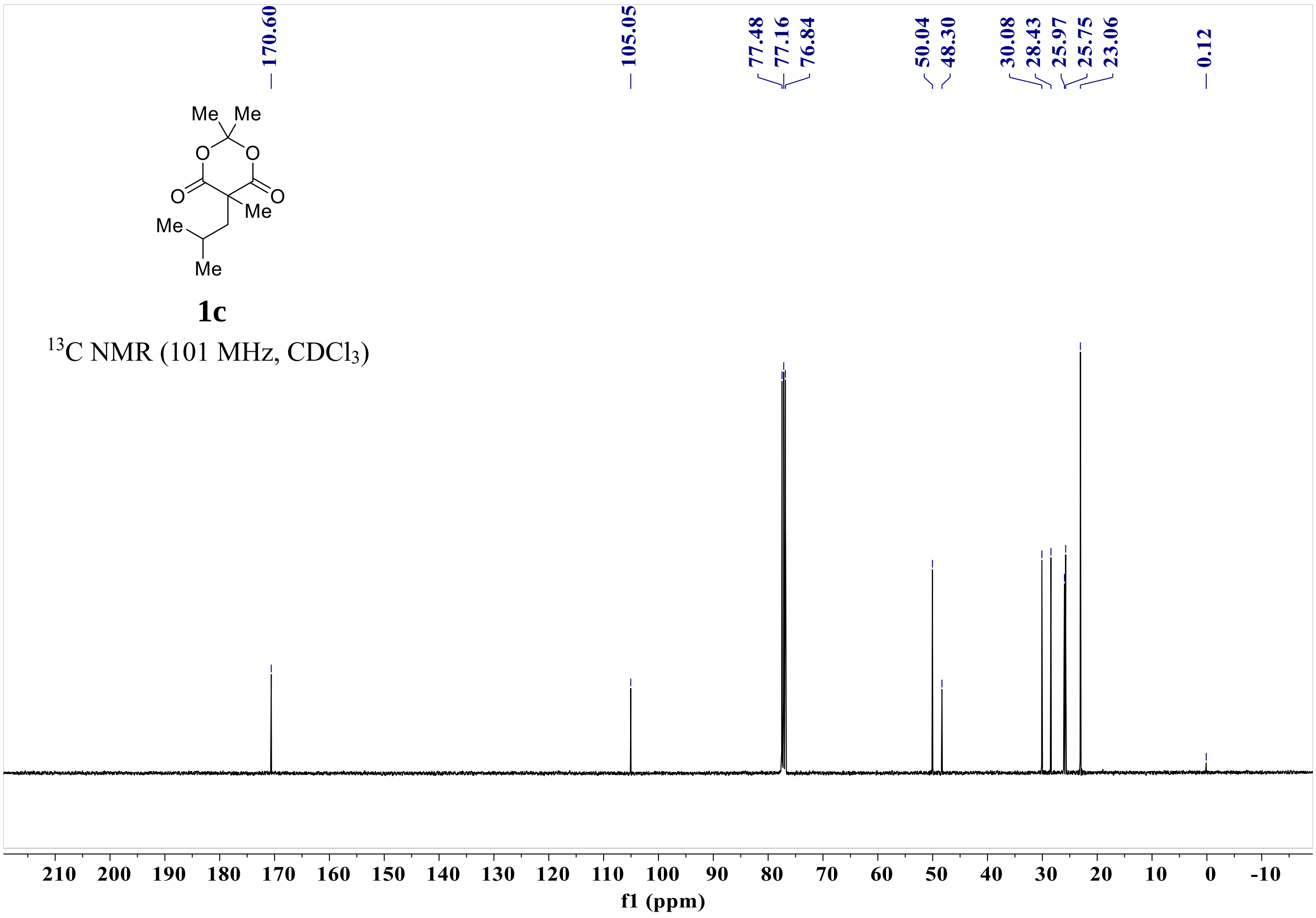

1c

^{13}C NMR (101 MHz, $CDCl_3$)

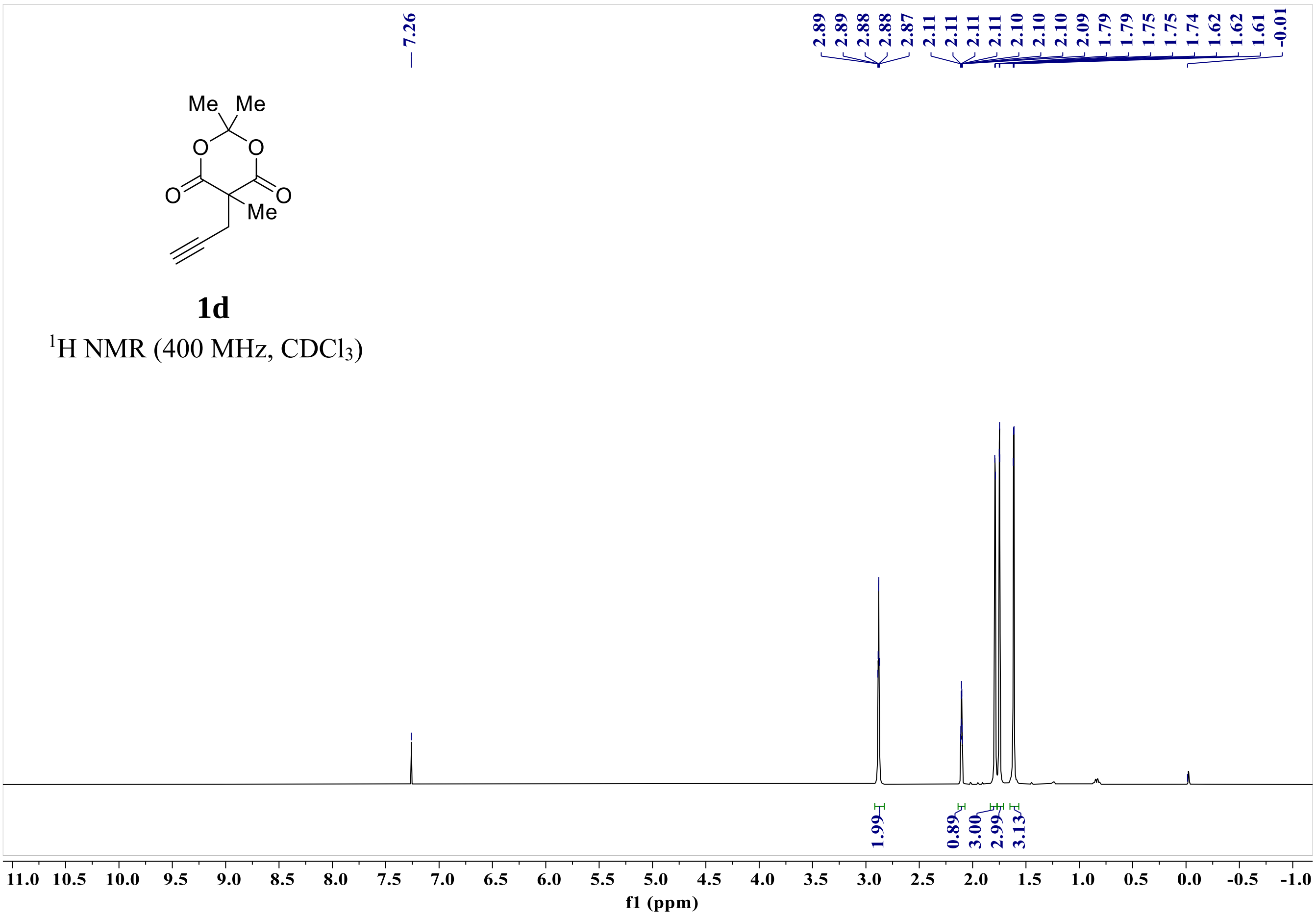
7.26
2.89 2.89 2.88 2.88 2.87 2.11 2.11 2.11 2.11 2.10 2.10 2.10 2.09 1.79 1.79 1.75 1.75 1.74 1.62 1.62 1.61 -0.01
Me Me O O O O Me
1d
1H NMR (400 MHz, CDCl3)
1.99 0.89 3.00 2.99 3.13
11.0 10.5 10.0 9.5 9.0 8.5 8.0 7.5 7.0 6.5 6.0 5.5 5.0 4.5 4.0 3.5 3.0 2.5 2.0 1.5 1.0 0.5 0.0 -0.5 -1.0
f1 (ppm)

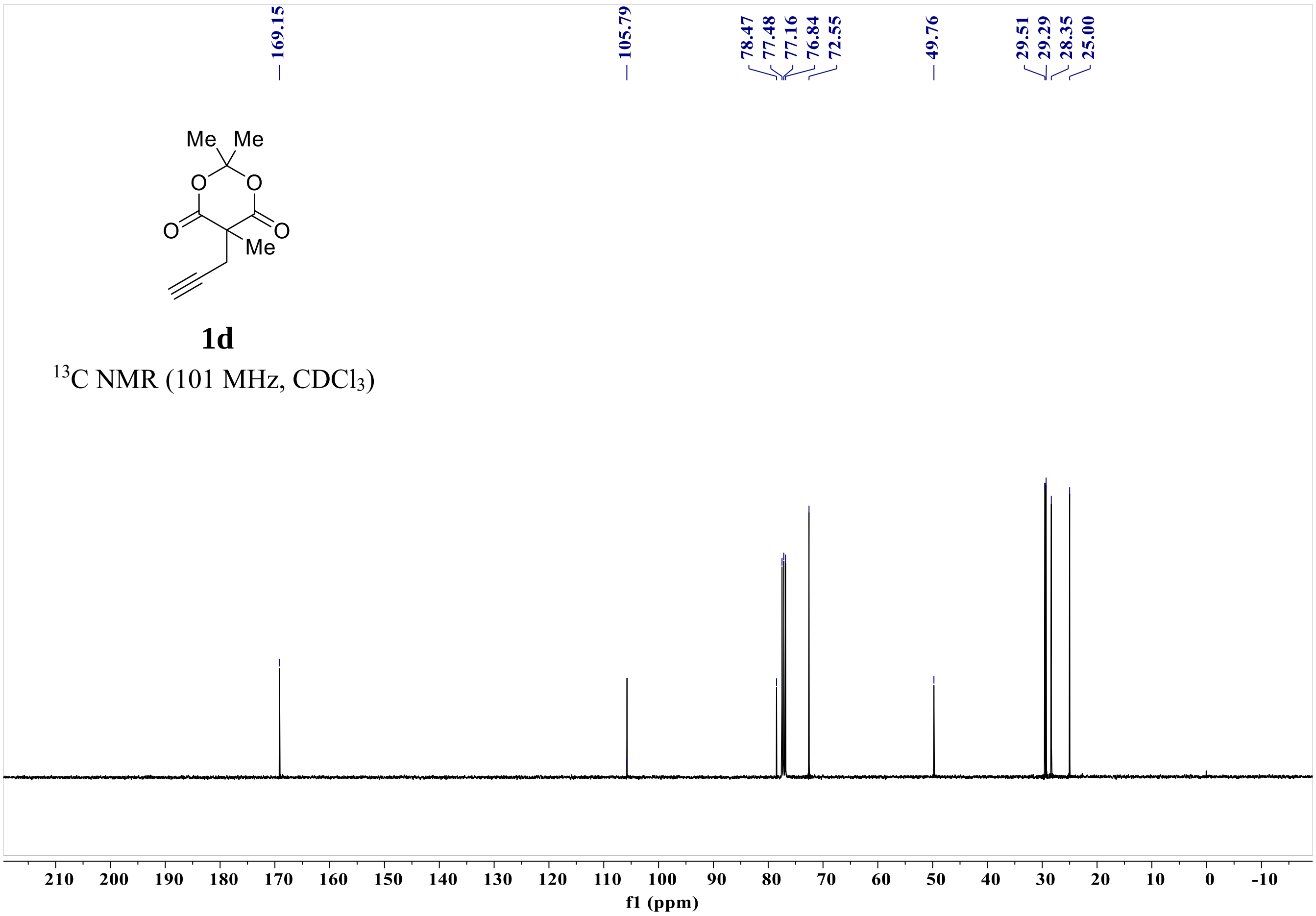
169.15
105.79
78.47 77.48 77.16 76.84 72.55
49.76
29.51 29.29 28.35 25.00
Me Me O O O O Me
1d
13C NMR (101 MHz, CDCl3)
210 200 190 180 170 160 150 140 130 120 110 100 90 80 70 60 50 40 30 20 10 0 -10
f1 (ppm)

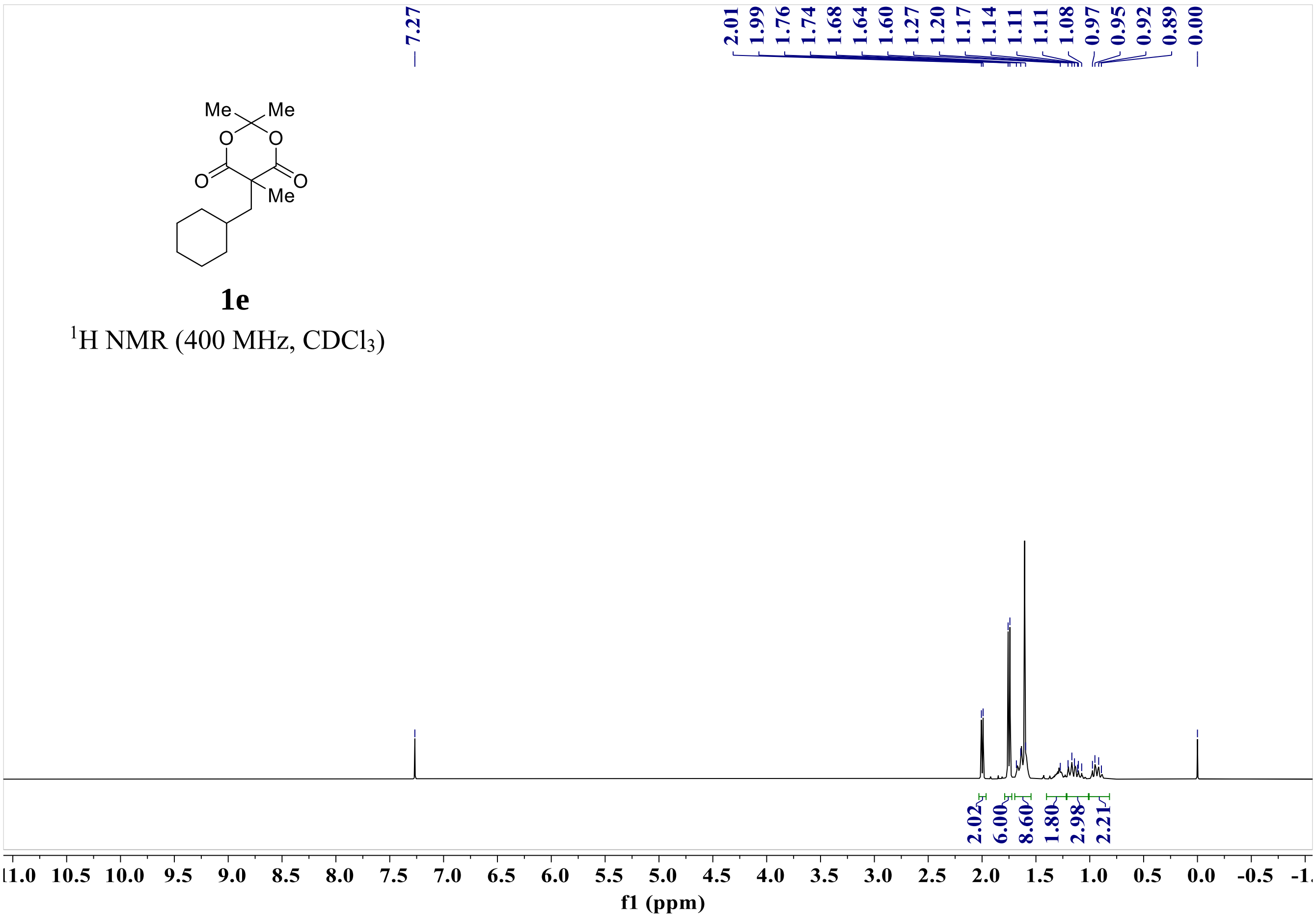
Me Me O O O O Me
1e
1H NMR (400 MHz, CDCl3)
7.27
2.01 1.99 1.76 1.74 1.68 1.64 1.60 1.27 1.20 1.17 1.14 1.11 1.11 1.08 0.97 0.95 0.92 0.89 0.00
2.02 6.00 8.60 1.80 2.98 2.21
11.0 10.5 10.0 9.5 9.0 8.5 8.0 7.5 7.0 6.5 6.0 5.5 5.0 4.5 4.0 3.5 3.0 2.5 2.0 1.5 1.0 0.5 0.0 -0.5 -1.
f1 (ppm)

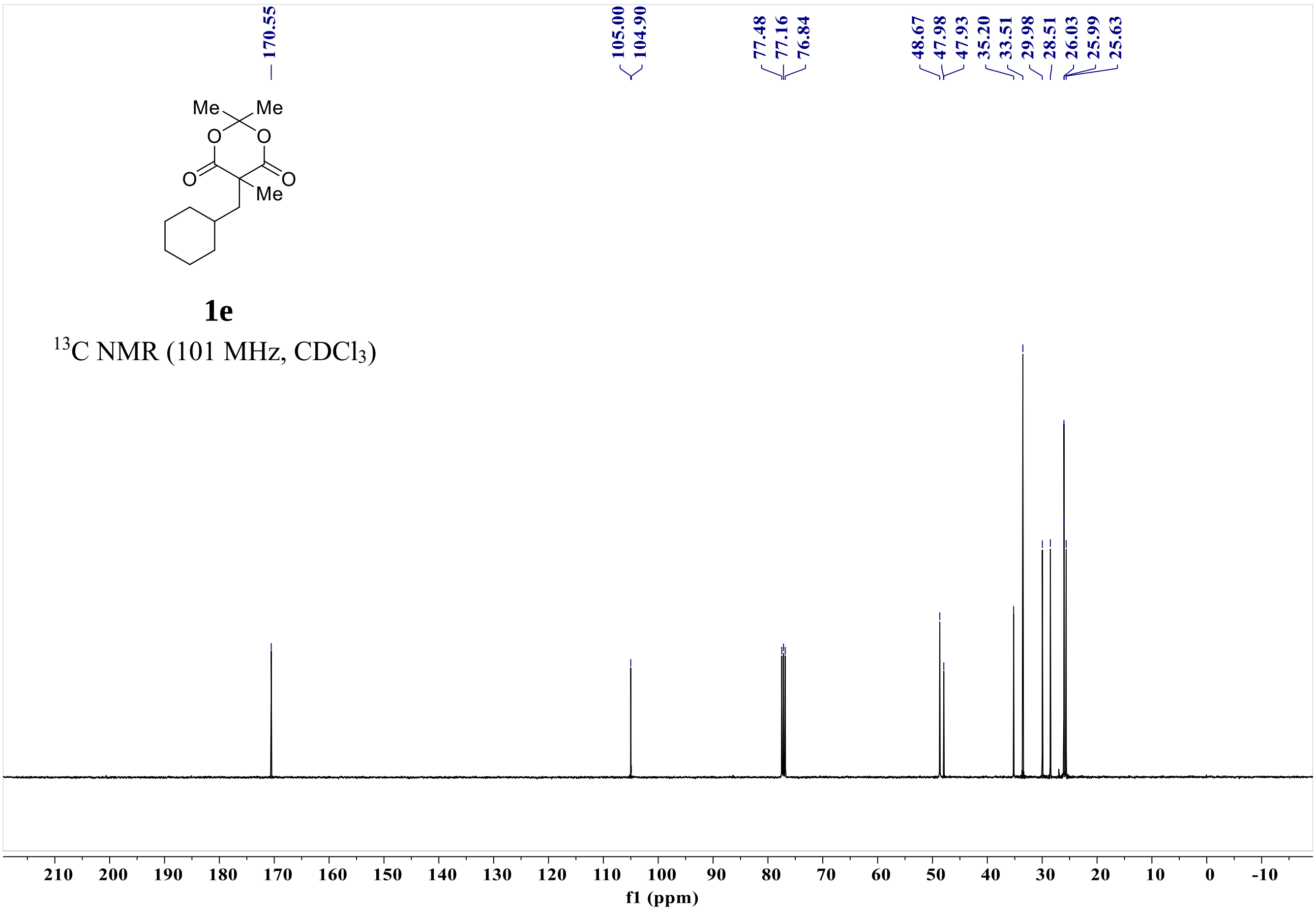
Me Me O O O O Me
1e
13C NMR (101 MHz, CDCl3)
170.55
105.00 104.90
77.48 77.16 76.84
48.67 47.98 47.93 35.20 33.51 29.98 28.51 26.03 25.99 25.63
210 200 190 180 170 160 150 140 130 120 110 100 90 80 70 60 50 40 30 20 10 0 -10
f1 (ppm)

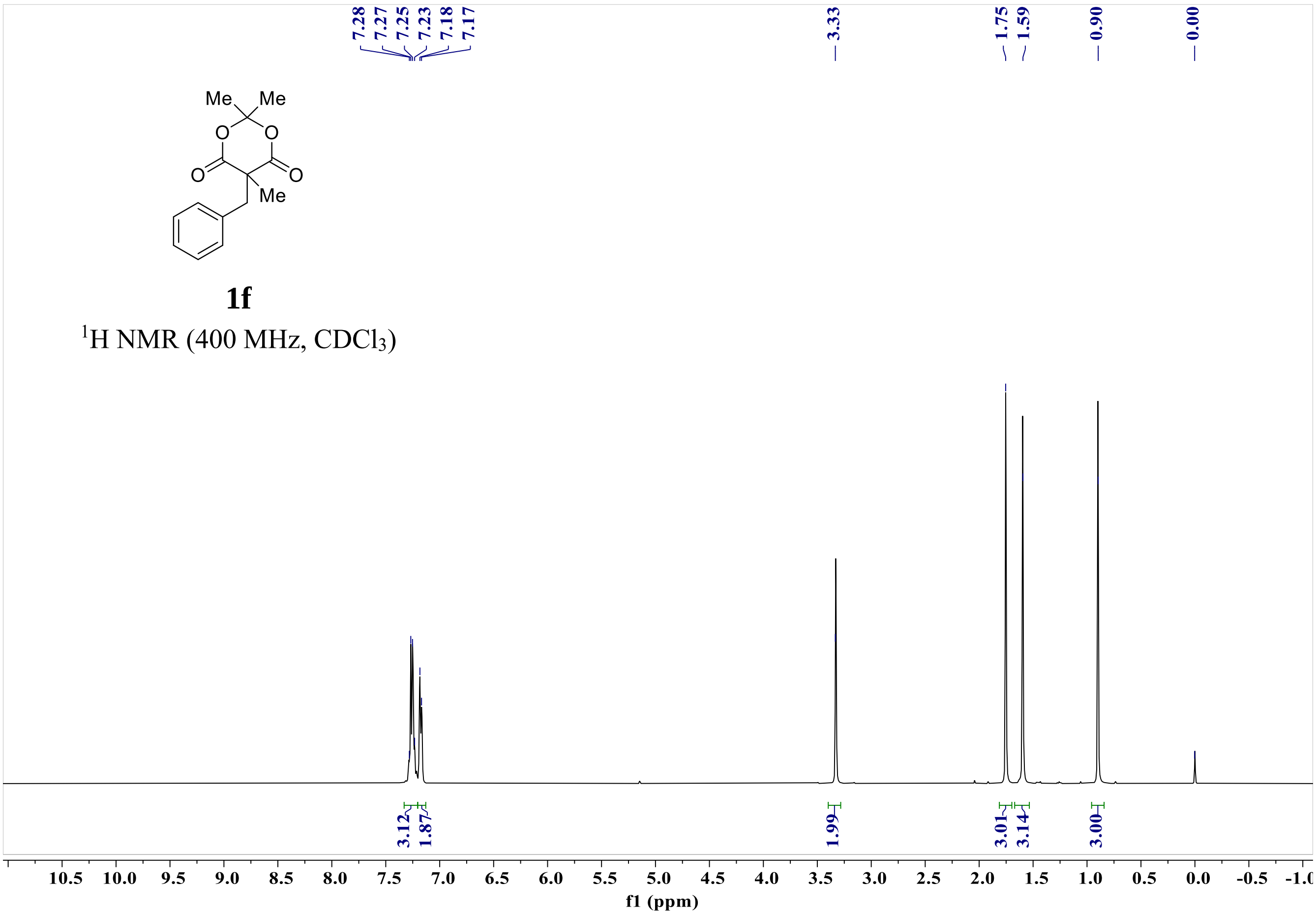
7.28
7.27
7.25
7.23
7.18
7.17
3.33
1.75
1.59
0.90
0.00
Me
Me
O
O
O
O
Me
1f
^{1}H NMR (400 MHz, CDCl$_3$)
3.12
1.87
1.99
3.01
3.14
3.00
10.5 10.0 9.5 9.0 8.5 8.0 7.5 7.0 6.5 6.0 5.5 5.0 4.5 4.0 3.5 3.0 2.5 2.0 1.5 1.0 0.5 0.0 -0.5
f1 (ppm)

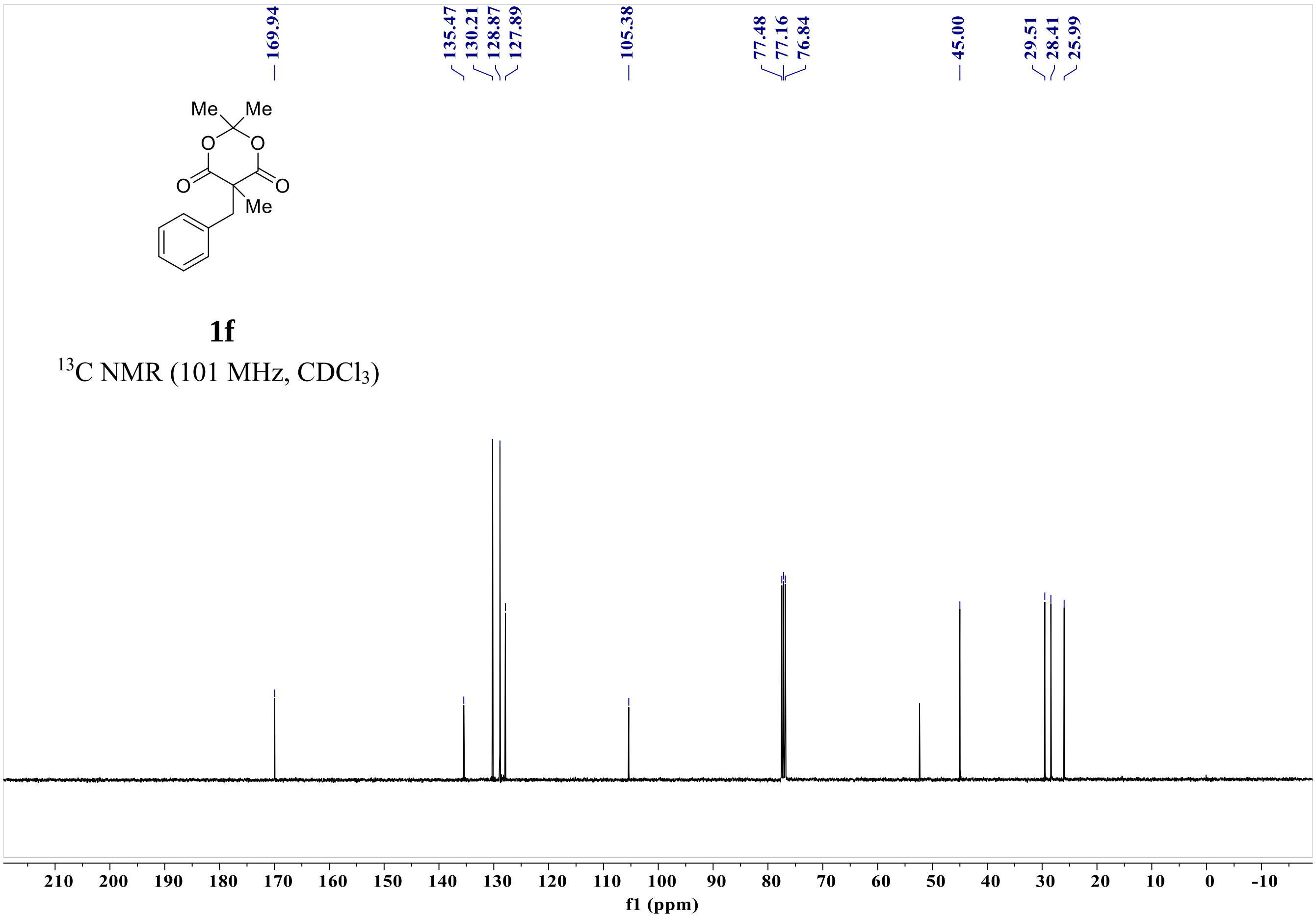
169.94
135.47
130.21
128.87
127.89
105.38
77.48
77.16
76.84
45.00
29.51
28.41
25.99
Me
Me
O
O
O
O
Me
1f
^{13}C NMR (101 MHz, CDCl$_3$)
210 200 190 180 170 160 150 140 130 120 110 100 90 80 70 60 50 40 30 20 10 0 -10
f1 (ppm)

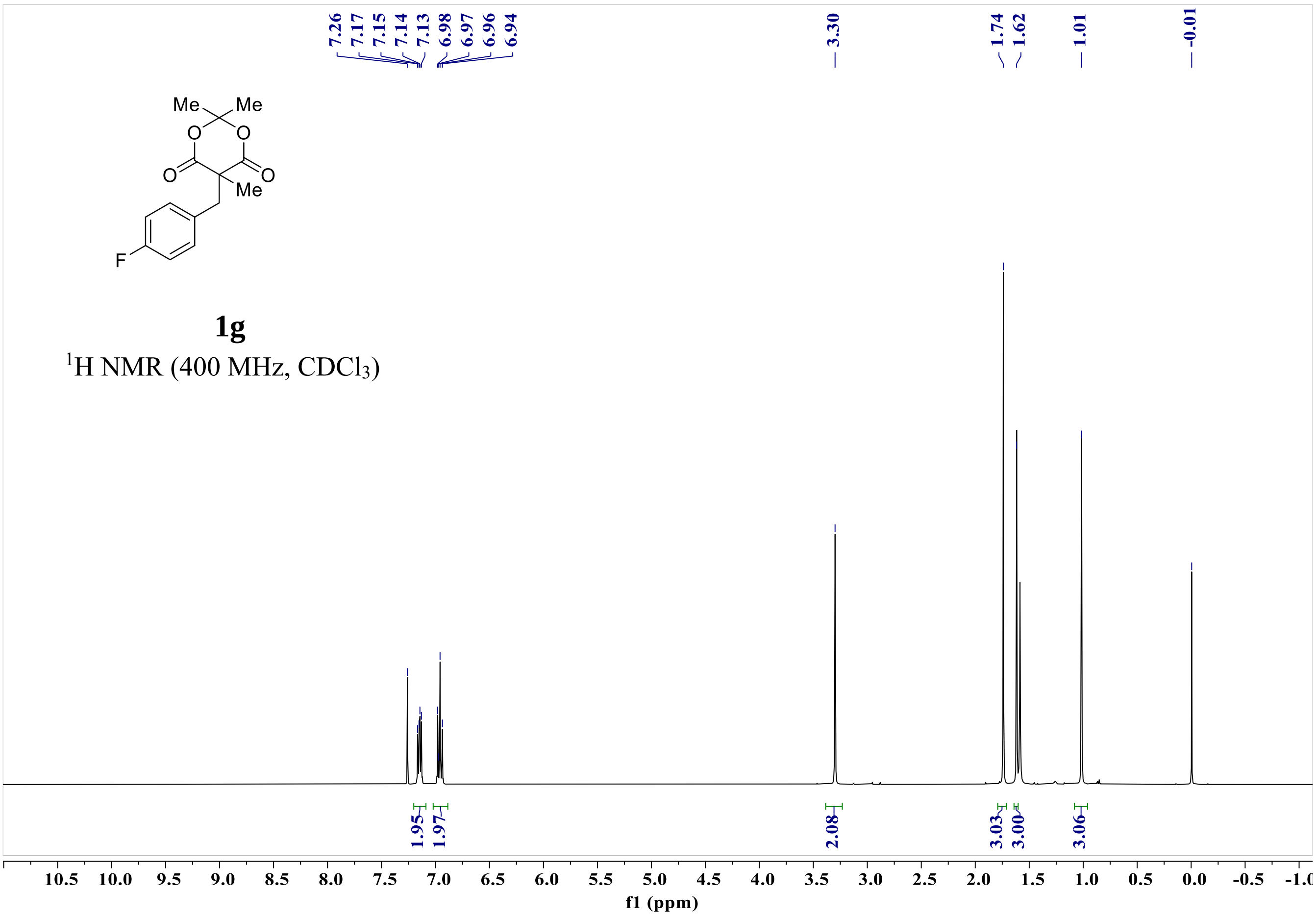

7.26
7.17
7.15
7.14
7.13
6.98
6.97
6.96
6.94
3.30
1.74
1.62
1.01
-0.01
1g
^{1}H NMR (400 MHz, CDCl$_3$)
1.95
1.97
2.08
3.03
3.00
3.06
10.5 10.0 9.5 9.0 8.5 8.0 7.5 7.0 6.5 6.0 5.5 5.0 4.5 4.0 3.5 3.0 2.5 2.0 1.5 1.0 0.5 0.0 -0.5
f1 (ppm)

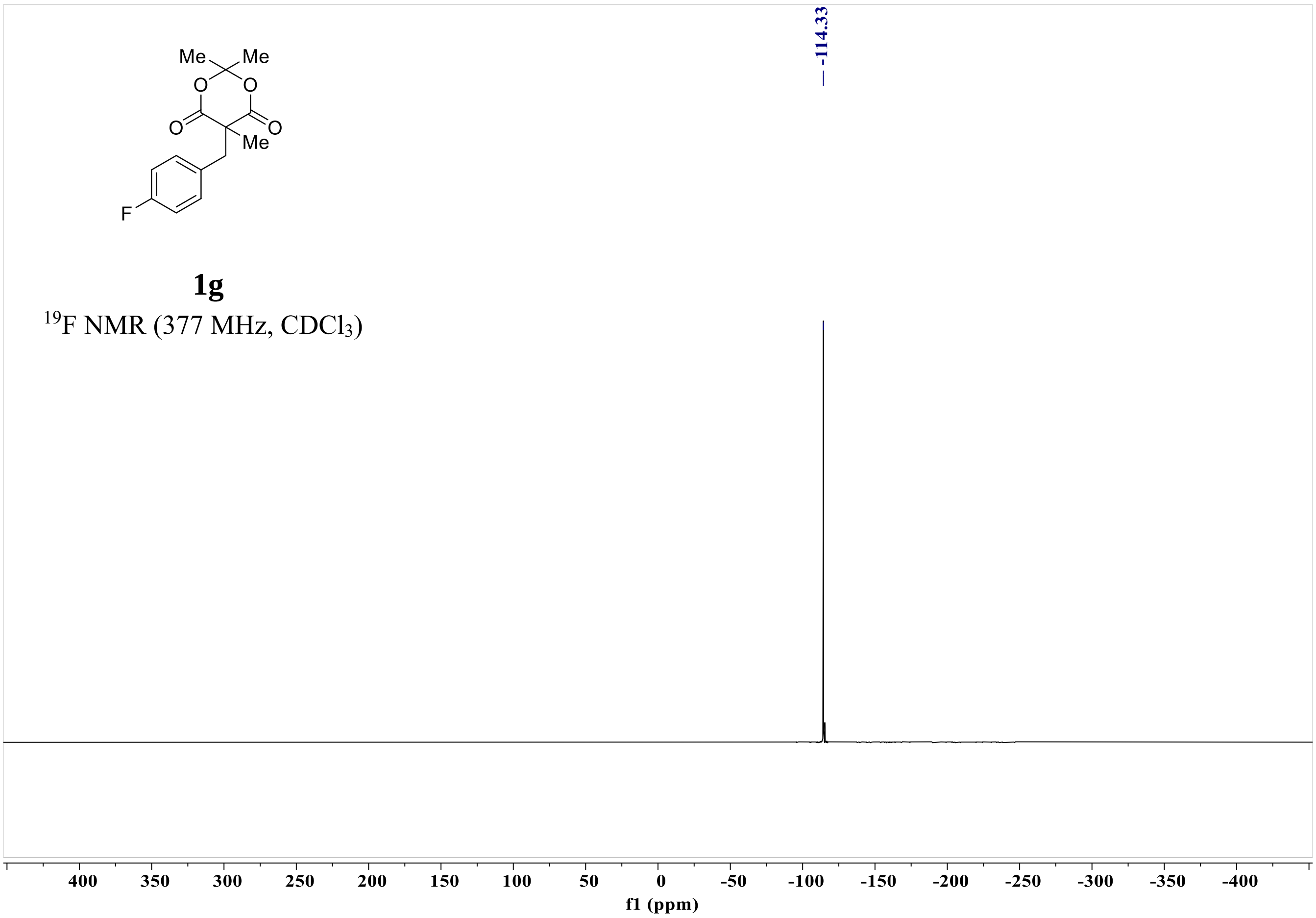

-114.33
1g
^{19}F NMR (377 MHz, CDCl$_3$)
400 350 300 250 200 150 100 50 0 -50 -100 -150 -200 -250 -300 -350 -400
f1 (ppm)

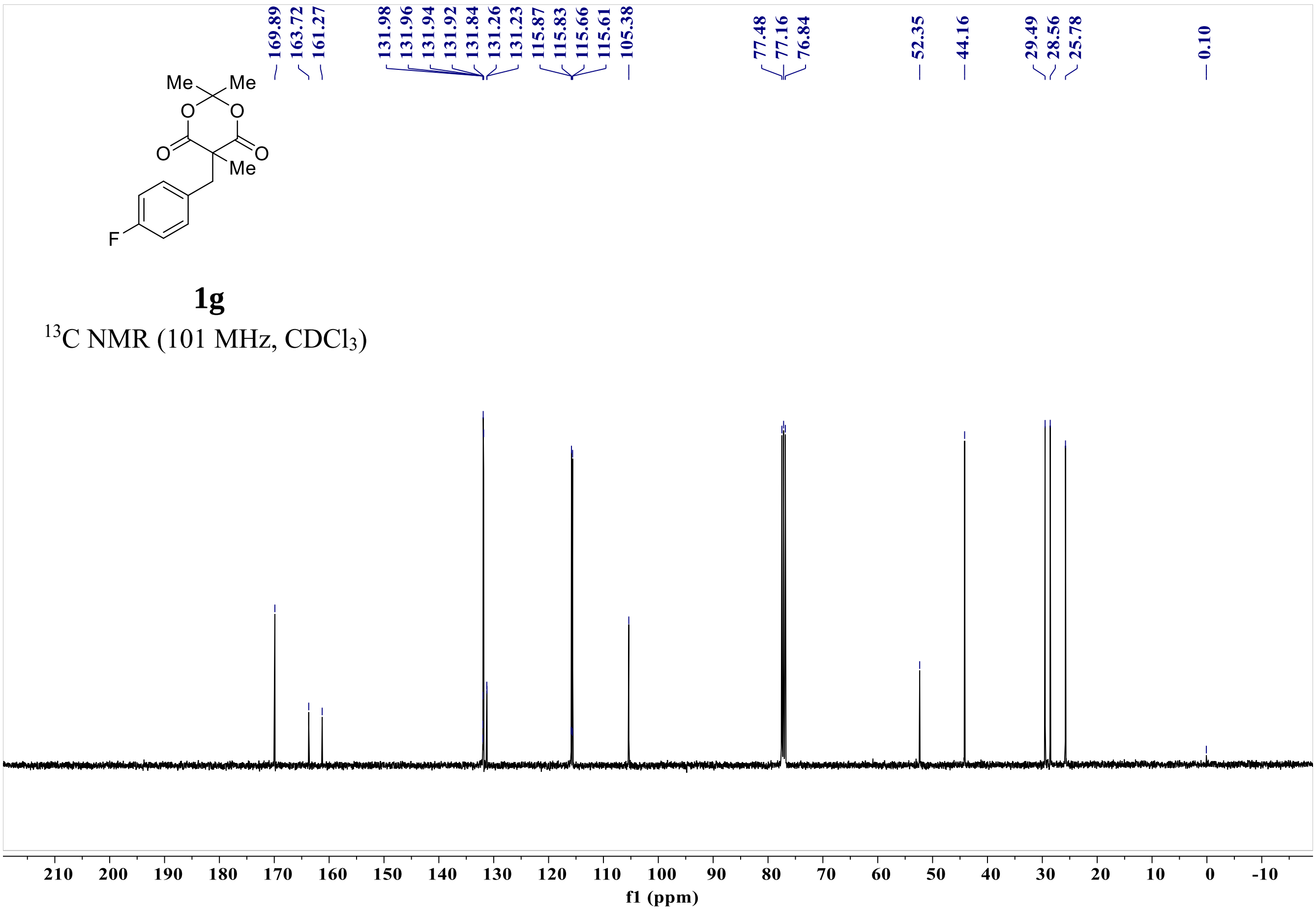

1g

^{13}C NMR (101 MHz, $CDCl_3$)

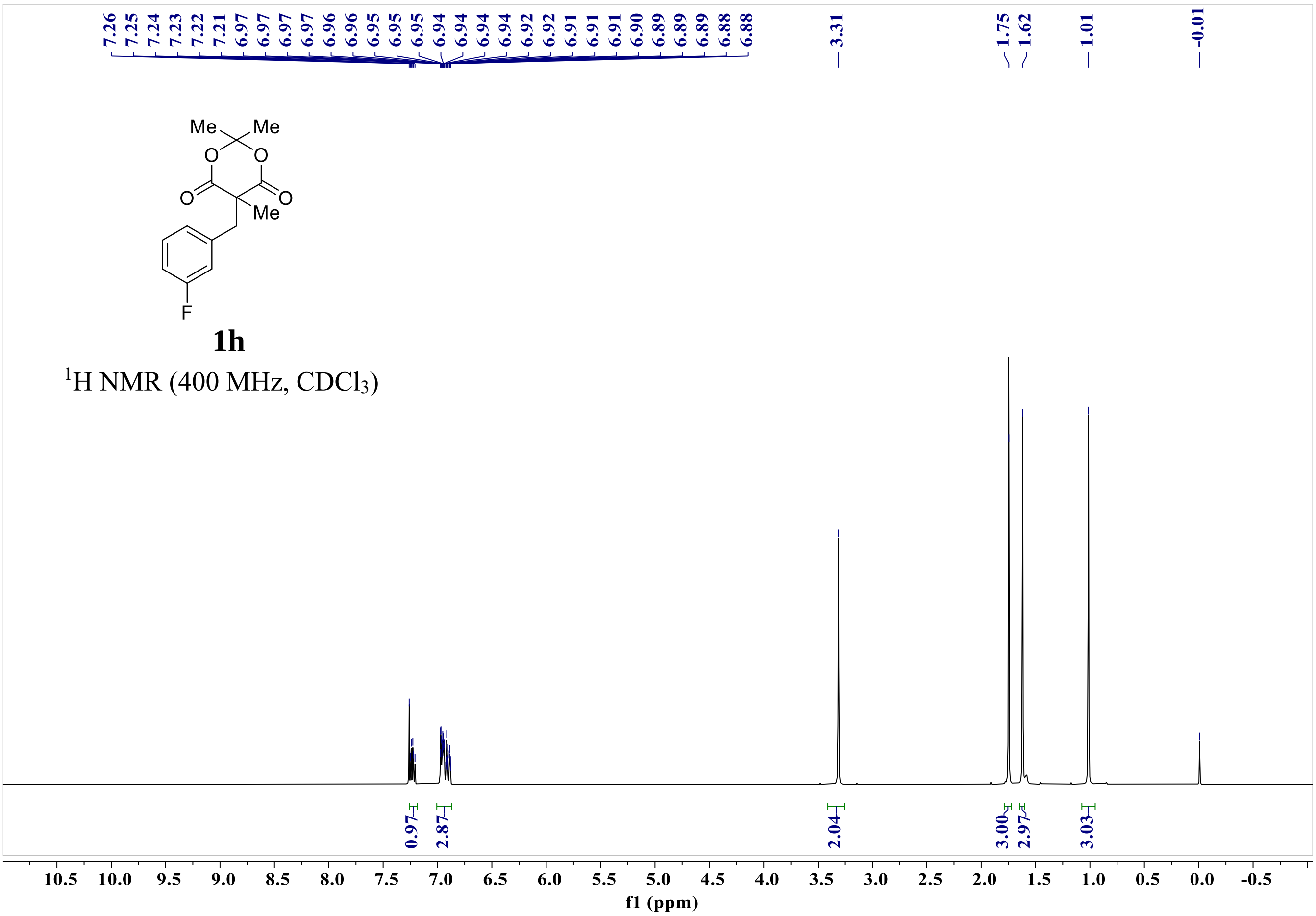

1h

^{1}H NMR (400 MHz, $CDCl_3$)

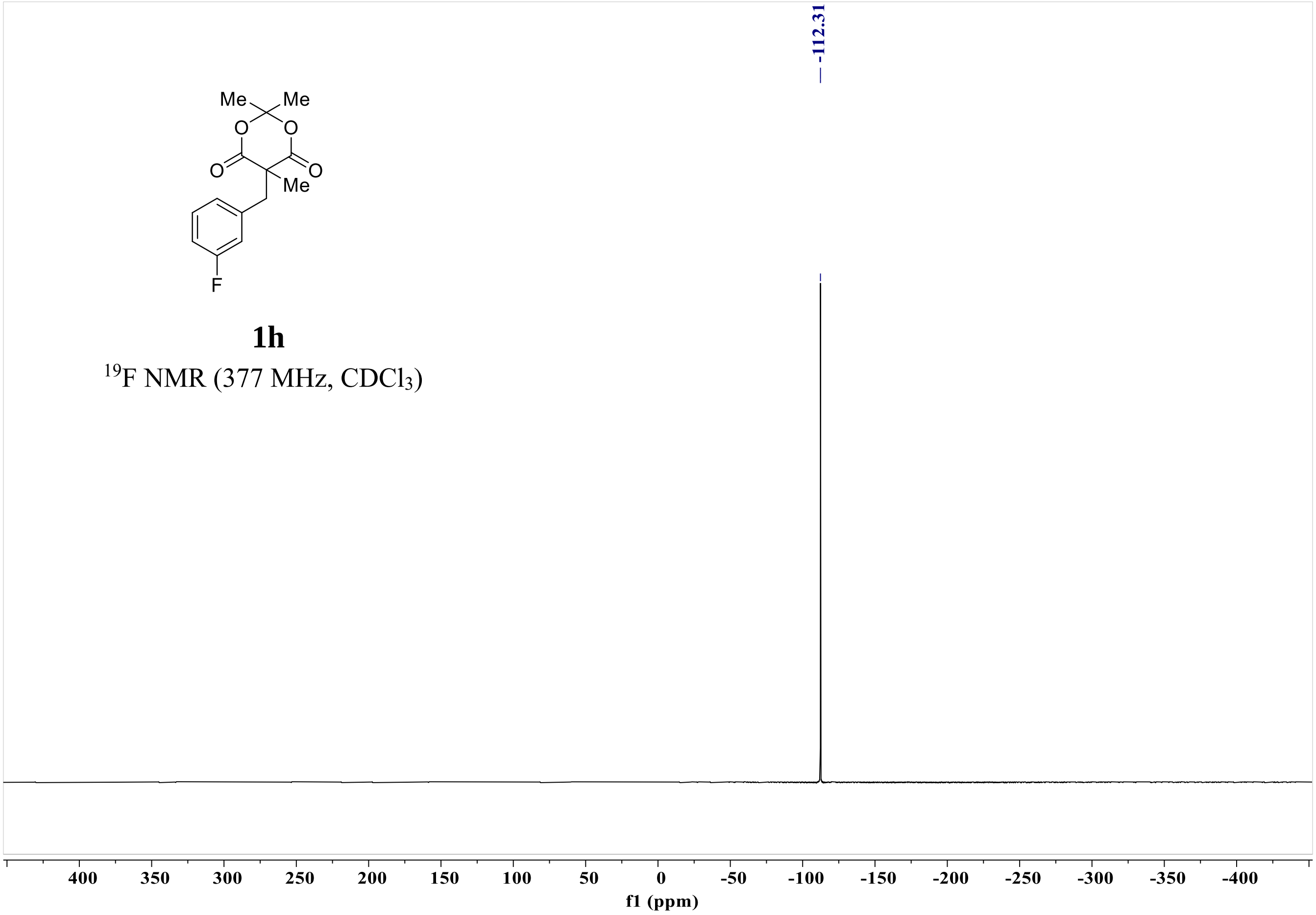
-112.31
Me
Me
O
O
O
O
Me
F
1h
^{19}F NMR (377 MHz, CDCl$_3$)
400
350
300
250
200
150
100
50
0
-50
-100
-150
-200
-250
-300
-350
-400
f1 (ppm)

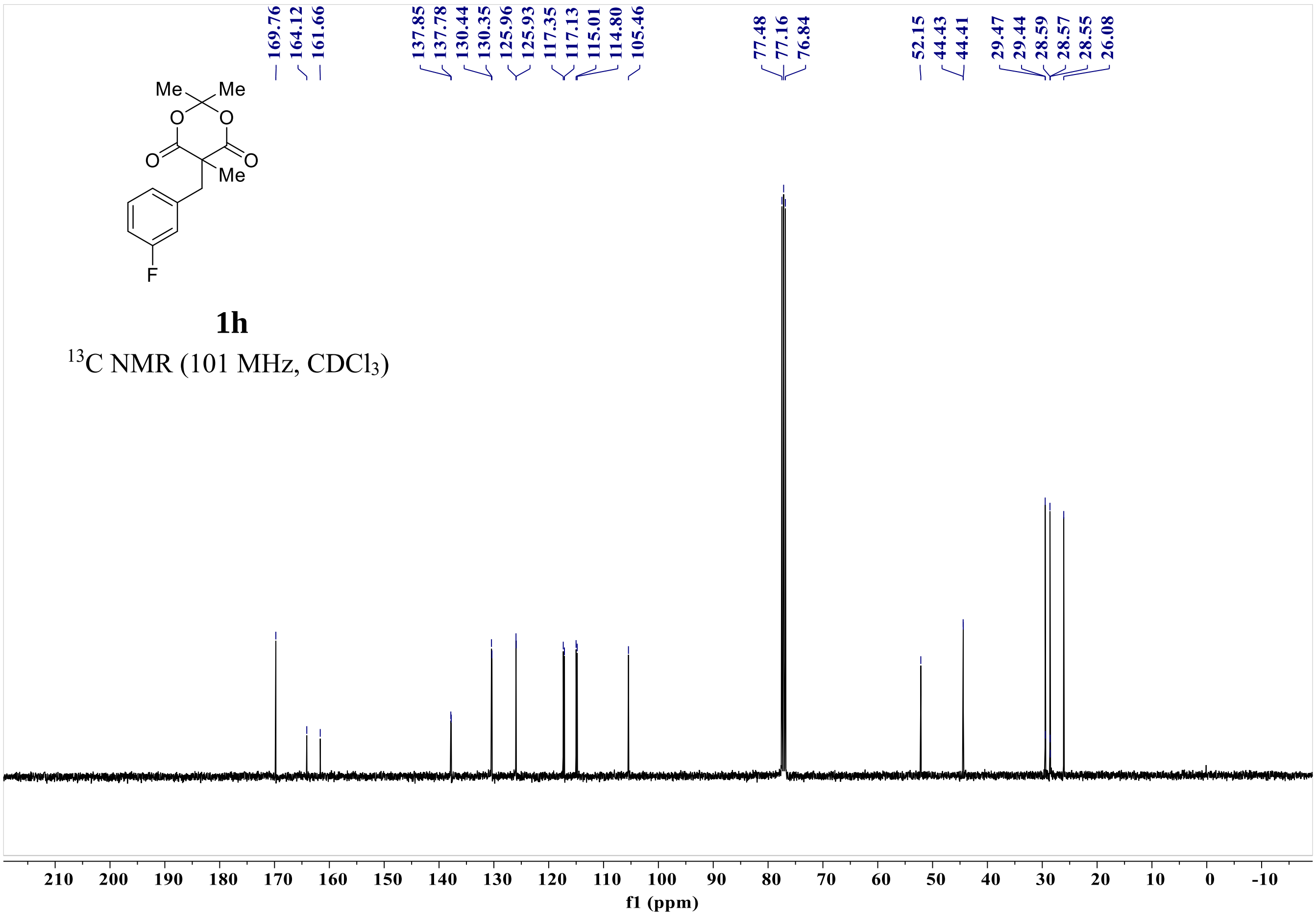
169.76
164.12
161.66
137.85
137.78
130.44
130.35
125.96
125.93
117.35
117.13
115.01
114.80
105.46
77.48
77.16
76.84
52.15
44.43
44.41
29.47
29.44
28.59
28.57
28.55
26.08
Me
Me
O
O
O
O
Me
F
1h
^{13}C NMR (101 MHz, CDCl$_3$)
210
200
190
180
170
160
150
140
130
120
110
100
90
80
70
60
50
40
30
20
10
0
-10
f1 (ppm)

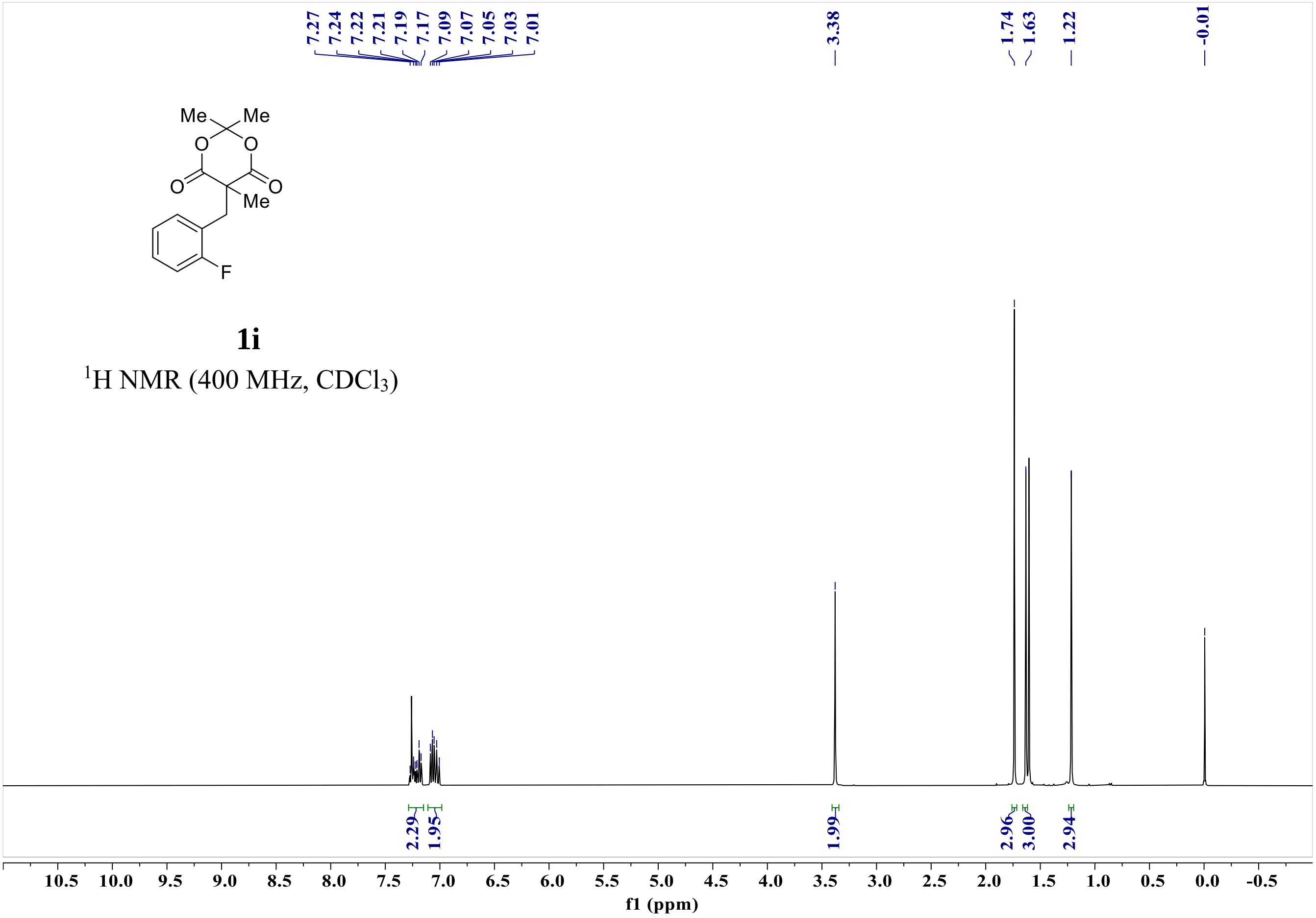
7.27 7.24 7.22 7.21 7.19 7.17 7.09 7.07 7.05 7.03 7.01
3.38
1.74 1.63
1.22
-0.01
Me Me O O O O Me F
1i
1H NMR (400 MHz, CDCl3)
2.29 1.95
1.99
2.96 3.00
2.94
10.5 10.0 9.5 9.0 8.5 8.0 7.5 7.0 6.5 6.0 5.5 5.0 4.5 4.0 3.5 3.0 2.5 2.0 1.5 1.0 0.5 0.0 -0.5
f1 (ppm)

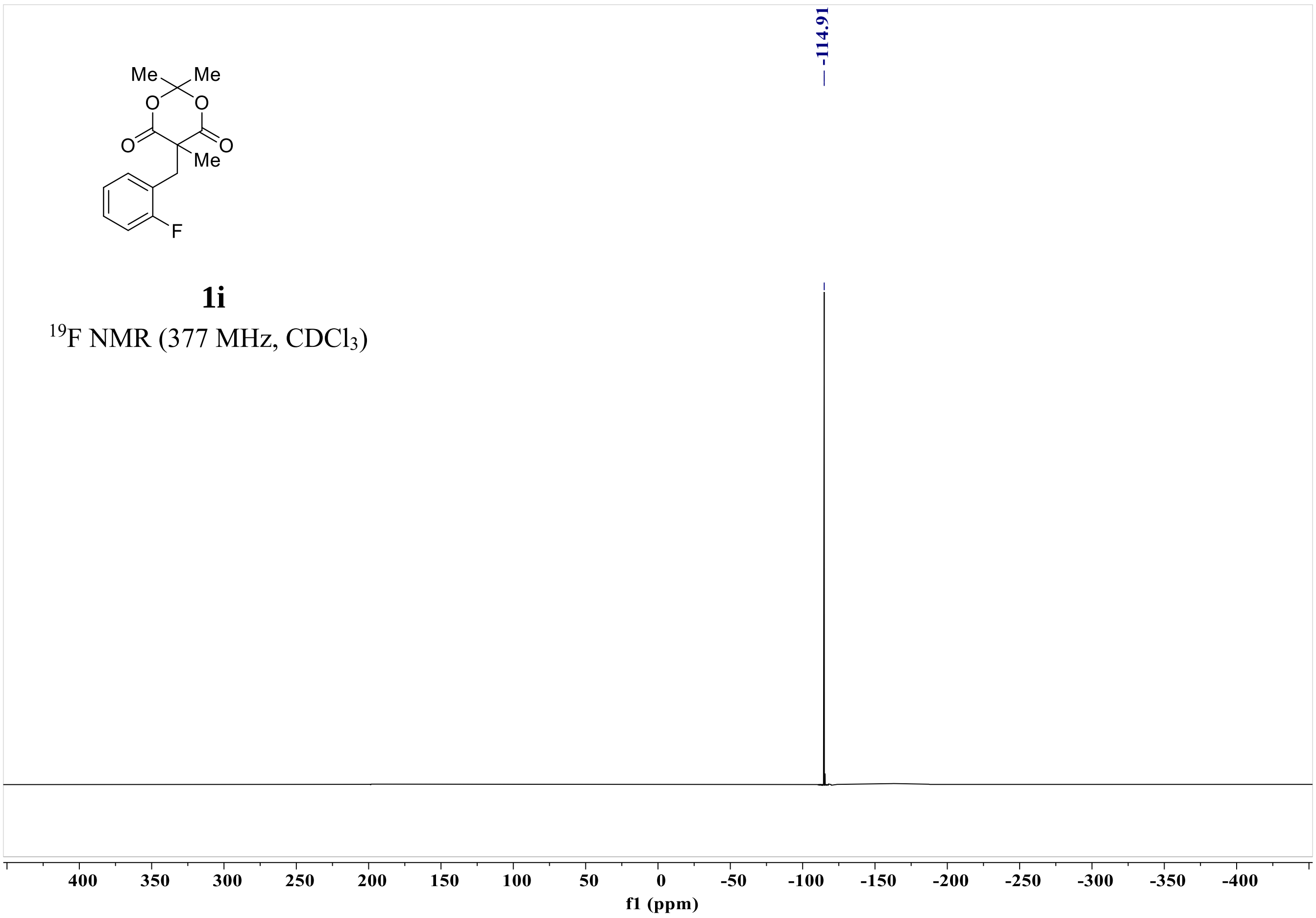
-114.91
Me Me O O O O Me F
1i
19F NMR (377 MHz, CDCl3)
400 350 300 250 200 150 100 50 0 -50 -100 -150 -200 -250 -300 -350 -400
f1 (ppm)

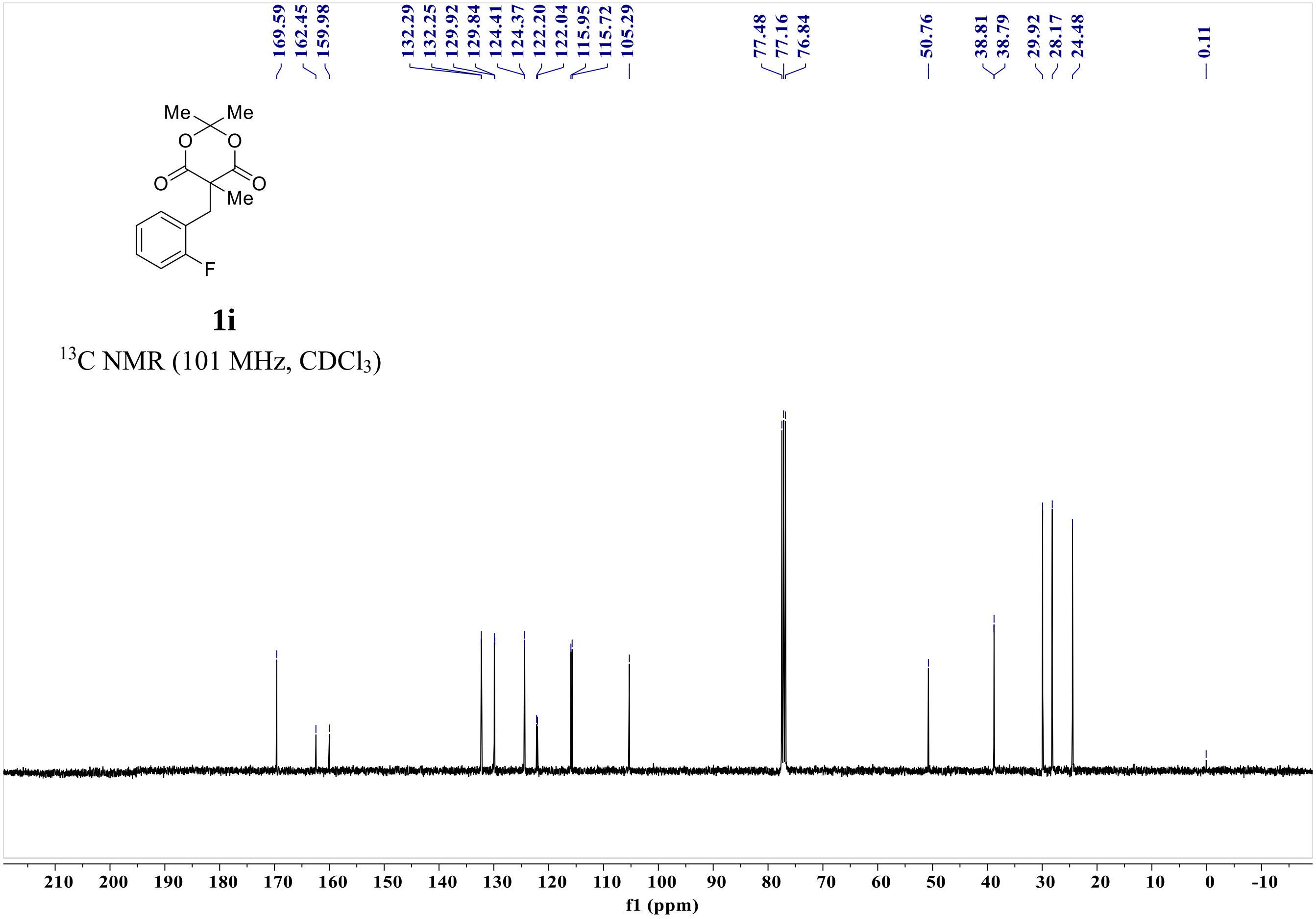

1i

^{13}C NMR (101 MHz, $CDCl_3$)

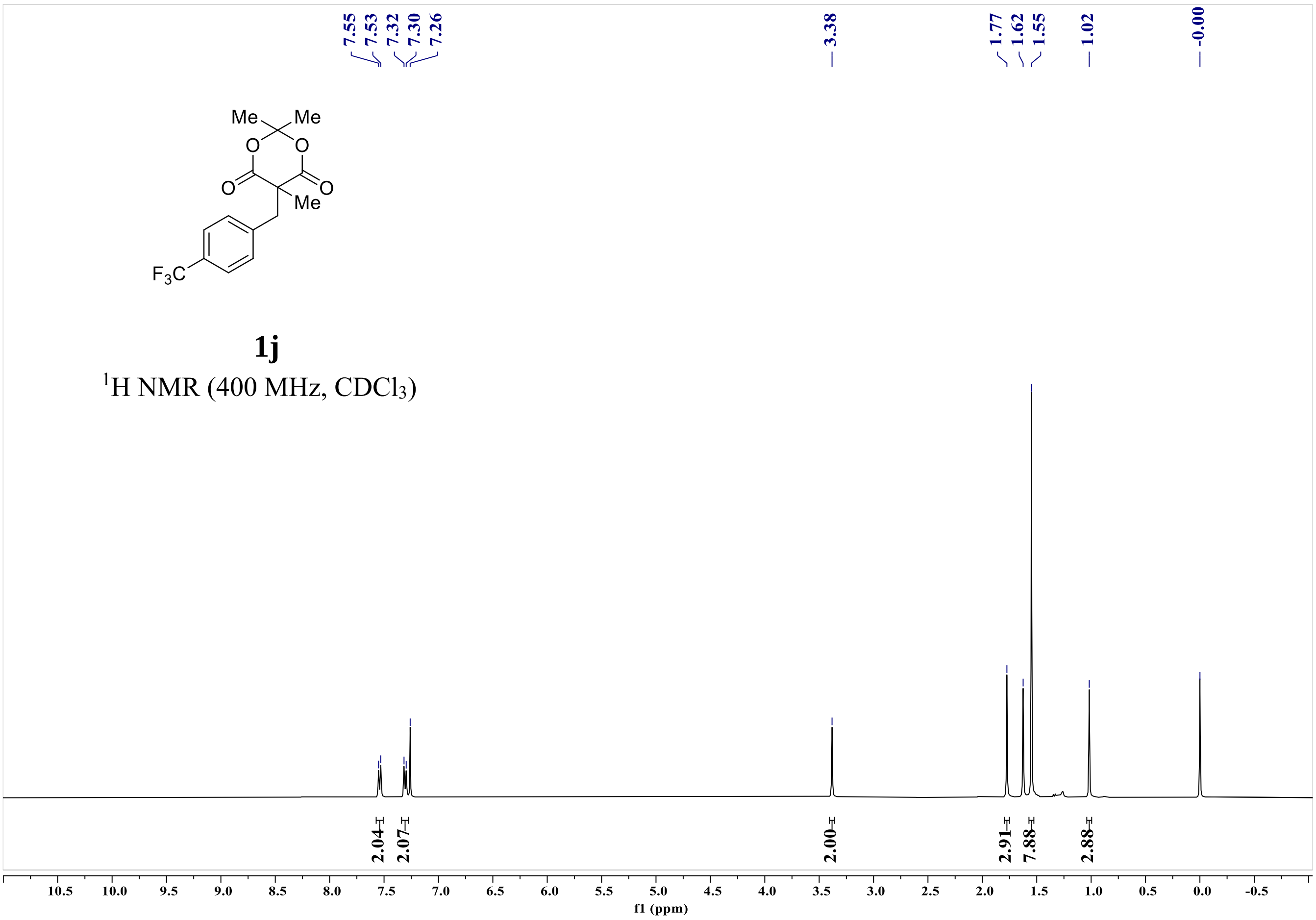

1j

^{1}H NMR (400 MHz, $CDCl_3$)

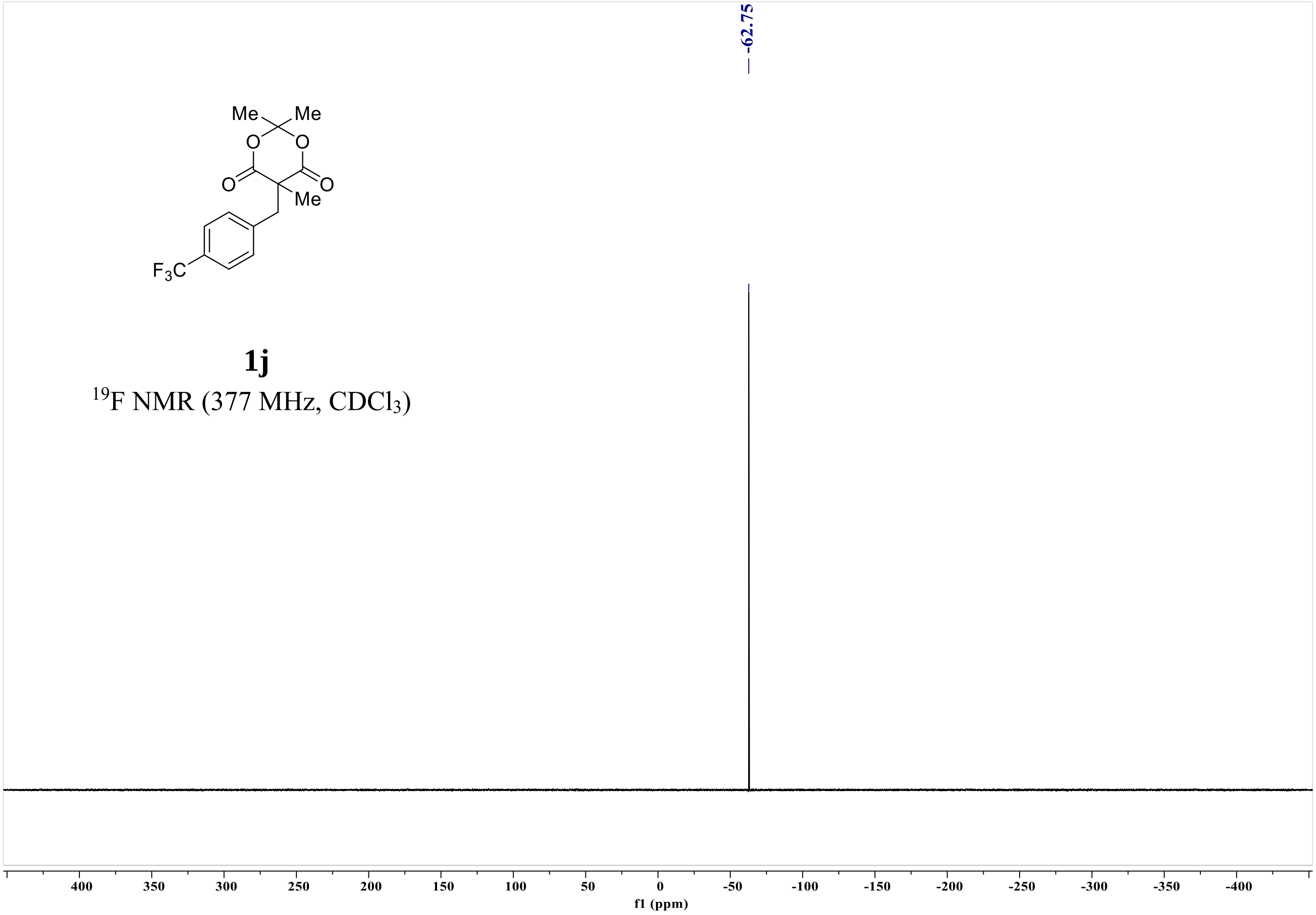

1j

^{19}F NMR (377 MHz, $CDCl_3$)

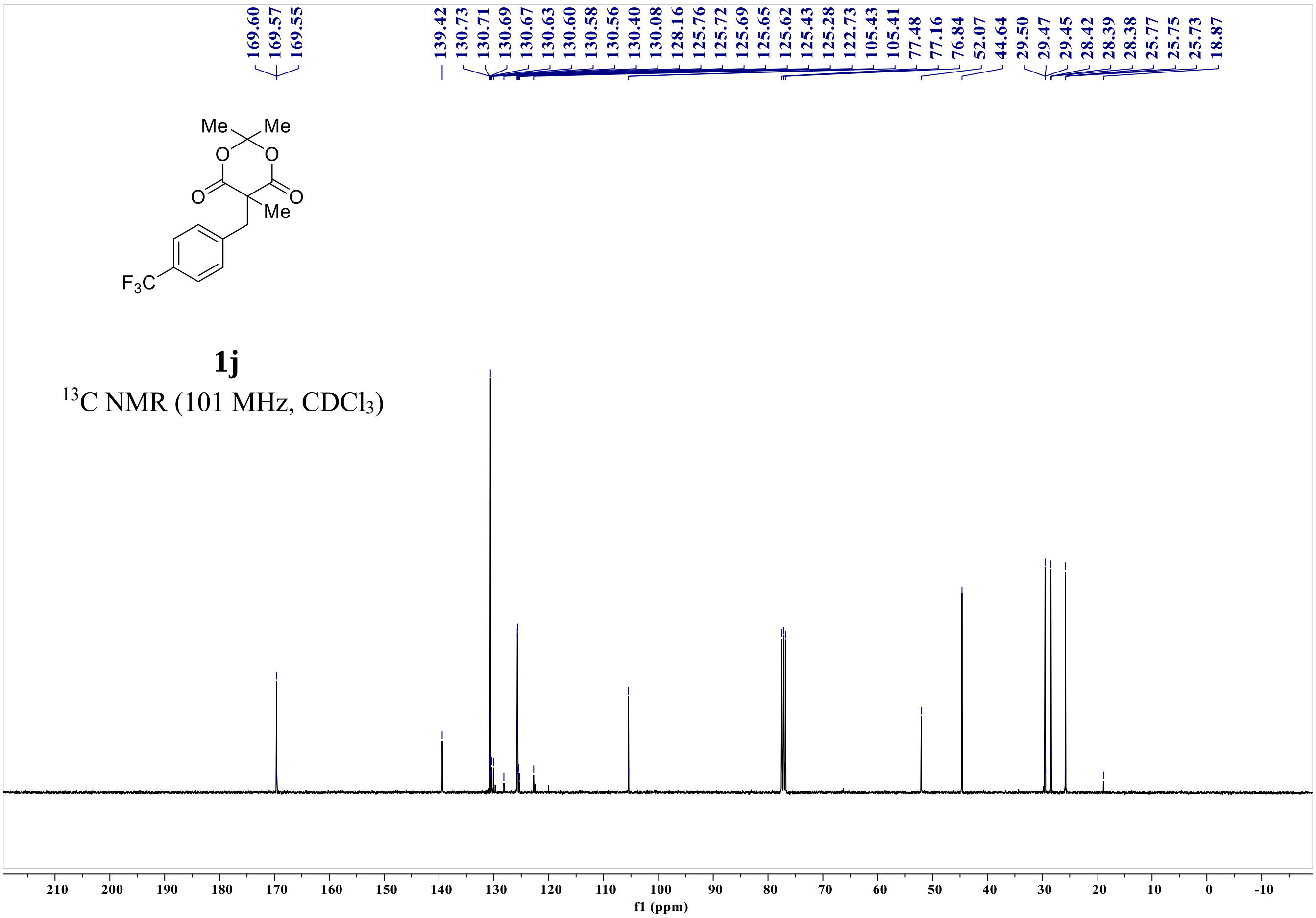

1j

^{13}C NMR (101 MHz, $CDCl_3$)

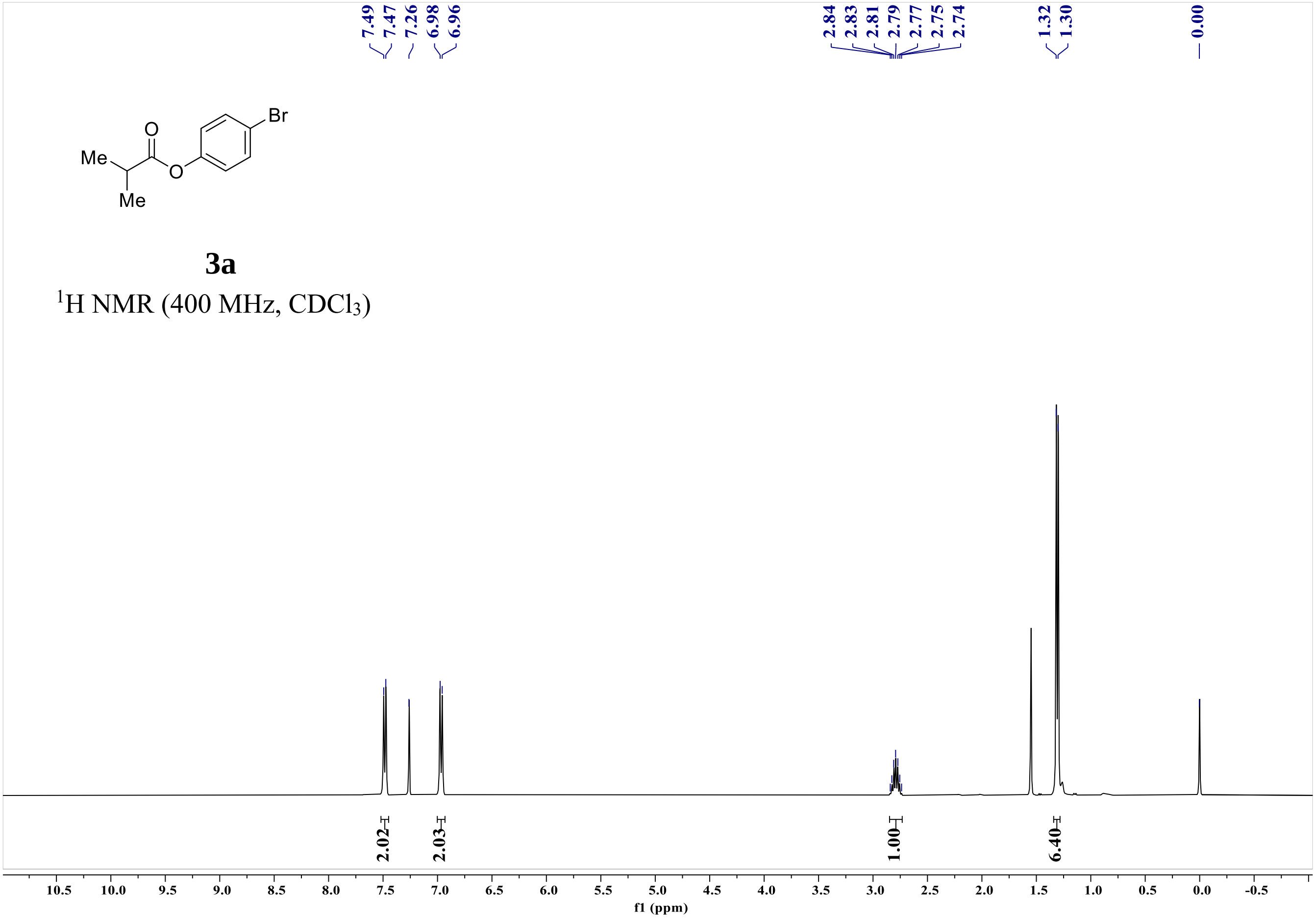
7.49
7.47
7.26
6.98
6.96
2.84
2.83
2.81
2.79
2.77
2.75
2.74
1.32
1.30
0.00
Br
O
Me
O
Me
3a
¹H NMR (400 MHz, CDCl₃)
2.02
2.03
1.00
6.40
10.5 10.0 9.5 9.0 8.5 8.0 7.5 7.0 6.5 6.0 5.5 5.0 4.5 4.0 3.5 3.0 2.5 2.0 1.5 1.0 0.5 0.0 -0.5
f1 (ppm)

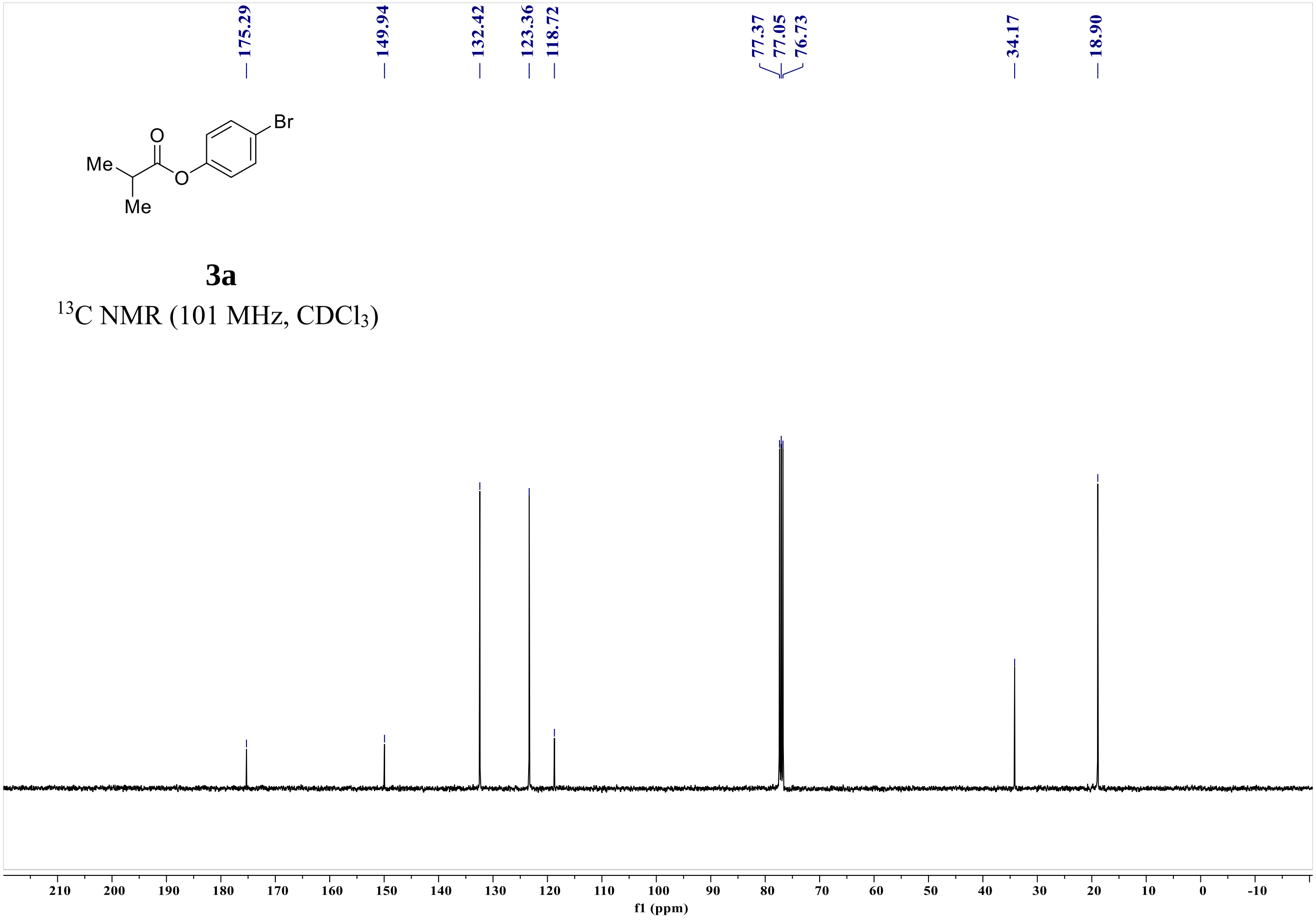
175.29
149.94
132.42
123.36
118.72
77.37
77.05
76.73
34.17
18.90
Br
O
Me
O
Me
3a
¹³C NMR (101 MHz, CDCl₃)
210 200 190 180 170 160 150 140 130 120 110 100 90 80 70 60 50 40 30 20 10 0 -10
f1 (ppm)

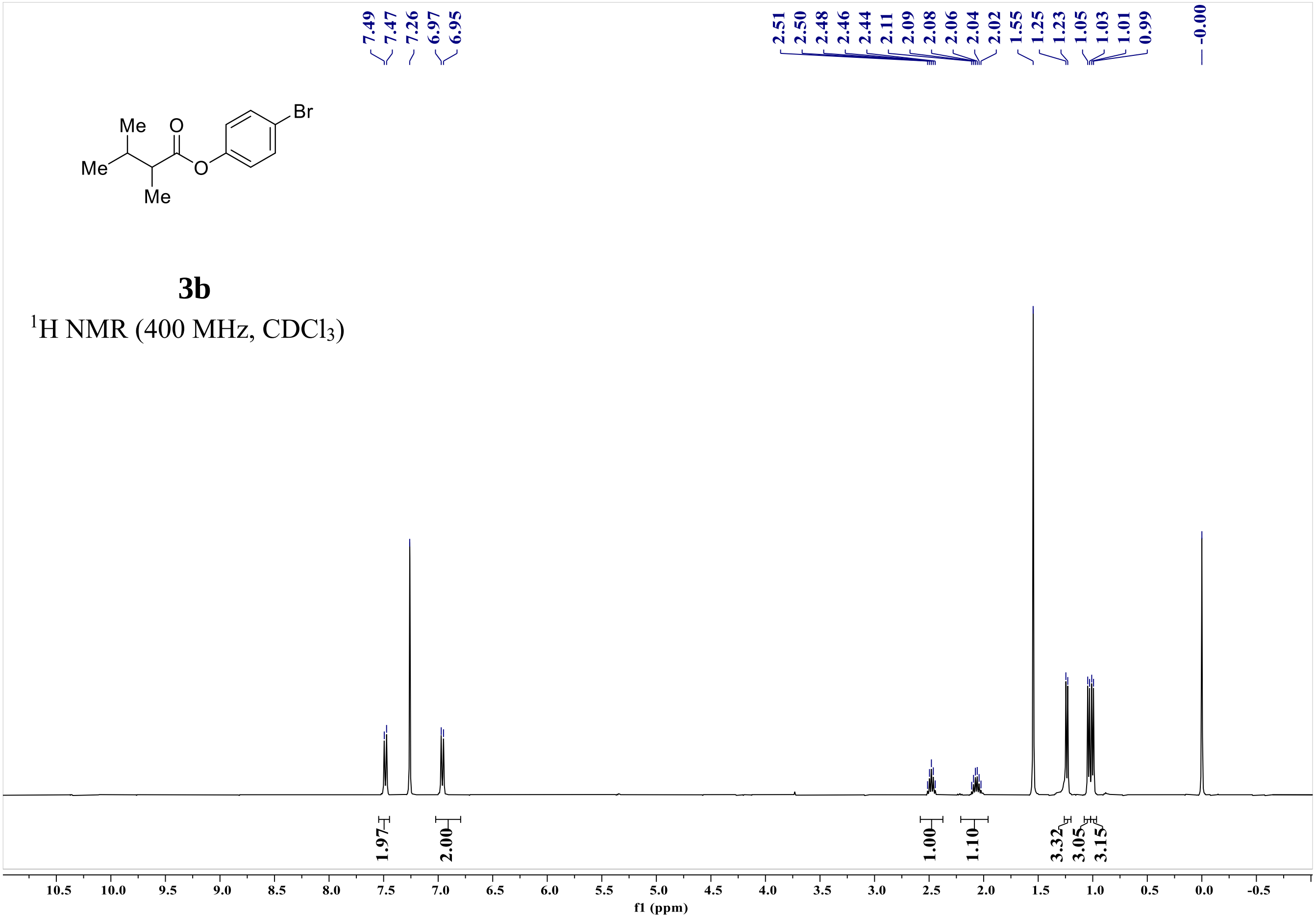
7.49
7.47
7.26
6.97
6.95
2.51
2.50
2.48
2.46
2.44
2.11
2.09
2.08
2.06
2.04
2.02
1.55
1.25
1.23
1.05
1.03
1.01
0.99
-0.00
Me
O
Br
Me
O
Me
3b
^{1}H NMR (400 MHz, $CDCl_3$)
1.97
2.00
1.00
1.10
3.32
3.05
3.15
f1 (ppm)

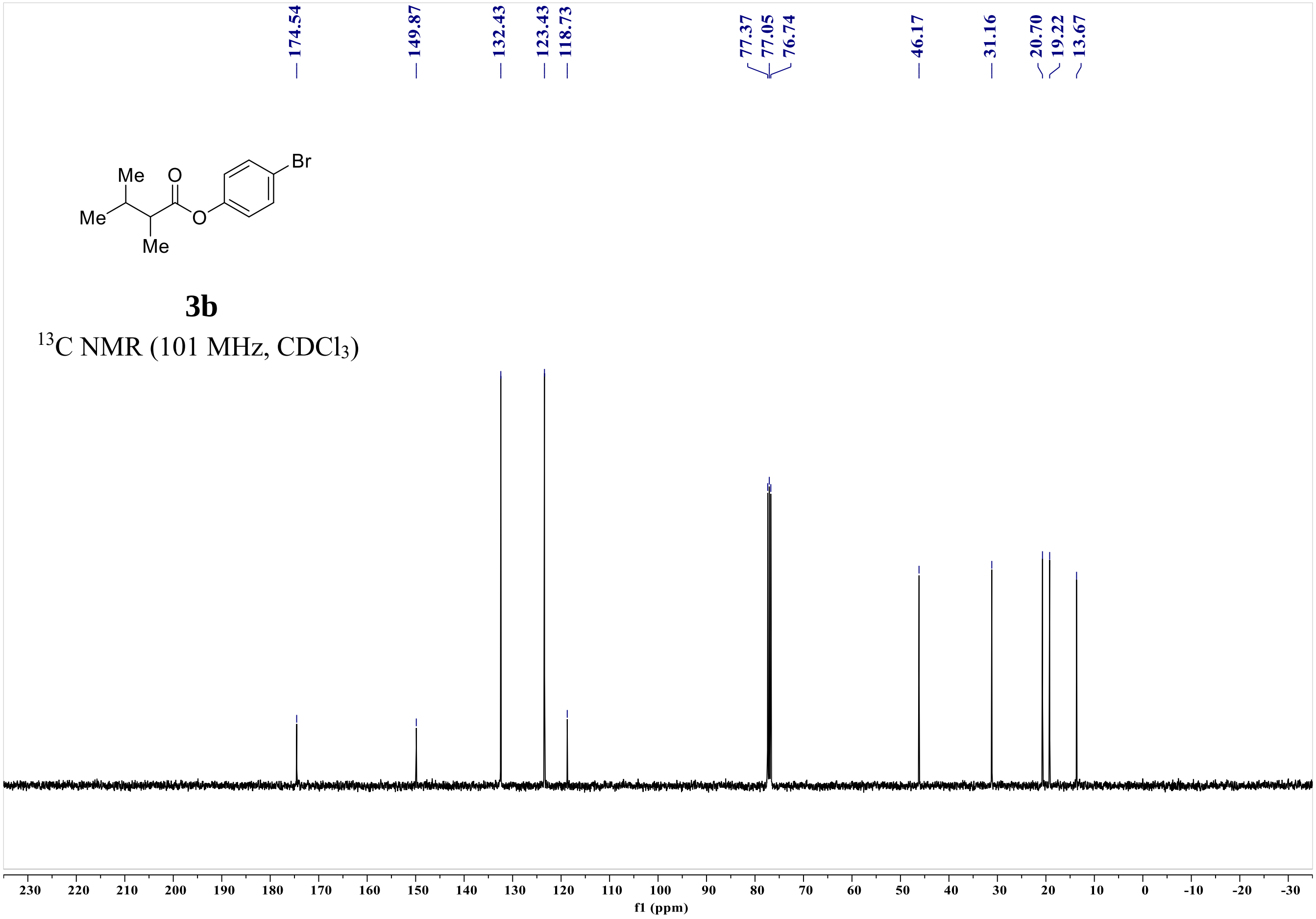
174.54
149.87
132.43
123.43
118.73
77.37
77.05
76.74
46.17
31.16
20.70
19.22
13.67
Me
O
Br
Me
O
Me
3b
^{13}C NMR (101 MHz, $CDCl_3$)
f1 (ppm)

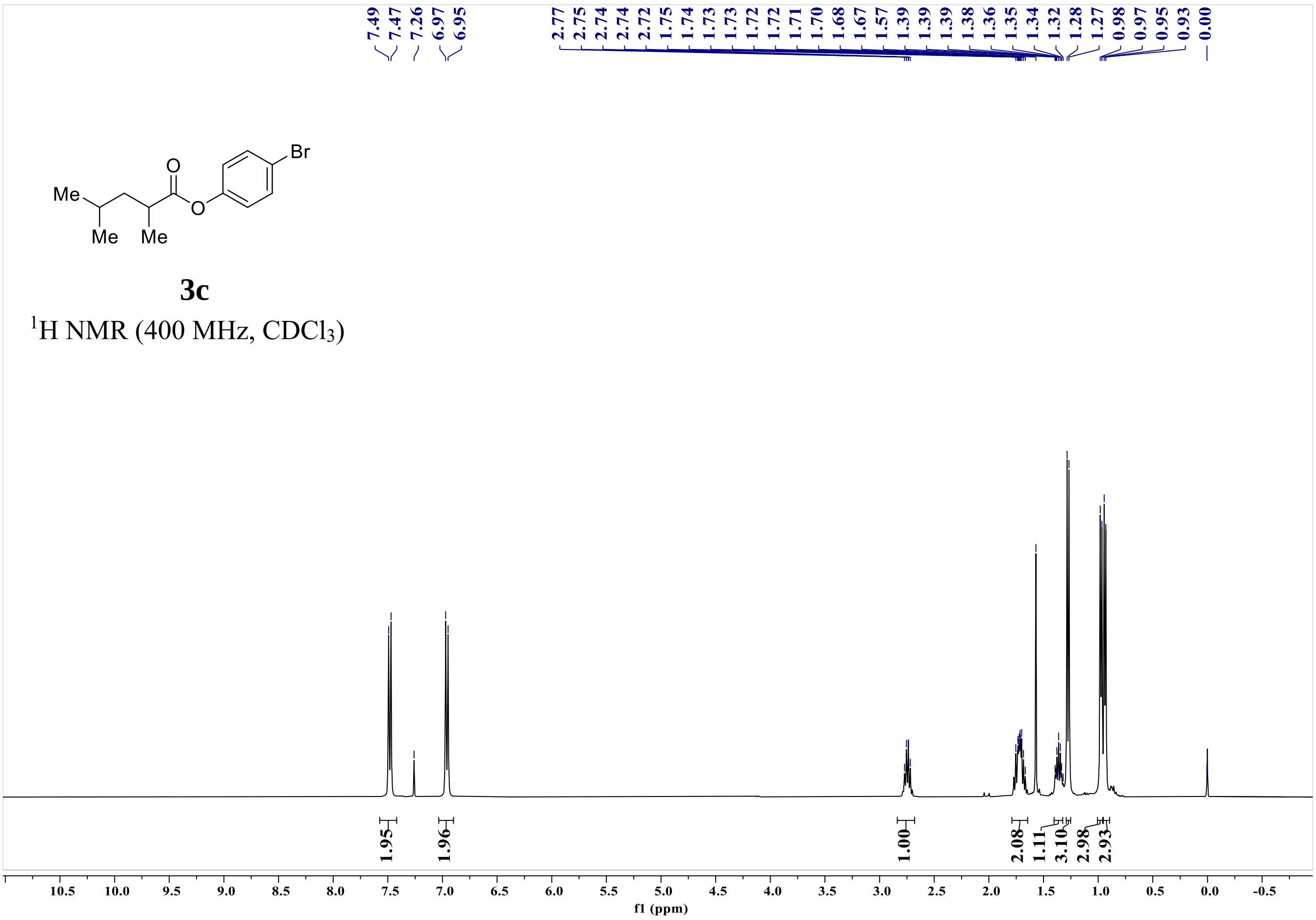
7.49
7.47
7.26
6.97
6.95
2.77
2.75
2.74
2.74
2.72
1.75
1.74
1.73
1.73
1.72
1.72
1.71
1.70
1.68
1.67
1.57
1.39
1.39
1.39
1.38
1.36
1.35
1.34
1.32
1.28
1.27
0.98
0.97
0.95
0.93
0.00
Br
O
Me
O
Me
Me
3c
^{1}H NMR (400 MHz, CDCl$_3$)
1.95
1.96
1.00
2.08
1.11
3.10
2.98
2.93
f1 (ppm)

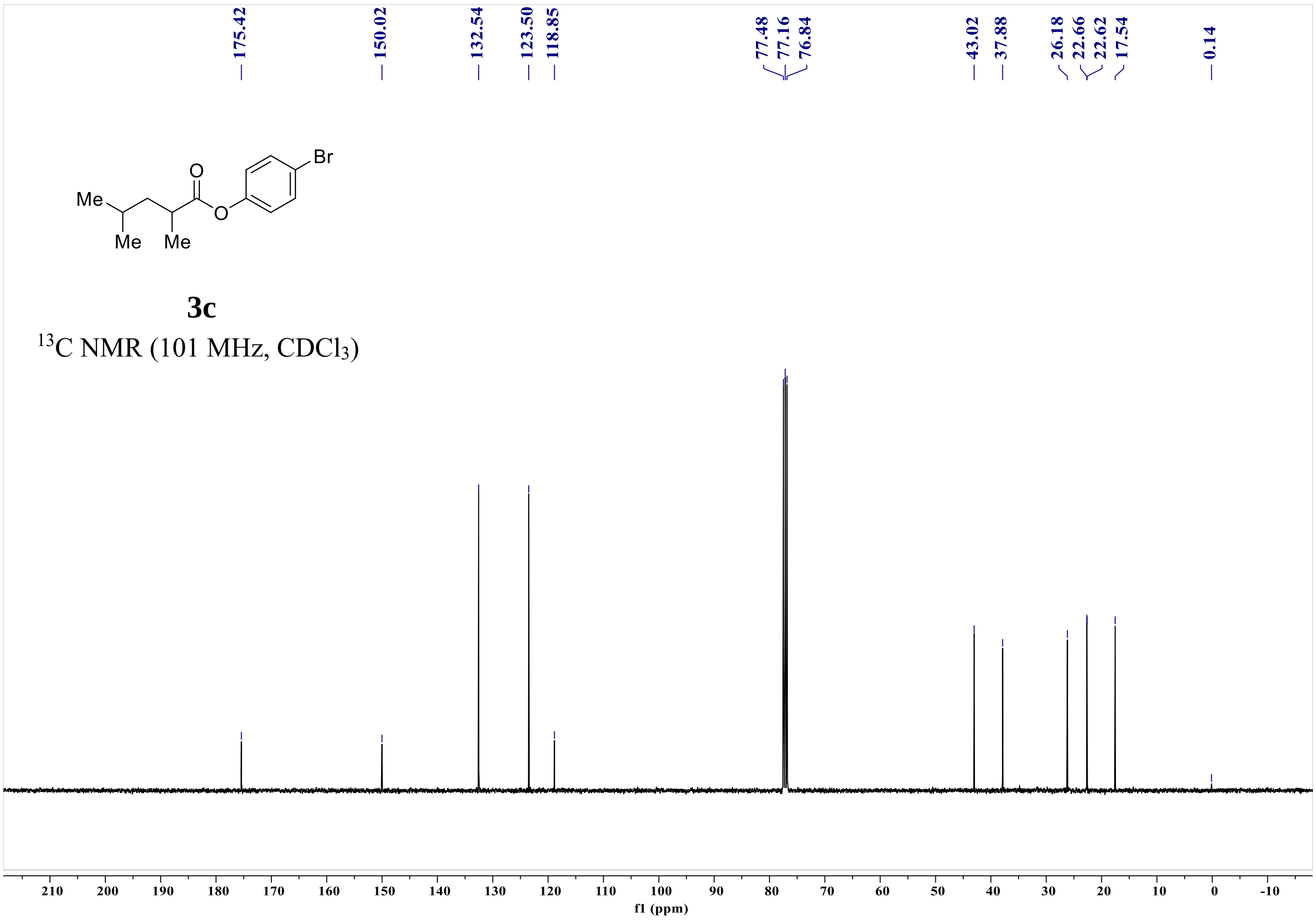
175.42
150.02
132.54
123.50
118.85
77.48
77.16
76.84
43.02
37.88
26.18
22.66
22.62
17.54
0.14
Br
O
Me
O
Me
Me
3c
^{13}C NMR (101 MHz, CDCl$_3$)
f1 (ppm)

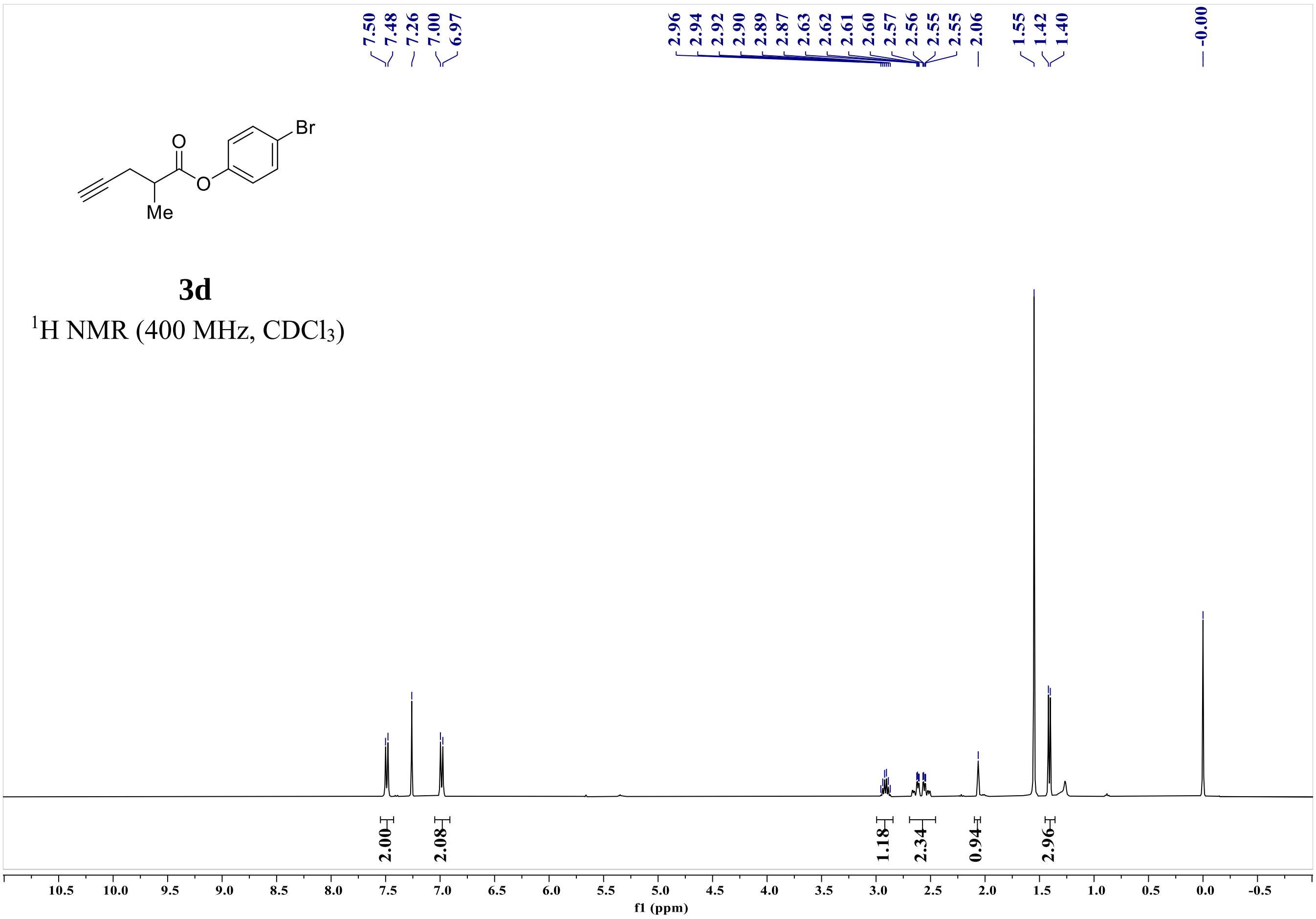

3d

^{1}H NMR (400 MHz, $CDCl_3$)

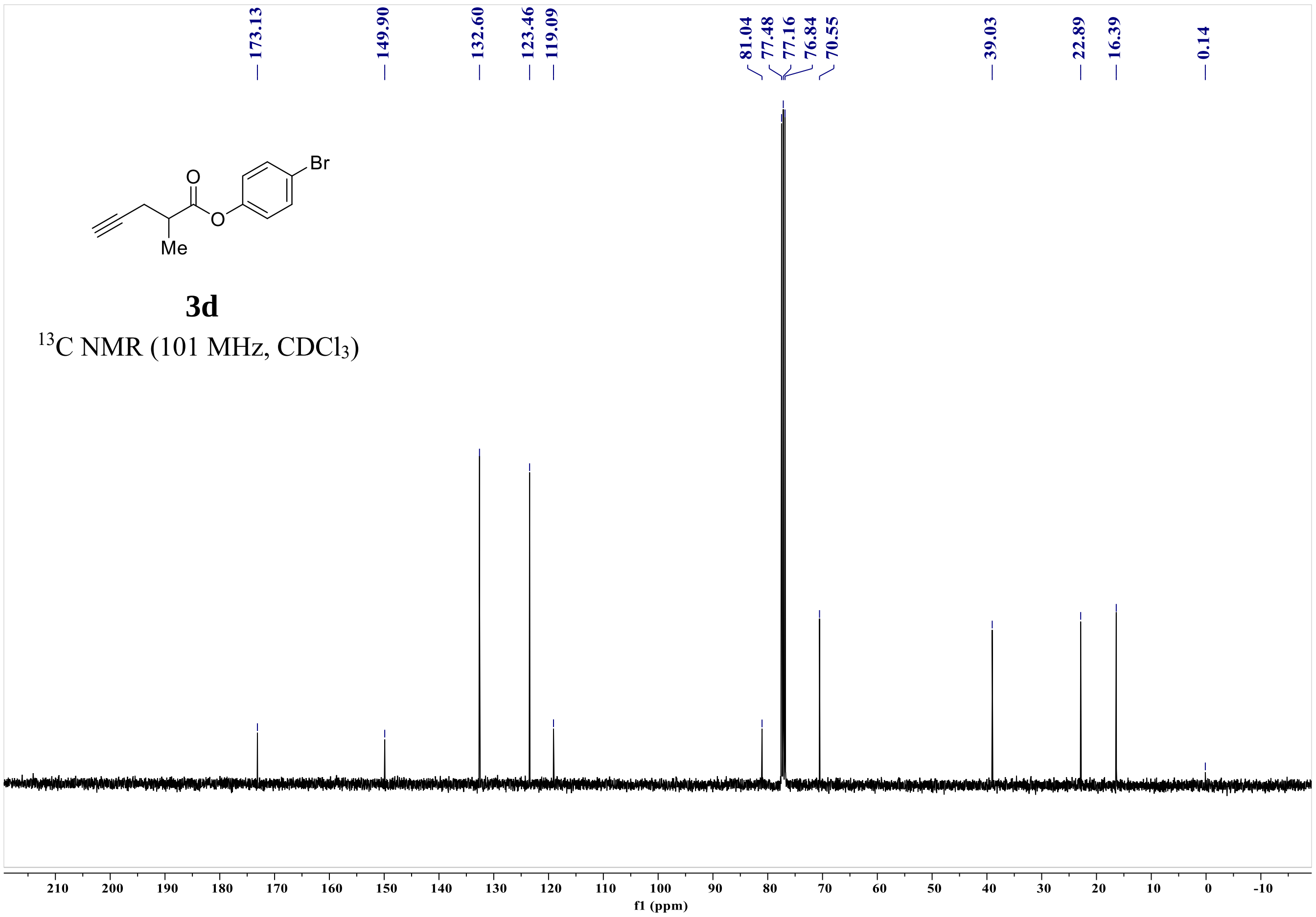

3d

^{13}C NMR (101 MHz, $CDCl_3$)

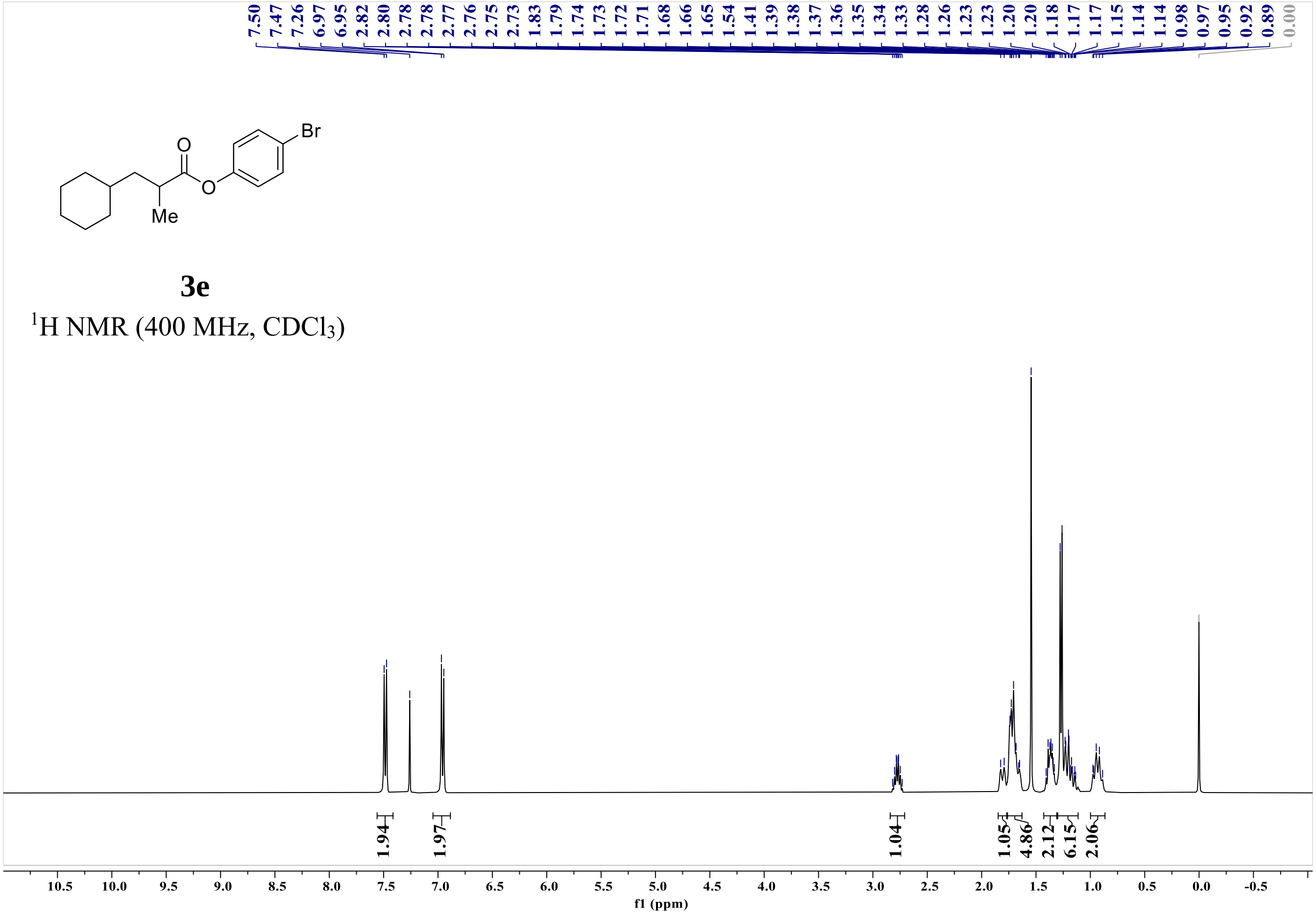
7.50 7.47 7.26 6.97 6.95 2.82 2.80 2.78 2.78 2.77 2.76 2.75 2.73 1.83 1.79 1.74 1.73 1.72 1.71 1.68 1.66 1.65 1.54 1.41 1.39 1.38 1.37 1.36 1.35 1.34 1.33 1.28 1.26 1.23 1.23 1.20 1.20 1.18 1.17 1.17 1.15 1.14 1.14 0.98 0.97 0.95 0.92 0.89 0.00
Br
O
O
Me
3e
^{1}H NMR (400 MHz, $CDCl_3$)
1.94
1.97
1.04
1.05
4.86
2.12
6.15
2.06
10.5 10.0 9.5 9.0 8.5 8.0 7.5 7.0 6.5 6.0 5.5 5.0 4.5 4.0 3.5 3.0 2.5 2.0 1.5 1.0 0.5 0.0 -0.5
f1 (ppm)

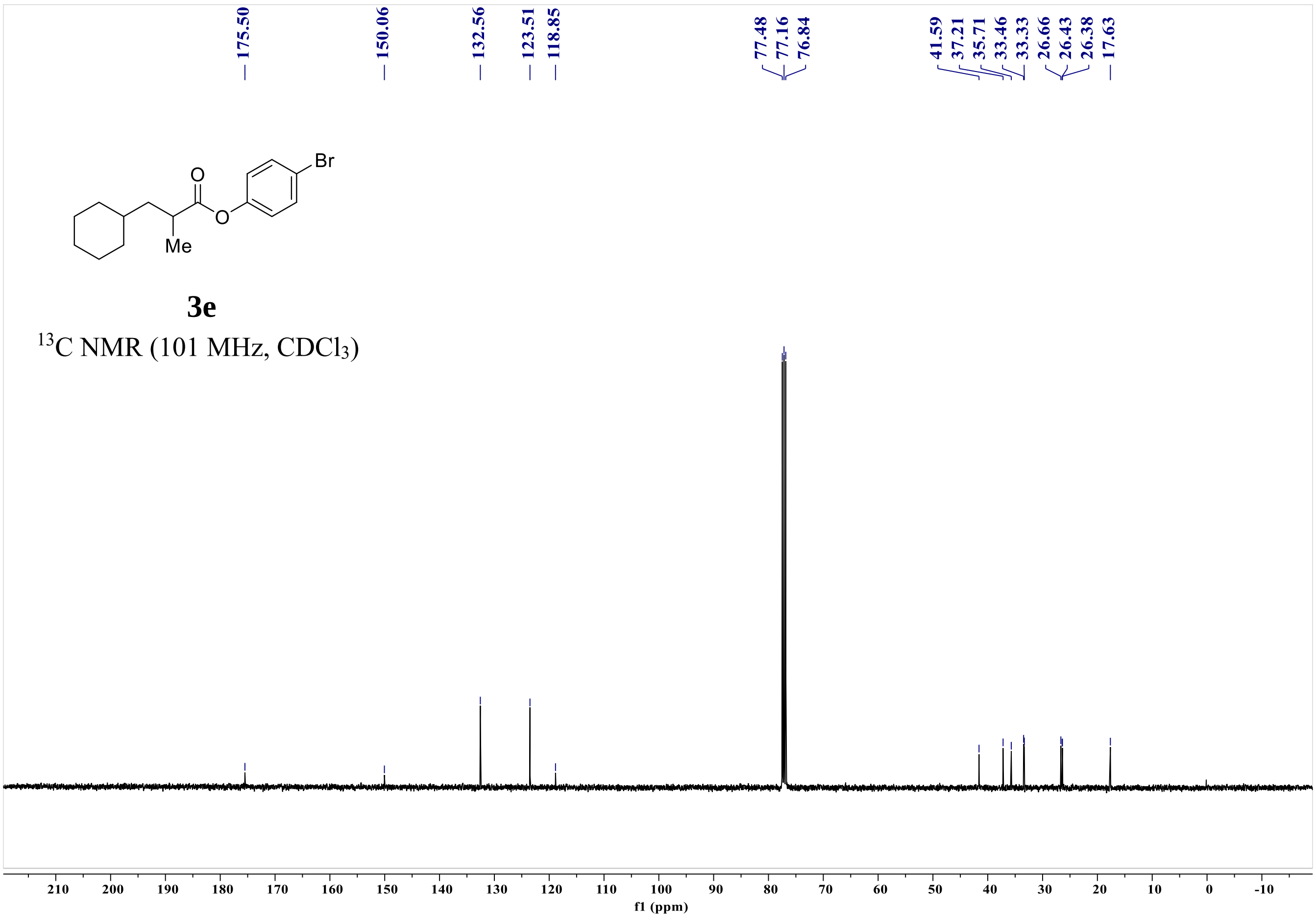
175.50
150.06
132.56
123.51
118.85
77.48 77.16 76.84
41.59 37.21 35.71 33.46 33.33 26.66 26.43 26.38 17.63
Br
O
O
Me
3e
^{13}C NMR (101 MHz, $CDCl_3$)
210 200 190 180 170 160 150 140 130 120 110 100 90 80 70 60 50 40 30 20 10 0 -10
f1 (ppm)

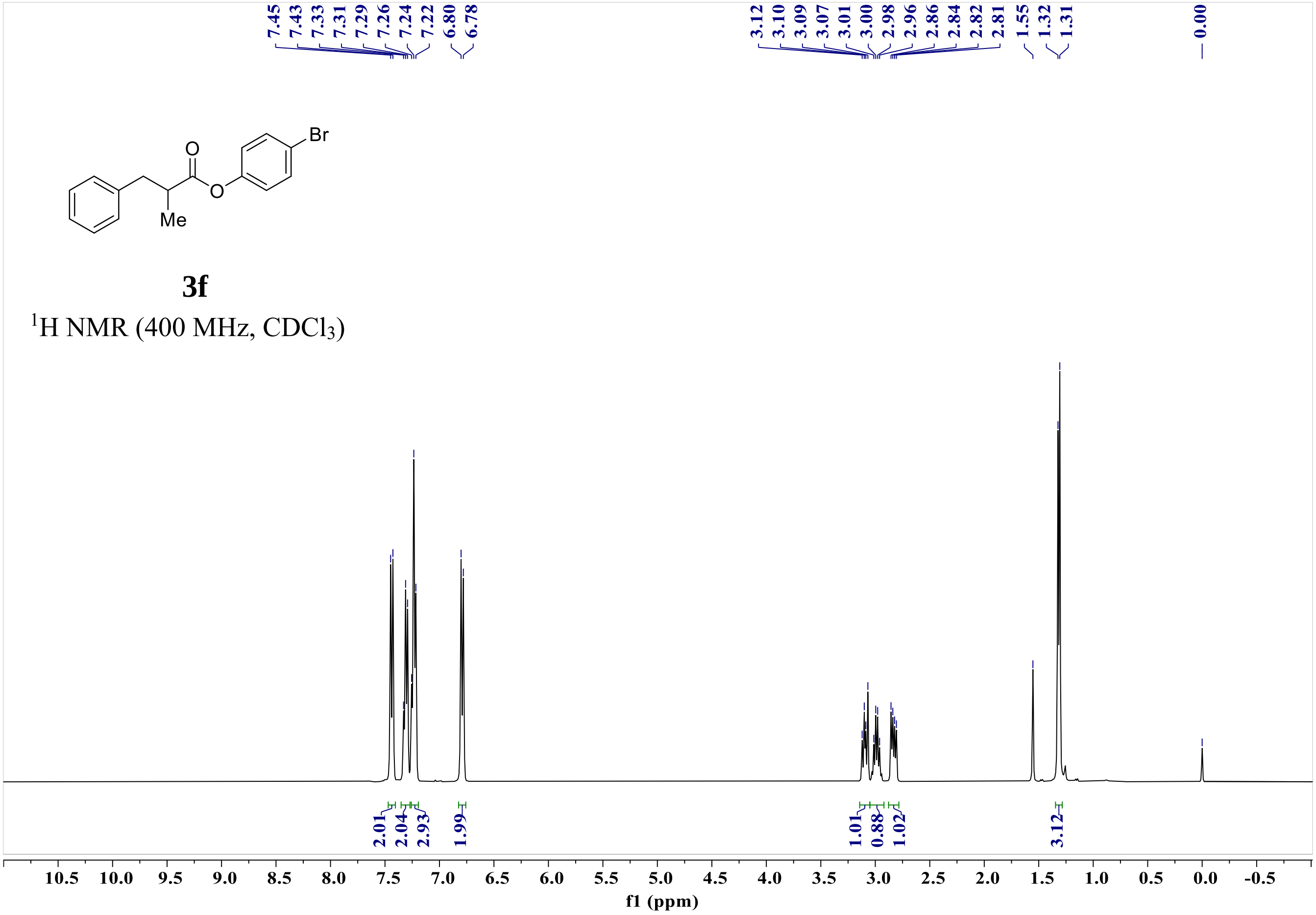
7.45 7.43 7.33 7.31 7.29 7.26 7.24 7.22 6.80 6.78
3.12 3.10 3.09 3.07 3.01 3.00 2.98 2.96 2.86 2.84 2.82 2.81 1.55 1.32 1.31
0.00
Br
O
O
Me
3f
1H NMR (400 MHz, CDCl3)
2.01 2.04 2.93 1.99
1.01 0.88 1.02
3.12
10.5 10.0 9.5 9.0 8.5 8.0 7.5 7.0 6.5 6.0 5.5 5.0 4.5 4.0 3.5 3.0 2.5 2.0 1.5 1.0 0.5 0.0 -0.5
f1 (ppm)

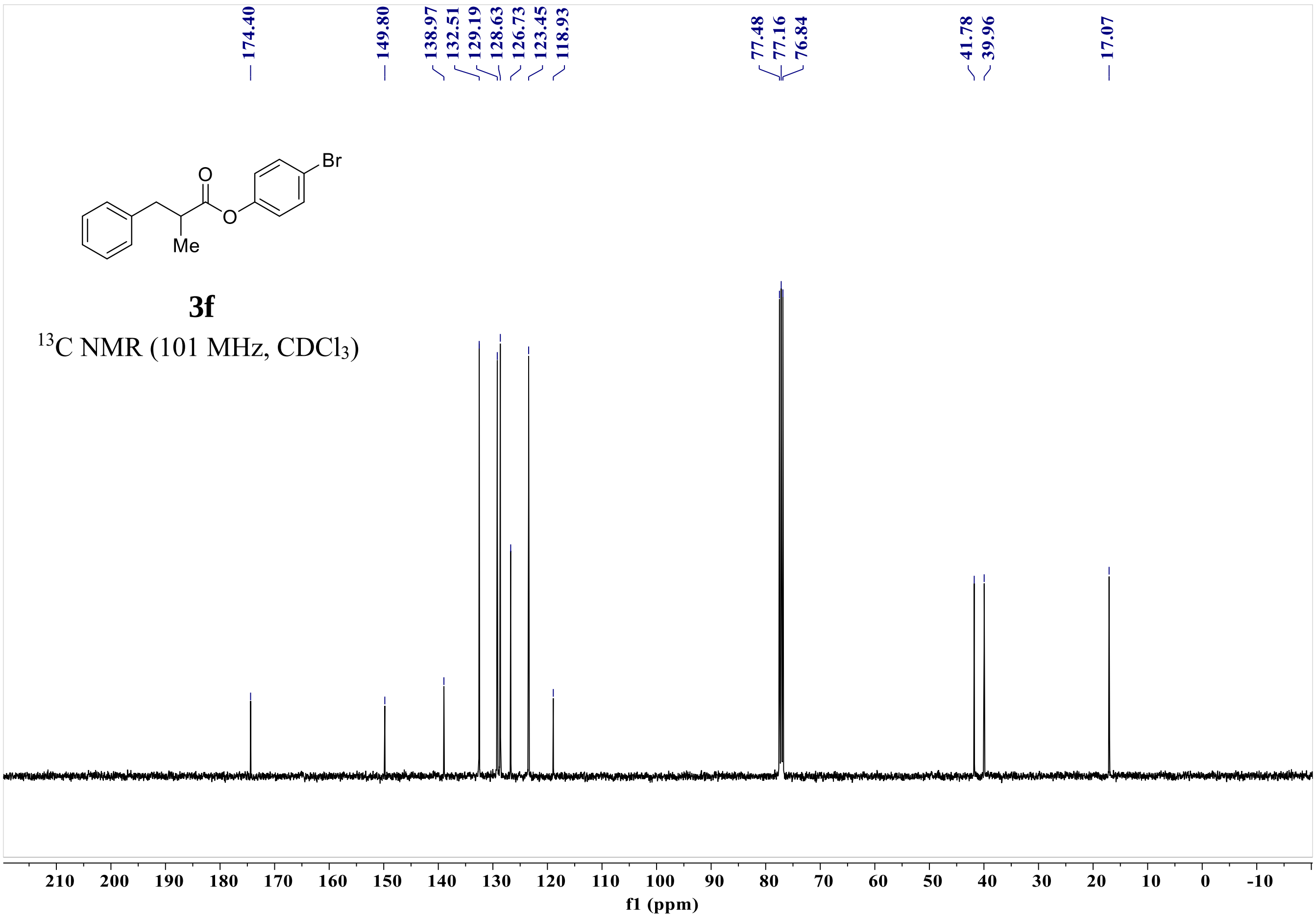
174.40
149.80
138.97 132.51 129.19 128.63 126.73 123.45 118.93
77.48 77.16 76.84
41.78 39.96
17.07
Br
O
O
Me
3f
13C NMR (101 MHz, CDCl3)
210 200 190 180 170 160 150 140 130 120 110 100 90 80 70 60 50 40 30 20 10 0 -10
f1 (ppm)

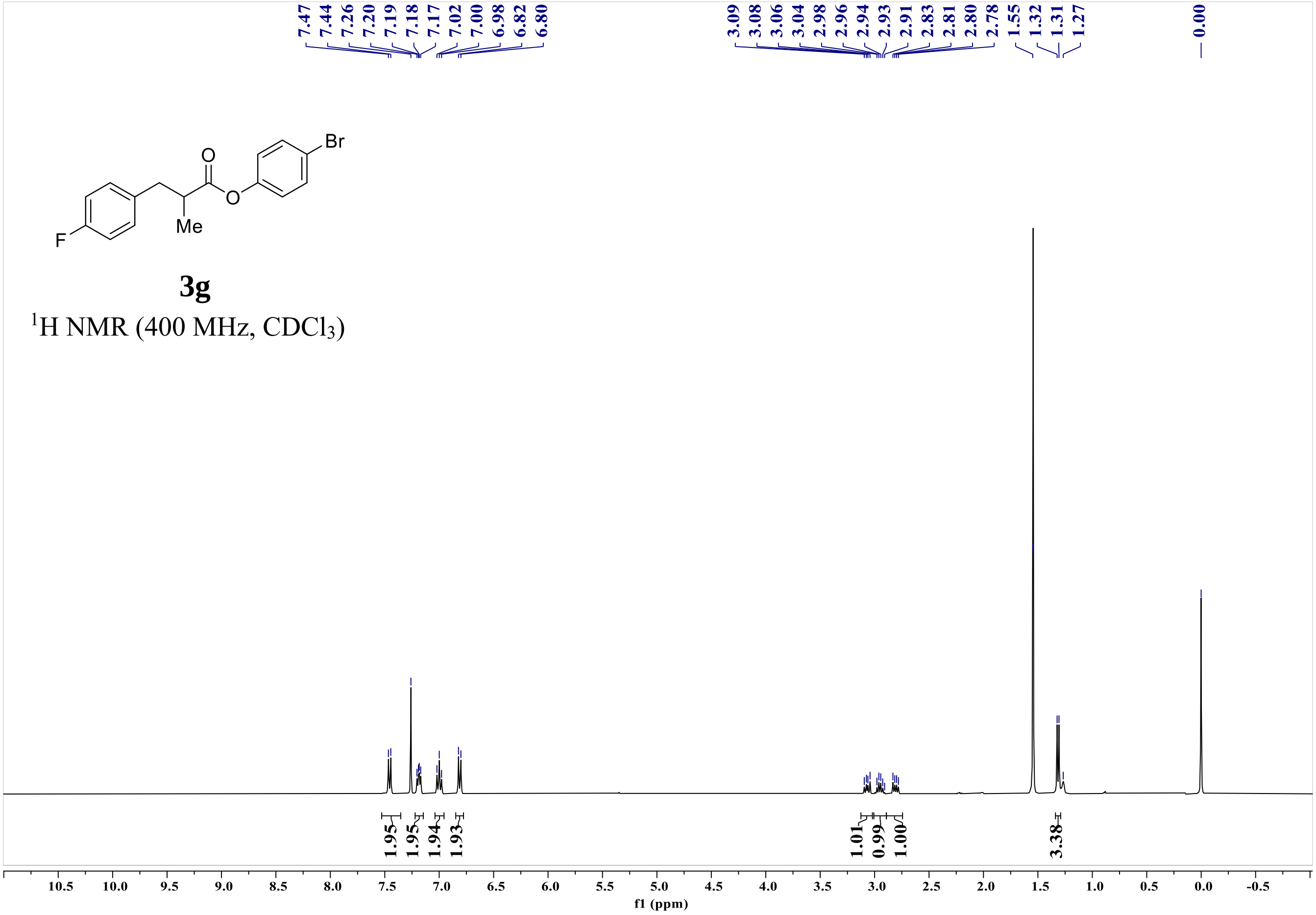

7.47 7.44 7.26 7.20 7.19 7.18 7.17 7.02 7.00 6.98 6.82 6.80
3.09 3.08 3.06 3.04 2.98 2.96 2.94 2.93 2.91 2.83 2.81 2.80 2.78 1.55 1.32 1.31 1.27
0.00
Br
O
O
Me
F
3g
1H NMR (400 MHz, CDCl3)
1.95 1.95 1.94 1.93
1.01 0.99 1.00
3.38
10.5 10.0 9.5 9.0 8.5 8.0 7.5 7.0 6.5 6.0 5.5 5.0 4.5 4.0 3.5 3.0 2.5 2.0 1.5 1.0 0.5 0.0 -0.5
f1 (ppm)

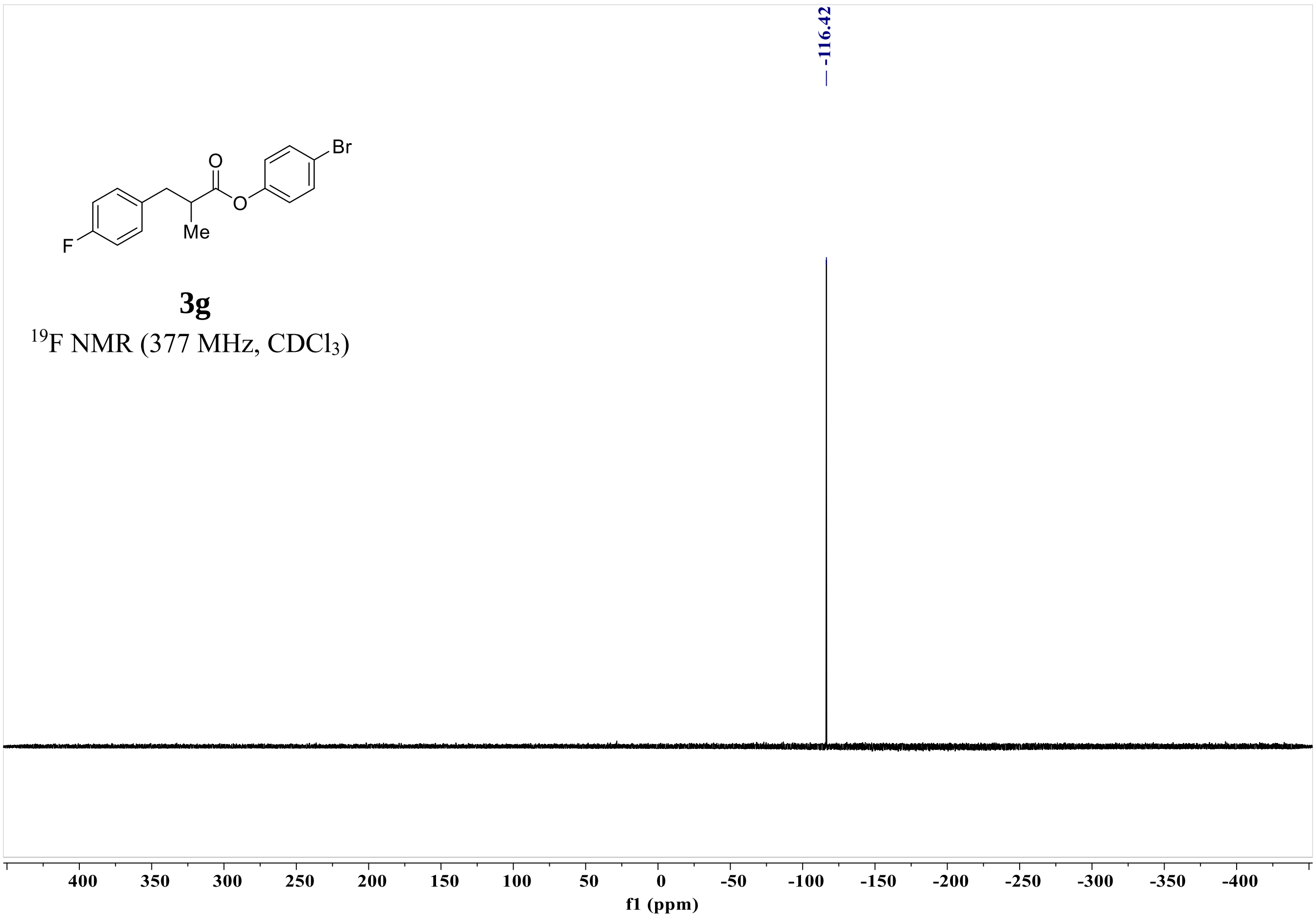

-116.42
Br
O
O
Me
F
3g
19F NMR (377 MHz, CDCl3)
400 350 300 250 200 150 100 50 0 -50 -100 -150 -200 -250 -300 -350 -400
f1 (ppm)

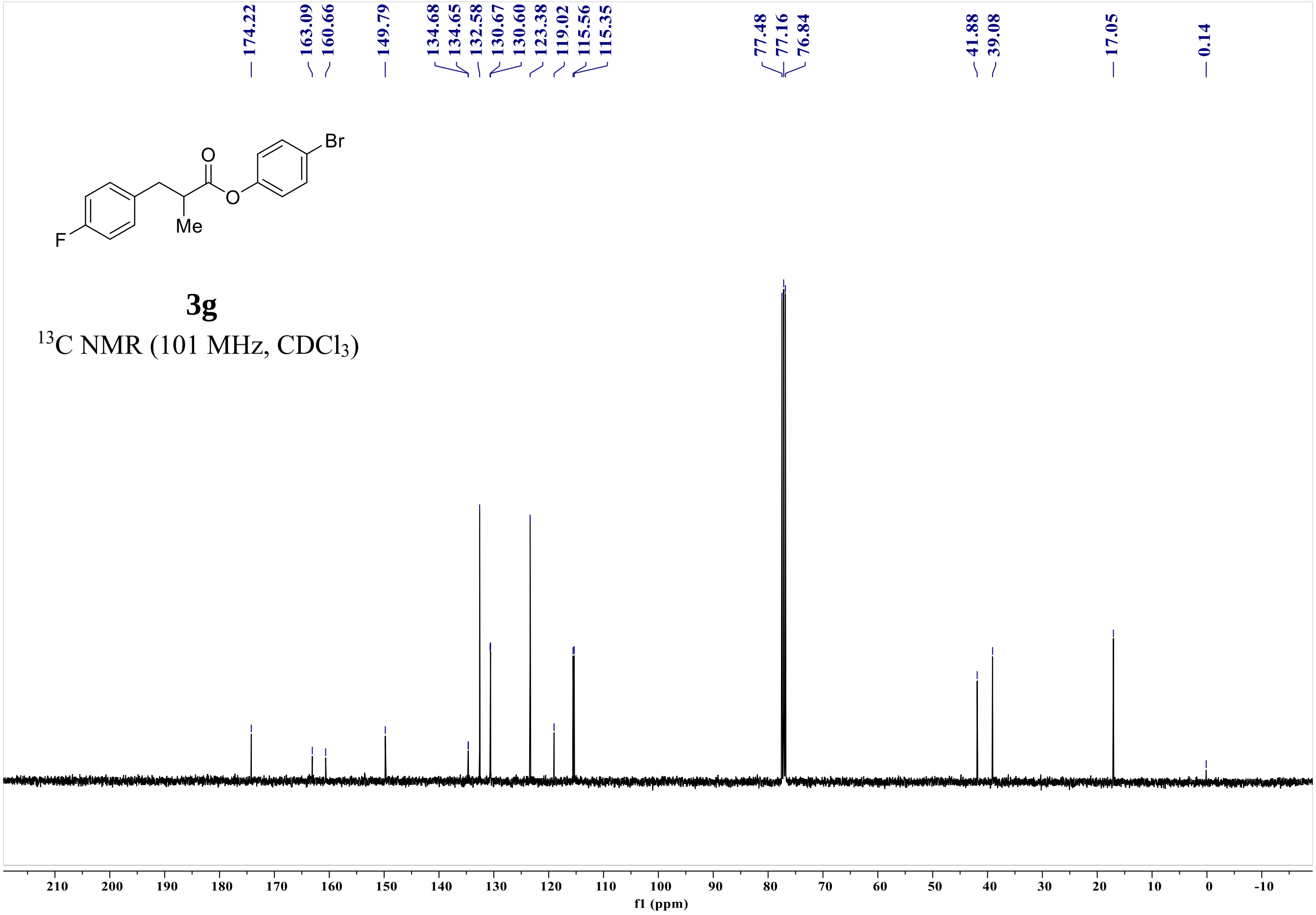
174.22
163.09
160.66
149.79
134.68
134.65
132.58
130.67
130.60
123.38
119.02
115.56
115.35
77.48
77.16
76.84
41.88
39.08
17.05
0.14
Br
O
O
Me
F
3g
^{13}C NMR (101 MHz, CDCl$_3$)
210 200 190 180 170 160 150 140 130 120 110 100 90 80 70 60 50 40 30 20 10 0 -10
f1 (ppm)

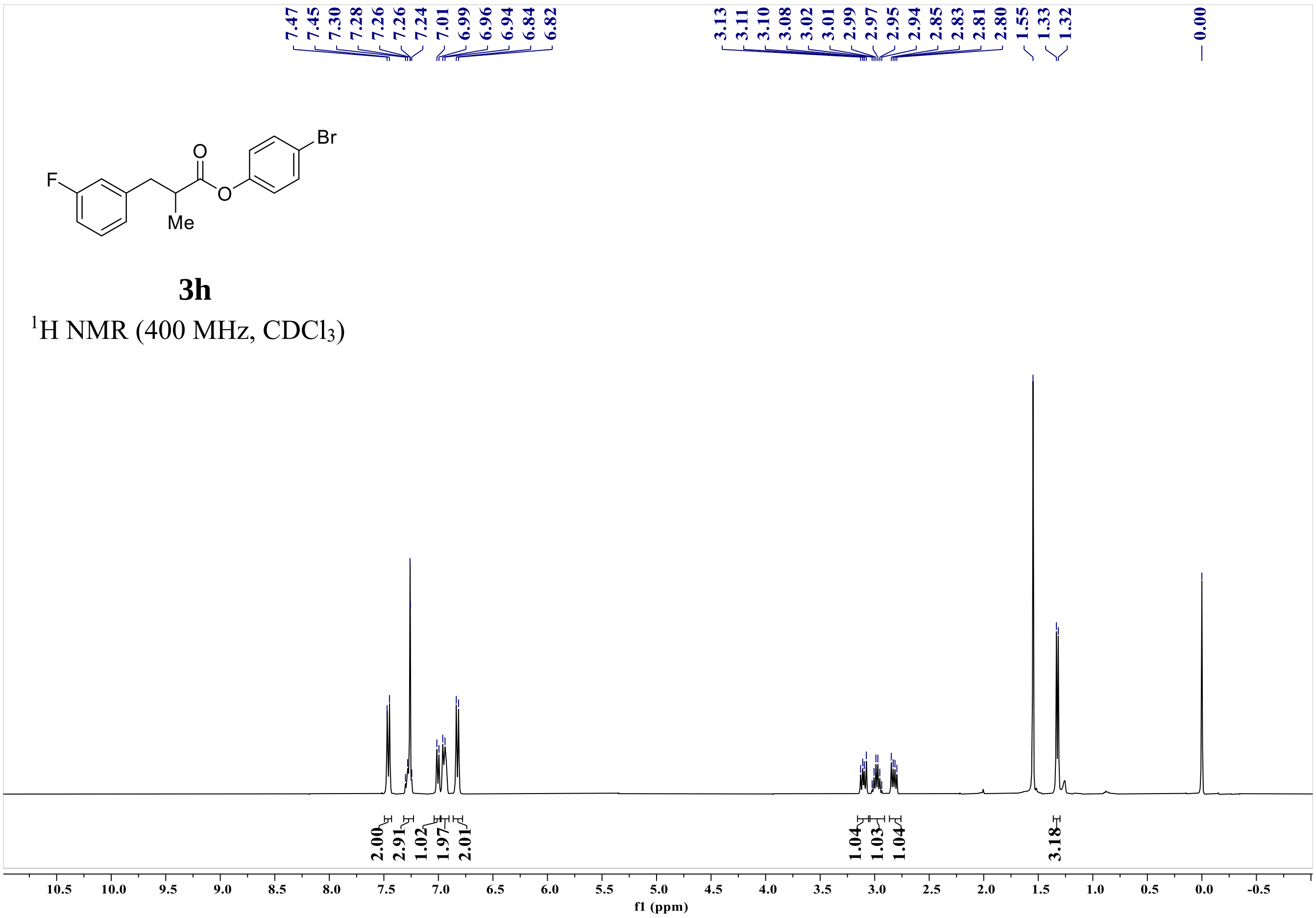
7.47
7.45
7.30
7.28
7.26
7.26
7.24
7.01
6.99
6.96
6.94
6.84
6.82
3.13
3.11
3.10
3.08
3.02
3.01
2.99
2.97
2.95
2.94
2.85
2.83
2.81
2.80
1.55
1.33
1.32
0.00
F
O
Br
O
Me
3h
^{1}H NMR (400 MHz, CDCl$_3$)
2.00
2.91
1.02
1.97
2.01
1.04
1.03
1.04
3.18
10.5 10.0 9.5 9.0 8.5 8.0 7.5 7.0 6.5 6.0 5.5 5.0 4.5 4.0 3.5 3.0 2.5 2.0 1.5 1.0 0.5 0.0 -0.5
f1 (ppm)

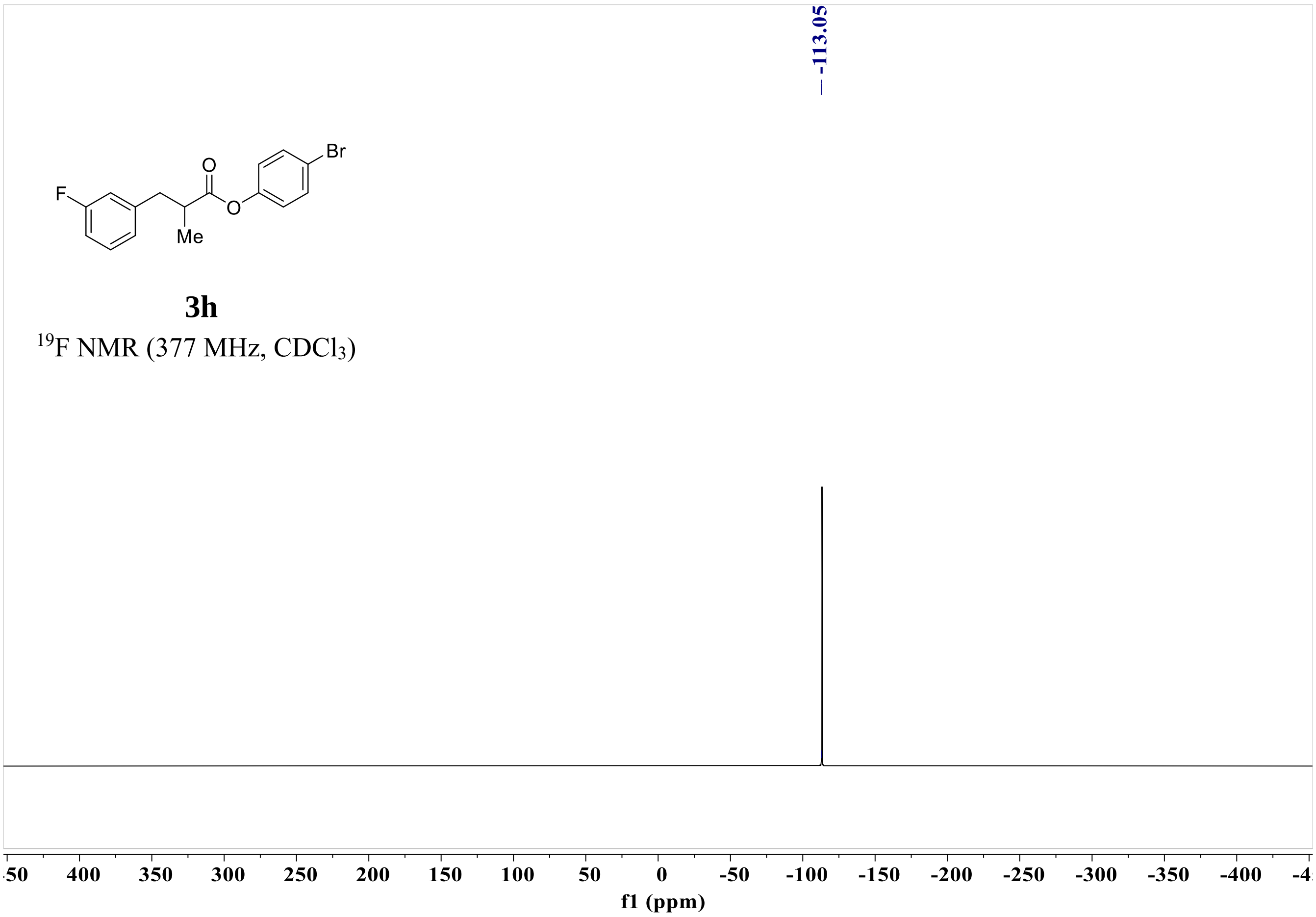

3h

^{19}F NMR (377 MHz, $CDCl_3$)

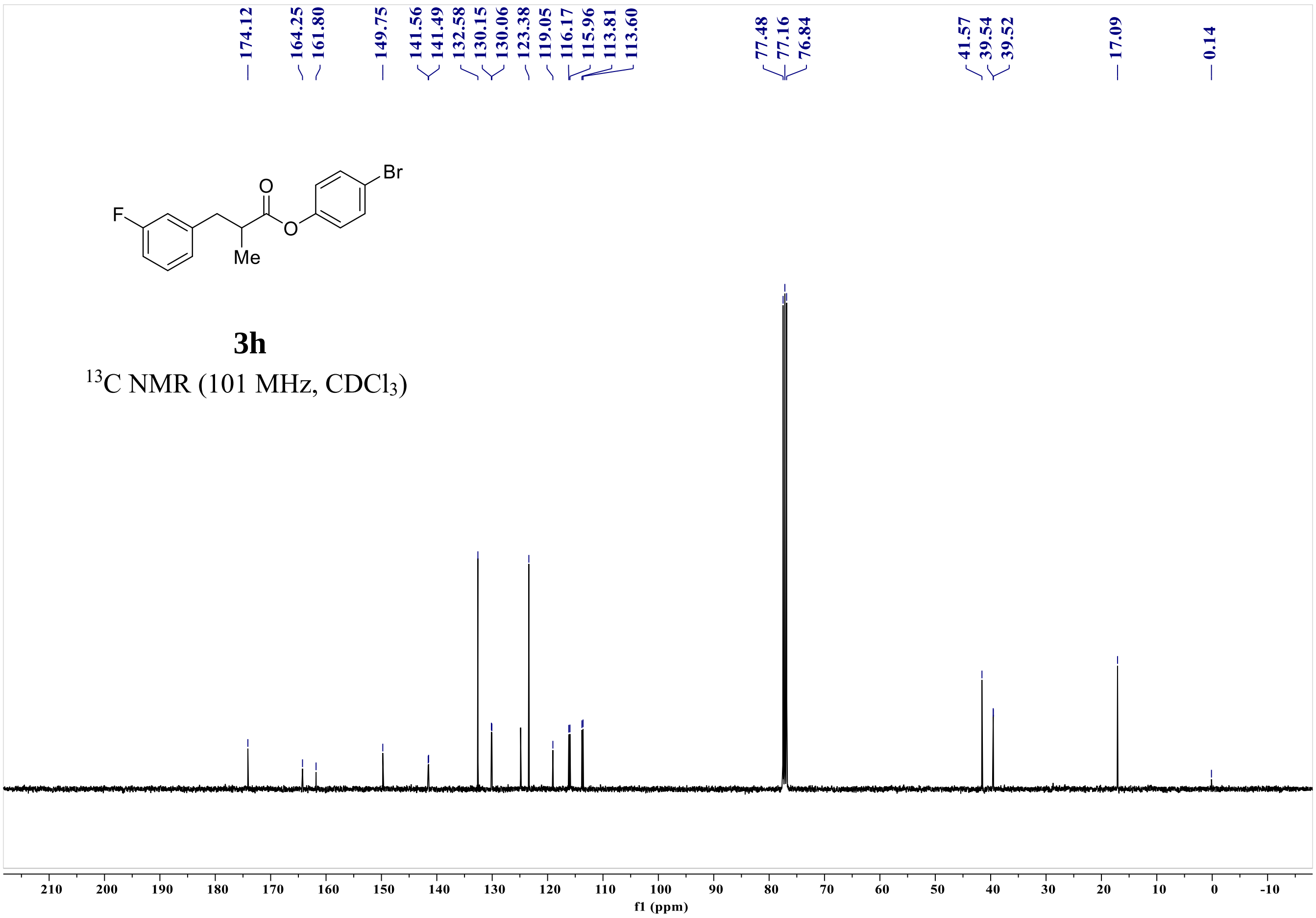

3h

^{13}C NMR (101 MHz, $CDCl_3$)

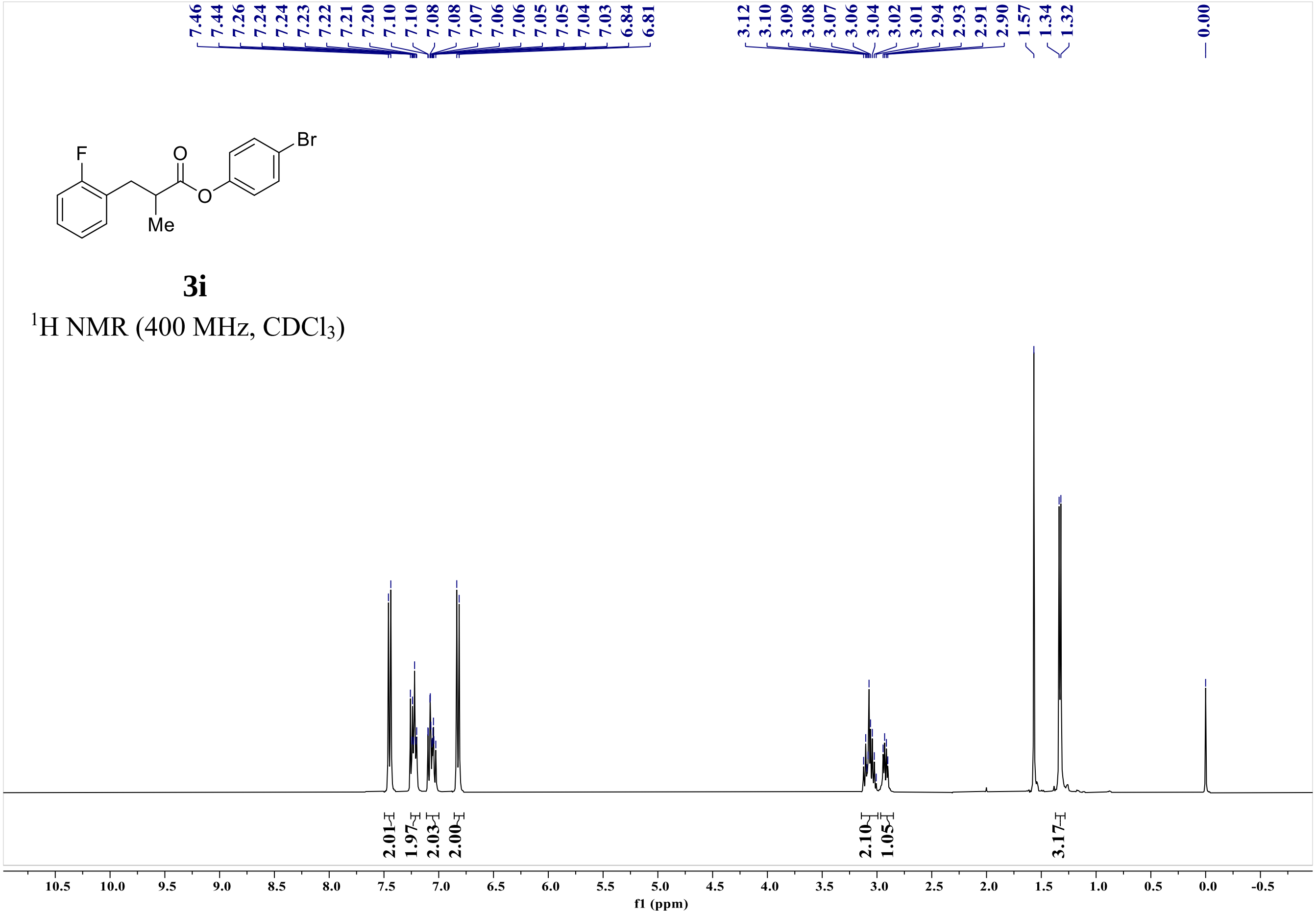
7.46 7.44 7.26 7.24 7.24 7.23 7.22 7.21 7.20 7.10 7.08 7.08 7.07 7.06 7.06 7.05 7.05 7.04 7.03 6.84 6.81
3.12 3.10 3.09 3.08 3.07 3.06 3.04 3.02 3.01 2.94 2.93 2.91 2.90 1.57 1.34 1.32
0.00
F
O
Br
O
Me
3i
^{1}H NMR (400 MHz, CDCl$_3$)
2.01 1.97 2.03 2.00
2.10 1.05
3.17
10.5 10.0 9.5 9.0 8.5 8.0 7.5 7.0 6.5 6.0 5.5 5.0 4.5 4.0 3.5 3.0 2.5 2.0 1.5 1.0 0.5 0.0 -0.5
f1 (ppm)

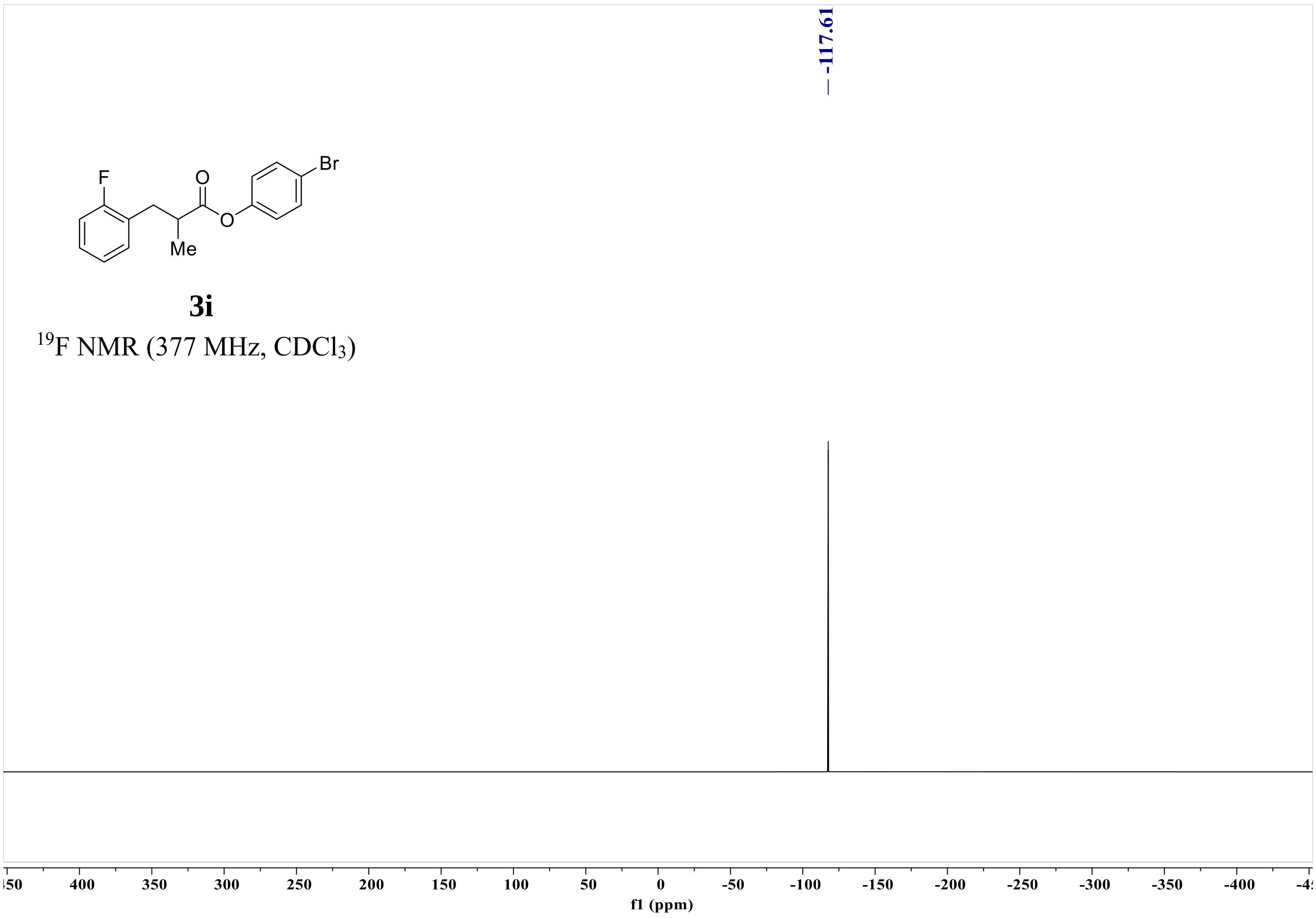
-117.61
F
O
Br
O
Me
3i
^{19}F NMR (377 MHz, CDCl$_3$)
400 350 300 250 200 150 100 50 0 -50 -100 -150 -200 -250 -300 -350 -400
f1 (ppm)

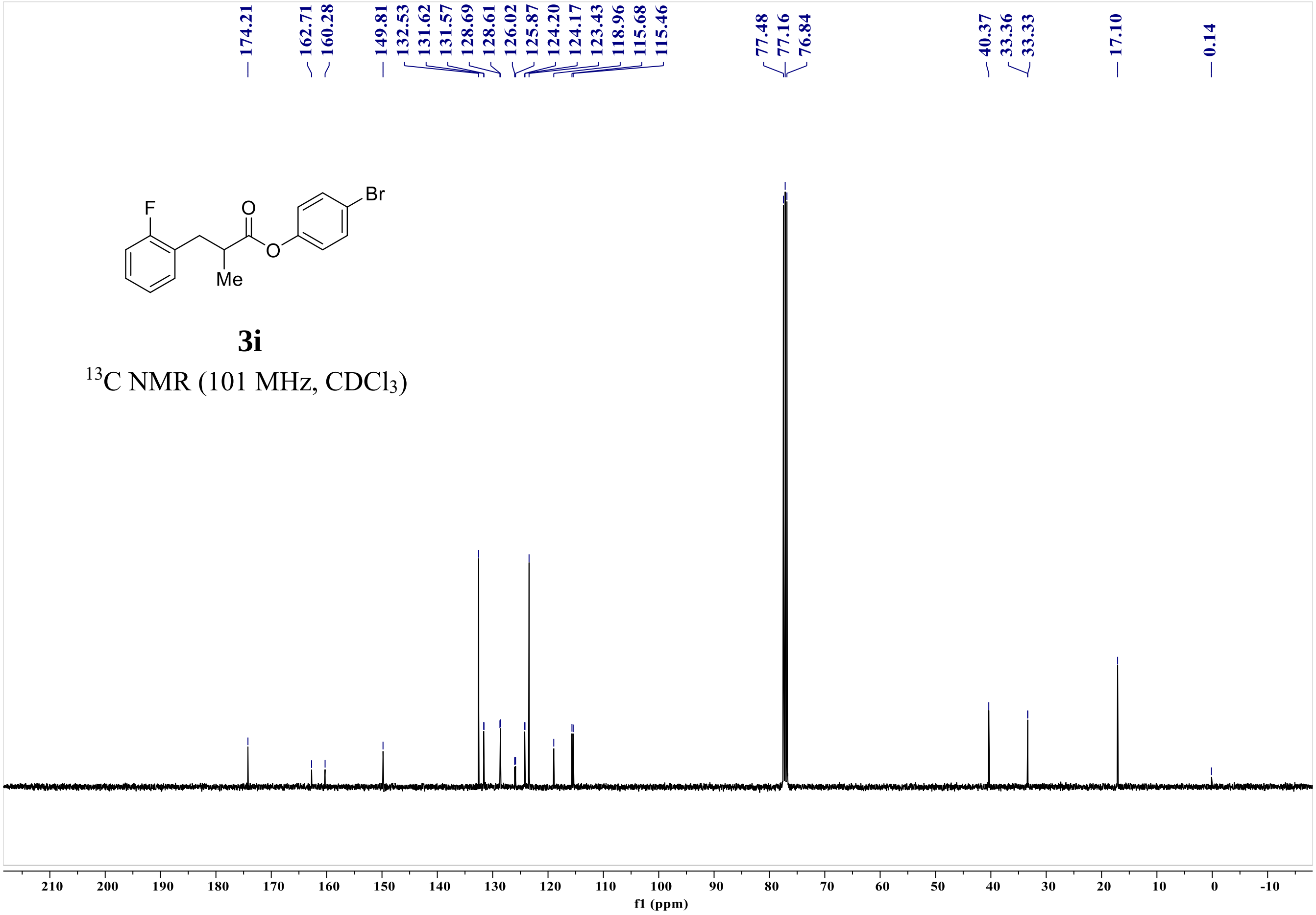
174.21
162.71
160.28
149.81
132.53
131.62
131.57
128.69
128.61
126.02
125.87
124.20
124.17
123.43
118.96
115.68
115.46
77.48
77.16
76.84
40.37
33.36
33.33
17.10
0.14
F
O
Br
O
Me
3i
13C NMR (101 MHz, CDCl3)
210 200 190 180 170 160 150 140 130 120 110 100 90 80 70 60 50 40 30 20 10 0 -10
f1 (ppm)

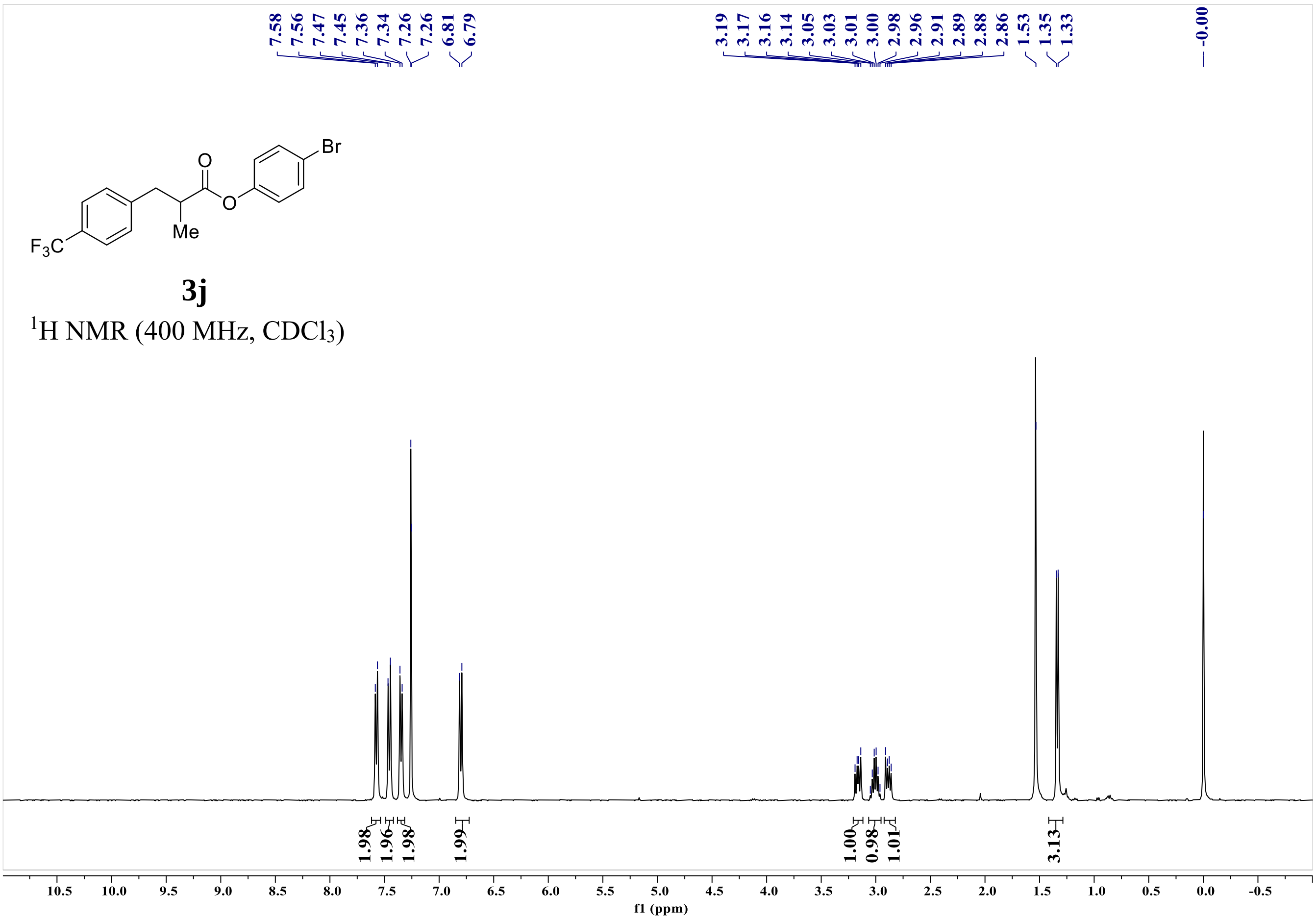
7.58
7.56
7.47
7.45
7.36
7.34
7.26
7.26
6.81
6.79
3.19
3.17
3.16
3.14
3.05
3.03
3.01
3.00
2.98
2.96
2.91
2.89
2.88
2.86
1.53
1.35
1.33
-0.00
O
Br
O
Me
F3C
3j
1H NMR (400 MHz, CDCl3)
1.98
1.96
1.98
1.99
1.00
0.98
1.01
3.13
10.5 10.0 9.5 9.0 8.5 8.0 7.5 7.0 6.5 6.0 5.5 5.0 4.5 4.0 3.5 3.0 2.5 2.0 1.5 1.0 0.5 0.0 -0.5
f1 (ppm)

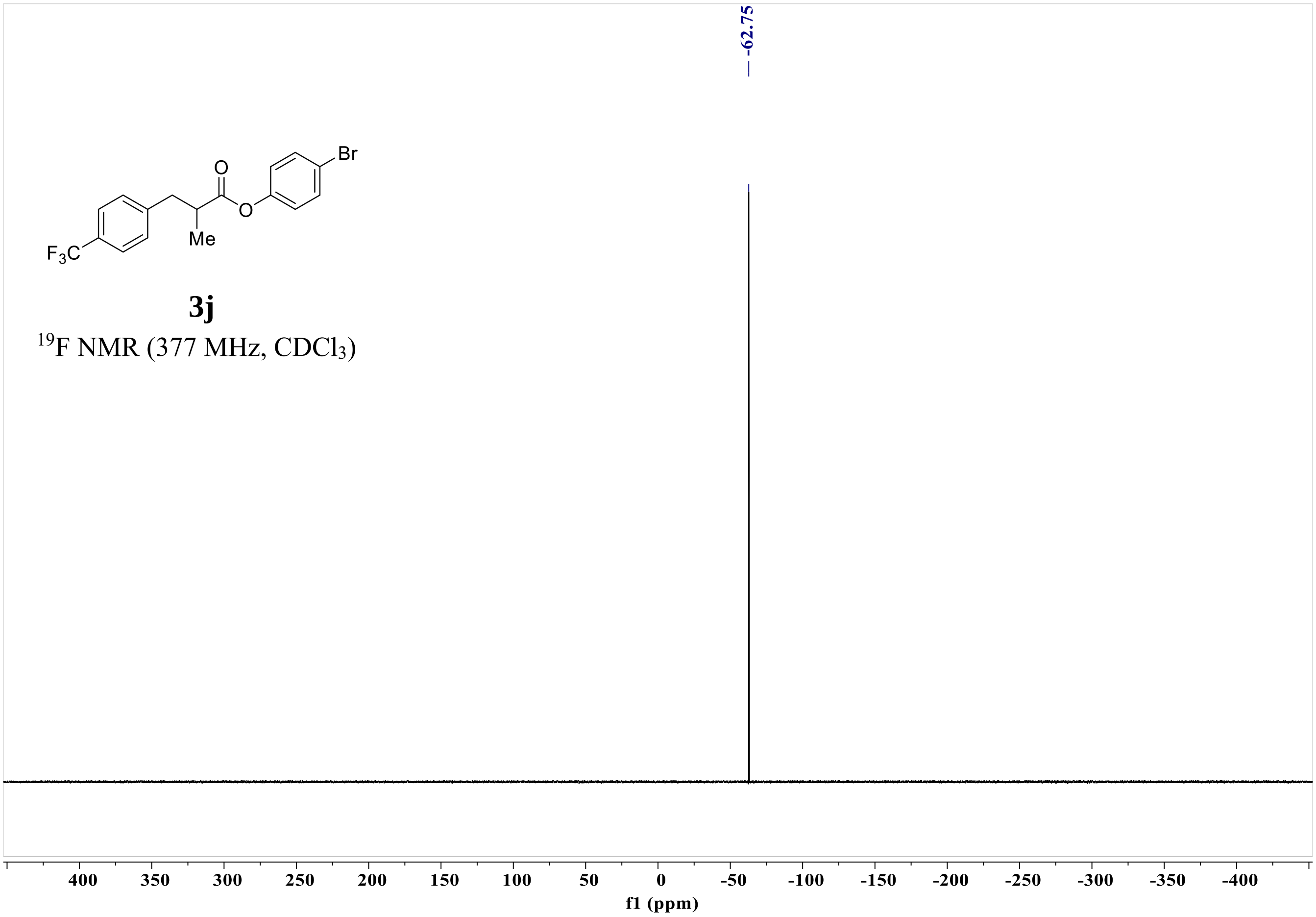

3j

^{19}F NMR (377 MHz, $CDCl_3$)

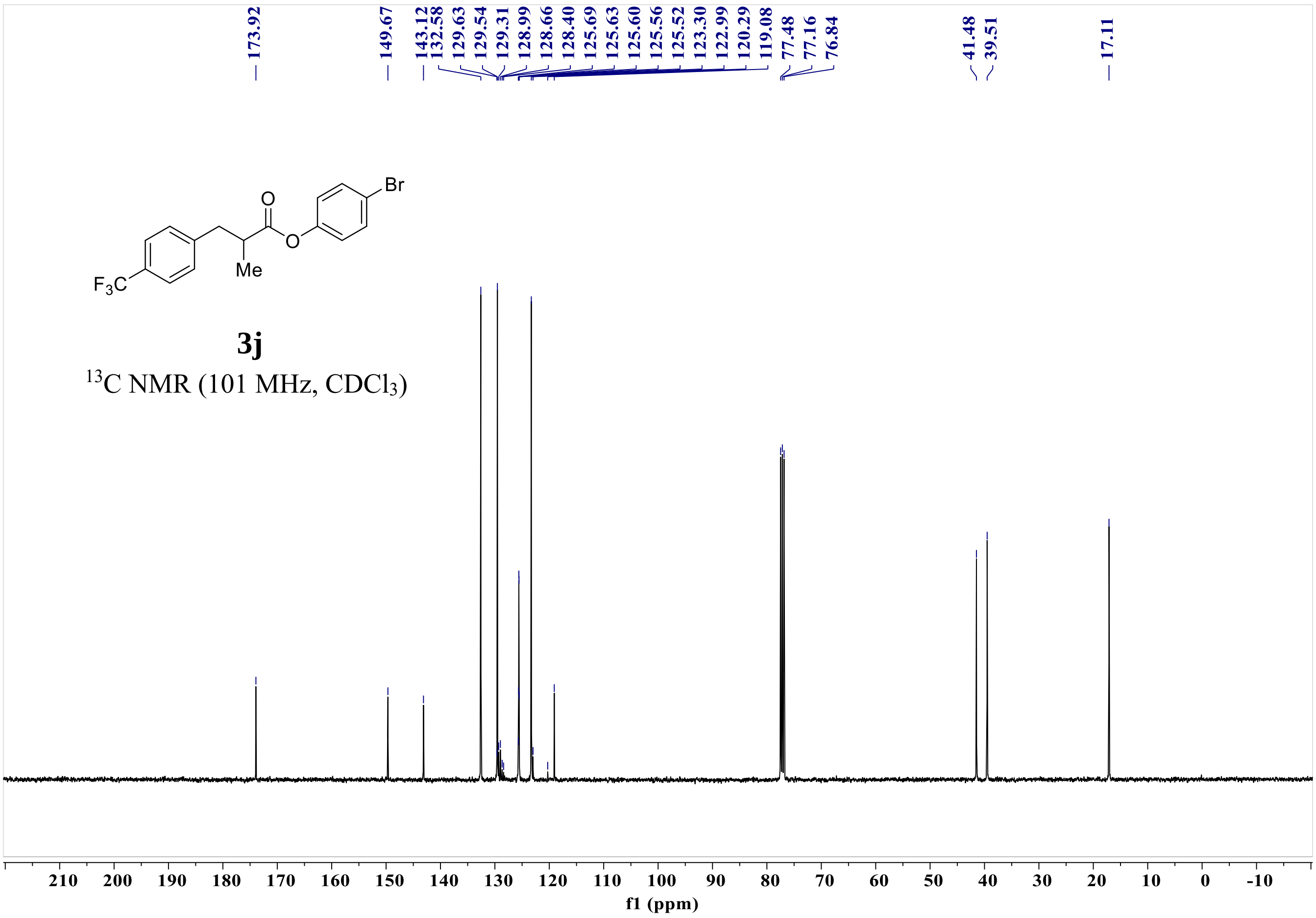

3j

^{13}C NMR (101 MHz, $CDCl_3$)

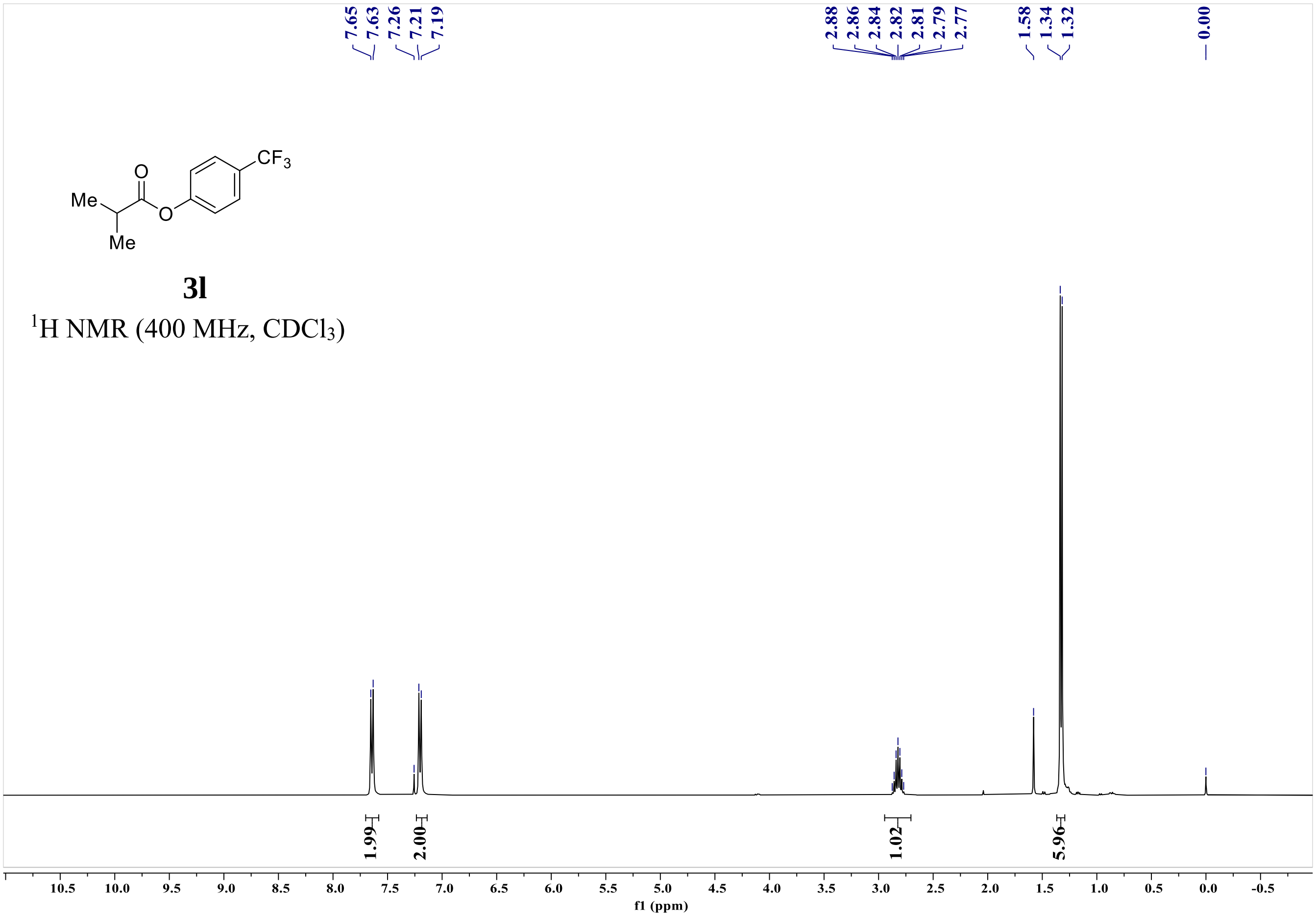
7.65
7.63
7.26
7.21
7.19
2.88
2.86
2.84
2.82
2.81
2.79
2.77
1.58
1.34
1.32
0.00
CF3
O
Me
O
Me
3l
1H NMR (400 MHz, CDCl3)
1.99
2.00
1.02
5.96
f1 (ppm)

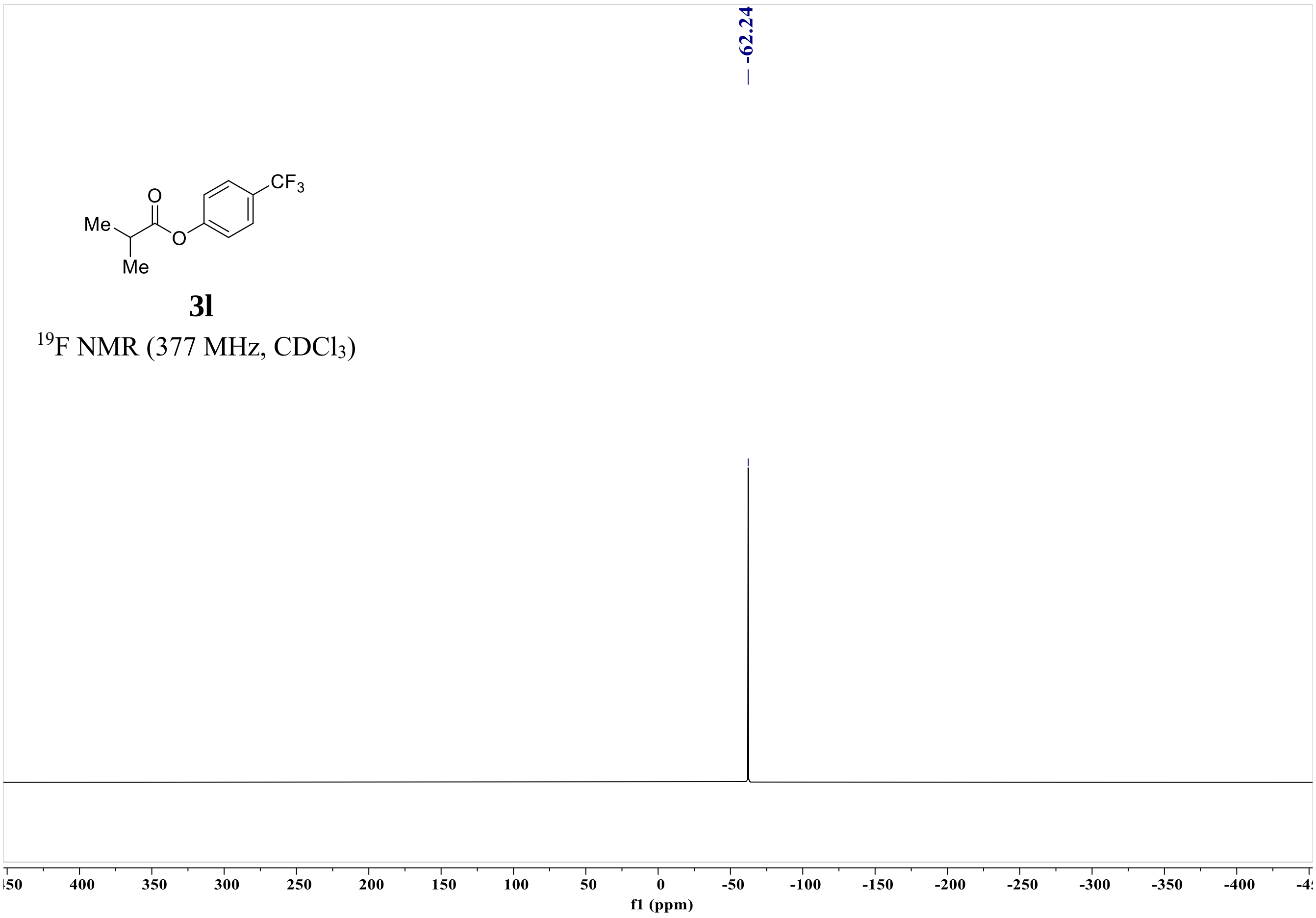
-62.24
CF3
O
Me
O
Me
3l
19F NMR (377 MHz, CDCl3)
f1 (ppm)

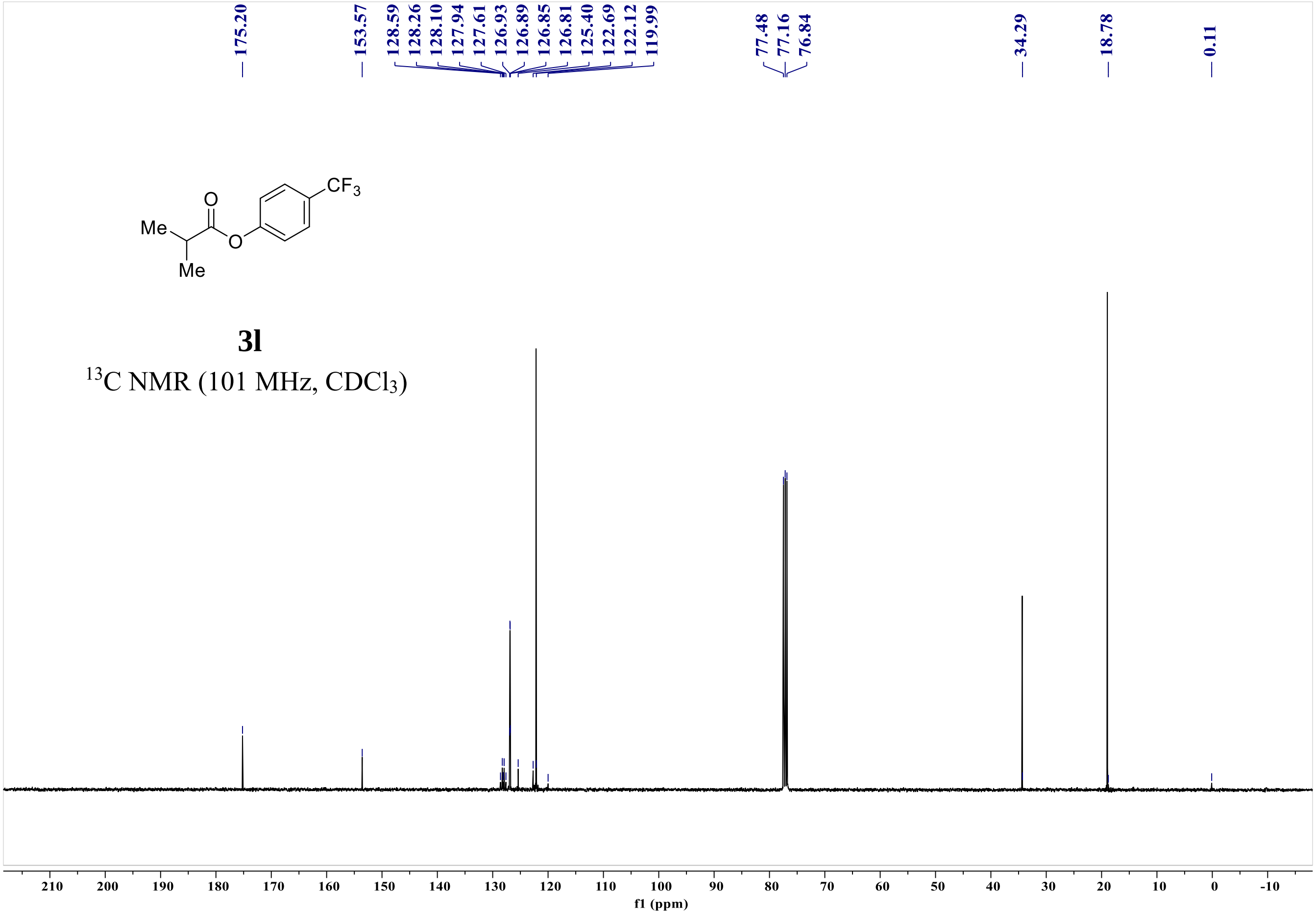

175.20
153.57
128.59
128.26
128.10
127.94
127.61
126.93
126.89
126.85
126.81
125.40
122.69
122.12
119.99
77.48
77.16
76.84
34.29
18.78
0.11
CF3
O
Me
O
Me
3l
13C NMR (101 MHz, CDCl3)
210 200 190 180 170 160 150 140 130 120 110 100 90 80 70 60 50 40 30 20 10 0 -10
f1 (ppm)

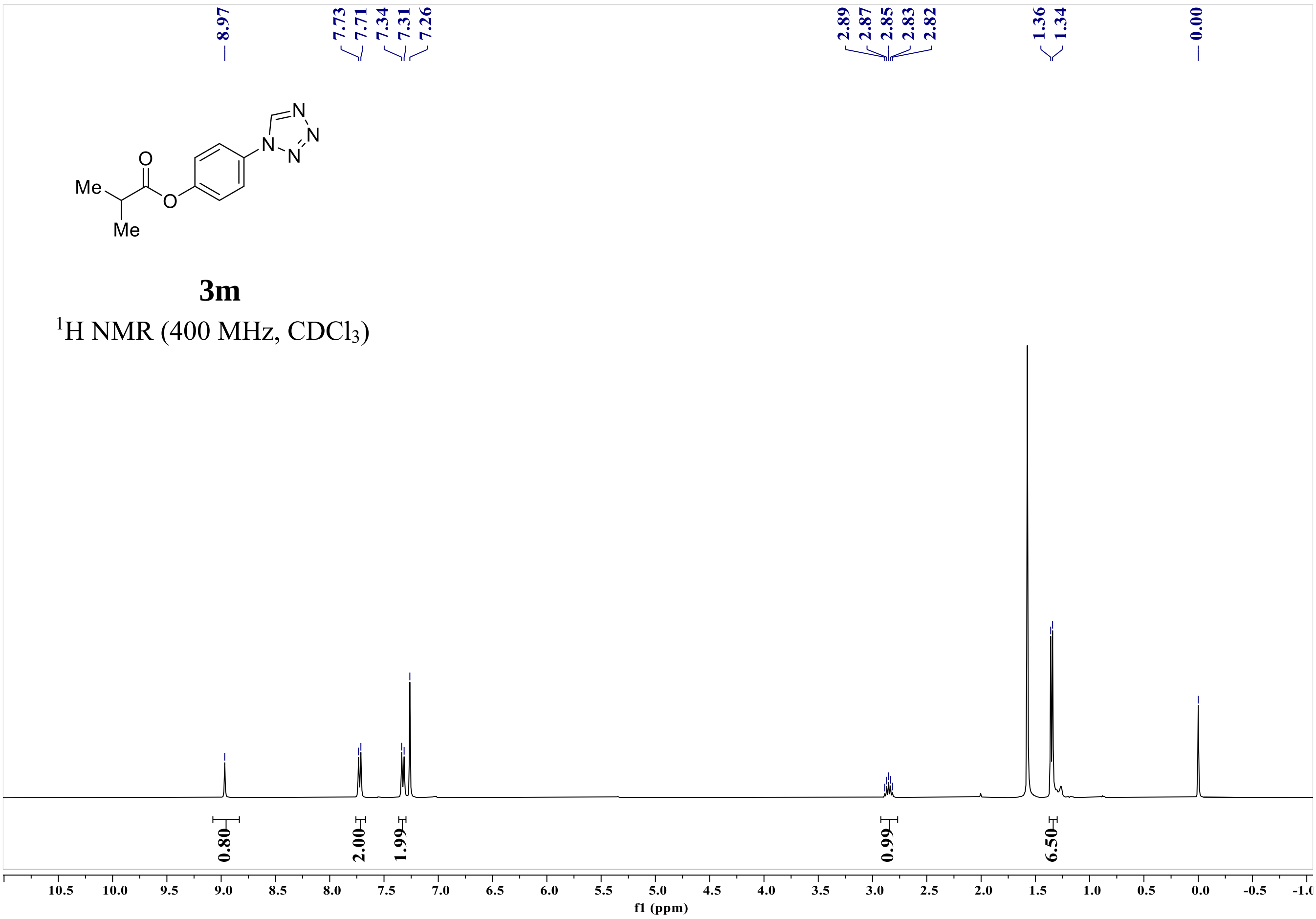

8.97
7.73
7.71
7.34
7.31
7.26
2.89
2.87
2.85
2.83
2.82
1.36
1.34
0.00
N
N
N
N
O
Me
O
Me
3m
1H NMR (400 MHz, CDCl3)
0.80
2.00
1.99
0.99
6.50
10.5 10.0 9.5 9.0 8.5 8.0 7.5 7.0 6.5 6.0 5.5 5.0 4.5 4.0 3.5 3.0 2.5 2.0 1.5 1.0 0.5 0.0 -0.5
f1 (ppm)

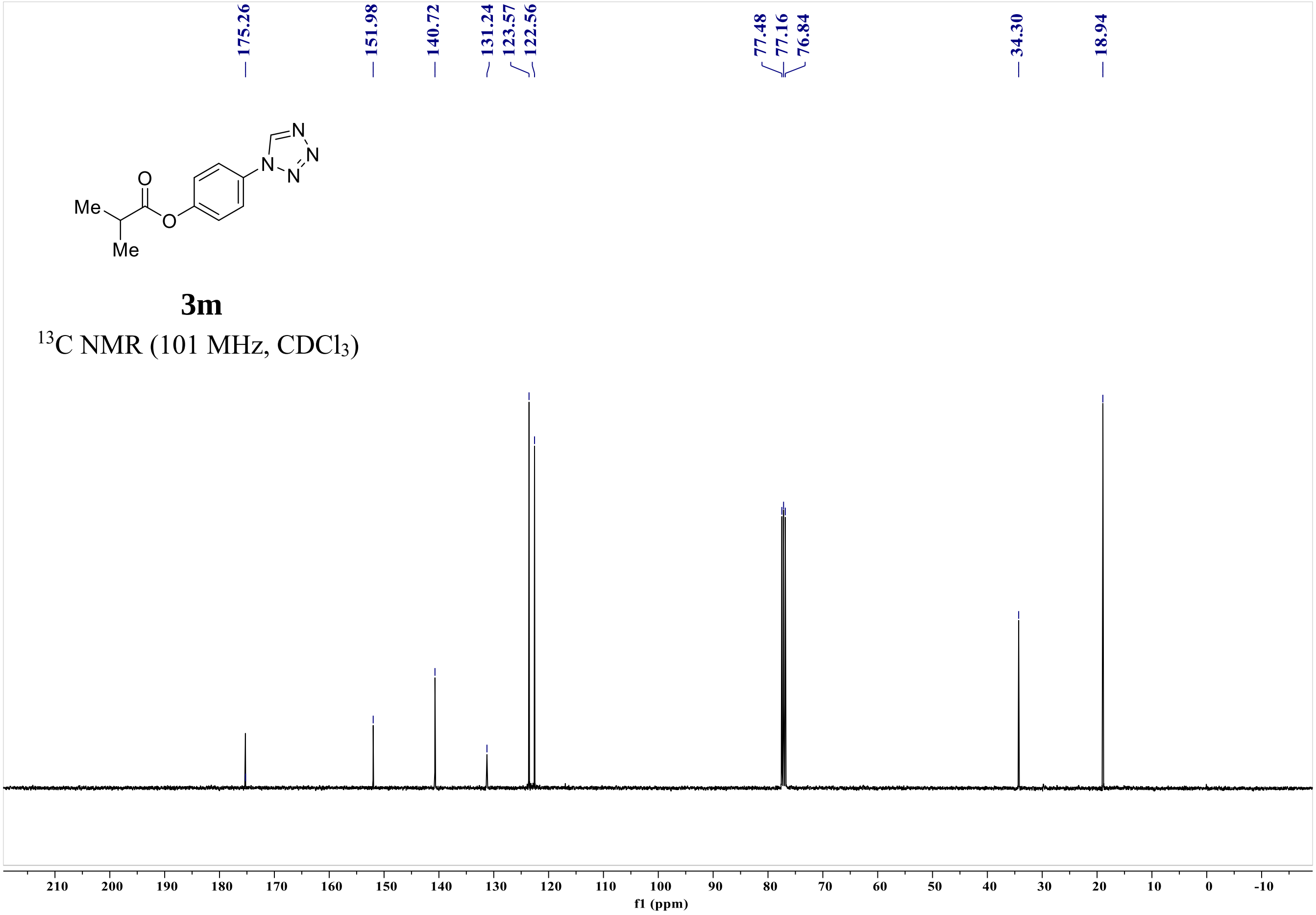

3m

^{13}C NMR (101 MHz, $CDCl_3$)

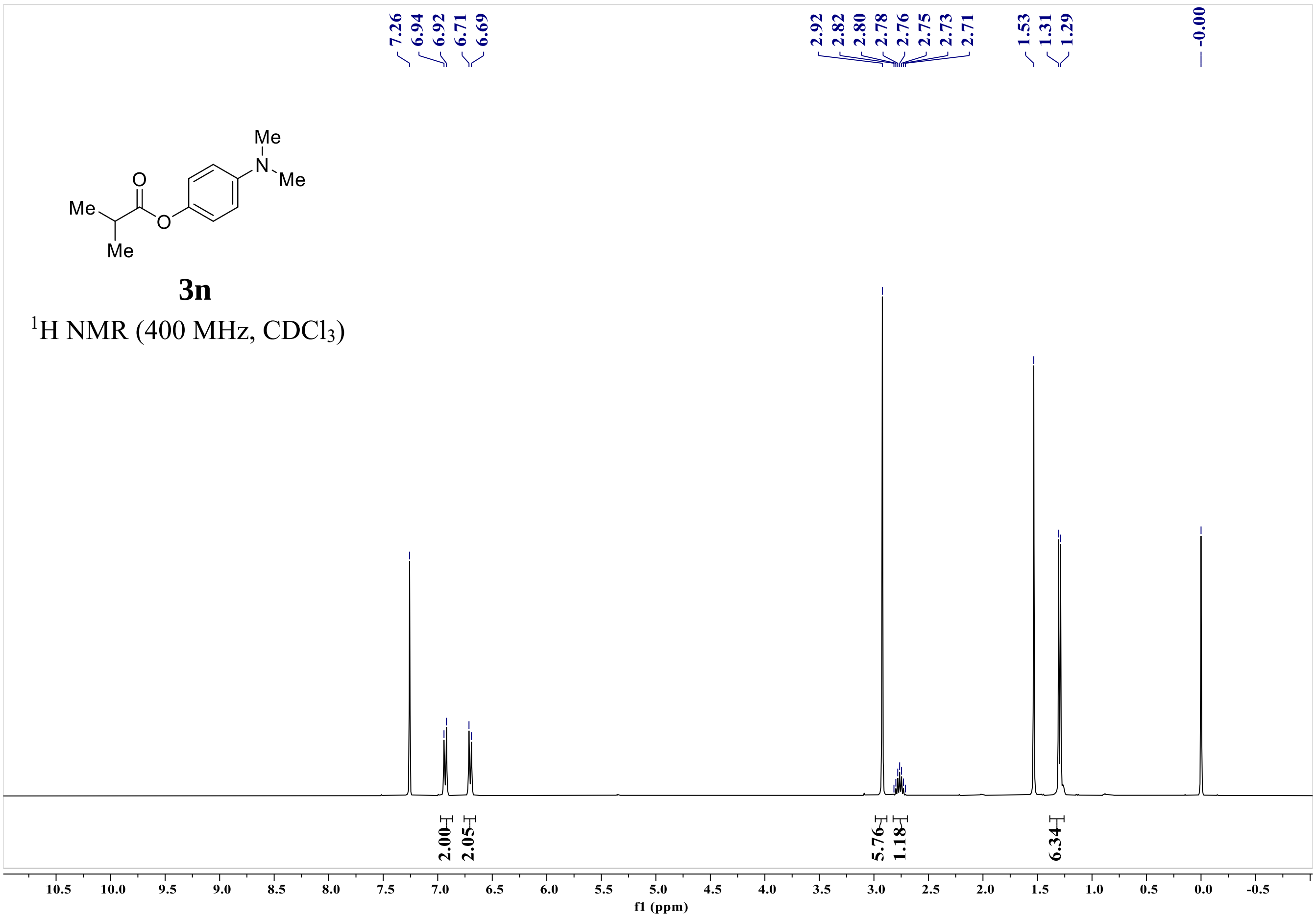

3n

^{1}H NMR (400 MHz, $CDCl_3$)

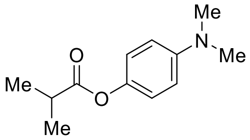

3n

^{13}C NMR (101 MHz, $CDCl_3$)

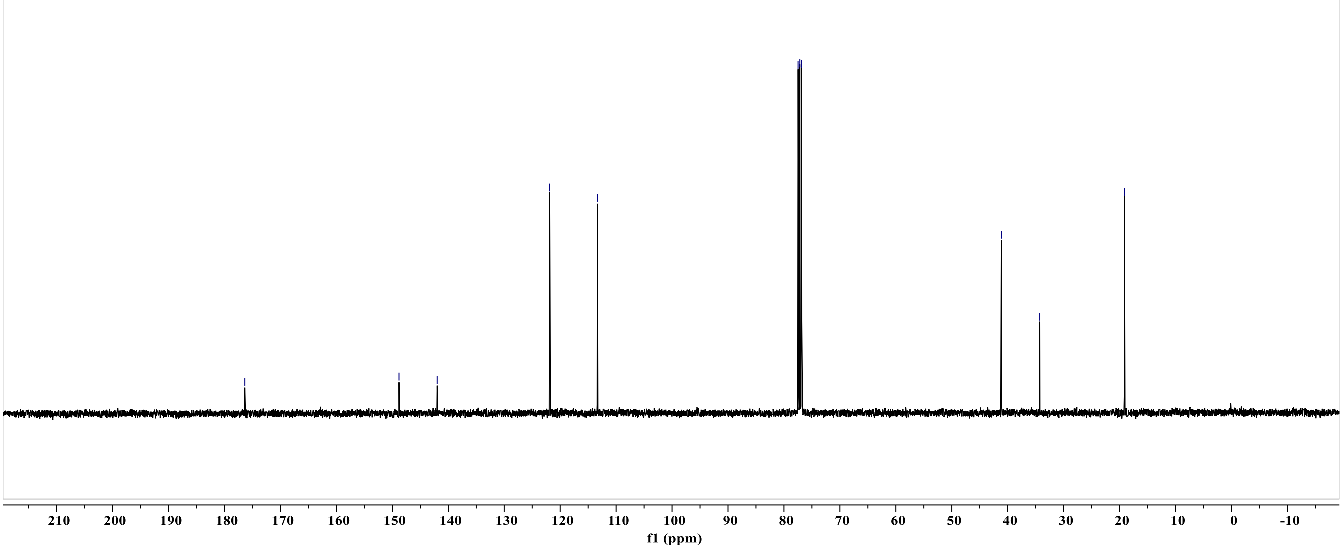

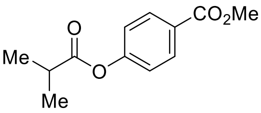

3p

^{1}H NMR (400 MHz, $CDCl_3$)

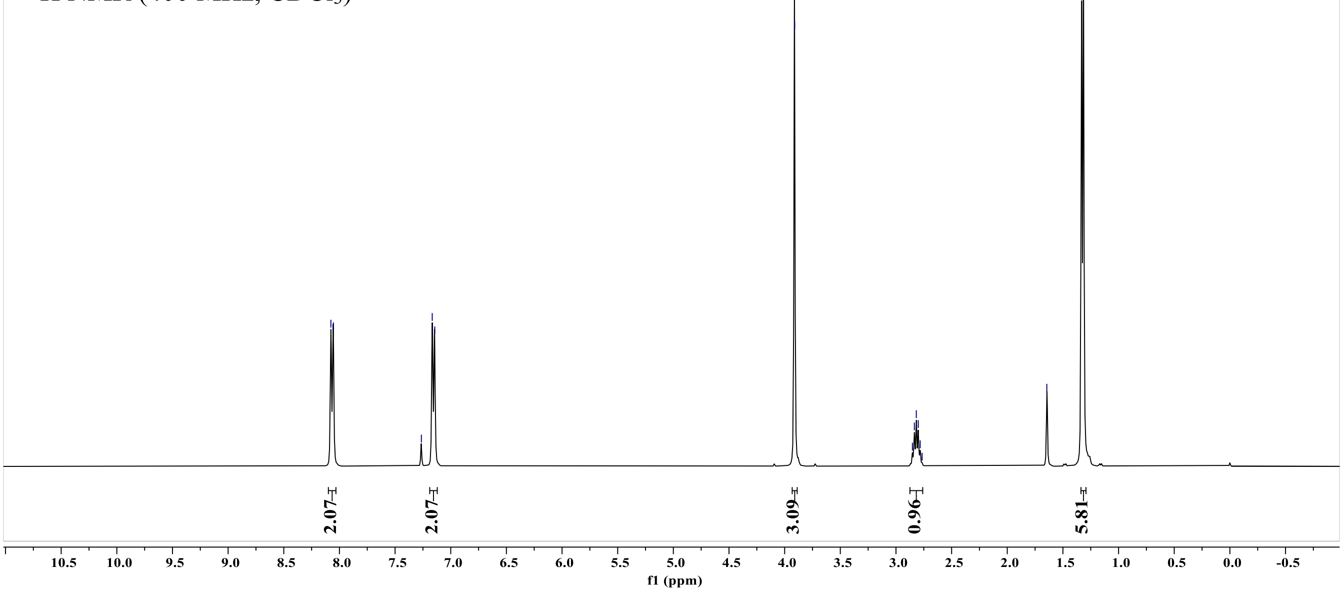

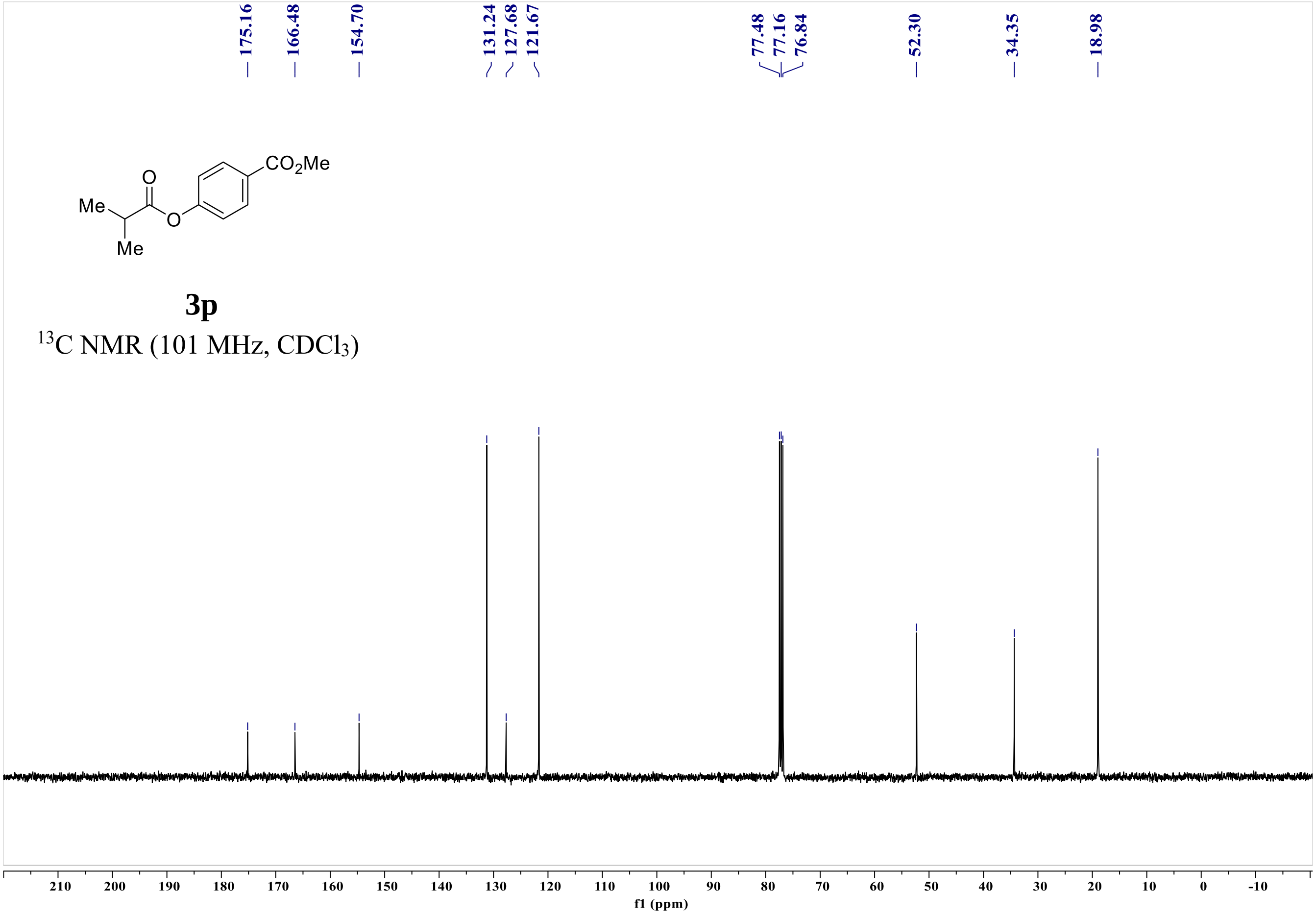
175.16
166.48
154.70
131.24
127.68
121.67
77.48
77.16
76.84
52.30
34.35
18.98
CO2Me
O
Me
O
Me
3p
13C NMR (101 MHz, CDCl3)
210 200 190 180 170 160 150 140 130 120 110 100 90 80 70 60 50 40 30 20 10 0 -10
f1 (ppm)

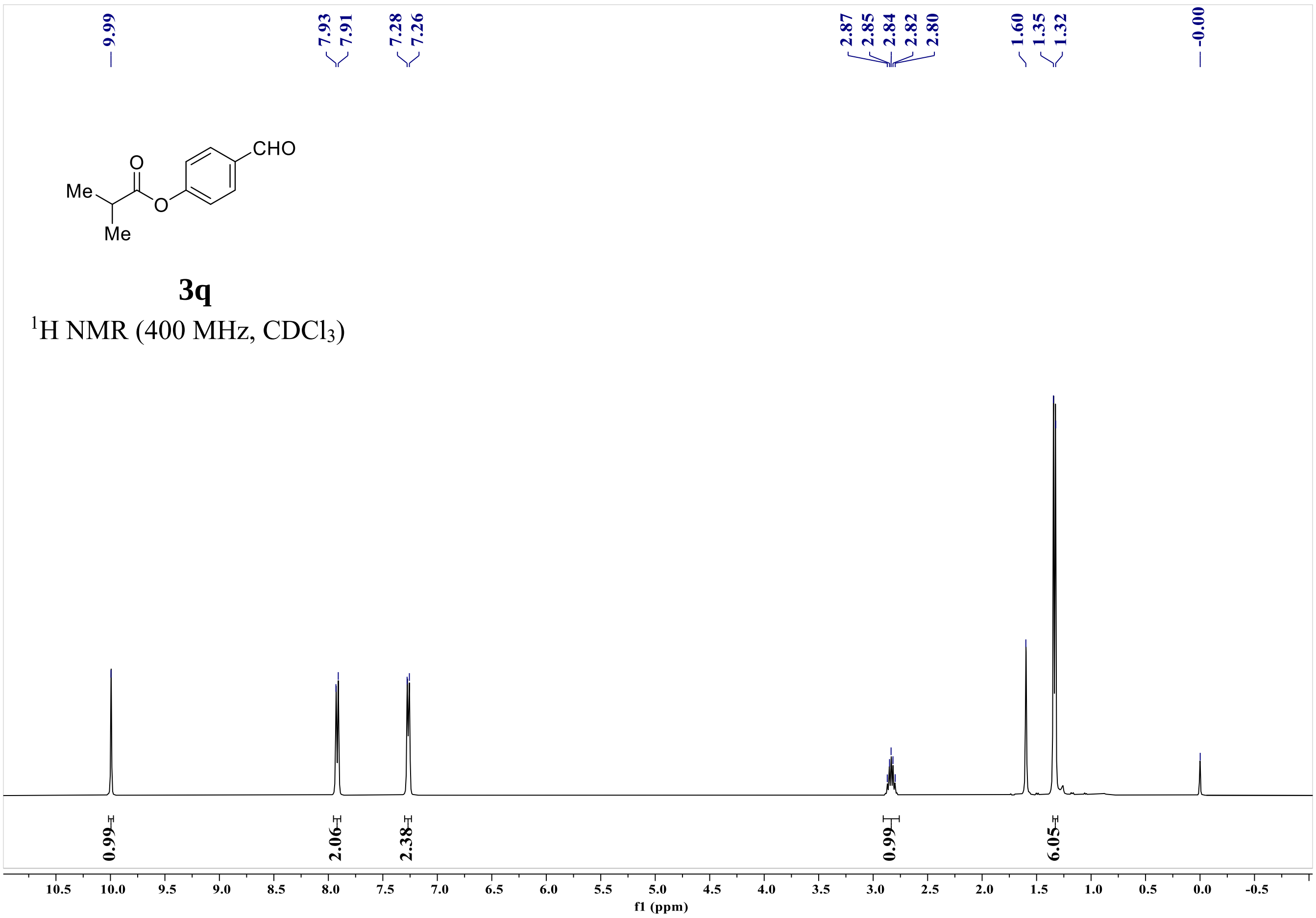
9.99
7.93
7.91
7.28
7.26
2.87
2.85
2.84
2.82
2.80
1.60
1.35
1.32
-0.00
CHO
O
Me
O
Me
3q
1H NMR (400 MHz, CDCl3)
0.99
2.06
2.38
0.99
6.05
10.5 10.0 9.5 9.0 8.5 8.0 7.5 7.0 6.5 6.0 5.5 5.0 4.5 4.0 3.5 3.0 2.5 2.0 1.5 1.0 0.5 0.0 -0.5
f1 (ppm)

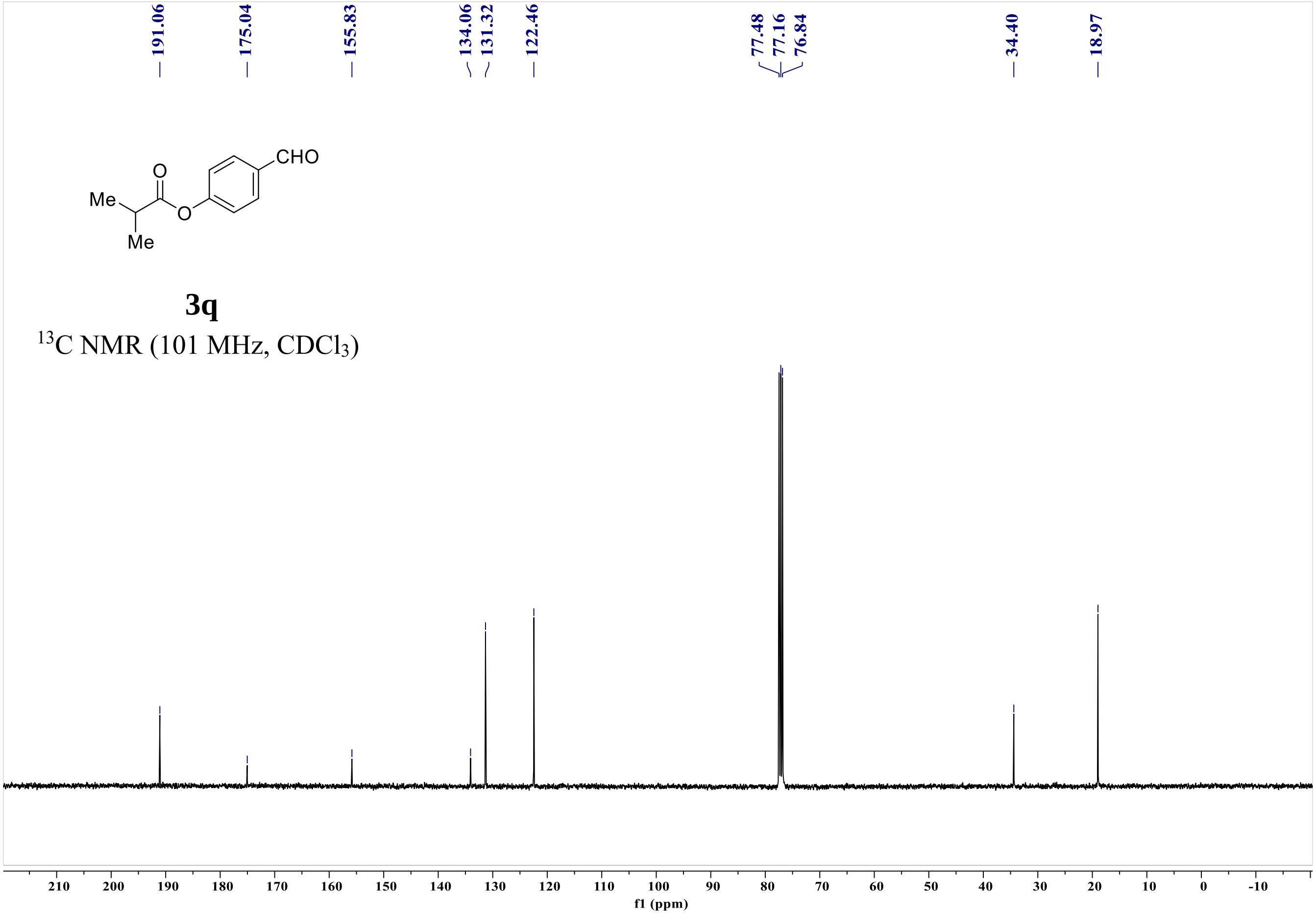
191.06
175.04
155.83
134.06
131.32
122.46
77.48
77.16
76.84
34.40
18.97
CHO
O
Me
O
Me
3q
13C NMR (101 MHz, CDCl3)
210
200
190
180
170
160
150
140
130
120
110
100
90
80
70
60
50
40
30
20
10
0
-10
f1 (ppm)

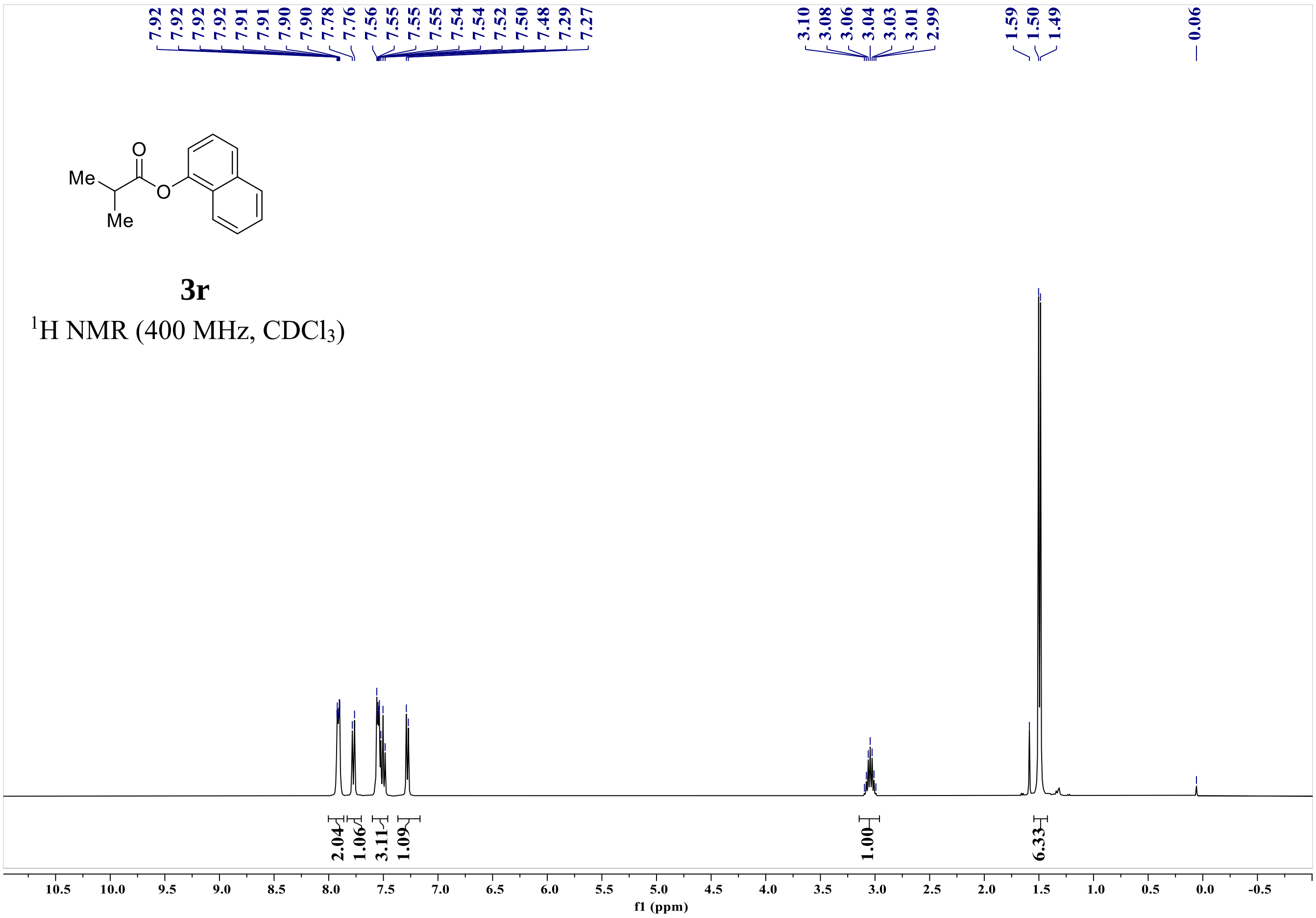
7.92
7.92
7.92
7.92
7.91
7.91
7.90
7.90
7.78
7.76
7.56
7.55
7.55
7.54
7.54
7.52
7.50
7.48
7.29
7.27
3.10
3.08
3.06
3.04
3.03
3.01
2.99
1.59
1.50
1.49
0.06
O
Me
O
Me
3r
1H NMR (400 MHz, CDCl3)
2.04
1.06
3.11
1.09
1.00
6.33
10.5
10.0
9.5
9.0
8.5
8.0
7.5
7.0
6.5
6.0
5.5
5.0
4.5
4.0
3.5
3.0
2.5
2.0
1.5
1.0
0.5
0.0
-0.5
f1 (ppm)

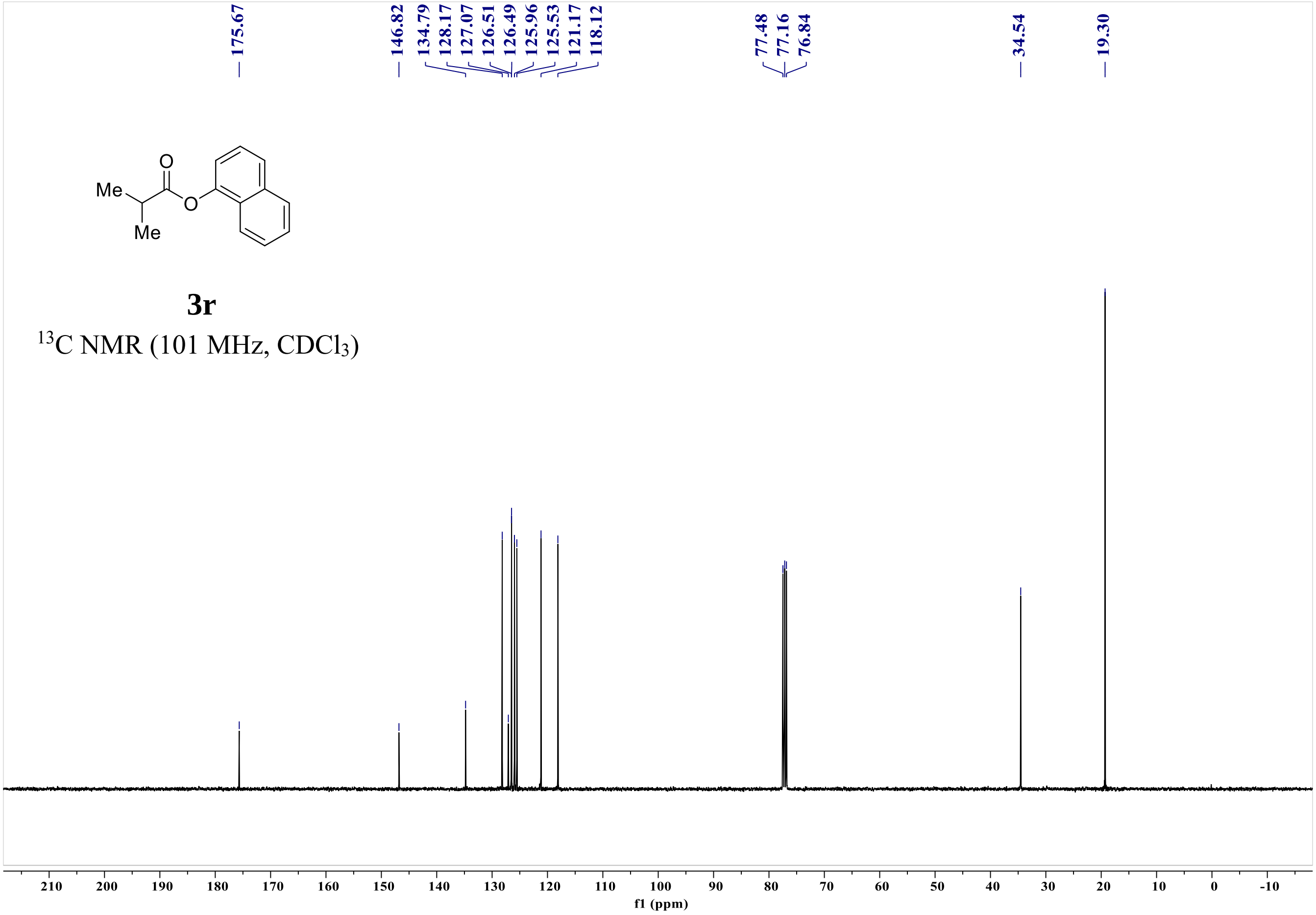

3r

^{13}C NMR (101 MHz, $CDCl_3$)

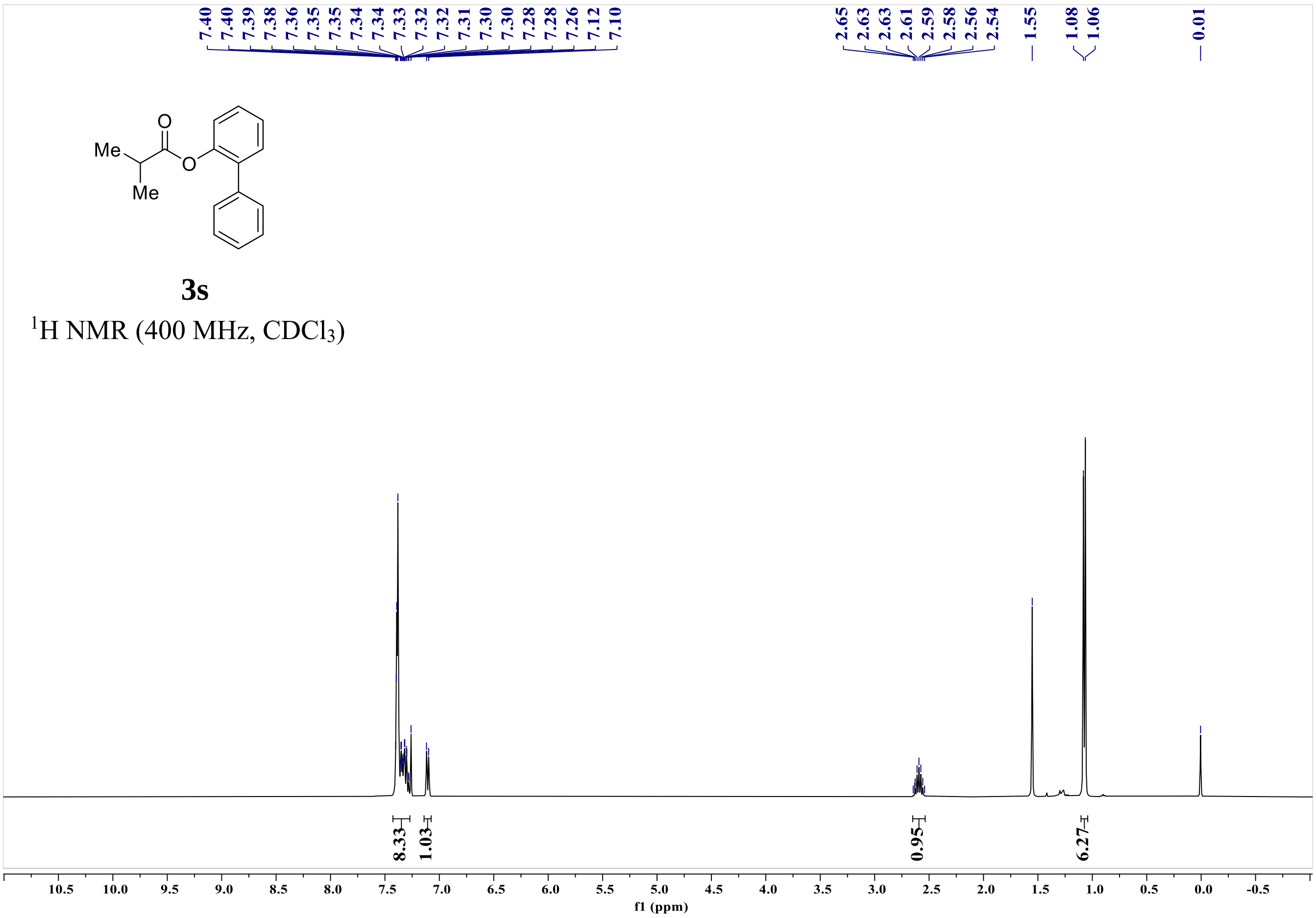

3s

^{1}H NMR (400 MHz, $CDCl_3$)

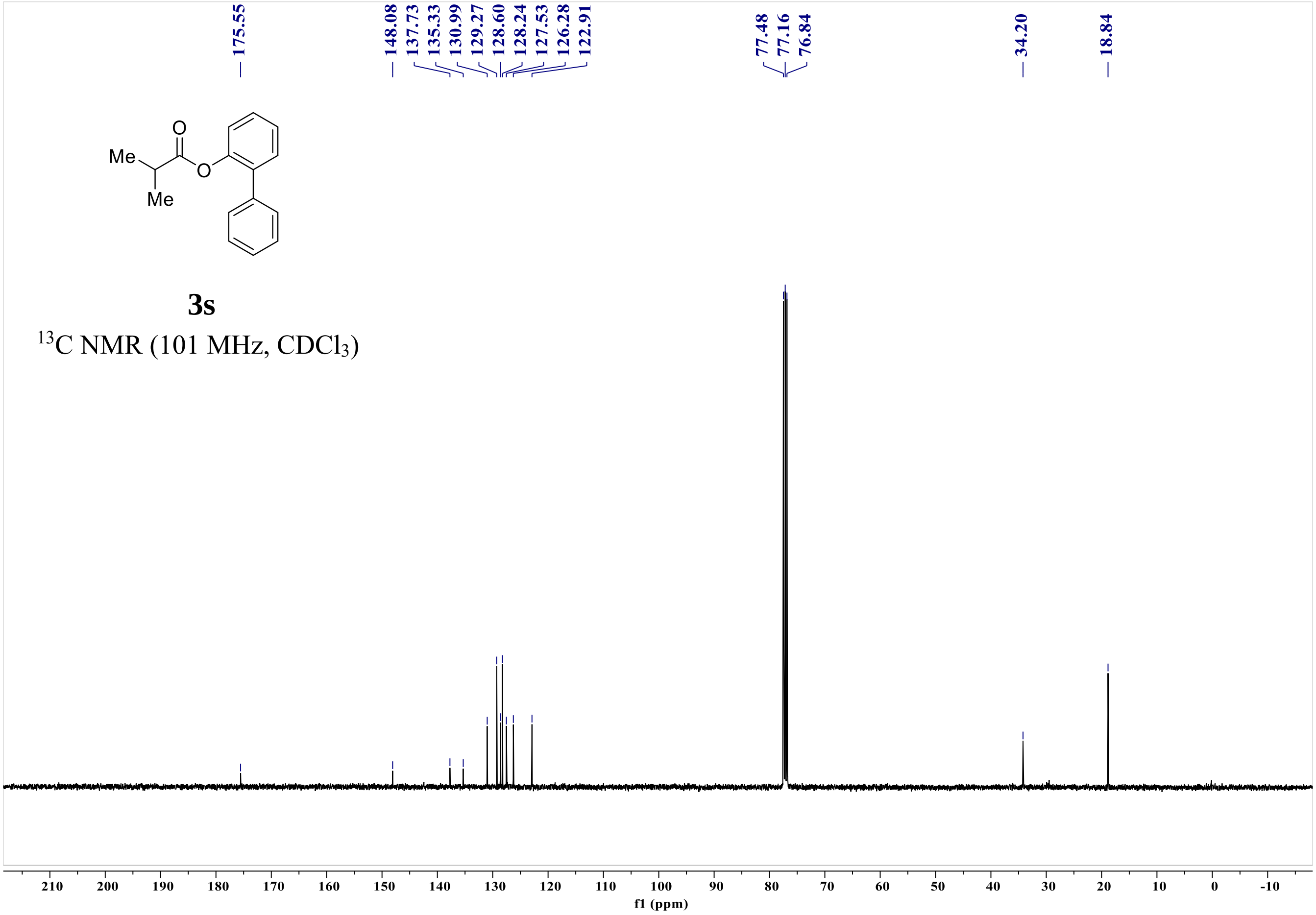

3s

^{13}C NMR (101 MHz, $CDCl_3$)

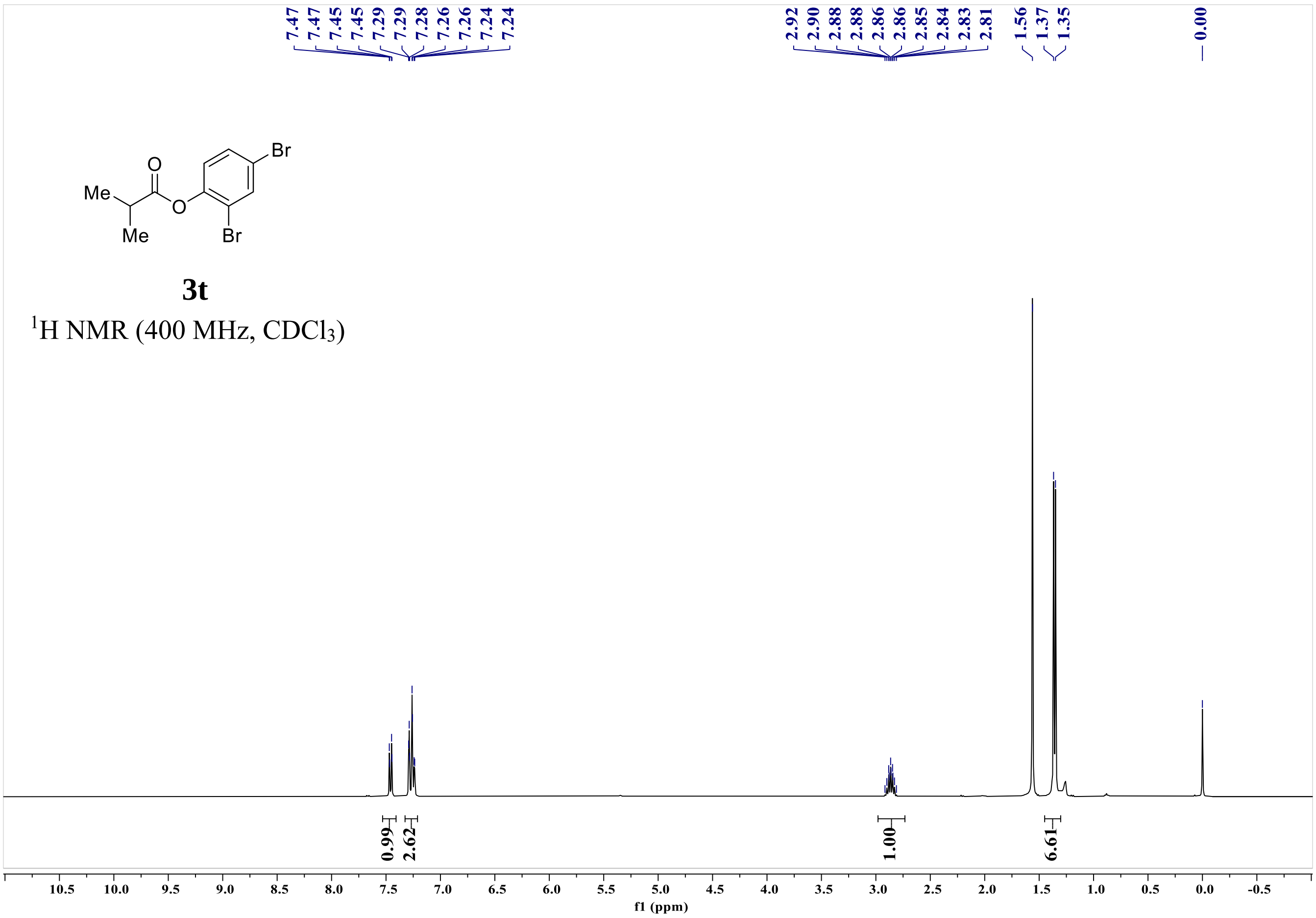

3t

^{1}H NMR (400 MHz, $CDCl_3$)

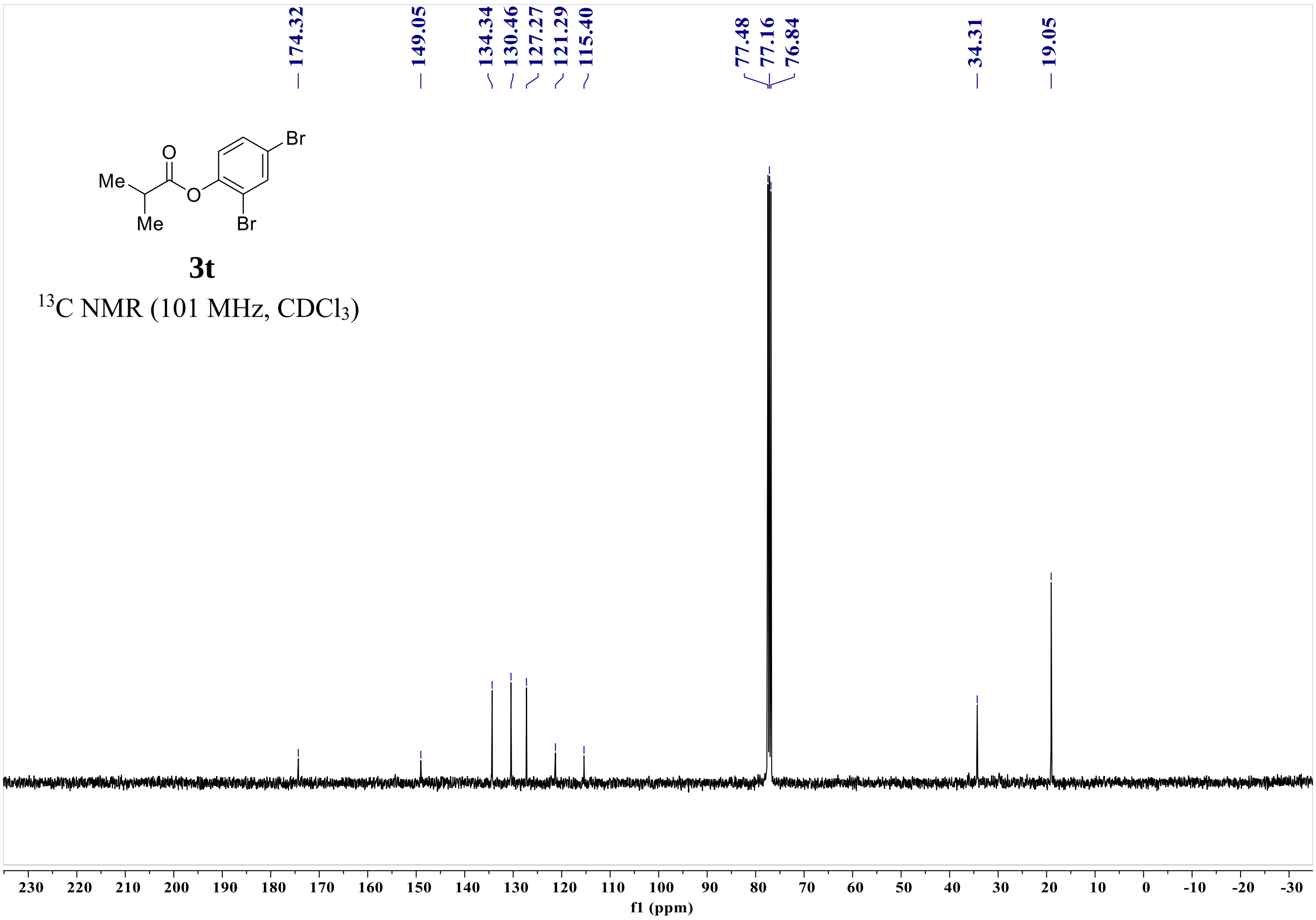
174.32
149.05
134.34
130.46
127.27
121.29
115.40
77.48
77.16
76.84
34.31
19.05
Br
O
Me
O
Me
Br
3t
^{13}C NMR (101 MHz, CDCl$_3$)
230 220 210 200 190 180 170 160 150 140 130 120 110 100 90 80 70 60 50 40 30 20 10 0 -10 -20 -30
f1 (ppm)

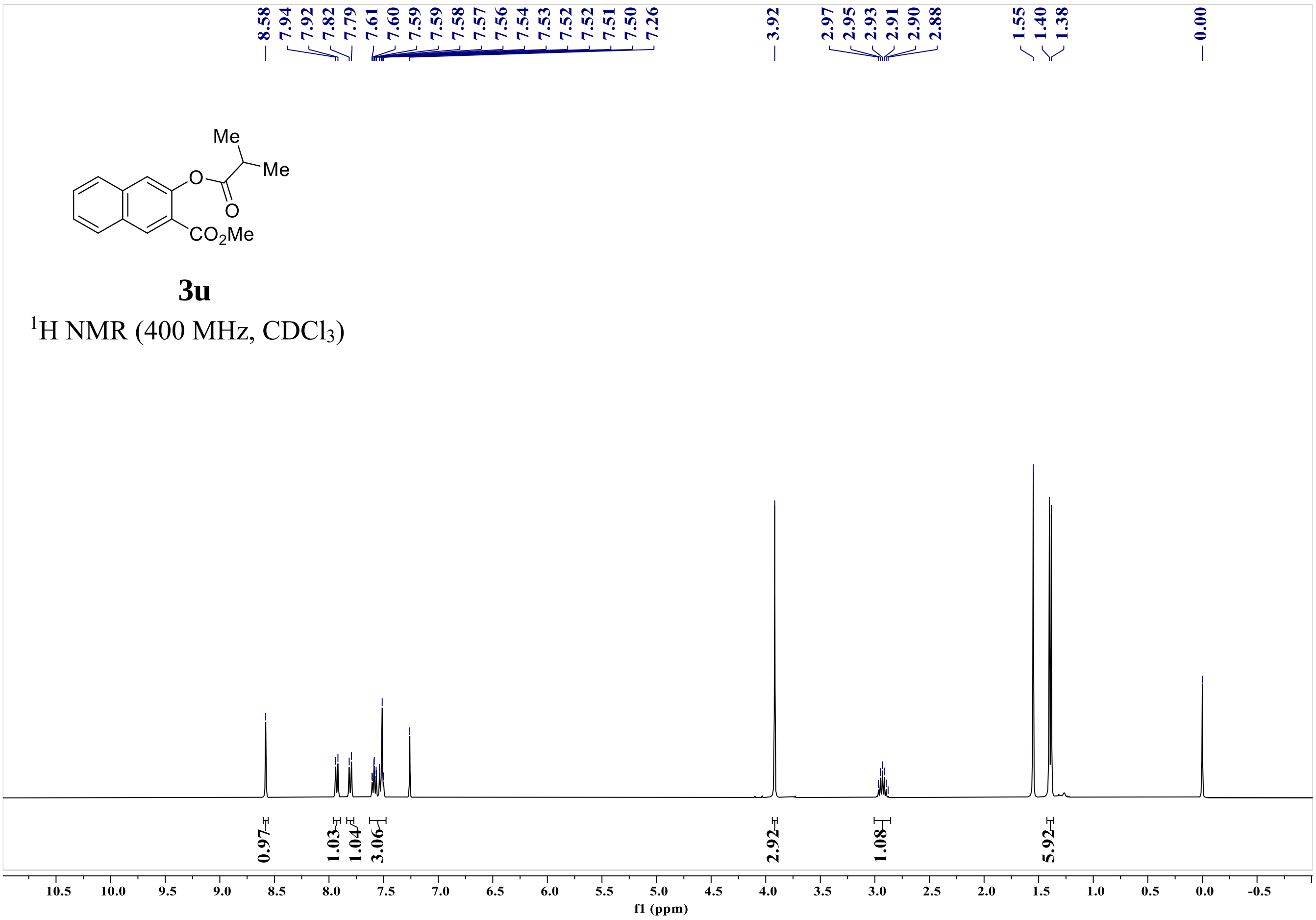
8.58
7.94
7.92
7.82
7.79
7.61
7.60
7.59
7.59
7.58
7.57
7.56
7.54
7.53
7.52
7.52
7.51
7.50
7.26
3.92
2.97
2.95
2.93
2.91
2.90
2.88
1.55
1.40
1.38
0.00
Me
O
Me
O
CO_2Me
3u
^{1}H NMR (400 MHz, CDCl$_3$)
0.97
1.03
1.04
3.06
2.92
1.08
5.92
10.5 10.0 9.5 9.0 8.5 8.0 7.5 7.0 6.5 6.0 5.5 5.0 4.5 4.0 3.5 3.0 2.5 2.0 1.5 1.0 0.5 0.0 -0.5
f1 (ppm)

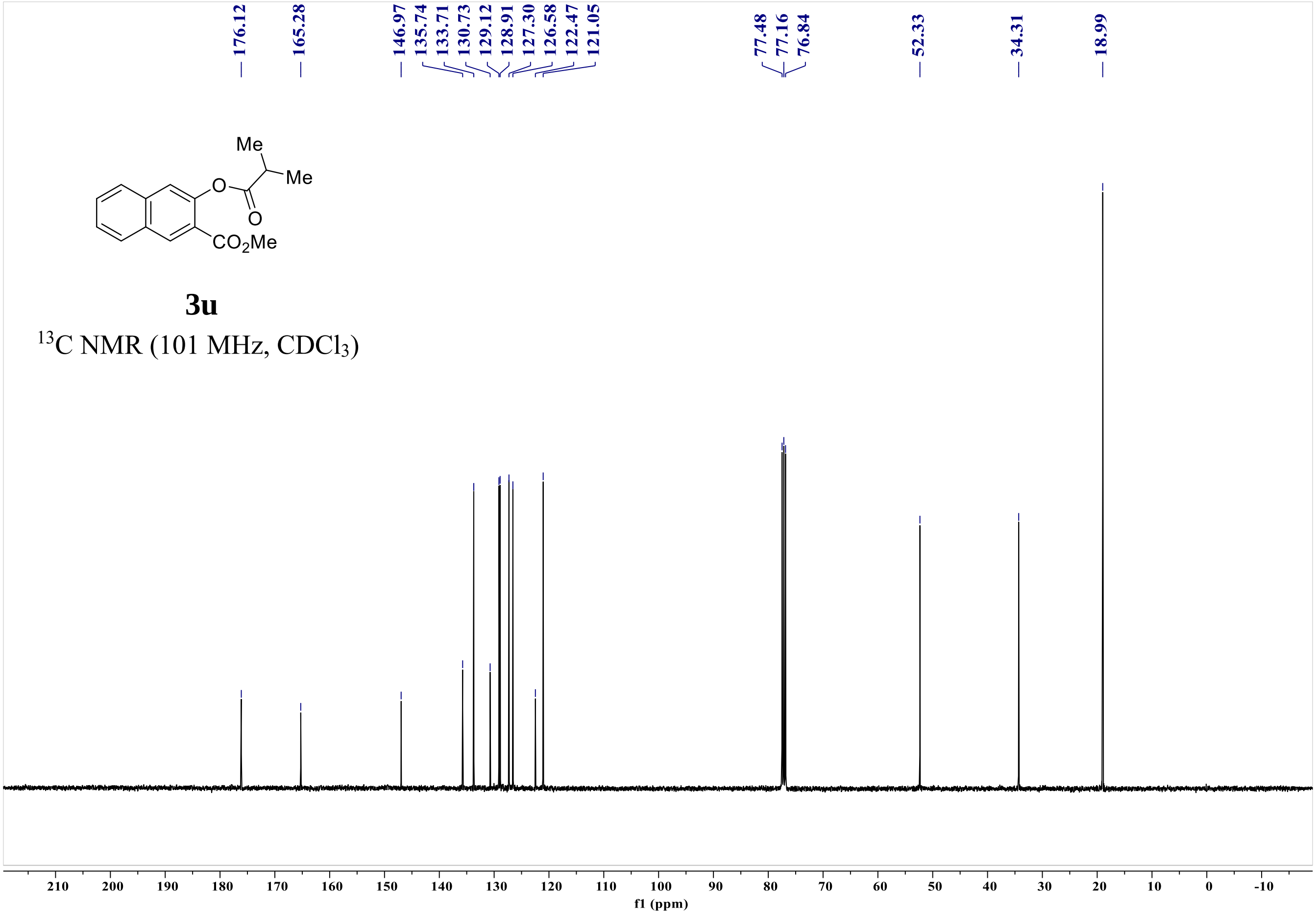
176.12
165.28
146.97
135.74
133.71
130.73
129.12
128.91
127.30
126.58
122.47
121.05
77.48
77.16
76.84
52.33
34.31
18.99
Me
O
Me
O
CO2Me
3u
13C NMR (101 MHz, CDCl3)
f1 (ppm)

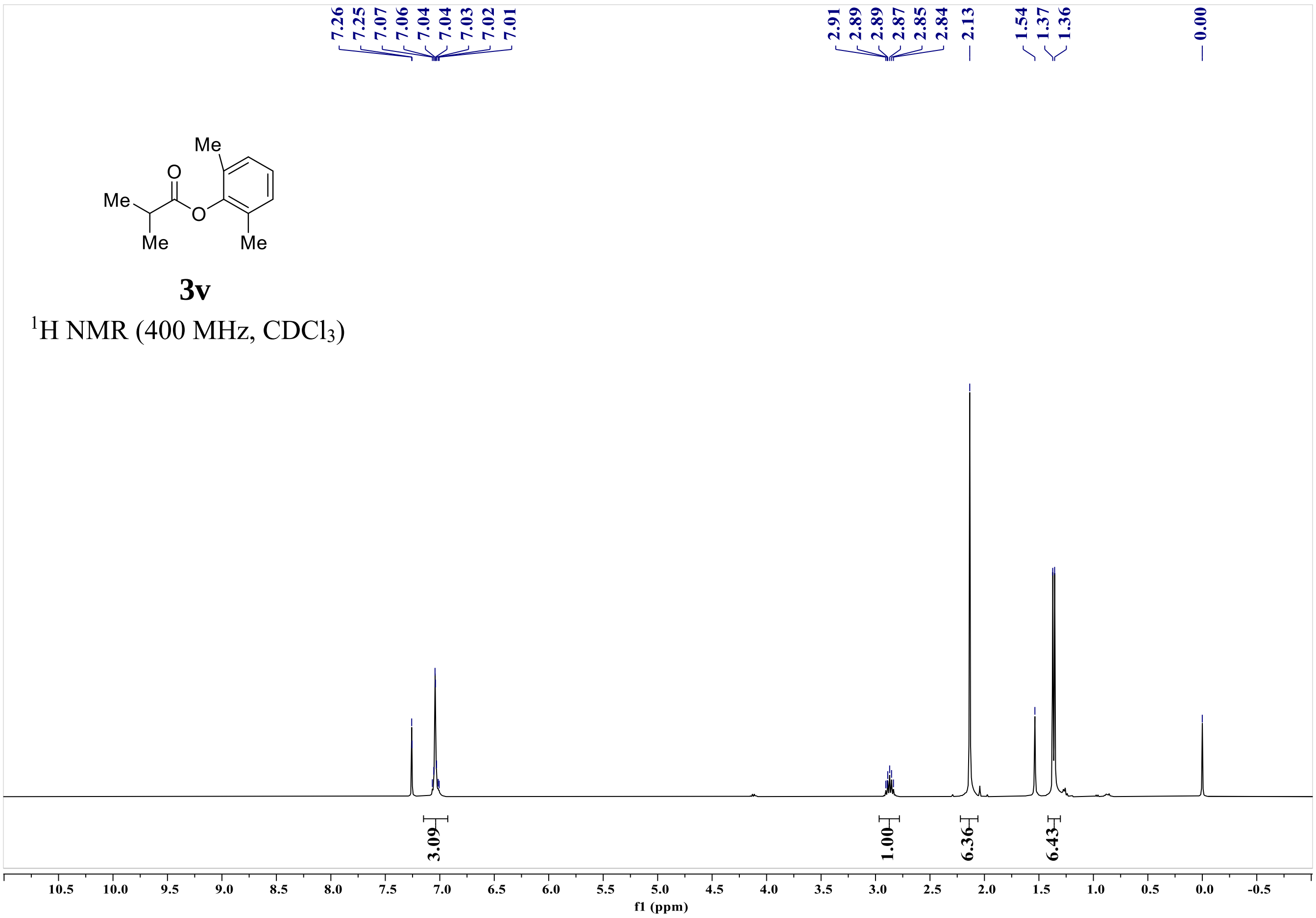
7.26
7.25
7.07
7.06
7.04
7.04
7.03
7.02
7.01
2.91
2.89
2.89
2.87
2.85
2.84
2.13
1.54
1.37
1.36
0.00
Me
O
Me
O
Me
Me
3v
1H NMR (400 MHz, CDCl3)
3.09
1.00
6.36
6.43
f1 (ppm)

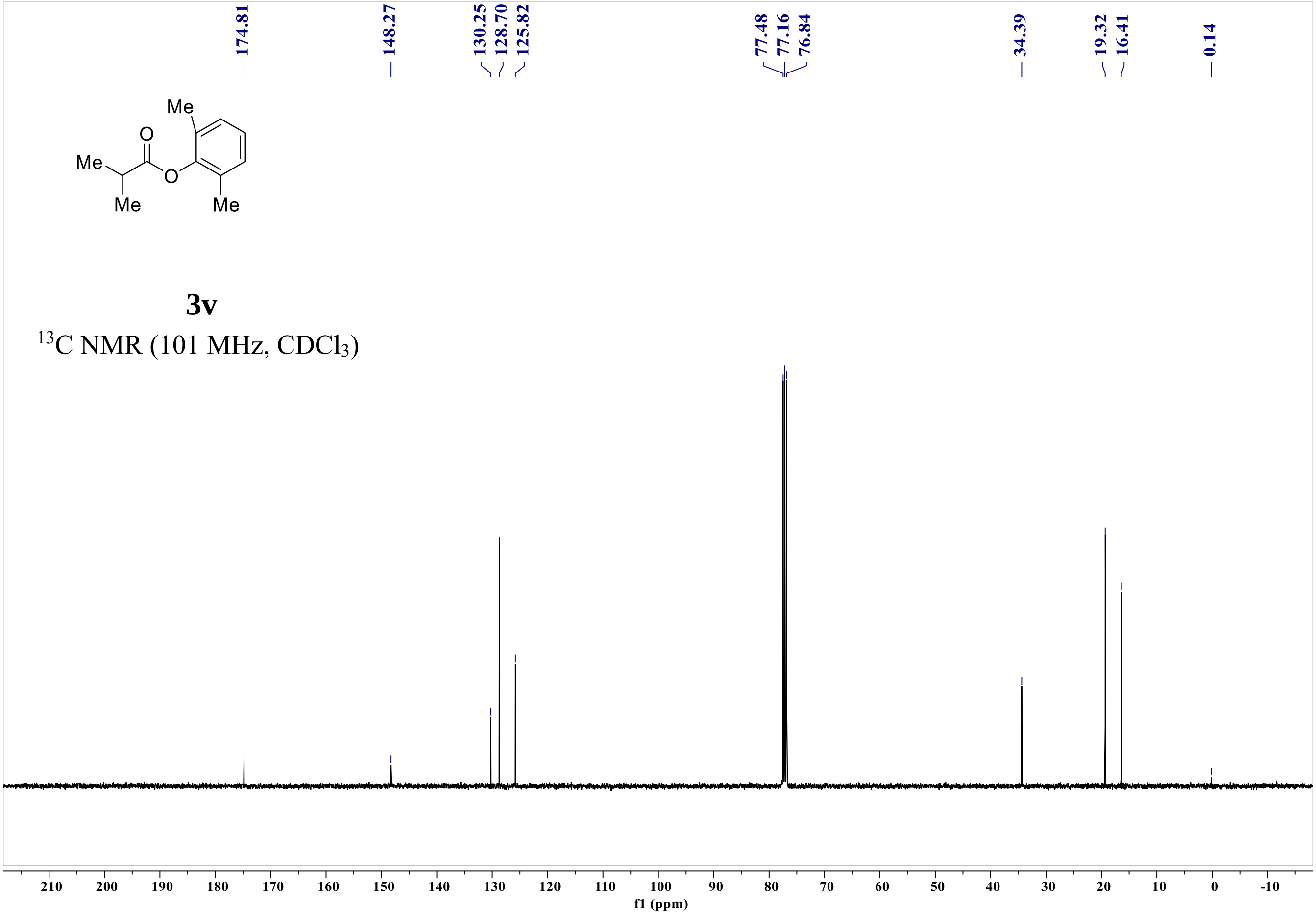

3v

^{13}C NMR (101 MHz, $CDCl_3$)

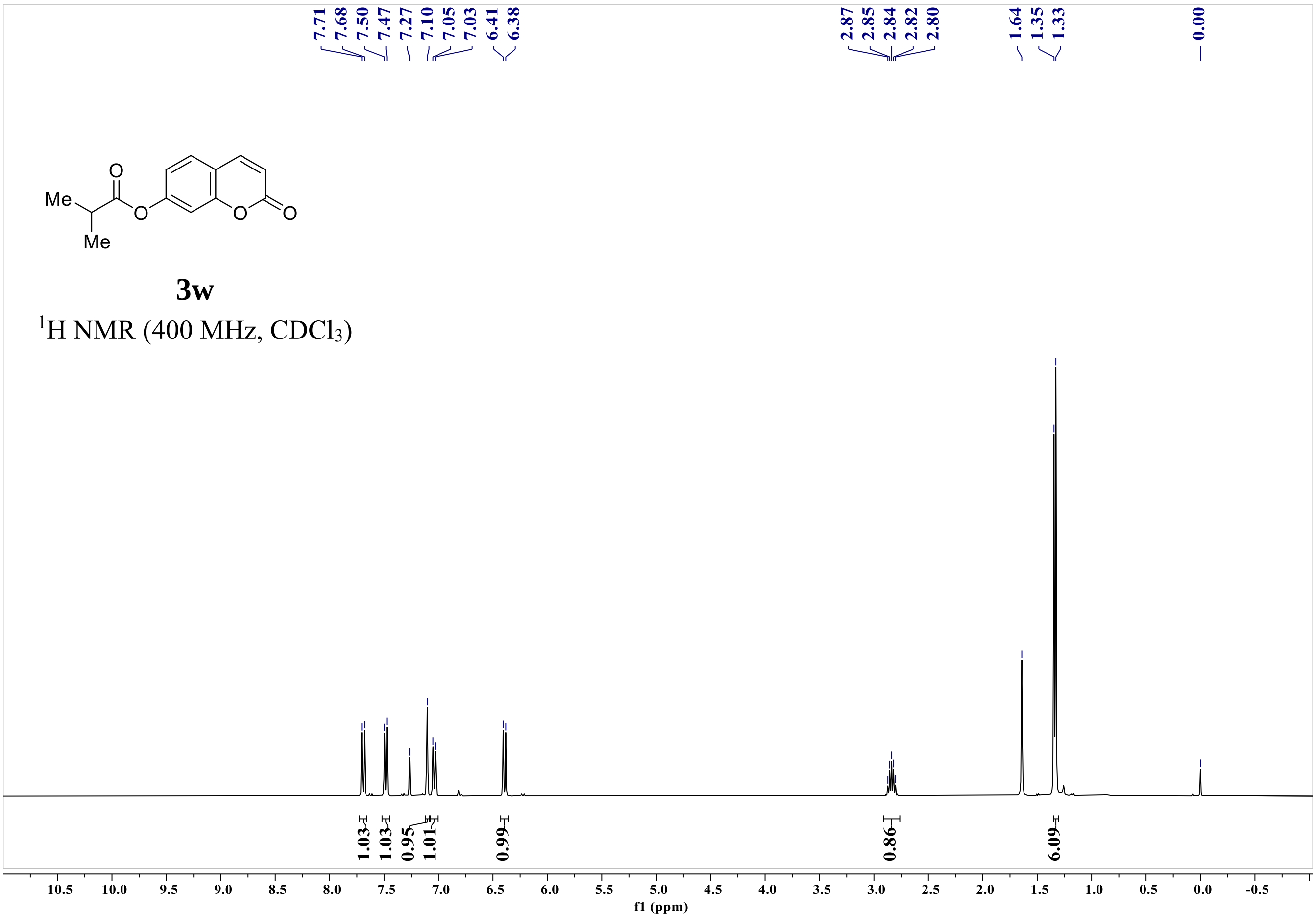

3w

^{1}H NMR (400 MHz, $CDCl_3$)

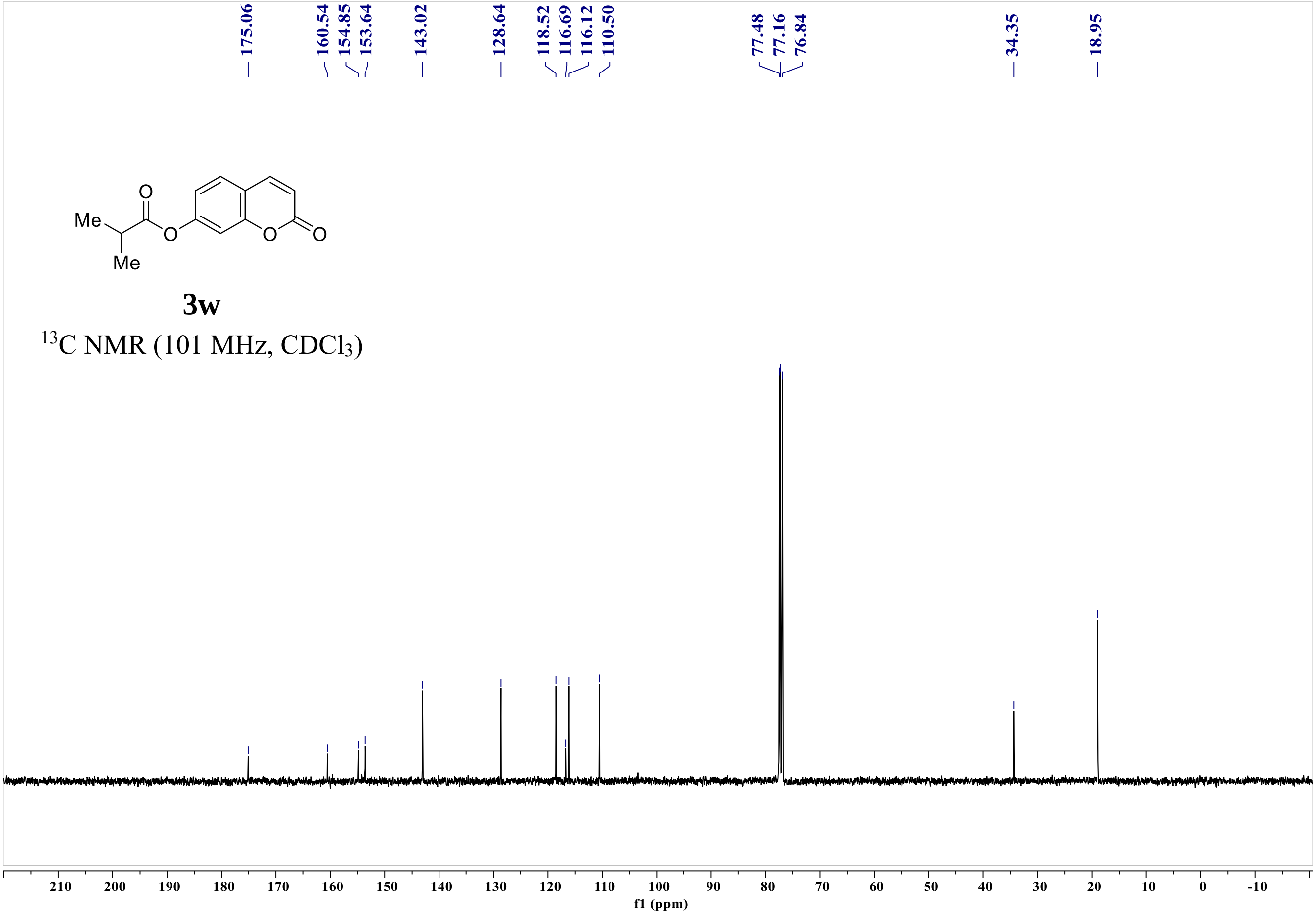

3w

^{13}C NMR (101 MHz, $CDCl_3$)

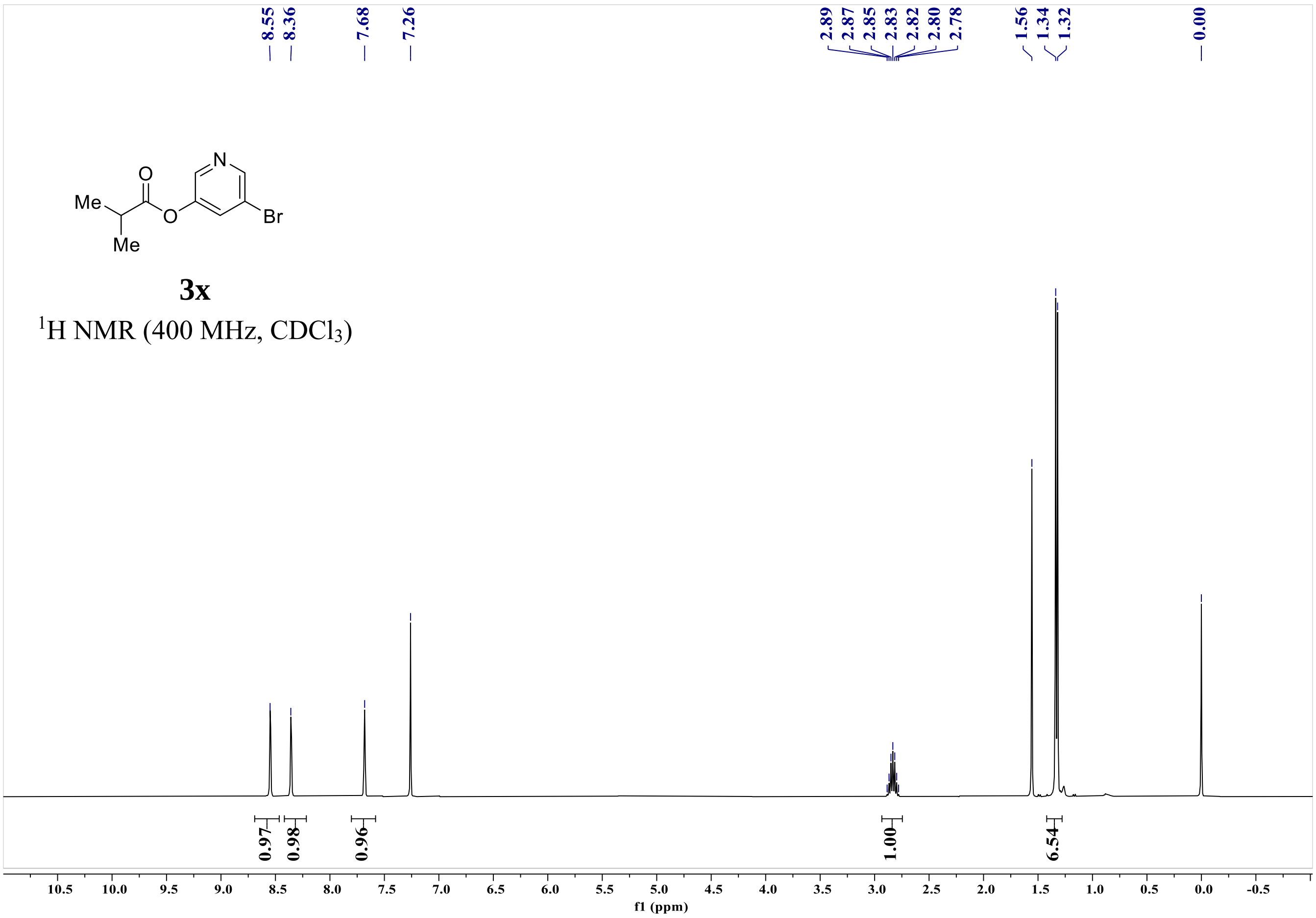

3x

^{1}H NMR (400 MHz, $CDCl_3$)

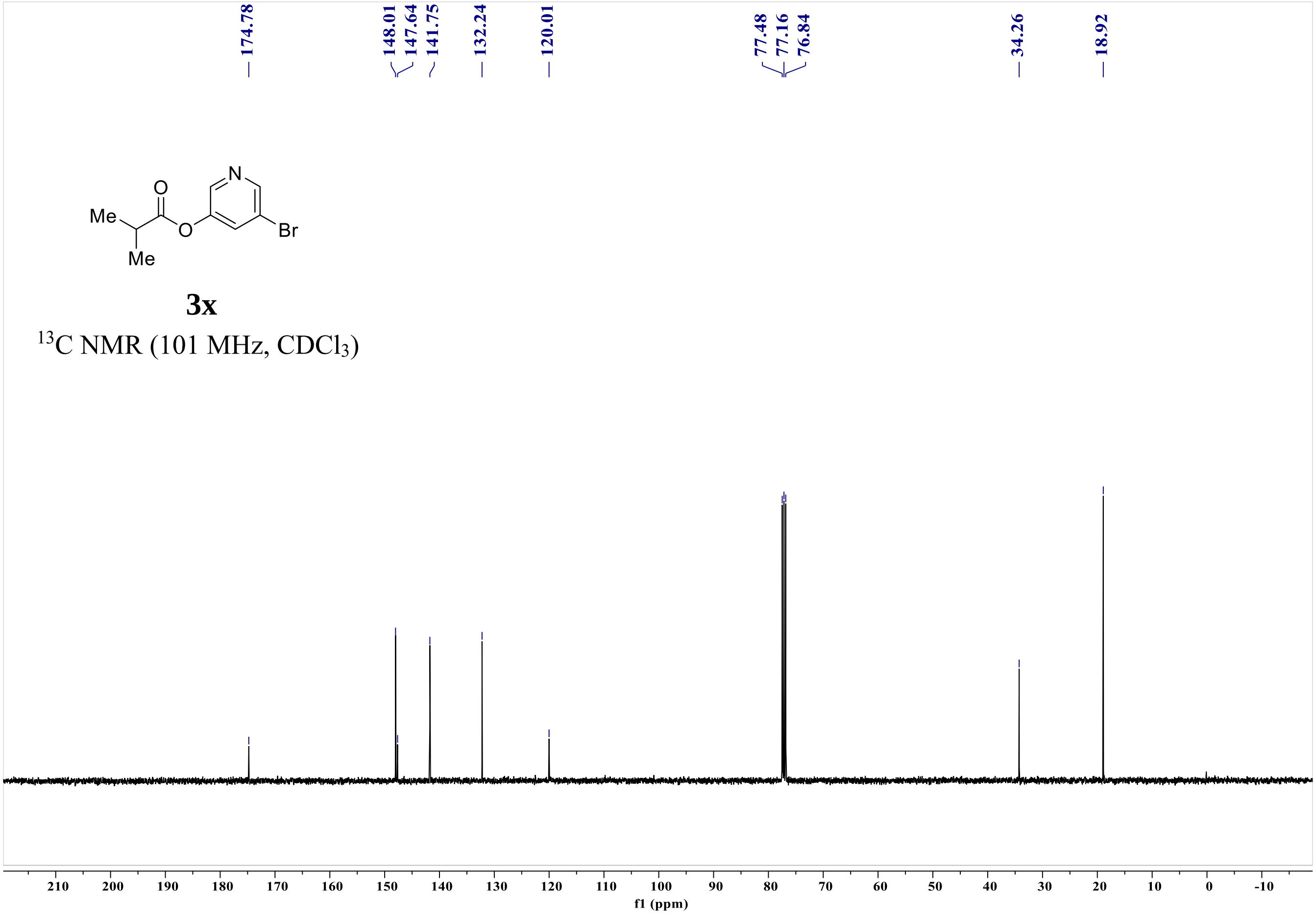

3x
^{13}C NMR (101 MHz, $CDCl_3$)

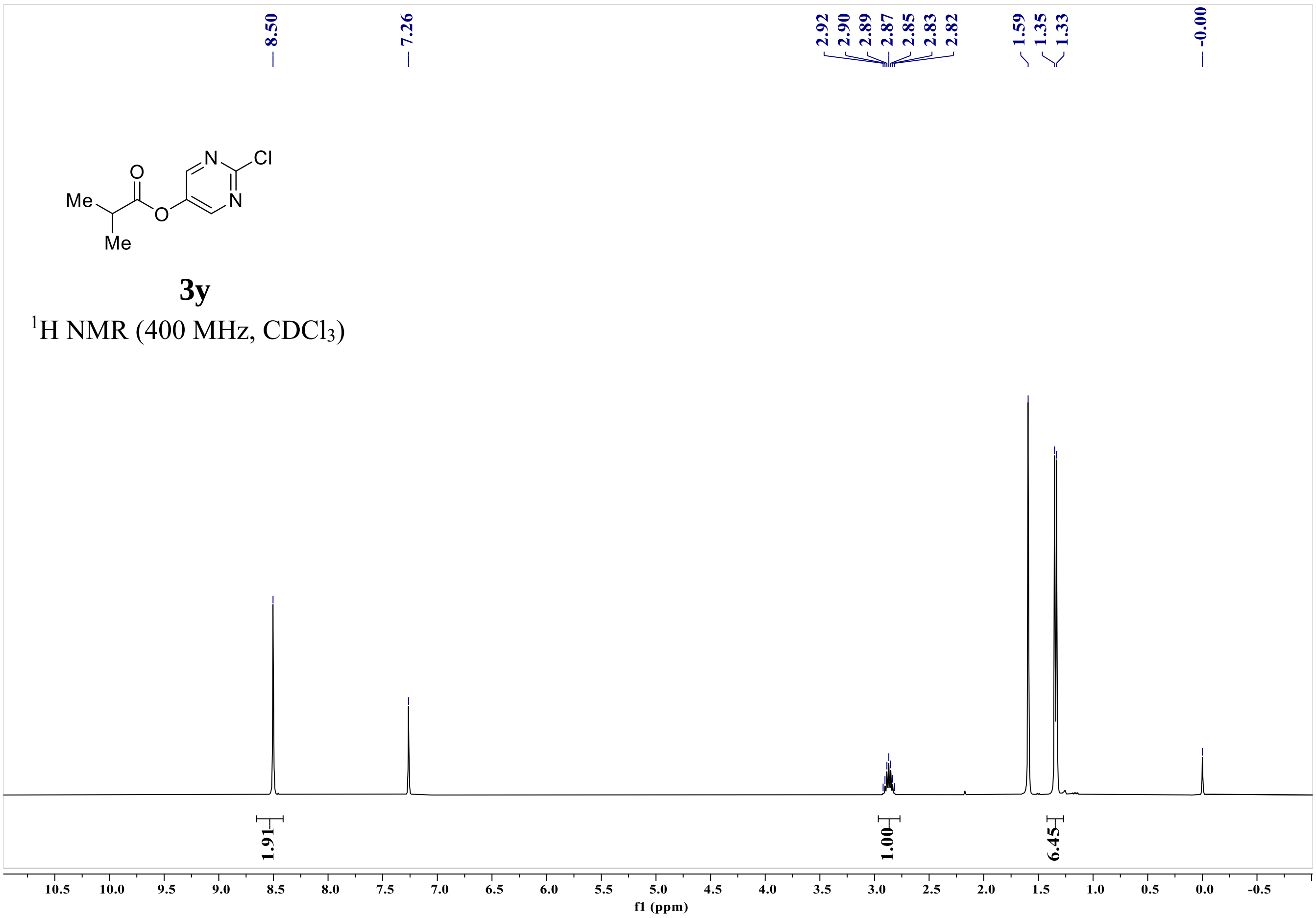

3y
^{1}H NMR (400 MHz, $CDCl_3$)

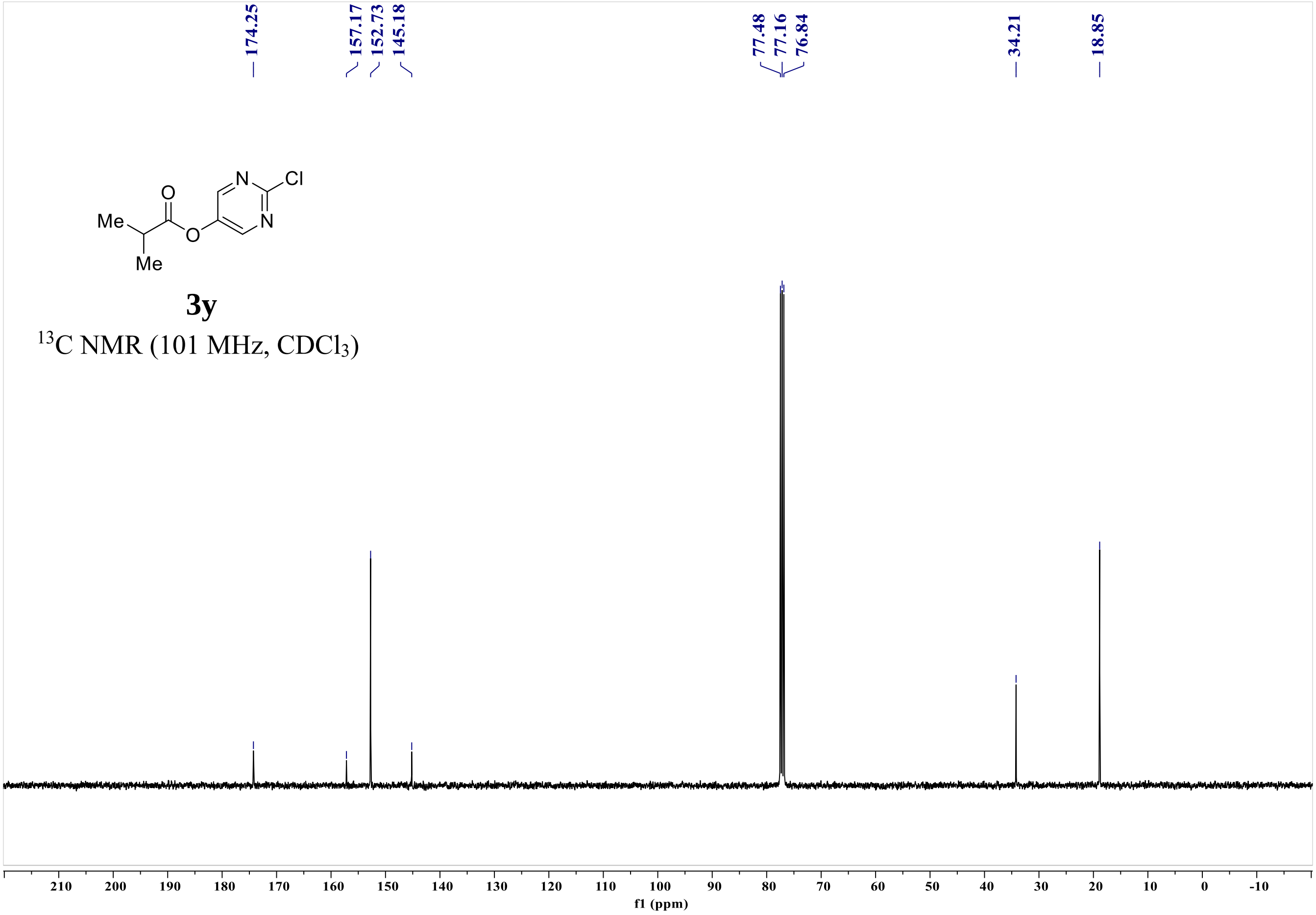
174.25
157.17
152.73
145.18
77.48
77.16
76.84
34.21
18.85
Me
O
O
N
N
Cl
Me
3y
13C NMR (101 MHz, CDCl3)
210 200 190 180 170 160 150 140 130 120 110 100 90 80 70 60 50 40 30 20 10 0 -10
f1 (ppm)

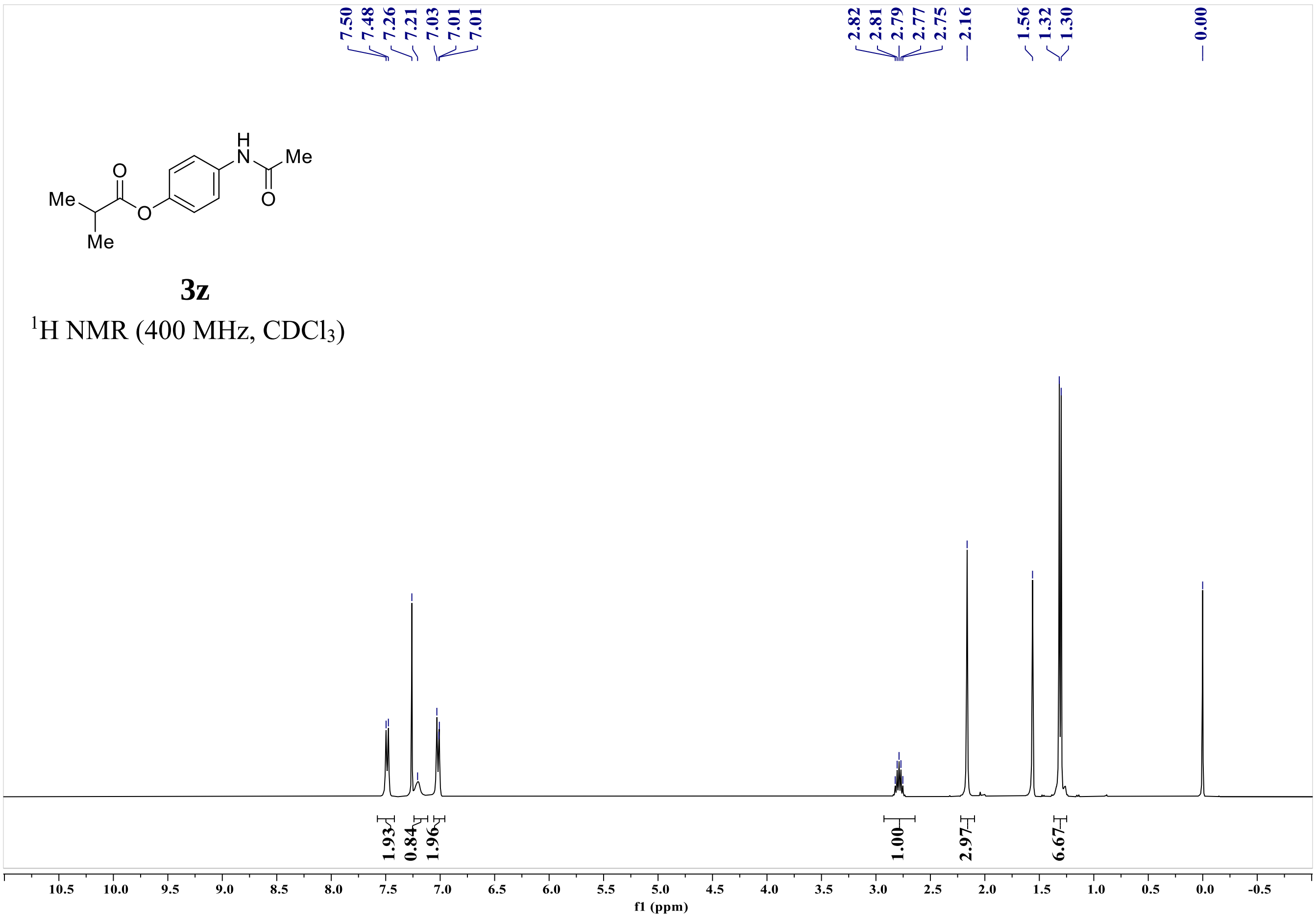
7.50
7.48
7.26
7.21
7.03
7.01
7.01
2.82
2.81
2.79
2.77
2.75
2.16
1.56
1.32
1.30
0.00
H
N
Me
O
Me
O
O
Me
3z
1H NMR (400 MHz, CDCl3)
1.93
0.84
1.96
1.00
2.97
6.67
10.5 10.0 9.5 9.0 8.5 8.0 7.5 7.0 6.5 6.0 5.5 5.0 4.5 4.0 3.5 3.0 2.5 2.0 1.5 1.0 0.5 0.0 -0.5
f1 (ppm)

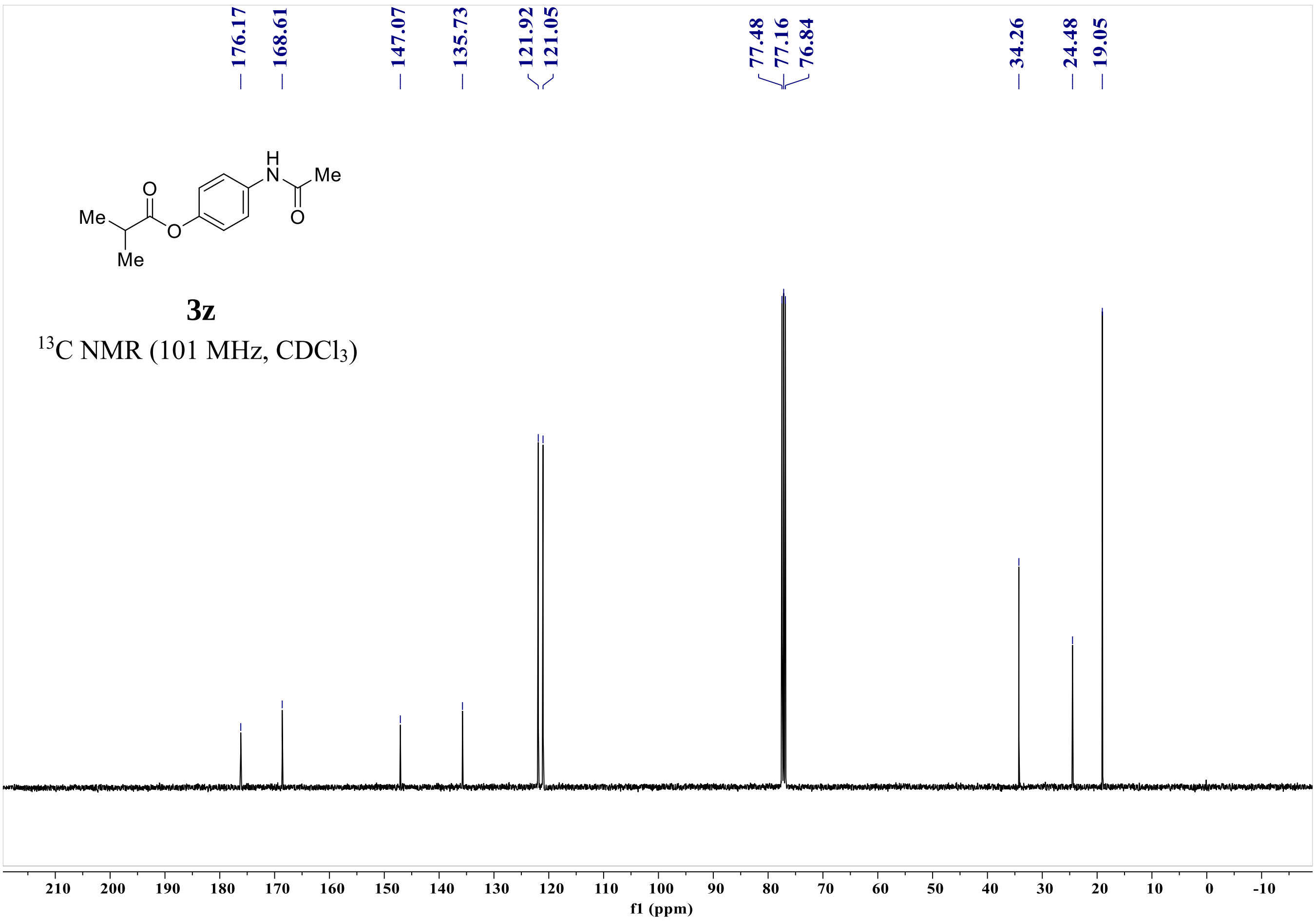
176.17
168.61
147.07
135.73
121.92
121.05
77.48
77.16
76.84
34.26
24.48
19.05
Me
Me
O
O
H
N
Me
O
3z
13C NMR (101 MHz, CDCl3)
210 200 190 180 170 160 150 140 130 120 110 100 90 80 70 60 50 40 30 20 10 0 -10
f1 (ppm)

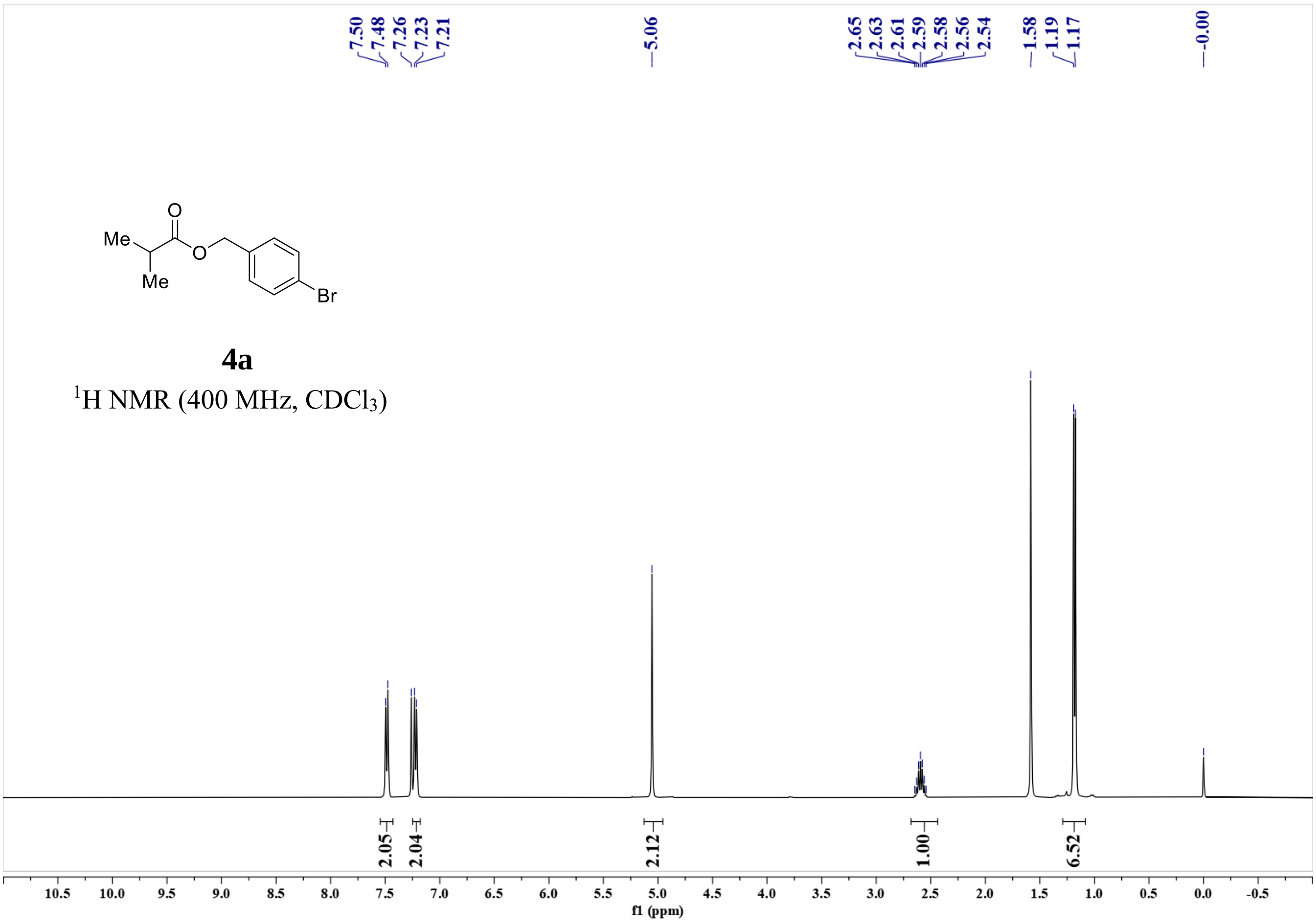
7.50
7.48
7.26
7.23
7.21
5.06
2.65
2.63
2.61
2.59
2.58
2.56
2.54
1.58
1.19
1.17
-0.00
Me
Me
O
O
Br
4a
1H NMR (400 MHz, CDCl3)
2.05
2.04
2.12
1.00
6.52
10.5 10.0 9.5 9.0 8.5 8.0 7.5 7.0 6.5 6.0 5.5 5.0 4.5 4.0 3.5 3.0 2.5 2.0 1.5 1.0 0.5 0.0 -0.5
f1 (ppm)

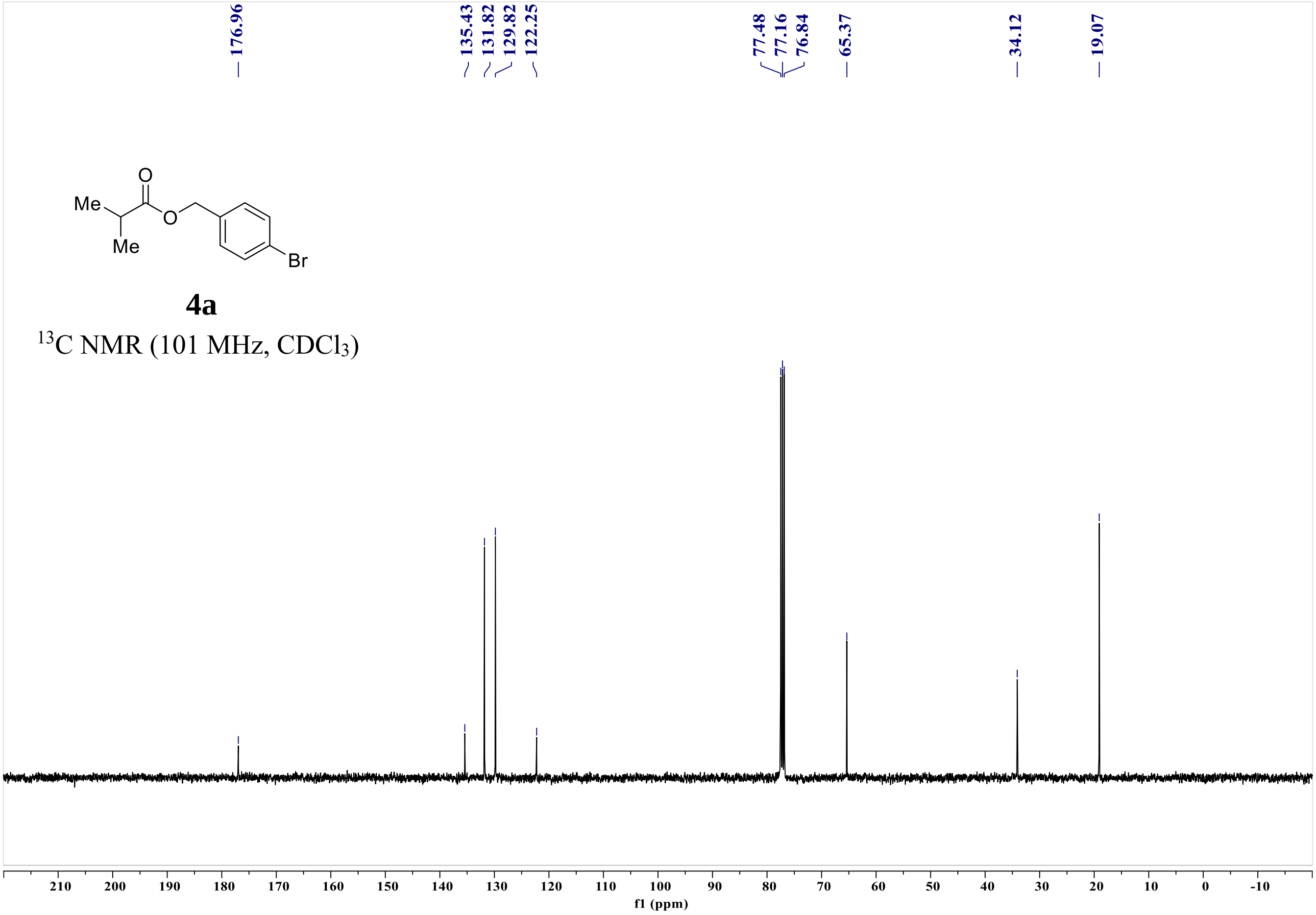

4a

^{13}C NMR (101 MHz, $CDCl_3$)

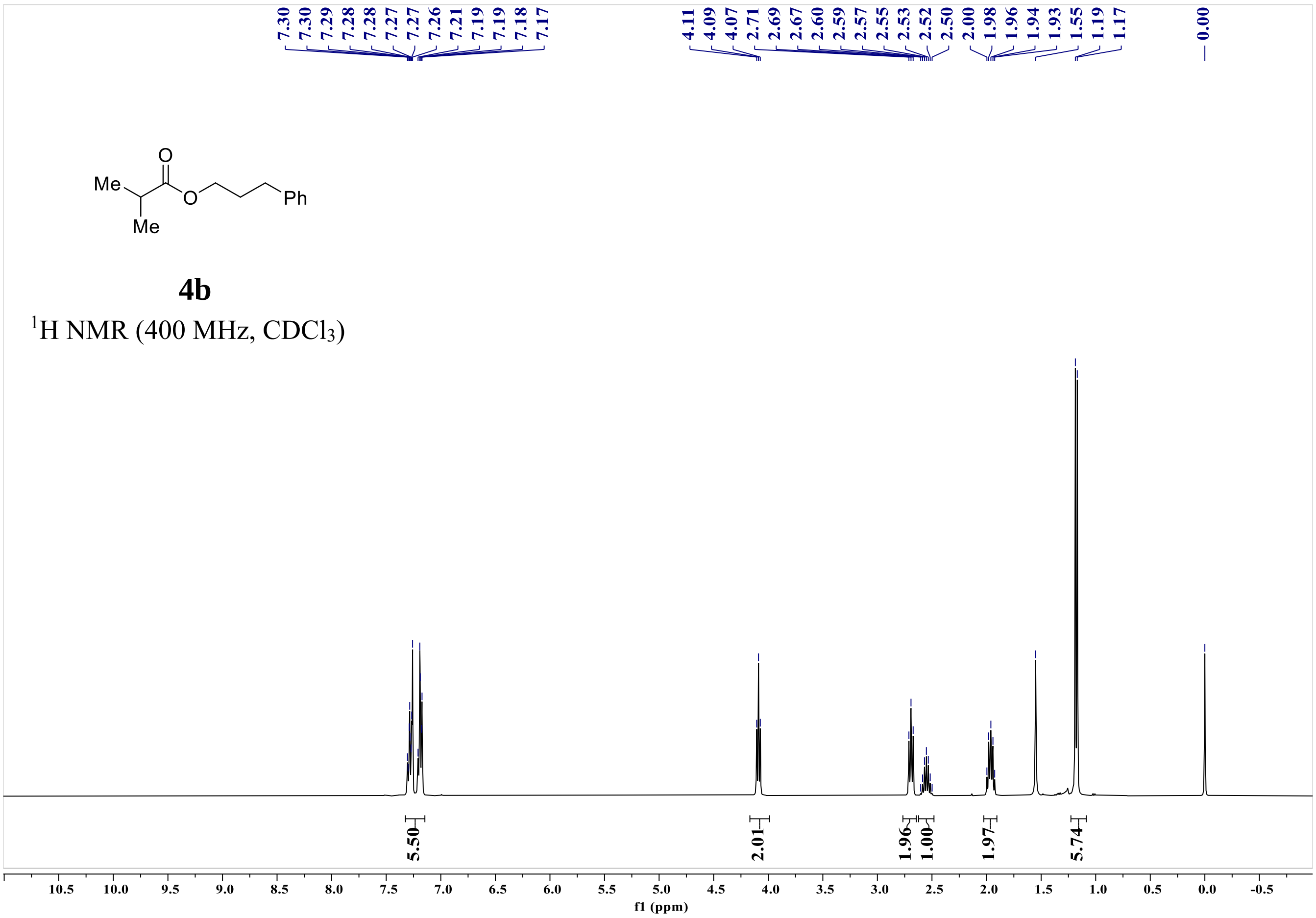

4b

^{1}H NMR (400 MHz, $CDCl_3$)

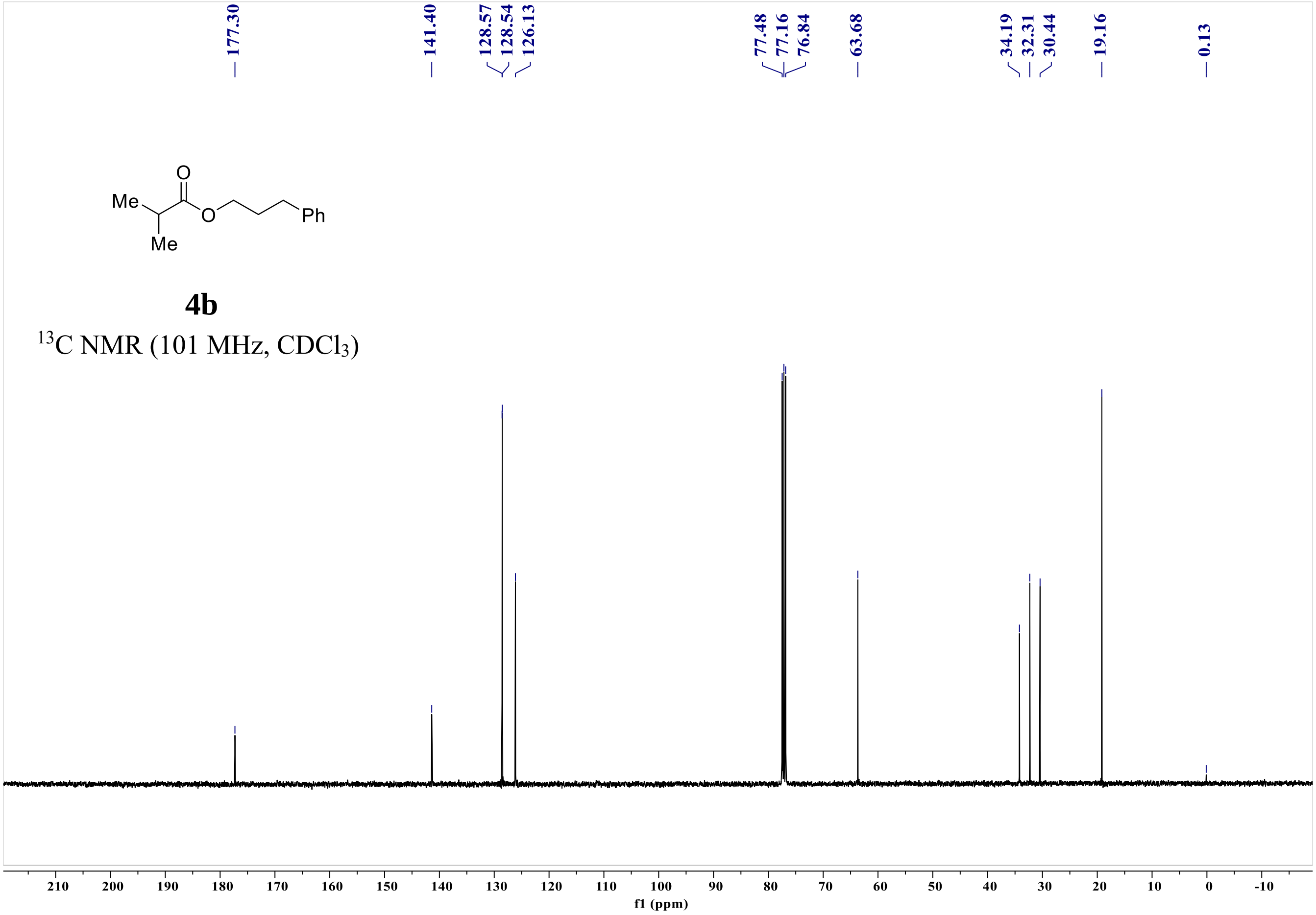

4b

^{13}C NMR (101 MHz, $CDCl_3$)

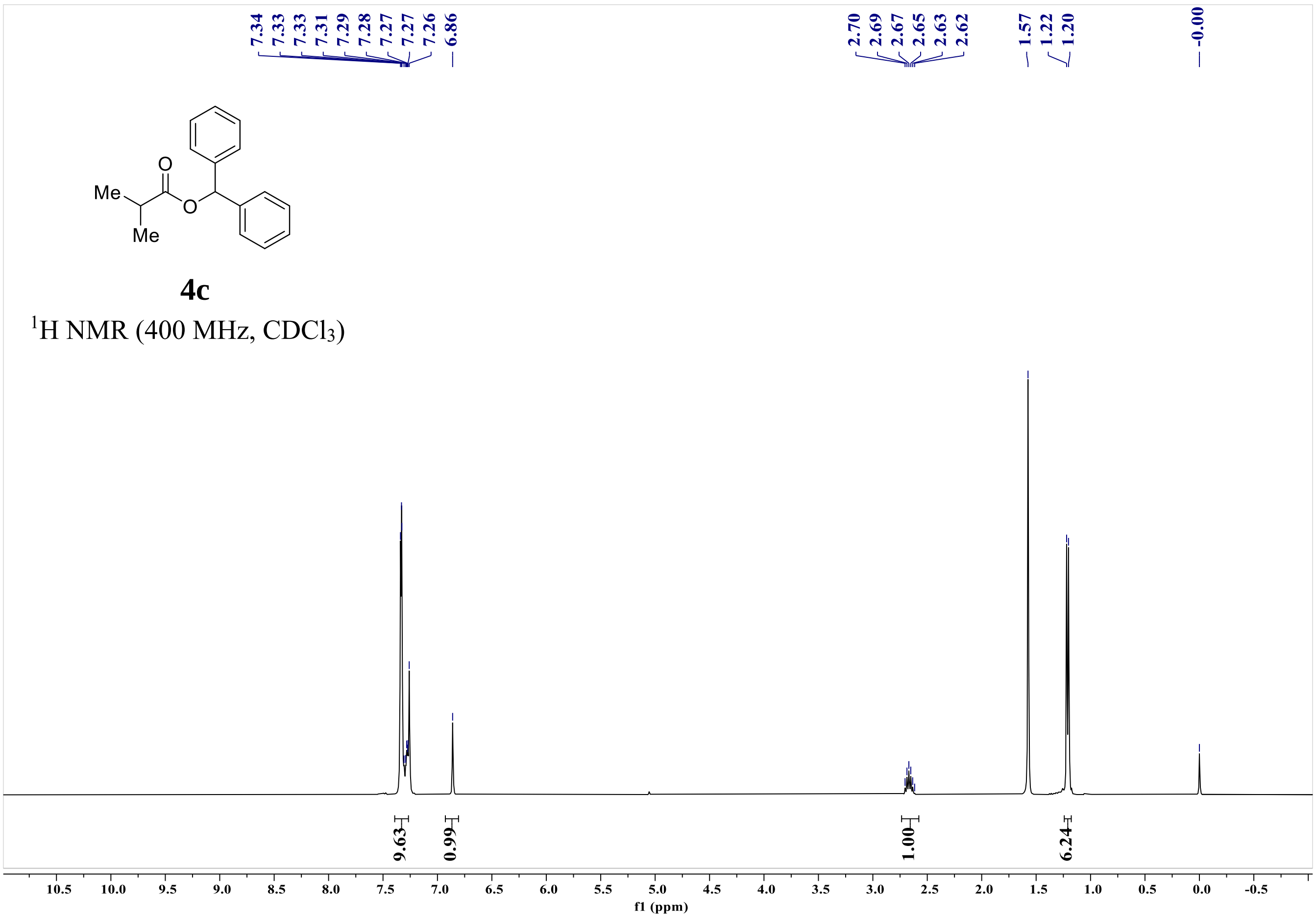

4c

^{1}H NMR (400 MHz, $CDCl_3$)

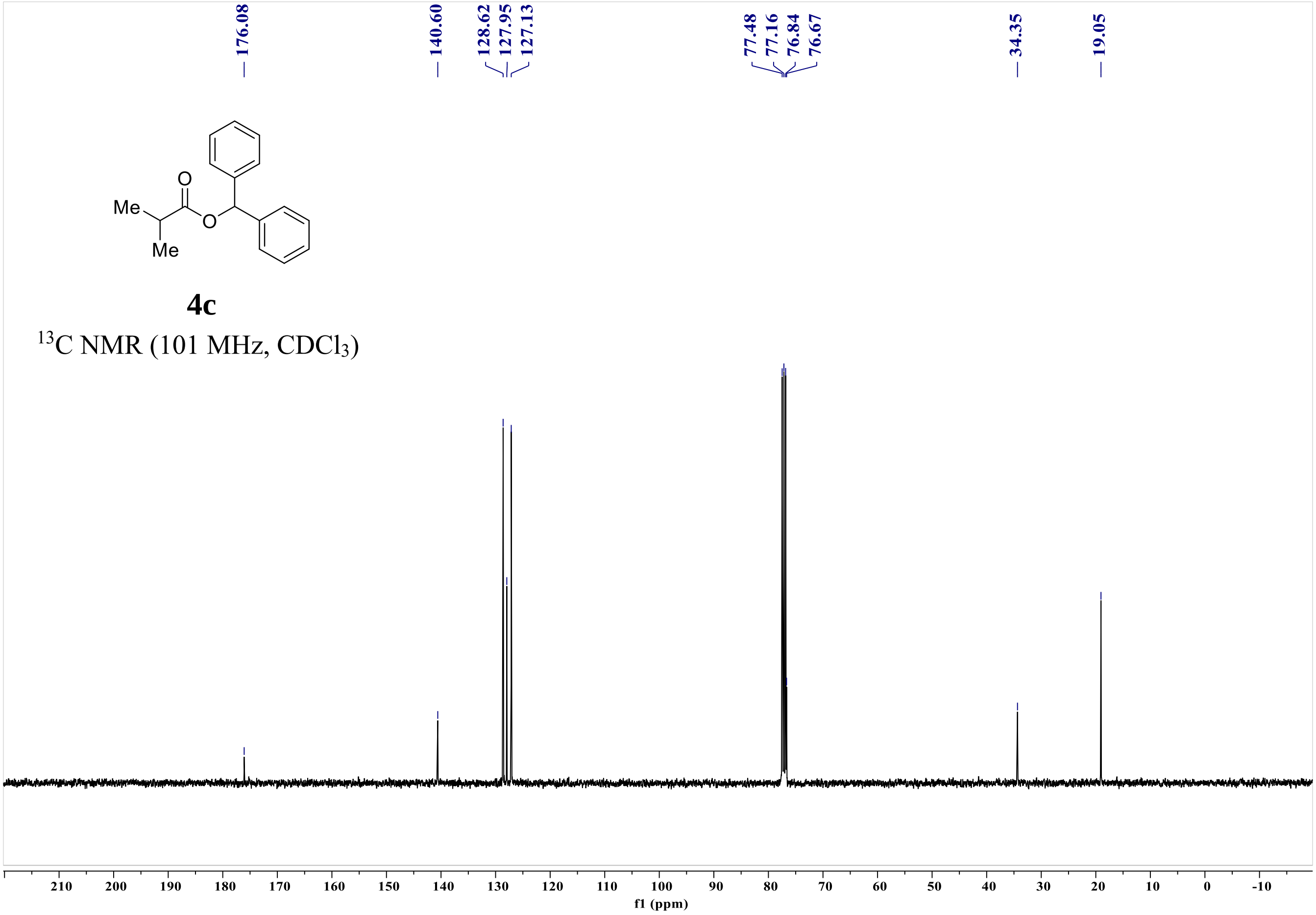
176.08
140.60
128.62
127.95
127.13
77.48
77.16
76.84
76.67
34.35
19.05
Me
Me
O
O
4c
13C NMR (101 MHz, CDCl3)
f1 (ppm)

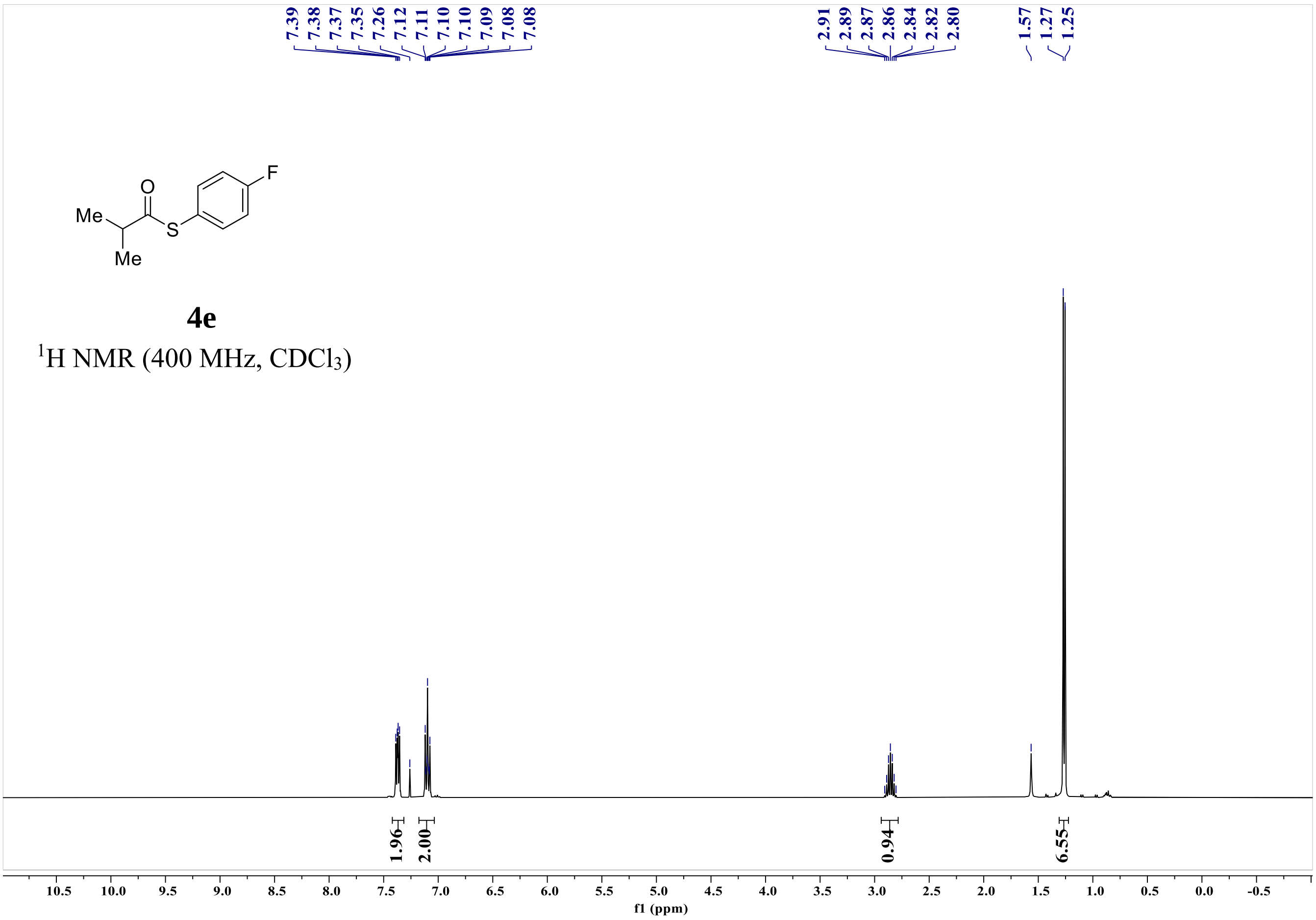
7.39
7.38
7.37
7.35
7.26
7.12
7.11
7.10
7.10
7.09
7.08
7.08
2.91
2.89
2.87
2.86
2.84
2.82
2.80
1.57
1.27
1.25
Me
Me
O
S
F
4e
1H NMR (400 MHz, CDCl3)
1.96
2.00
0.94
6.55
f1 (ppm)

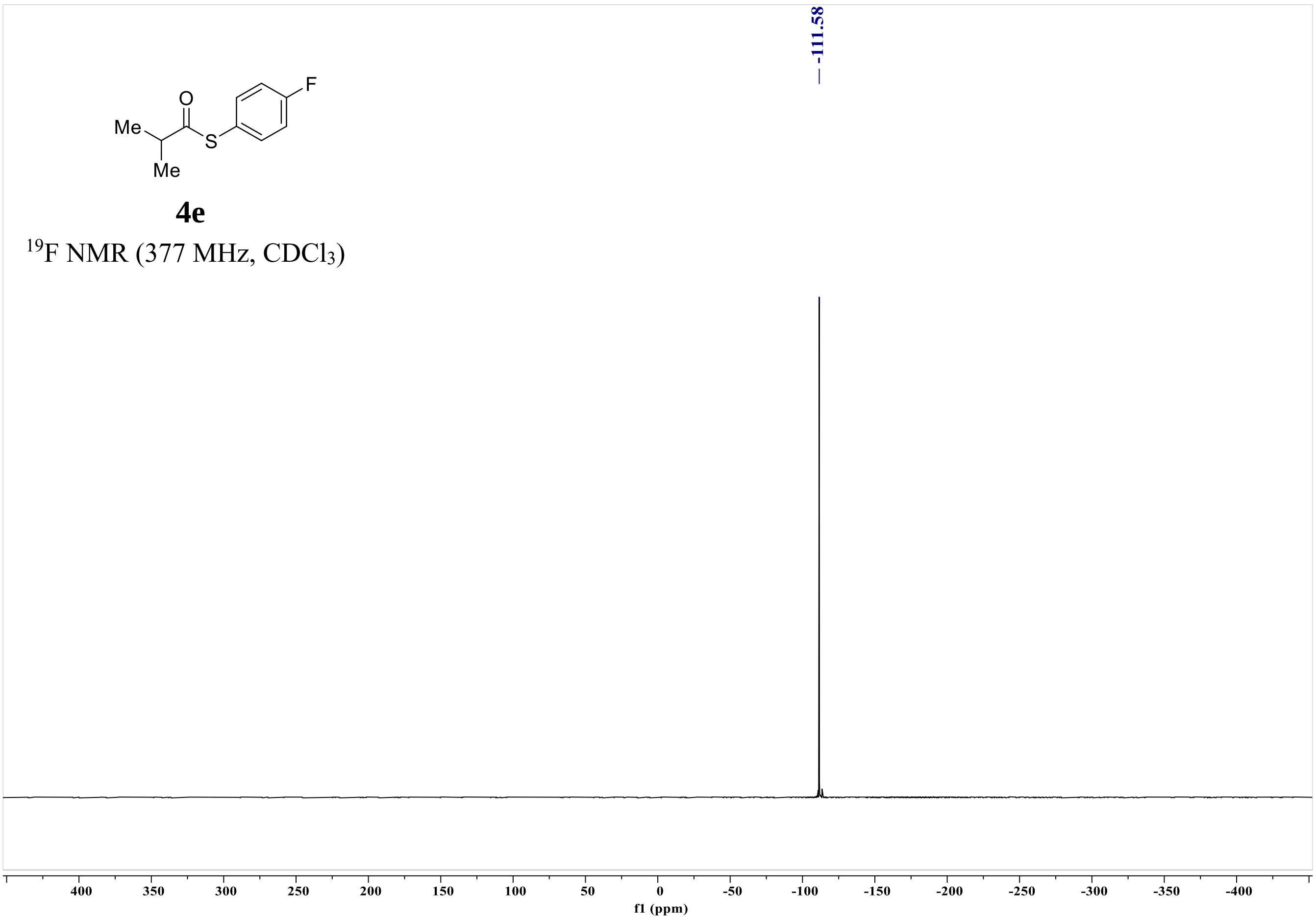
-111.58
O
F
Me
S
Me
4e
19F NMR (377 MHz, CDCl3)
400 350 300 250 200 150 100 50 0 -50 -100 -150 -200 -250 -300 -350 -400
f1 (ppm)

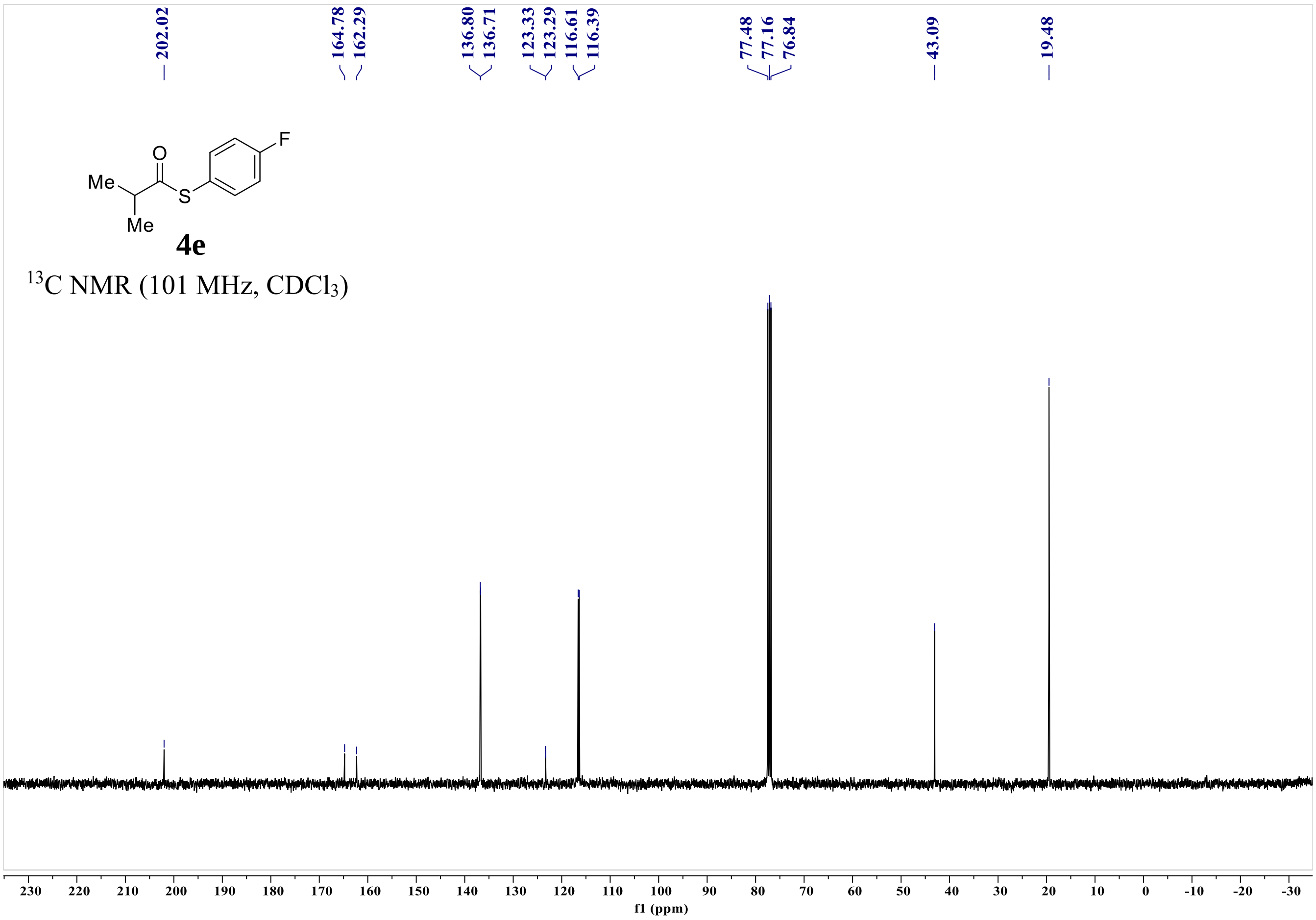
202.02
164.78
162.29
136.80
136.71
123.33
123.29
116.61
116.39
77.48
77.16
76.84
43.09
19.48
O
F
Me
S
Me
4e
13C NMR (101 MHz, CDCl3)
230 220 210 200 190 180 170 160 150 140 130 120 110 100 90 80 70 60 50 40 30 20 10 0 -10 -20 -30
f1 (ppm)

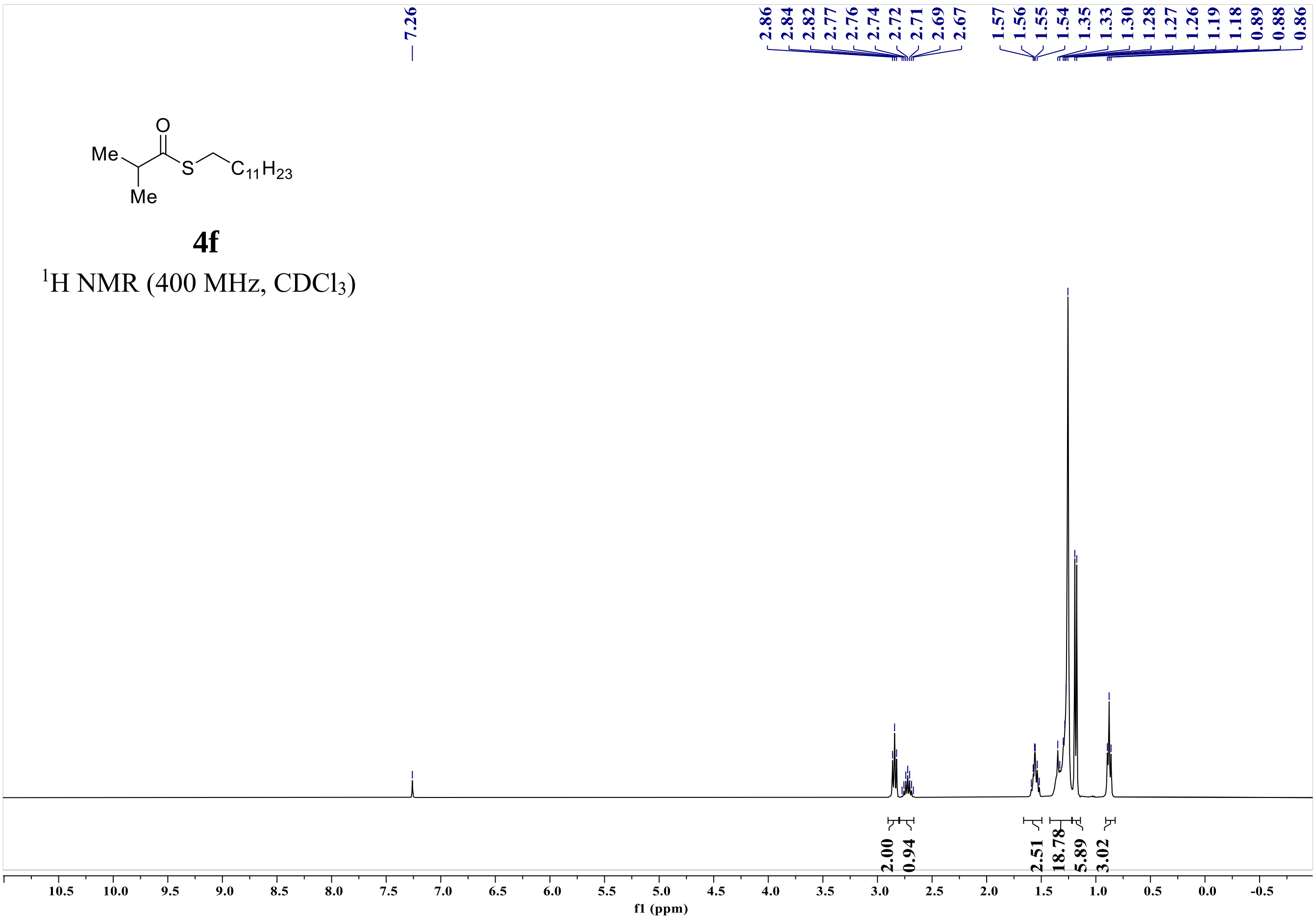

7.26
2.86
2.84
2.82
2.77
2.76
2.74
2.72
2.71
2.69
2.67
1.57
1.56
1.55
1.54
1.35
1.33
1.30
1.28
1.27
1.26
1.19
1.18
0.89
0.88
0.86
Me
O
S
C11H23
Me
4f
1H NMR (400 MHz, CDCl3)
2.00
0.94
2.51
18.78
5.89
3.02
f1 (ppm)

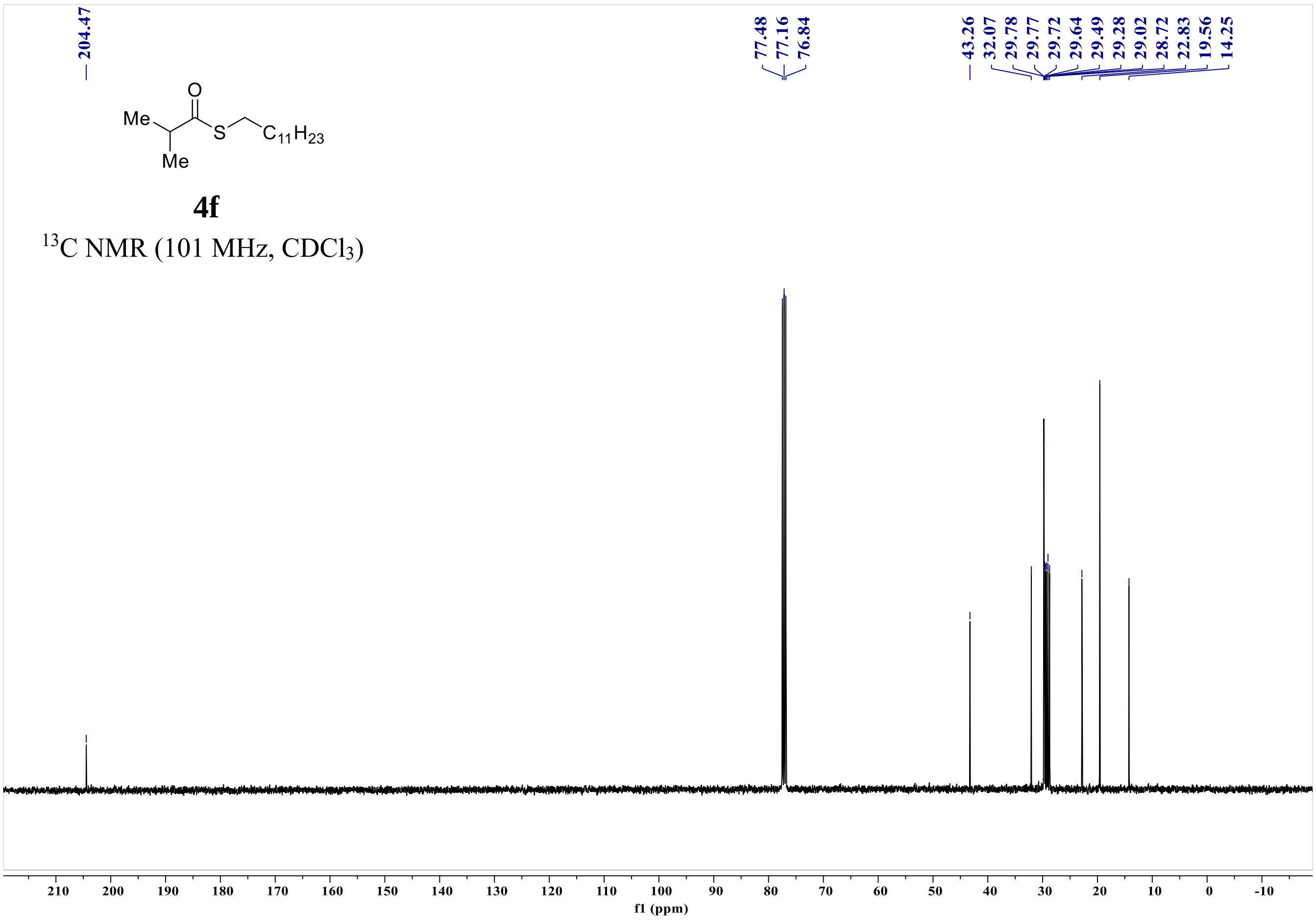

204.47
77.48
77.16
76.84
43.26
32.07
29.78
29.77
29.72
29.64
29.49
29.28
29.02
28.72
22.83
19.56
14.25
Me
O
S
C11H23
Me
4f
13C NMR (101 MHz, CDCl3)
f1 (ppm)

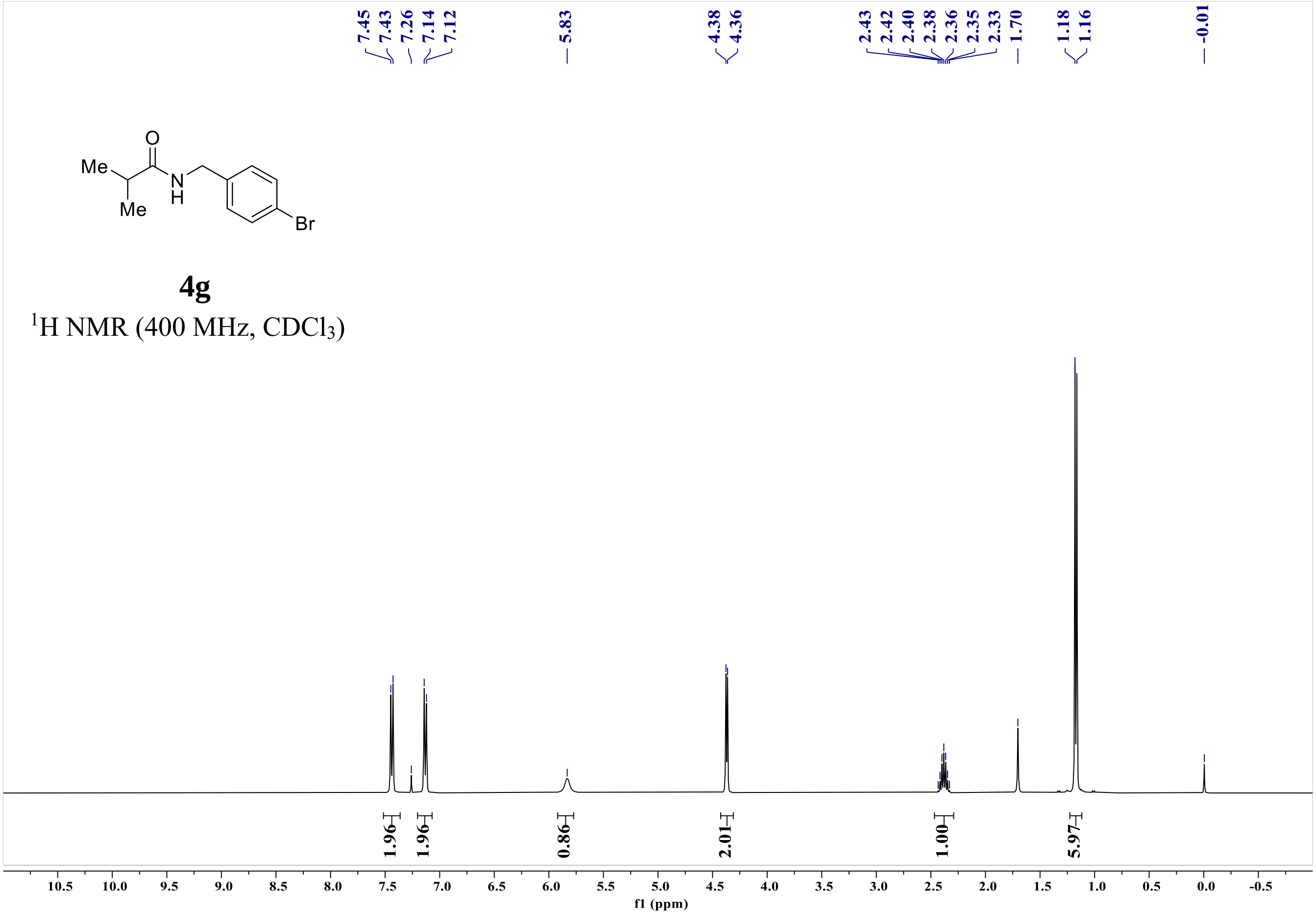

4g

^{1}H NMR (400 MHz, $CDCl_3$)

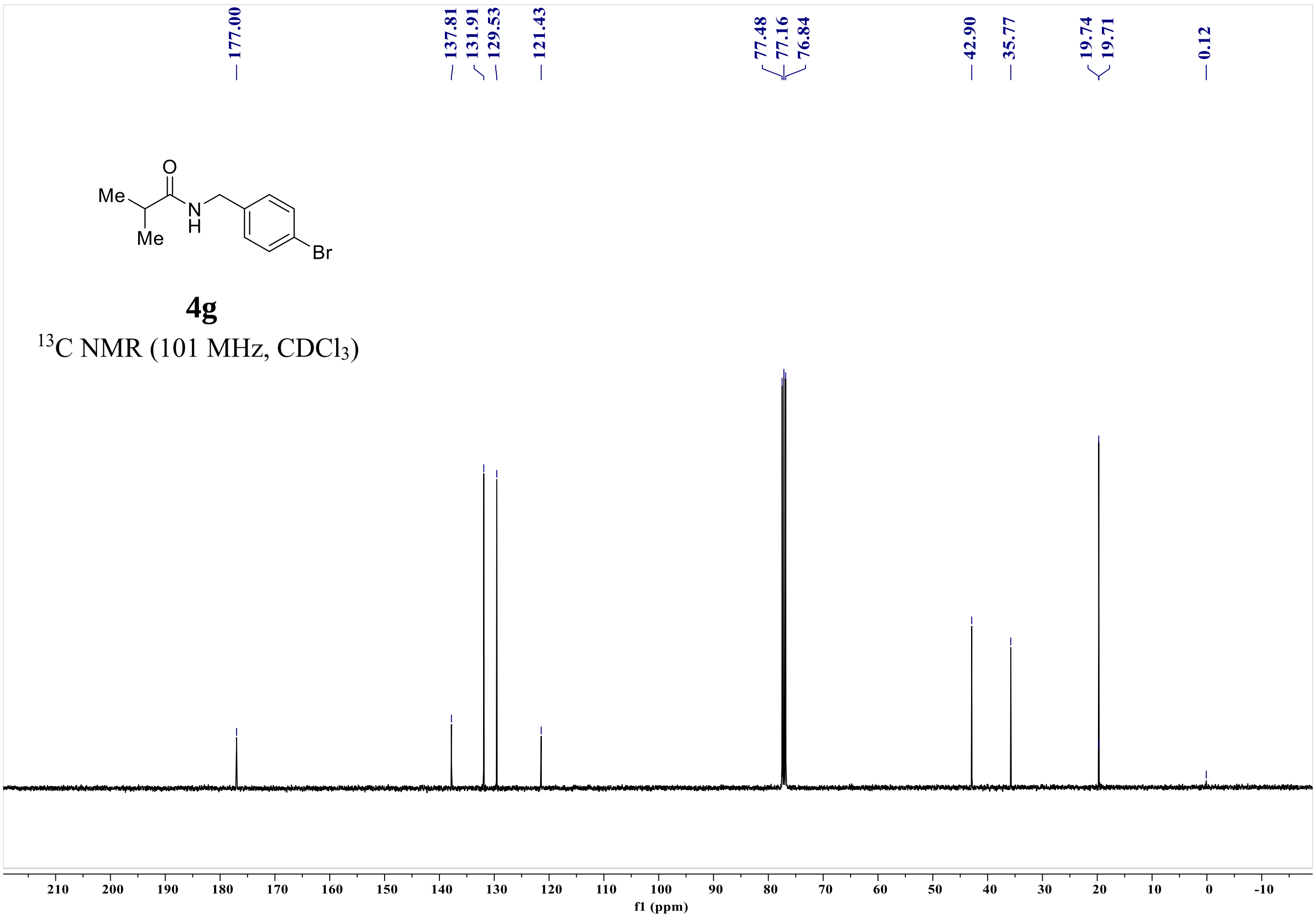

4g

^{13}C NMR (101 MHz, $CDCl_3$)

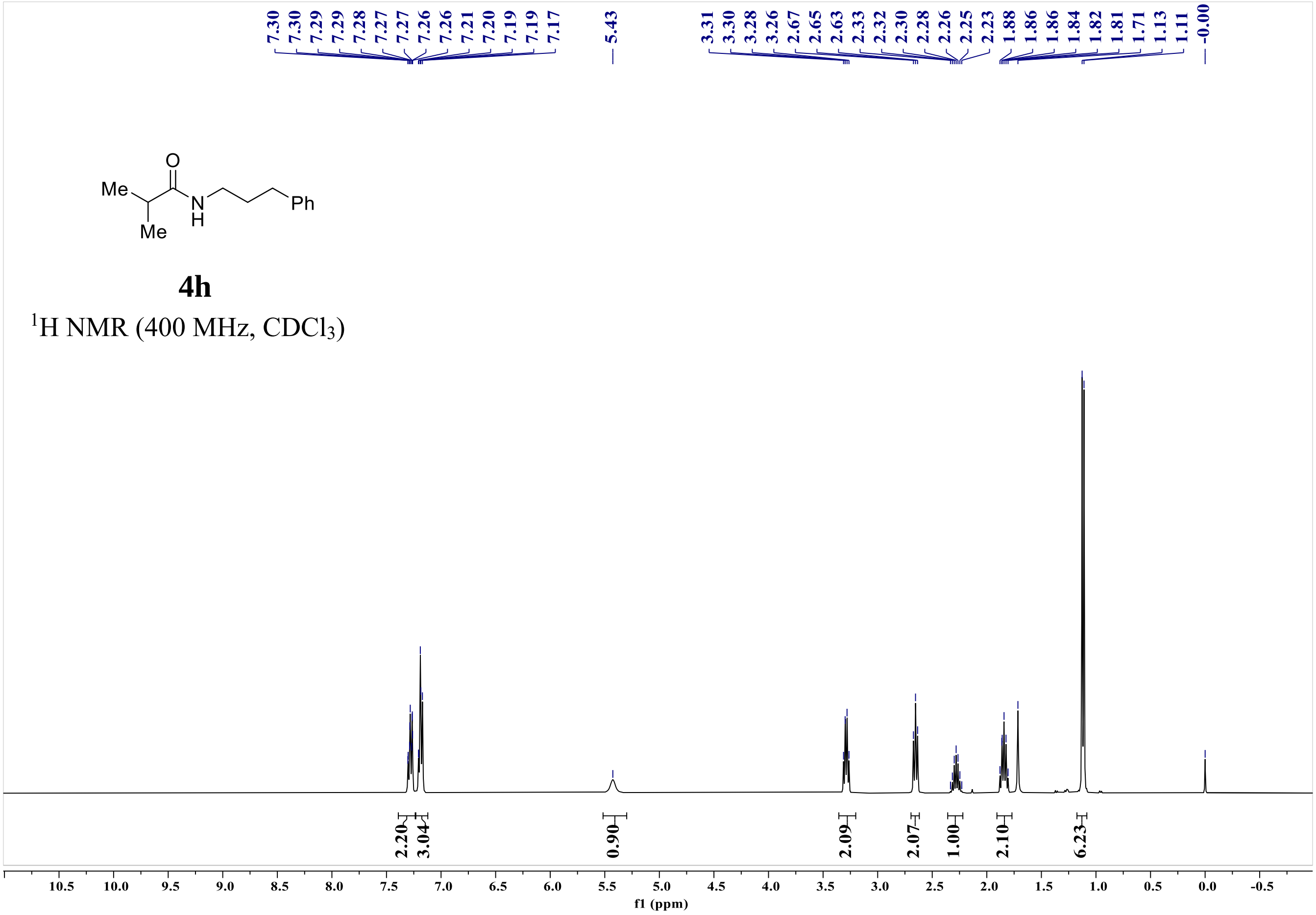
7.30
7.30
7.29
7.29
7.28
7.27
7.27
7.26
7.26
7.21
7.20
7.19
7.19
7.17
5.43
3.31
3.30
3.28
3.26
2.67
2.65
2.63
2.33
2.32
2.30
2.28
2.26
2.25
2.23
1.88
1.86
1.86
1.84
1.82
1.81
1.71
1.13
1.11
-0.00
Me
O
N
H
Ph
Me
4h
^{1}H NMR (400 MHz, CDCl3)
2.20
3.04
0.90
2.09
2.07
1.00
2.10
6.23
f1 (ppm)

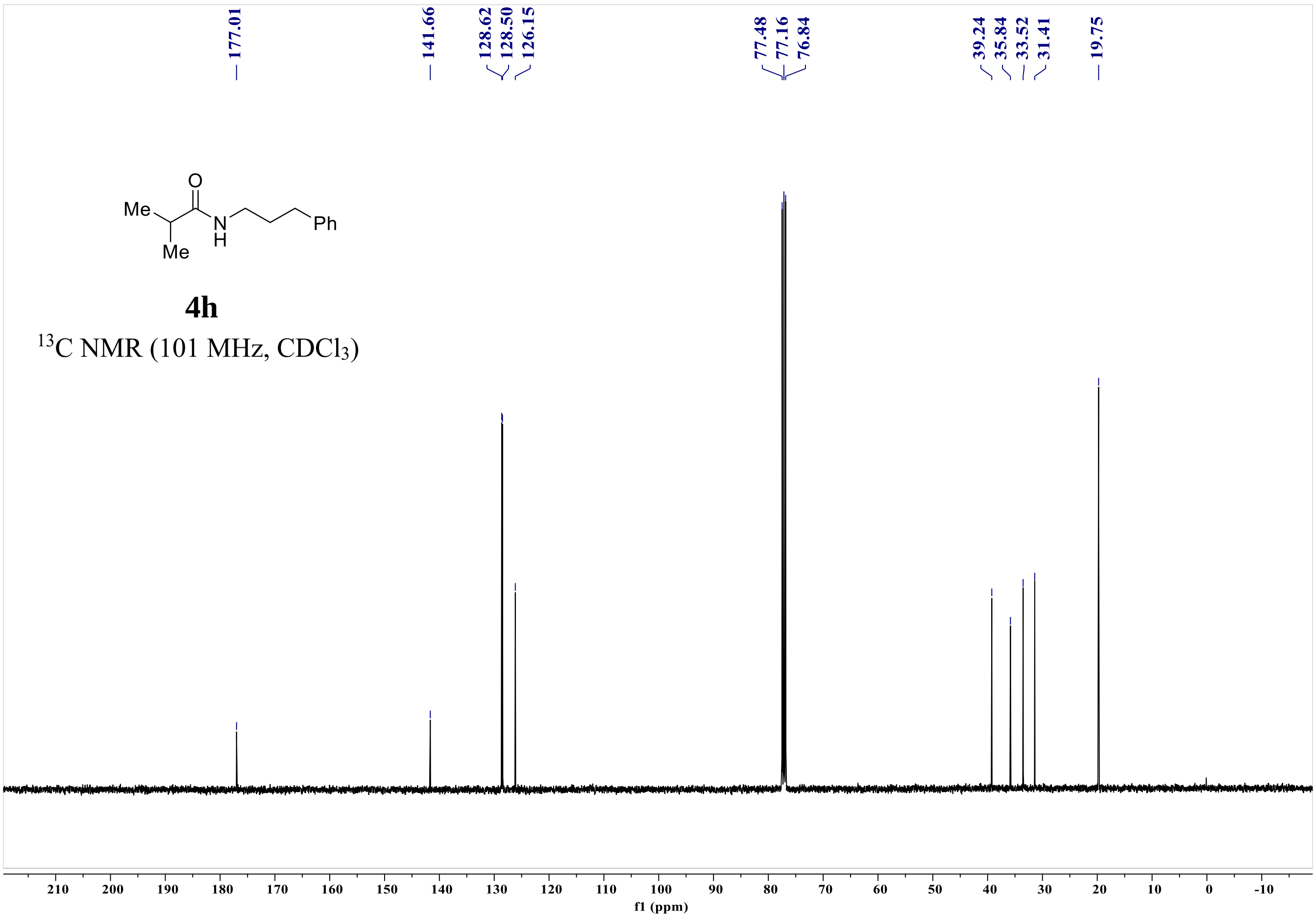
177.01
141.66
128.62
128.50
126.15
77.48
77.16
76.84
39.24
35.84
33.52
31.41
19.75
Me
O
N
H
Ph
Me
4h
^{13}C NMR (101 MHz, CDCl3)
f1 (ppm)

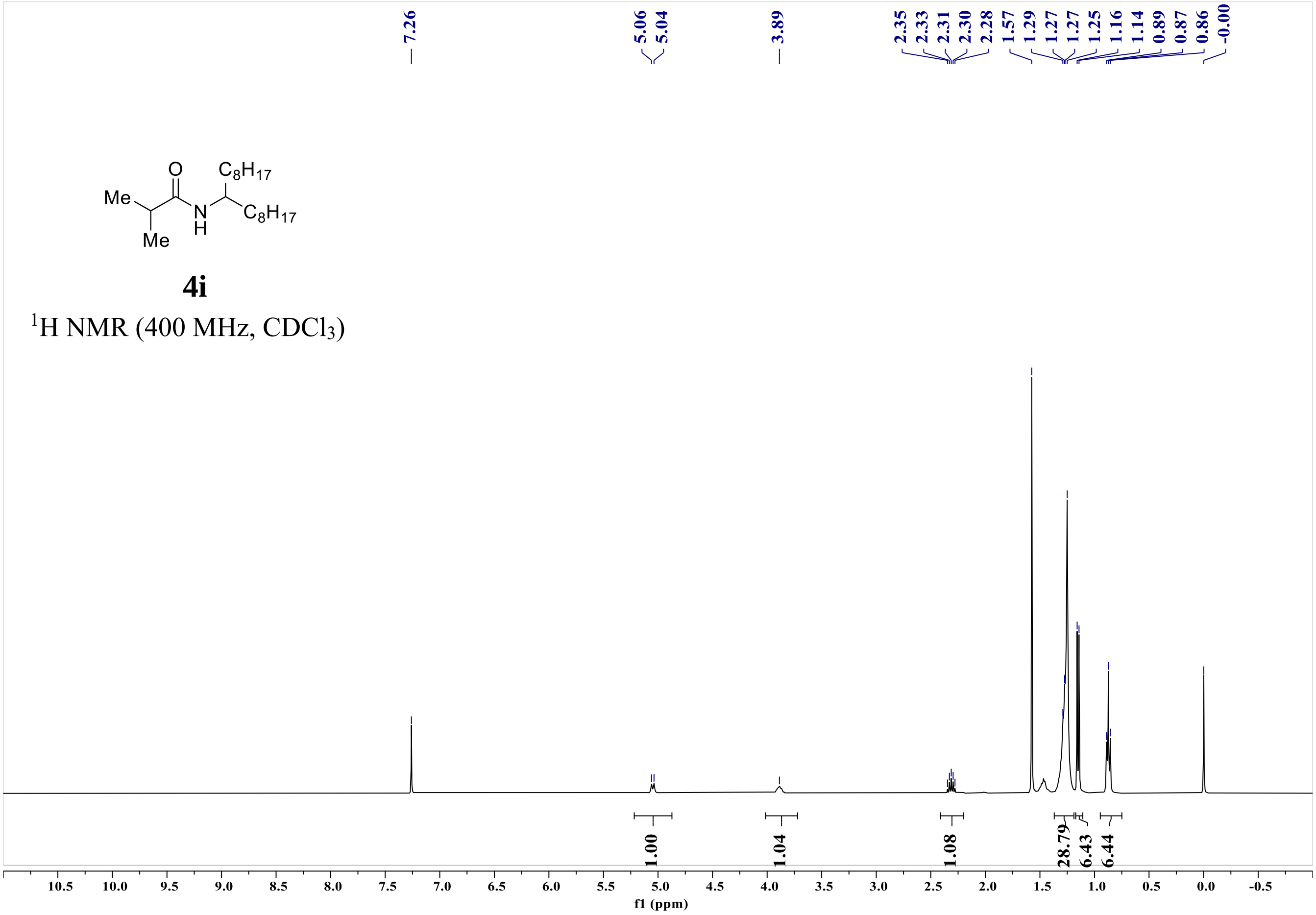
7.26
5.06
5.04
3.89
2.35
2.33
2.31
2.30
2.28
1.57
1.29
1.27
1.27
1.25
1.16
1.14
0.89
0.87
0.86
-0.00
Me
O
N
H
Me
C8H17
C8H17
4i
1H NMR (400 MHz, CDCl3)
1.00
1.04
1.08
28.79
6.43
6.44
10.5
10.0
9.5
9.0
8.5
8.0
7.5
7.0
6.5
6.0
5.5
5.0
4.5
4.0
3.5
3.0
2.5
2.0
1.5
1.0
0.5
0.0
-0.5
f1 (ppm)

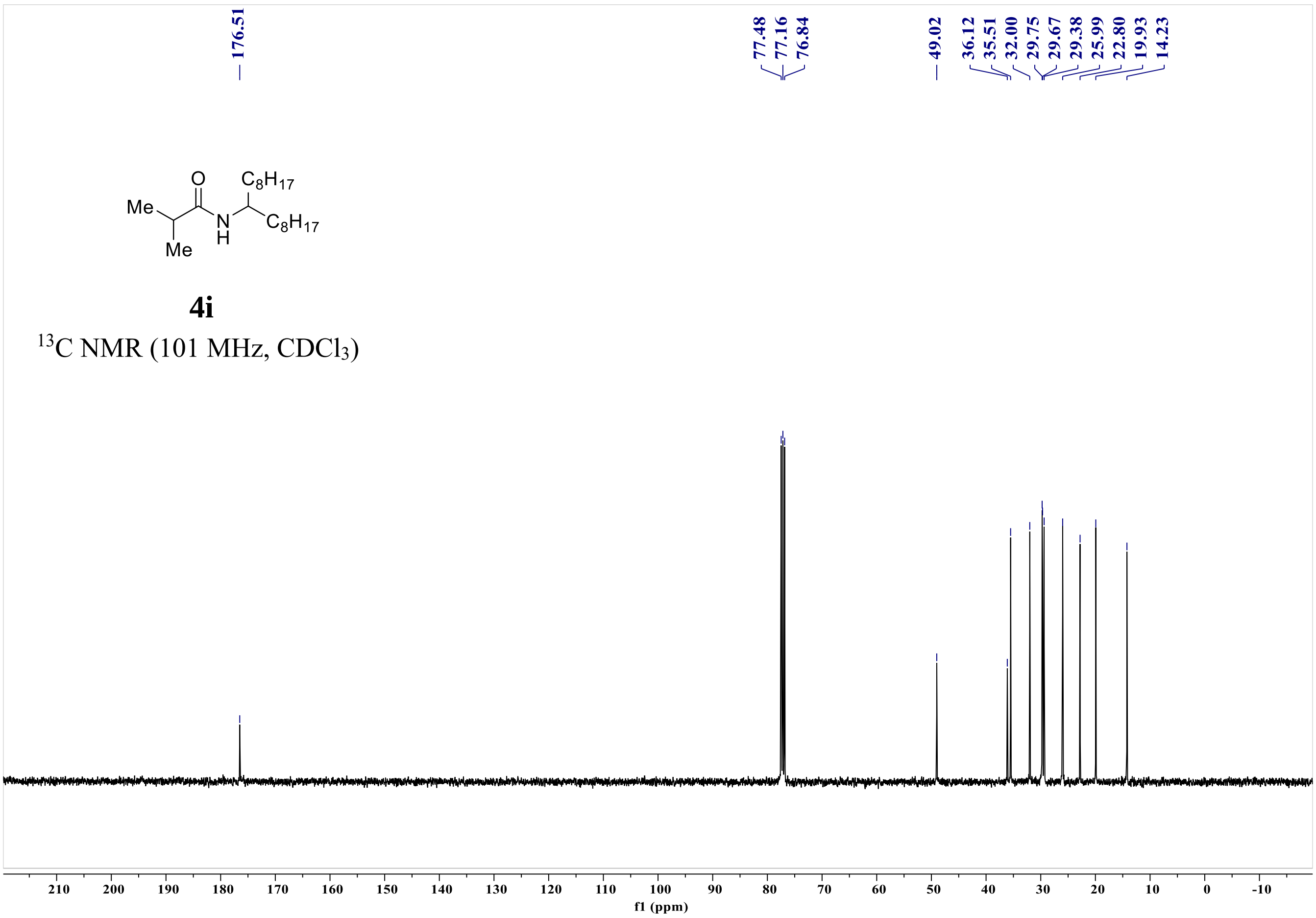
176.51
77.48
77.16
76.84
49.02
36.12
35.51
32.00
29.75
29.67
29.38
25.99
22.80
19.93
14.23
Me
O
N
H
Me
C8H17
C8H17
4i
13C NMR (101 MHz, CDCl3)
210
200
190
180
170
160
150
140
130
120
110
100
90
80
70
60
50
40
30
20
10
0
-10
f1 (ppm)

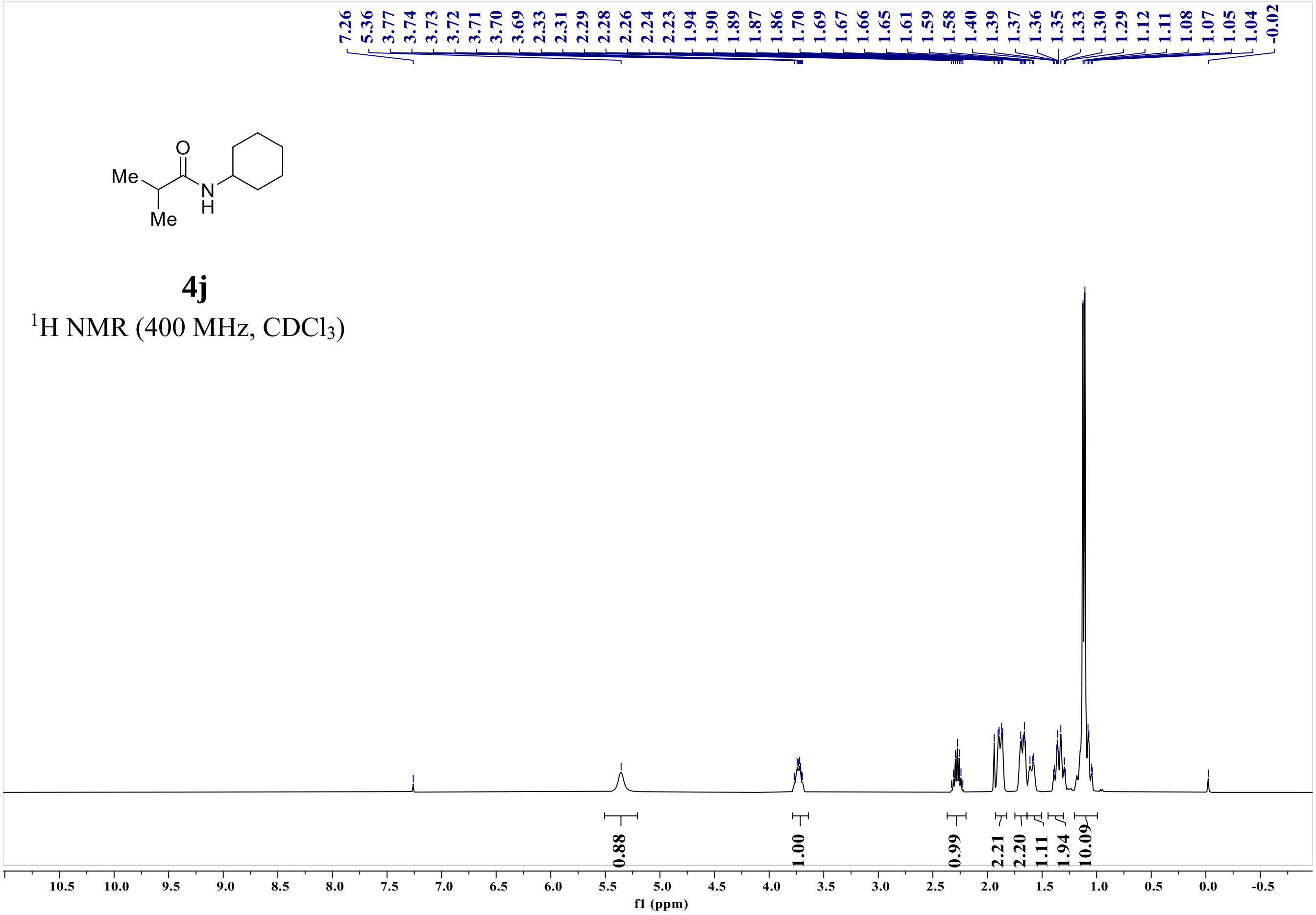
7.26 5.36 3.77 3.74 3.73 3.72 3.71 3.70 3.69 2.33 2.31 2.29 2.28 2.26 2.24 2.23 1.94 1.90 1.89 1.87 1.86 1.70 1.69 1.67 1.66 1.65 1.61 1.59 1.58 1.40 1.39 1.37 1.36 1.35 1.33 1.30 1.29 1.12 1.11 1.08 1.07 1.05 1.04 -0.02
O
Me
N
H
Me
4j
^{1}H NMR (400 MHz, $CDCl_3$)
0.88 1.00 0.99 2.21 2.20 1.11 1.94 10.09
10.5 10.0 9.5 9.0 8.5 8.0 7.5 7.0 6.5 6.0 5.5 5.0 4.5 4.0 3.5 3.0 2.5 2.0 1.5 1.0 0.5 0.0 -0.5
f1 (ppm)

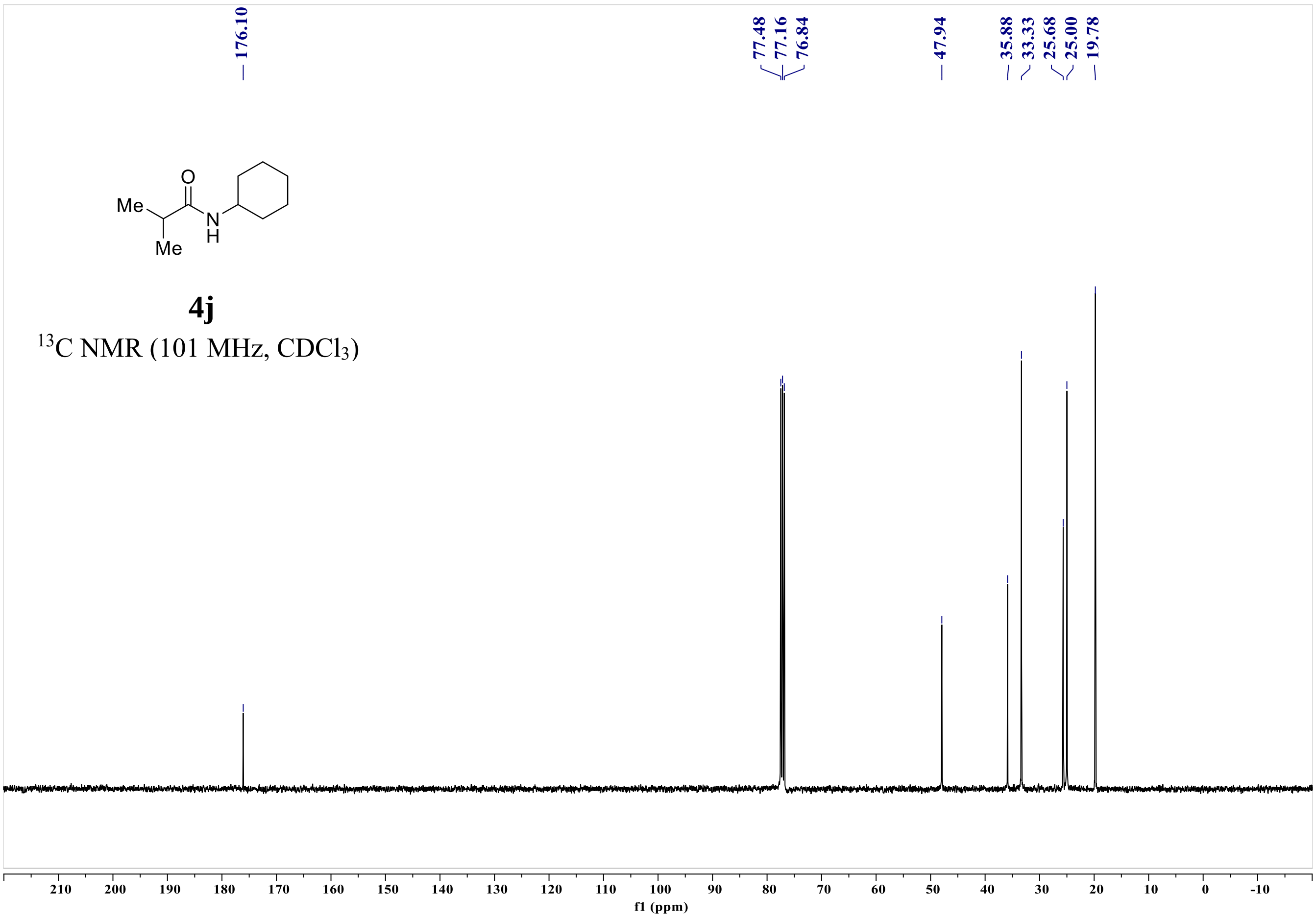
176.10
77.48 77.16 76.84
47.94
35.88 33.33 25.68 25.00 19.78
O
Me
N
H
Me
4j
^{13}C NMR (101 MHz, $CDCl_3$)
210 200 190 180 170 160 150 140 130 120 110 100 90 80 70 60 50 40 30 20 10 0 -10
f1 (ppm)

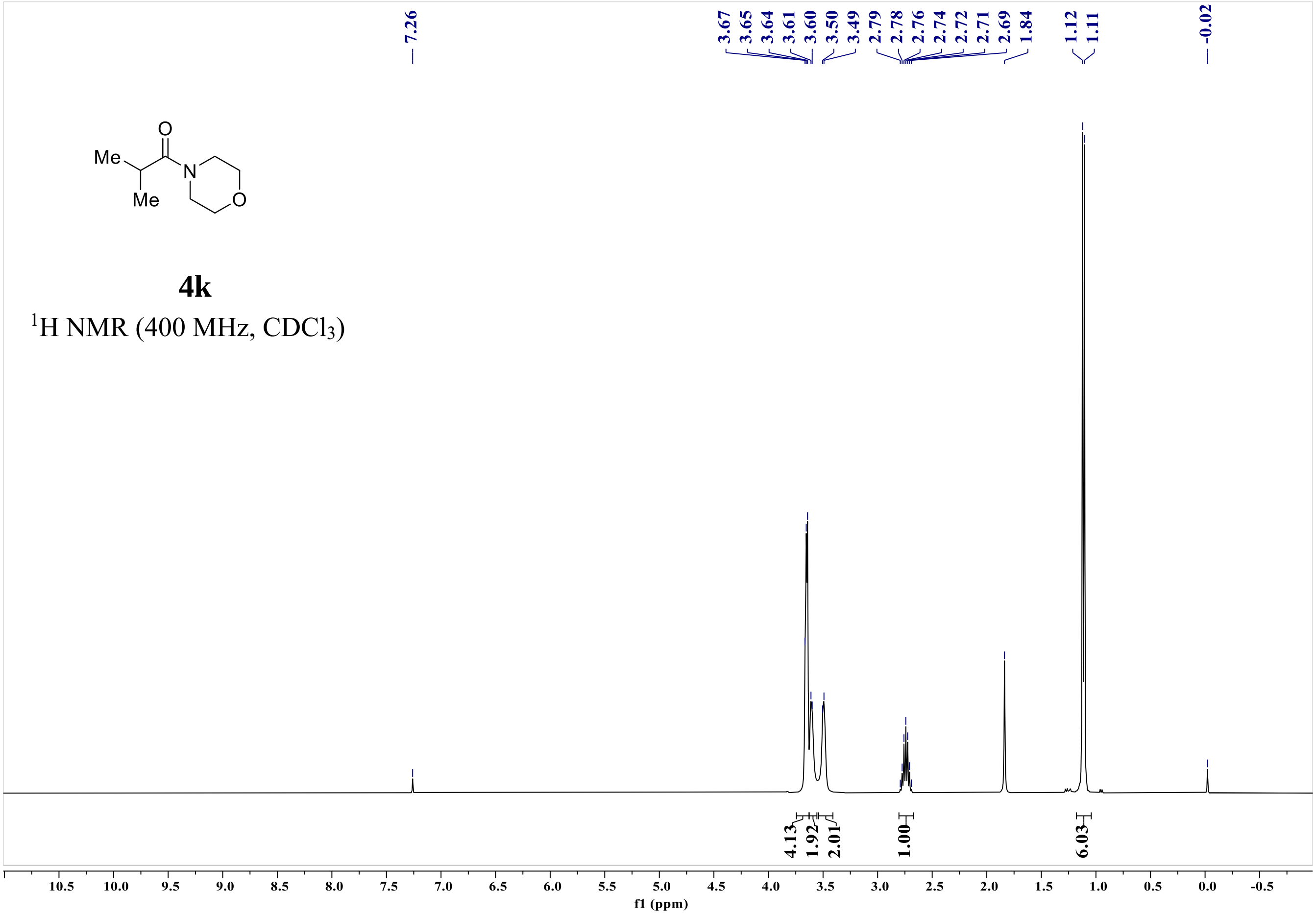
7.26
3.67
3.65
3.64
3.61
3.60
3.50
3.49
2.79
2.78
2.76
2.74
2.72
2.71
2.69
1.84
1.12
1.11
-0.02
Me
O
N
Me
O
4k
1H NMR (400 MHz, CDCl3)
4.13
1.92
2.01
1.00
6.03
10.5 10.0 9.5 9.0 8.5 8.0 7.5 7.0 6.5 6.0 5.5 5.0 4.5 4.0 3.5 3.0 2.5 2.0 1.5 1.0 0.5 0.0 -0.5
f1 (ppm)

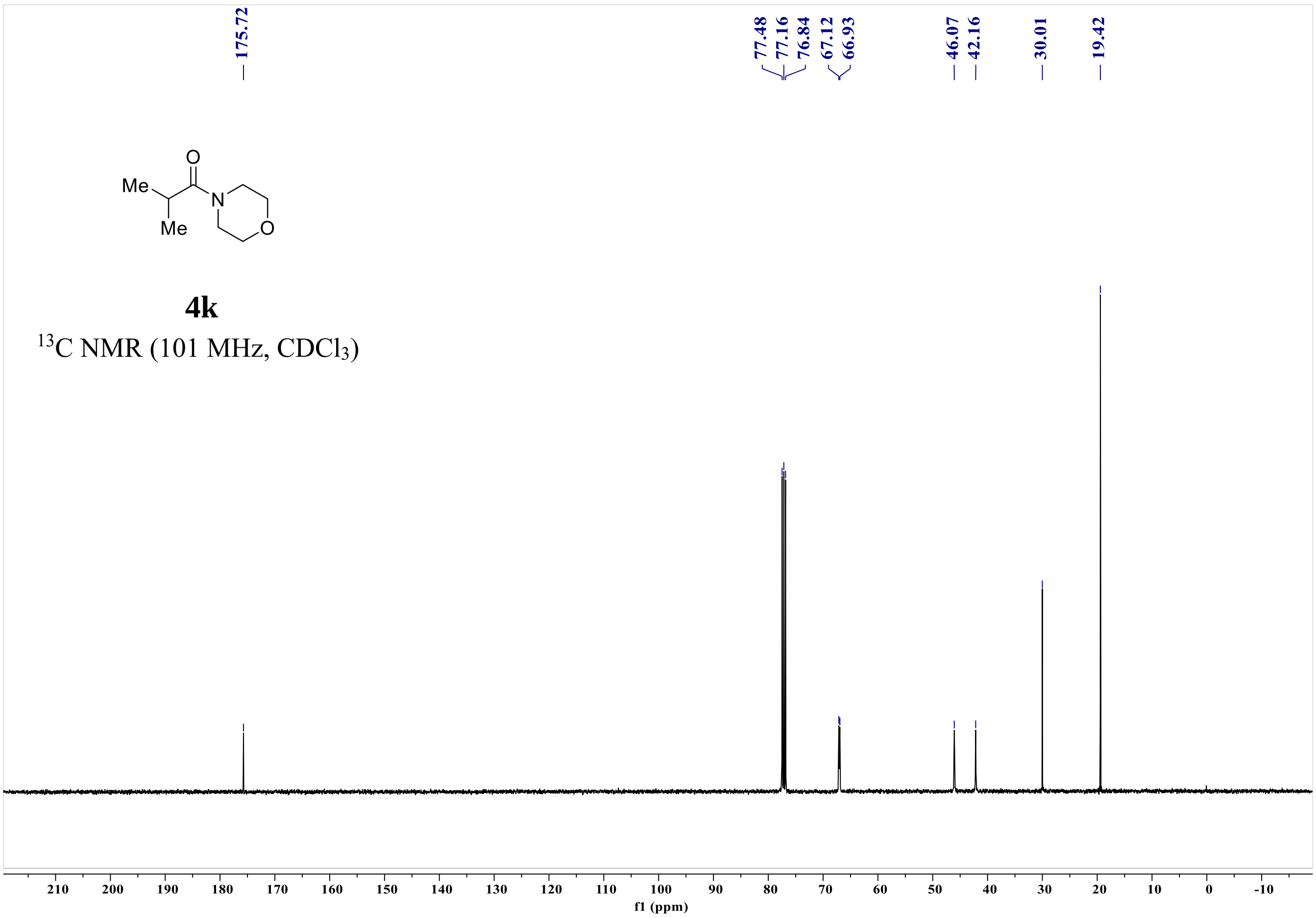
175.72
77.48
77.16
76.84
67.12
66.93
46.07
42.16
30.01
19.42
Me
O
N
Me
O
4k
13C NMR (101 MHz, CDCl3)
210 200 190 180 170 160 150 140 130 120 110 100 90 80 70 60 50 40 30 20 10 0 -10
f1 (ppm)

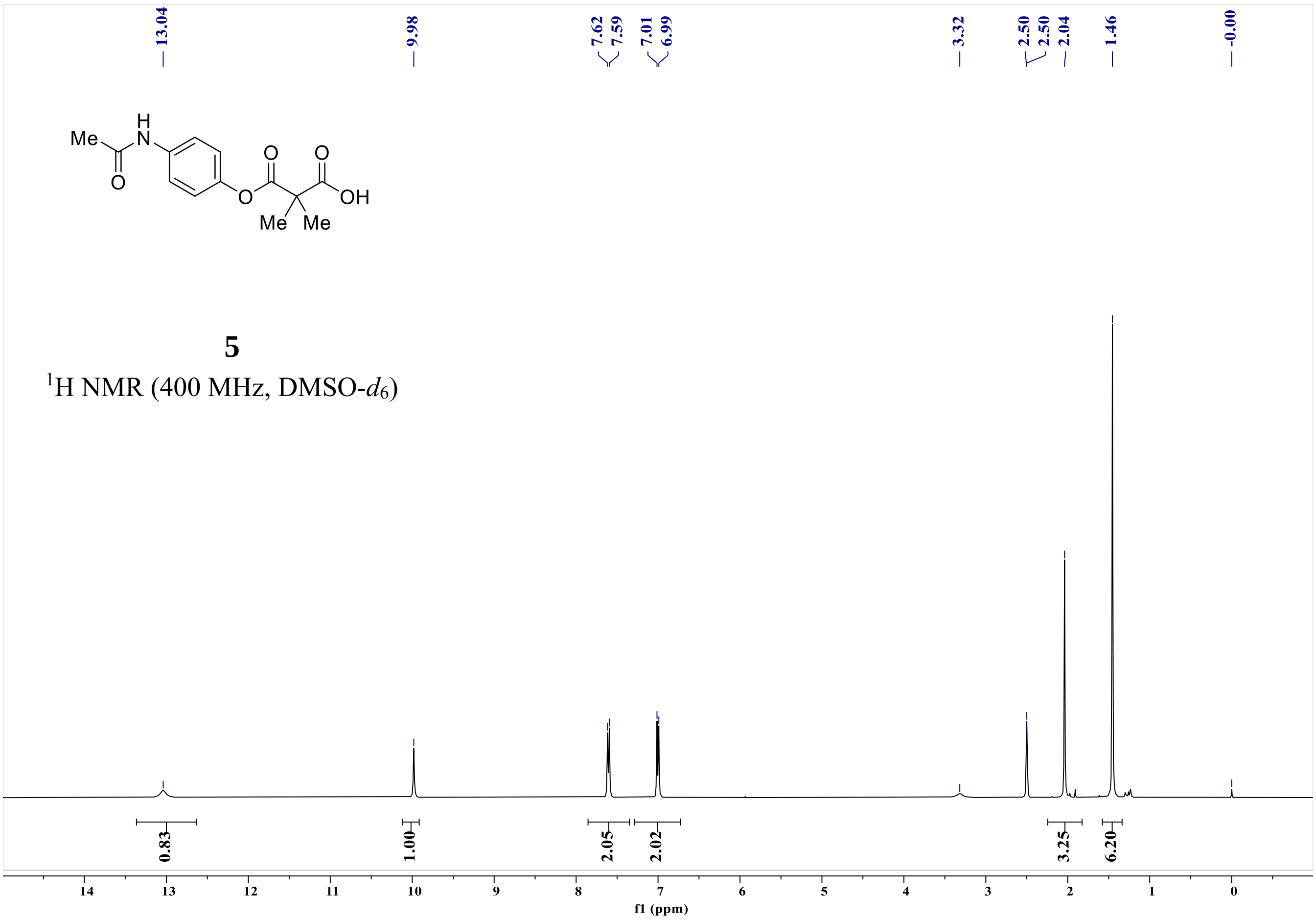
13.04
9.98
7.62
7.59
7.01
6.99
3.32
2.50
2.50
2.04
1.46
-0.00
Me
H
N
O
O
O
O
OH
Me Me
5
1H NMR (400 MHz, DMSO-d6)
0.83
1.00
2.05
2.02
3.25
6.20
14 13 12 11 10 9 8 7 6 5 4 3 2 1 0
f1 (ppm)

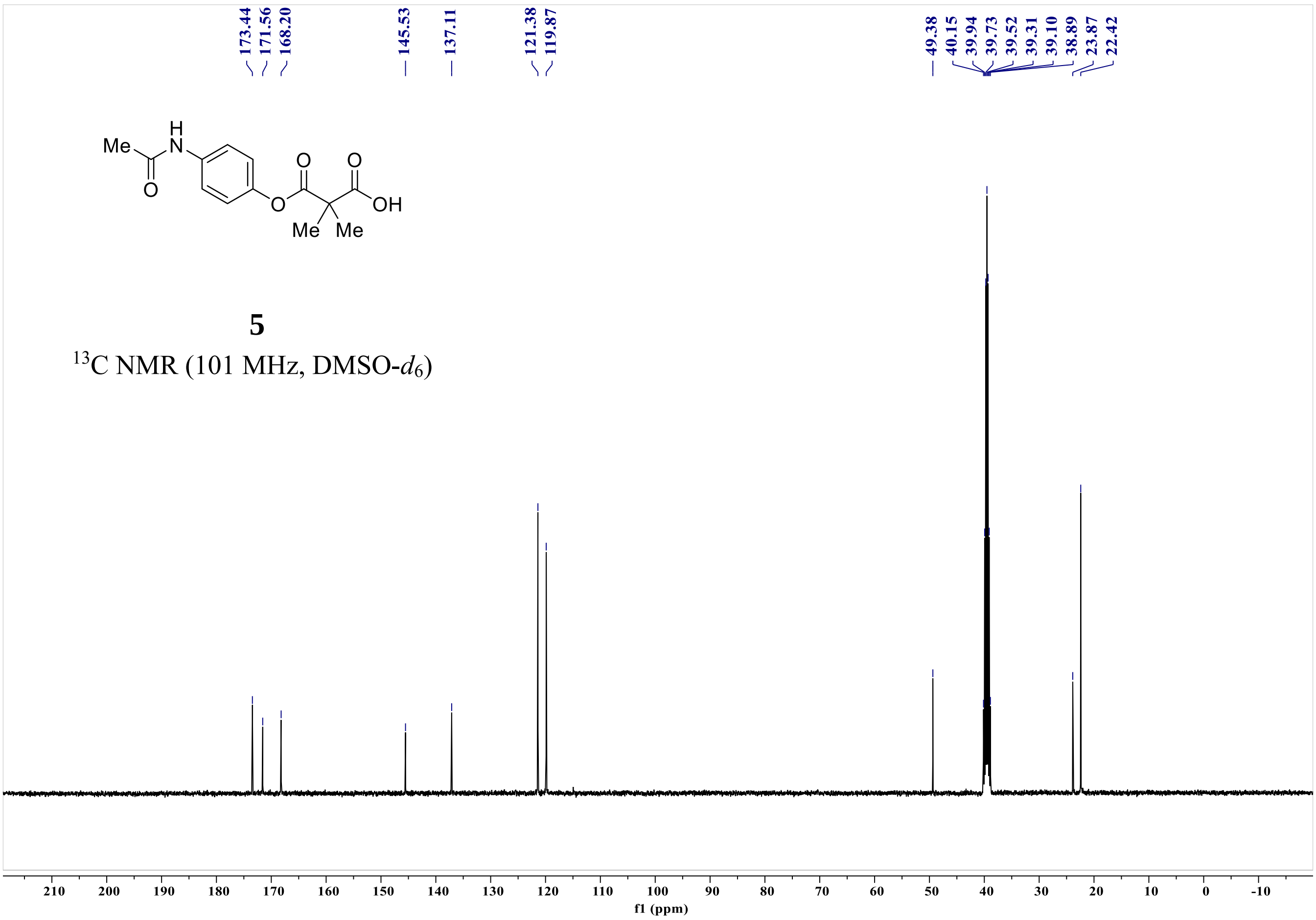
173.44
171.56
168.20
145.53
137.11
121.38
119.87
49.38
40.15
39.94
39.73
39.52
39.31
39.10
38.89
23.87
22.42
Me
H
N
O
O
O
O
OH
Me Me
5
13C NMR (101 MHz, DMSO-d6)
210 200 190 180 170 160 150 140 130 120 110 100 90 80 70 60 50 40 30 20 10 0 -10
f1 (ppm)

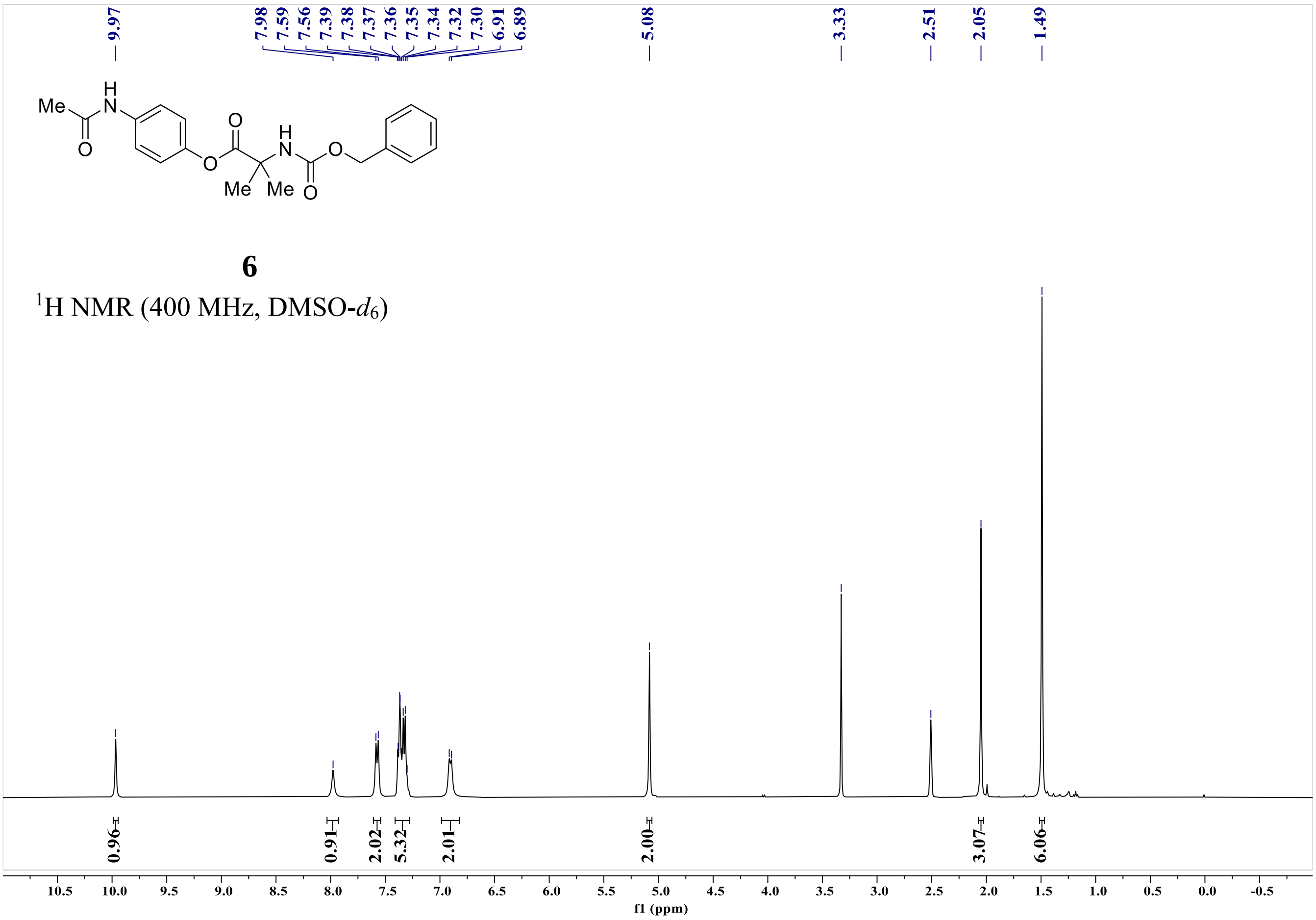
9.97
7.98
7.59
7.56
7.39
7.38
7.37
7.36
7.35
7.34
7.32
7.30
6.91
6.89
5.08
3.33
2.51
2.05
1.49
Me
H
N
O
O
O
H
N
O
O
Me
Me
O
6
^{1}H NMR (400 MHz, DMSO-d_6)
0.96
0.91
2.02
5.32
2.01
2.00
3.07
6.06
f1 (ppm)

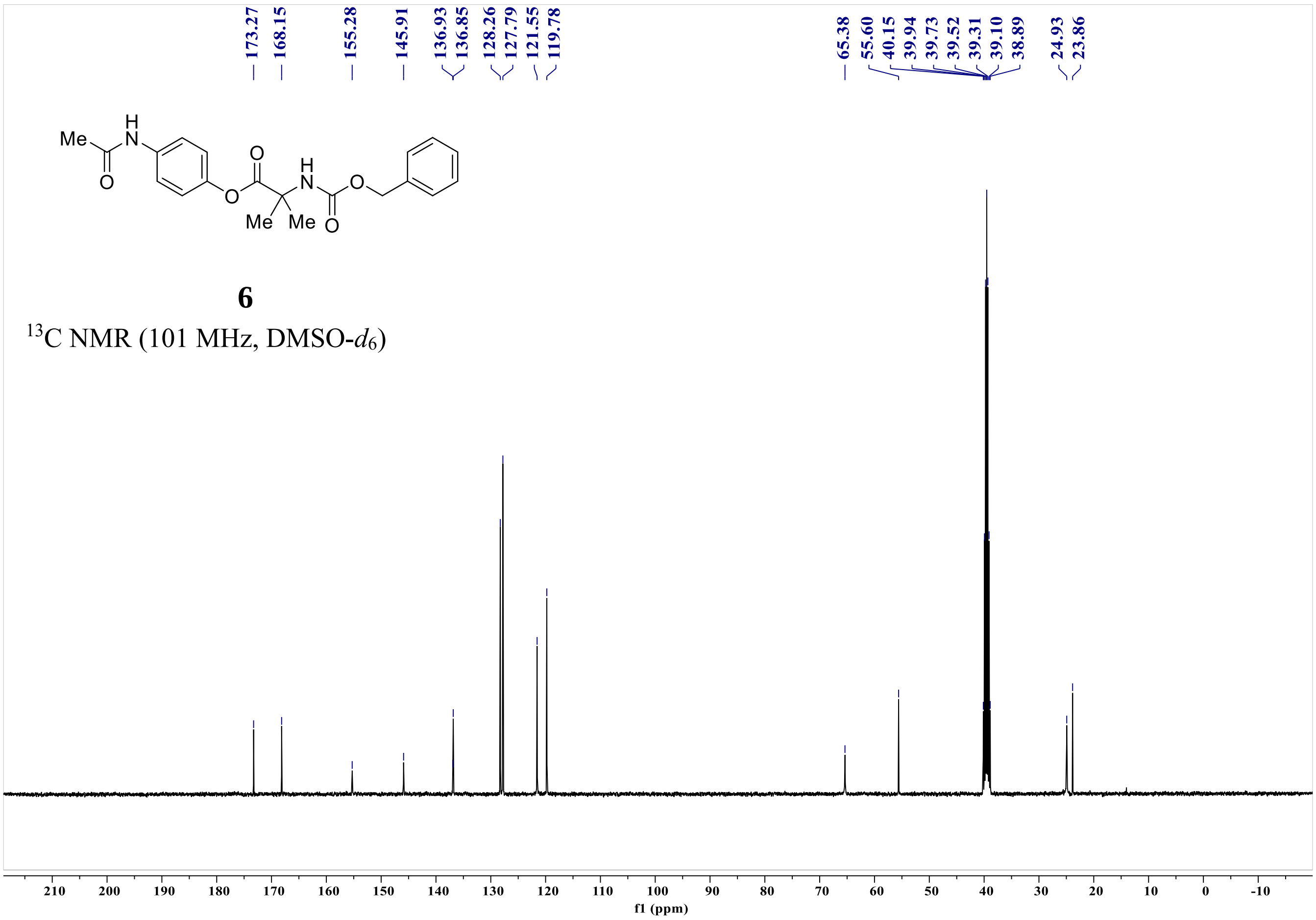
173.27
168.15
155.28
145.91
136.93
136.85
128.26
127.79
121.55
119.78
65.38
55.60
40.15
39.94
39.73
39.52
39.31
39.10
38.89
24.93
23.86
Me
H
N
O
O
O
H
N
O
O
Me
Me
O
6
^{13}C NMR (101 MHz, DMSO-d_6)
f1 (ppm)

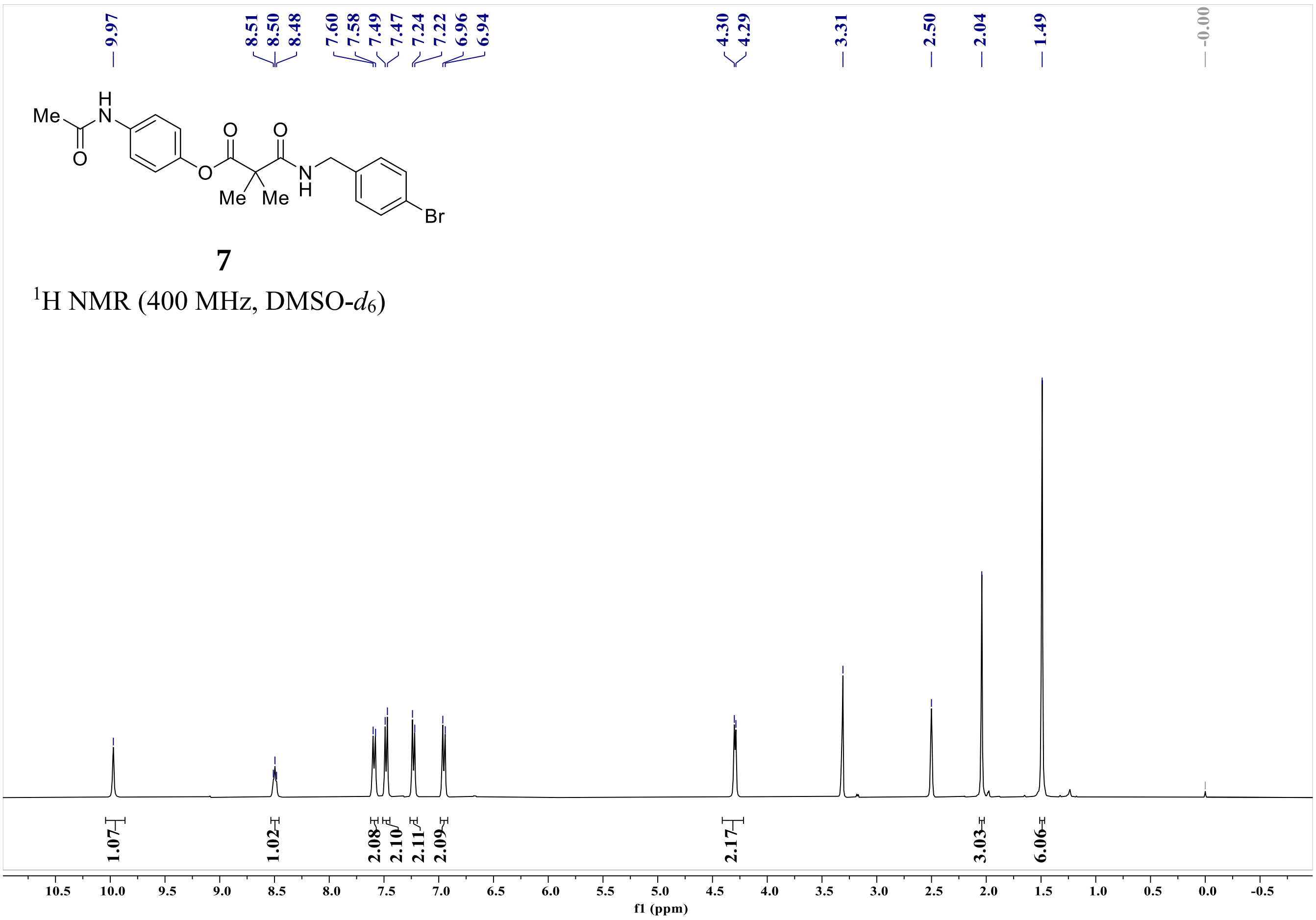
9.97
8.51
8.50
8.48
7.60
7.58
7.49
7.47
7.24
7.22
6.96
6.94
4.30
4.29
3.31
2.50
2.04
1.49
-0.00
Me
H
N
O
O
O
O
N
H
Me
Me
Br
7
^{1}H NMR (400 MHz, DMSO-d_6)
1.07
1.02
2.08
2.10
2.11
2.09
2.17
3.03
6.06
f1 (ppm)

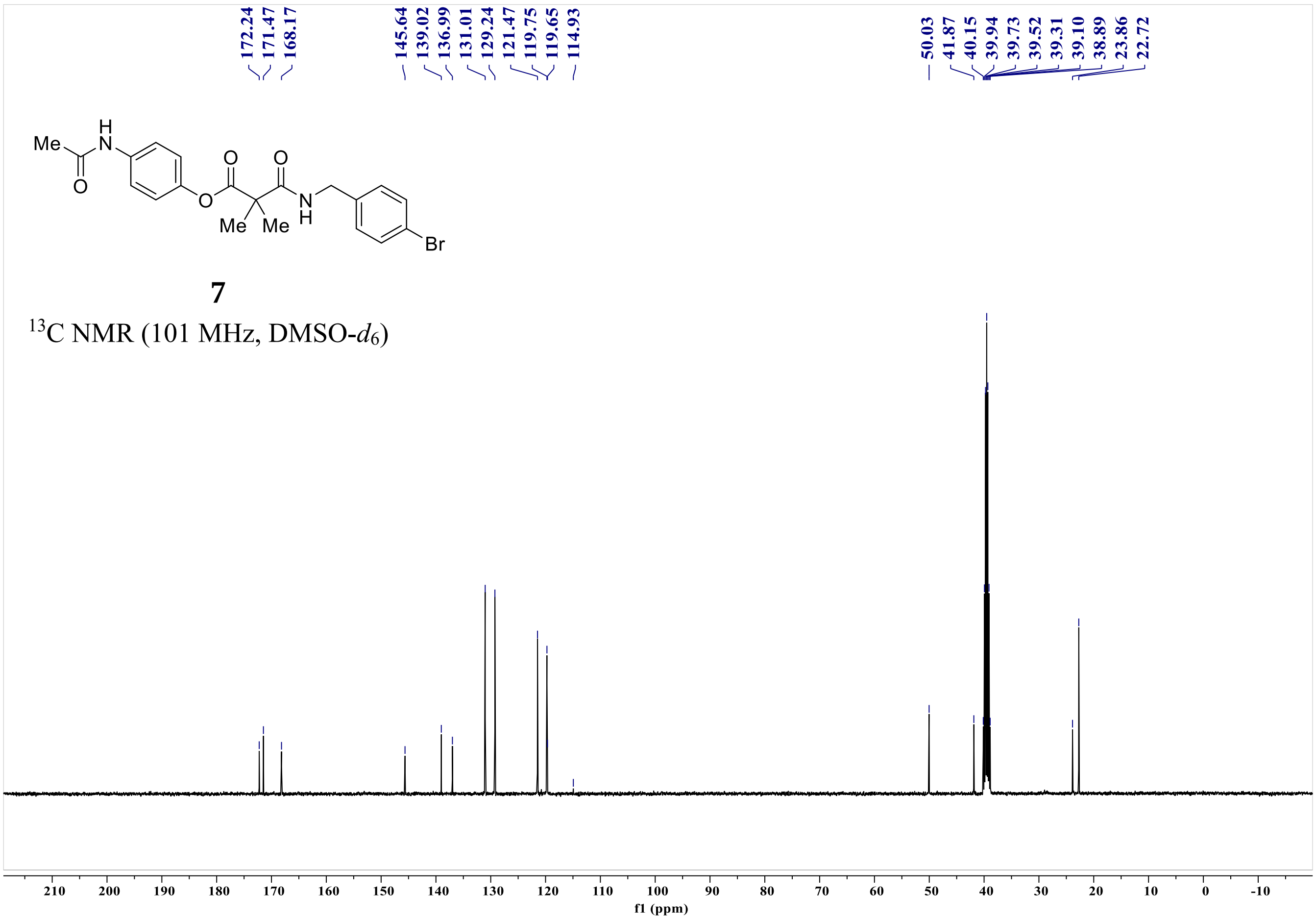
172.24
171.47
168.17
145.64
139.02
136.99
131.01
129.24
121.47
119.75
119.65
114.93
50.03
41.87
40.15
39.94
39.73
39.52
39.31
39.10
38.89
23.86
22.72
Me
H
N
O
O
O
O
N
H
Me
Me
Br
7
^{13}C NMR (101 MHz, DMSO-d_6)
f1 (ppm)

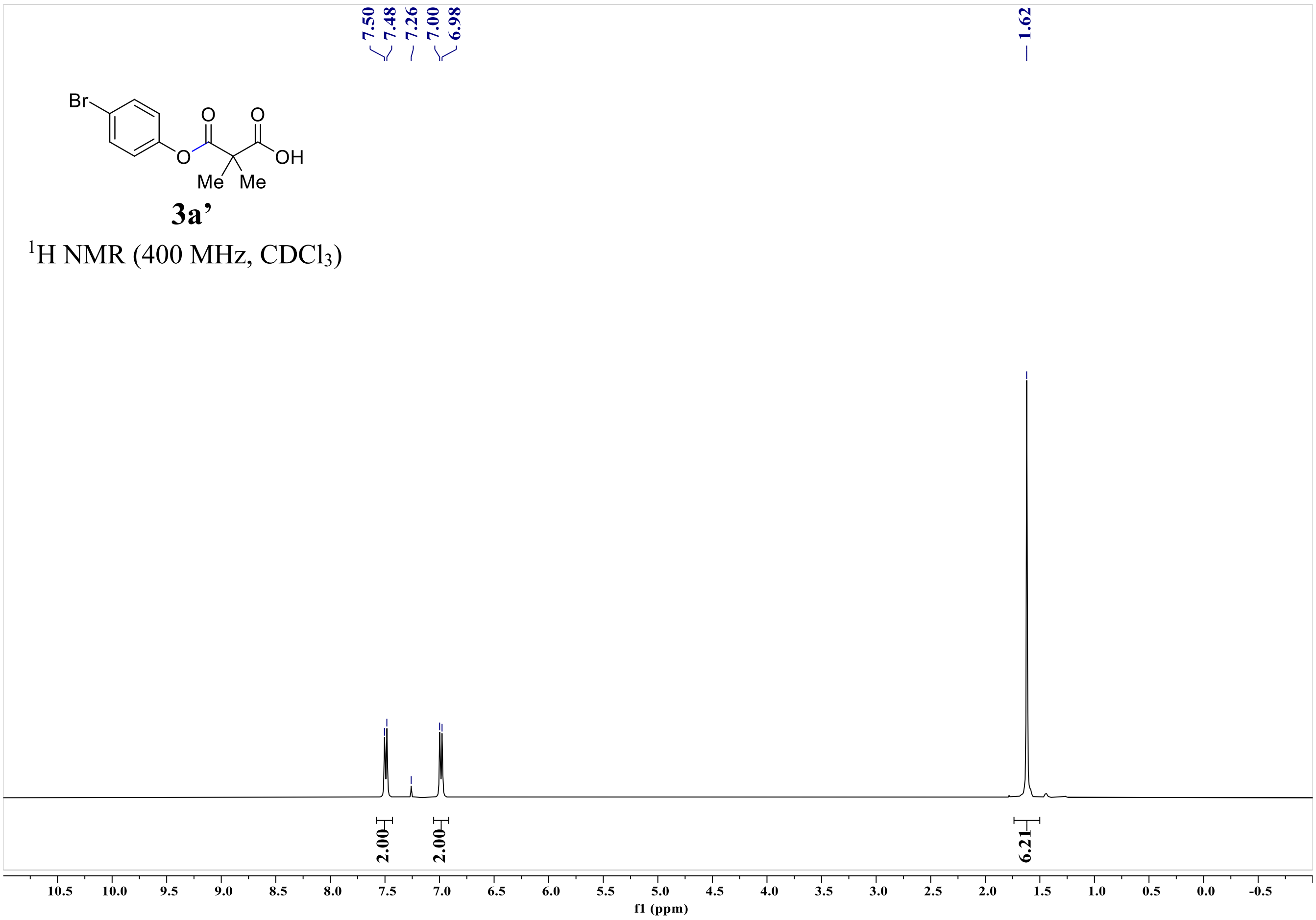
7.50
7.48
7.26
7.00
6.98
1.62
Br
O
O
O
OH
Me Me
3a'
^{1}H NMR (400 MHz, $CDCl_3$)
2.00
2.00
6.21
10.5 10.0 9.5 9.0 8.5 8.0 7.5 7.0 6.5 6.0 5.5 5.0 4.5 4.0 3.5 3.0 2.5 2.0 1.5 1.0 0.5 0.0 -0.5
f1 (ppm)

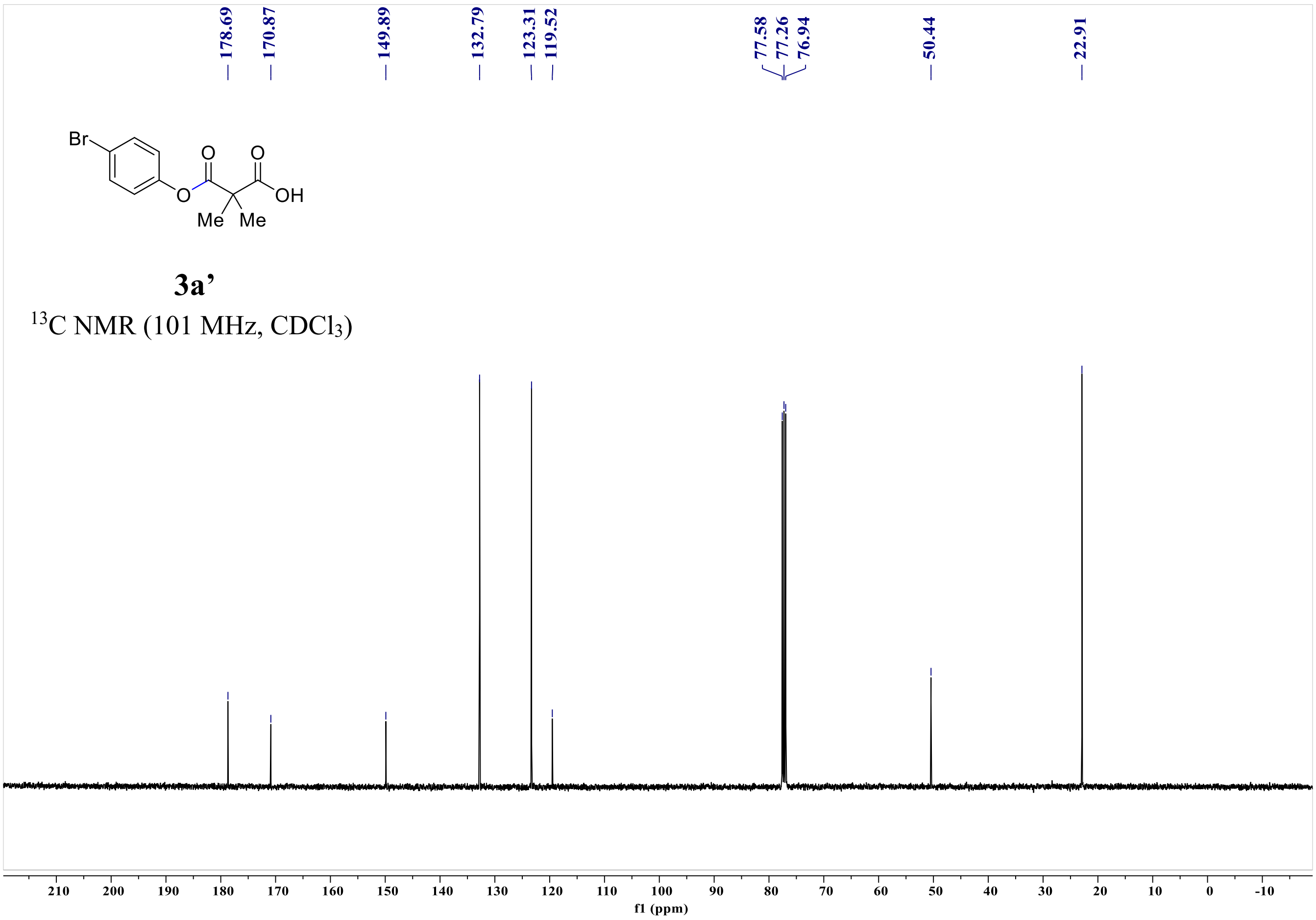
178.69
170.87
149.89
132.79
123.31
119.52
77.58
77.26
76.94
50.44
22.91
Br
O
O
O
OH
Me Me
3a'
^{13}C NMR (101 MHz, $CDCl_3$)
210 200 190 180 170 160 150 140 130 120 110 100 90 80 70 60 50 40 30 20 10 0 -10
f1 (ppm)
